# Discovery of a New Class of Cathepsin K Inhibitors in *Rhizoma Drynariae* as Potential Candidates for the Treatment of Osteoporosis

**DOI:** 10.3390/ijms17122116

**Published:** 2016-12-16

**Authors:** Zuo-Cheng Qiu, Xiao-Li Dong, Yi Dai, Gao-Keng Xiao, Xin-Luan Wang, Ka-Chun Wong, Man-Sau Wong, Xin-Sheng Yao

**Affiliations:** 1 Institute of Traditional Chinese Medicine & Natural Products, College of Pharmacy, Jinan University, Guangzhou 510632, China; qiu666@stu2014.jnu.edu.cn (Z.-C.Q.); daiyi1004@163.com (Y.D.); xiaogk@yaolab.org (G.-K.X.); 2 Department of Applied Biology and Chemical Technology, The Hong Kong Polytechnic University, Hung Hom, Kowloon, Hong Kong, China; bcxldong@polyu.edu.hk (X.-L.D.); aguesses@yahoo.com.hk (K.-C.W.); 3 Shenzhen Key Laboratory of Food Biological Safety Control, Shenzhen 518057, China; 4 Translational Medicine R&D Center, Institute of Biomedical and Health Engineering, Shenzhen Institutes of Advanced Technology, Chinese Academy of Sciences, Shenzhen 518000, China; xl.wang@siat.ac.cn; 5 State Key Laboratory of Chinese Medicine and Molecular Pharmacology (Incubation), Shenzhen 518057, China

**Keywords:** in silico target fishing, osteoporosis, Cathepsin K, *Rhizoma Drynariae*, Kushennol F, Sophoraflavanone G, RAW264.7 cells, osteoclasts, bone resorption

## Abstract

*Rhizoma Drynariae* (RD), as one of the most common clinically used folk medicines, has been reported to exert potent anti-osteoporotic activity. The bioactive ingredients and mechanisms that account for its bone protective effects are under active investigation. Here we adopt a novel in silico target fishing method to reveal the target profile of RD. Cathepsin K (Ctsk) is one of the cysteine proteases that is over-expressed in osteoclasts and accounts for the increase in bone resorption in metabolic bone disorders such as postmenopausal osteoporosis. It has been the focus of target based drug discovery in recent years. We have identified two components in RD, Kushennol F and Sophoraflavanone G, that can potentially interact with Ctsk. Biological studies were performed to verify the effects of these compounds on Ctsk and its related bone resorption process, which include the use of in vitro fluorescence-based Ctsk enzyme assay, bone resorption pit formation assay, as well as Receptor Activator of Nuclear factor κB (NF-κB) ligand (RANKL)-induced osteoclastogenesis using murine RAW264.7 cells. Finally, the binding mode and stability of these two compounds that interact with Ctsk were determined by molecular docking and dynamics methods. The results showed that the in silico target fishing method could successfully identify two components from RD that show inhibitory effects on the bone resorption process related to protease Ctsk.

## 1. Introduction

With the increase in aging population, osteoporosis is becoming one of the major public health problems that poses a significant medical and socioeconomic burden to the society [1]. It is a systemic metabolic disease characterized by low bone mass and deterioration of microarchitecture as a result of the imbalance of bone formation and bone resorption [2]. Currently, the actions of available therapeutics mainly include improvement of bone formation or inhibition of bone resorption. Parathyroid hormone PTH (1–84) is the major bone anabolic agent that can stimulate bone formation. Anti-resorptive agents, such as bisphosphonates, selective estrogen receptor modulators (SERMs), and the most recently approved denosumab, are proven to reduce bone resorption via different mechanisms of action [3]. Considering that postmenopausal osteoporosis accounts for 80% of incidence of osteoporosis and that estrogen-deficiency induced increase in bone resorption contributes to significant bone loss, most of the osteoporotic drugs are designed to suppress bone resorption process. However, unavoidable side effects and limitations are reported with the use of commonly used clinical agents. For example, PTH (1–84) was reported to cause hypercalcaemia [1], treatment with SERMs such as raloxifene can potentially induce thromboembolic disease [4]. In addition, recent reports indicated that many anti-osteoporotic drugs show limited efficacy due to the coupling effect between bone resorption and bone formation, i.e., the inhibition of bone resorption is accompanied by the inhibition of bone formation and vice versa [2]. Thus, there is a strong demand for developing new therapeutics with novel mechanisms of action for management of osteoporosis.

Traditional Chinese Medicine (TCM) has been used for treatment of osteoporosis for thousands of years in China [5,6]. *Rhizoma Drynariae* (RD), also named as “Gu-Sui-Bu” in folk medicine, is one of the most frequently used herbs in clinical formulas to treat bone related diseases [7,8,9]. Our previous publications have reported the osteoprotective effects of RD as well as its active ingredients. The crude extract of RD could improve bone formation in pre-osteoblastic MC3T3-E1 and human osteoprecursor MG63 cells [7,10]. Total flavonoids in RD were found to significantly enhance bone mineral density in an ovariectomized rat model. Numerous compounds have been isolated from RD and some of them have been proven to exert osteoprotective effects in both in vitro and in vivo studies [11,12,13,14,15]. However, only a few reports mentioned the targets which mediating the bone protective actions of RD. Jeong et al. reported that RD crude extracts suppressed bone resorption via inhibiting Cathepsin K [16]. Other investigators reported that naringin and its metabolite naringenin are the main active ingredients of RD that showed higher binding affinity to estrogen receptor-α (ER-α) than ER-β in yeast two-hybrid experiments [17]. Therefore, the exact acting targets of compounds identified from RD are still needed to be fully determined.

Early drug discovery strategy mainly aimed to identify “magic bullets”; i.e., broadly screening compounds for therapeutic targets related to specific diseases [18]. However, this ‘one to one’ strategy is challenging due to the lack of efficacy and clinical safety or toxicology of the identified compounds [19], so new approaches are sought in drug-discovery strategies. The emergence of a novel concept of poly-pharmacology, which emphasizes one or multiple drug(s) for treatment of diseases via specific binding to multiple targets within a network, has attracted much attention recently [20]. This systemic biological concept inspired further drug discovery in DR, since evidence hinted that multiple targets may involved in its anti-osteoporosis activity [13,16,17]. The premise of ‘poly-pharmacology’ study was the clear illustration of the interaction between small molecules and their therapeutic targets. However, it will always be a time consuming and costly process until the development of in silico target fishing method. As a novel computational approach emerging in recent years to reveal target profile of small molecules [21], in silico target fishing is characterized by its ability to rapidly identify specific target of query molecule from a collection of proteins in a cost-effective way. Recent target fishing includes four primary approaches: chemical similarity searching, data mining/machine learning, bioactivity spectra, and panel docking [22]. With the explosive increase of the available biologically annotated chemical database, it is now possible to predict the potential targets of query compounds with a relatively high hit rate. Recent published reports [21,23] have employed this method to successfully identify targets in their studies. Lounkine et al. adopted a ‘Similarity Ensemble Approach’ to predict the activity of 656 marketed drugs on 73 unintended side-effect targets. They have successfully identified and validated many unanticipated drug-target associations that exhibit binding affinities from 1 to 30 nM [24]. Muller et al. [25] utilized receptor-based docking method to screen for 2150 active sites of drug targets using their self-developed protein target library (also known as sc-PDB (Protein Database Bank)). They have obtained potential targets of five representative compounds with commonly shared 1,3,5-triazepan-2,6-dione scaffold. In vitro tests confirmed that secreted phospholipase A2 (sPLA2) was the true target out of the five predicted targets [25]. These examples indicate that in silico target fishing is an effective method to elucidate the target profiles of small molecules which modulate cellular phenotypes.

In the present study, a novel in silico target fishing method [26] was applied to reveal the target profile of the compounds isolated from *Rhizoma Drynariae* (RD), then the compounds that interacted with novel target were selected for further validation by in vitro biological assay. Moreover, the binding mode of the compound-target interaction was investigated by molecular docking and dynamics. Thus, the ingredients in RD exhibiting anti-osteoporotic activity via acting on the novel target were discovered.

## 2. Results

### 2.1. In Silico Target Fishing

The results of target prediction of 48 query compounds from RD (Table S1) generated by PredictFX 1.1 (Tripos International, St. Louis, MO, USA) including their hitting targets, predicted affinity value, and neighbouring compounds (reference compounds retrieved from database which share the same target) are annotated with biological data and are summarized as supplementary information (Table S2 and Table S3). Although the results are composed of large scale data containing targets involved in various diseases, only osteoporosis-related targets were the focus of the present study. Through surveying the literature related to these compounds and their biological activities, the reliability of the predicted results given by PredictFX 1.1 was further confirmed.

**Figure 1:**
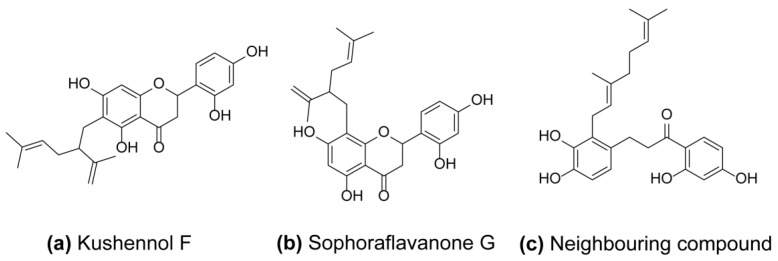
The chemial structures of (**a**) Kushennol F; (**b**) Sophoraflavanone G; and (**c**) Neighbouring compound acquired by prediction.

We have identified two compounds Kushennol F (KF) and Sophoraflavone G (SG) (Figure 1a,b) in which their hitting target was an osteoclast-related target, Cathepsin K (Ctsk), using the above method. The details of the predicted target information of these two hitting compounds and the information about their neighbouring compounds such as similarity rate, measured IC50, and original source were shown in Table 1.

### 2.2. Kushennol F (KF) and Sophoraflavone G (SG) Directly Inhibited the Proteolytic Activity of Cathepsin K

**Figure 2:**
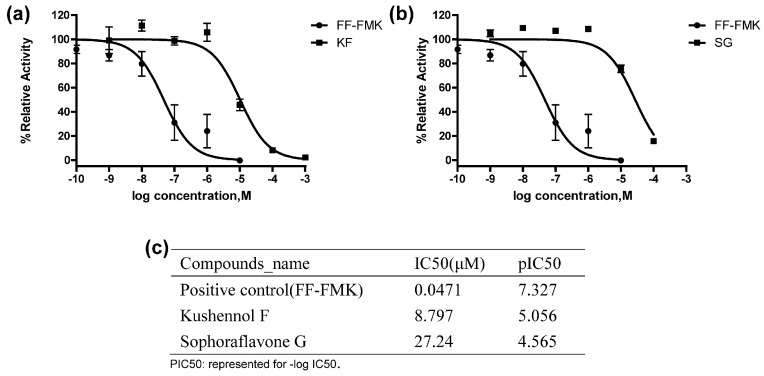
(**a**–**c**) Inhibitory effects of FF-FMK (Phe-Phe-Fluoromethylketone), Kushennol F (KF), and Sophoraflavone G (SG) on Cathepsin K activities.

To determine if Cathepsin K is the target of compounds KF and SG, their effects on the activities of recombinant protein Cathepsin K were determined. As shown in Figure 2, FF-FMK (Phe-Phe-Fluoromethylketone) as the positive control, dose-dependently inhibited Ctsk activities and its IC50 was calculated to be 0.047 μΜ. Similarly, both KF and SG could inhibit the activities of Ctsk in a dose-dependent manner and the IC50 of KF and SG was calculated to be 8.79 and 27.2 μM, respectively. The results clearly indicate that Ctsk is the direct molecular target of KF and SG.

### 2.3. Kushennol F and Sophoraflavone G Suppressed RANKL-induced Osteoclastogenesis of RAW264.7

**Figure 3:**
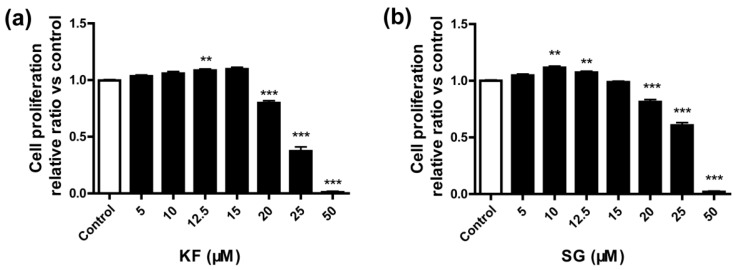
Evaluation of the cytotoxic effects of (**a**) Kushennol F (KF) and (**b**) Sophoraflavone (SG) in RAW264.7 pre-osteoclastic cells. Cell viabilities were measured by MTS assay. Data are means ± SEM (*n* = 5) and ** *p* < 0.01 and *** *p* < 0.001 versus vehicle group.

To determine if KF and SG inhibit the bone resorption process at a cellular level, their cytotoxic effects on mouse macrophage-derived RAW264.7 pre-osteoclastic cells were first evaluated. As shown in Figure 3, KF and SG did not exert cytotoxic effect until the concentration reached 20 μM. Thus, the concentrations of KF and SG for subsequent studies on osteoclast differentiation and function were chosen to be 5 and 12.5 μM to avoid cytotoxic effects of the two compounds.

**Figure 4:**
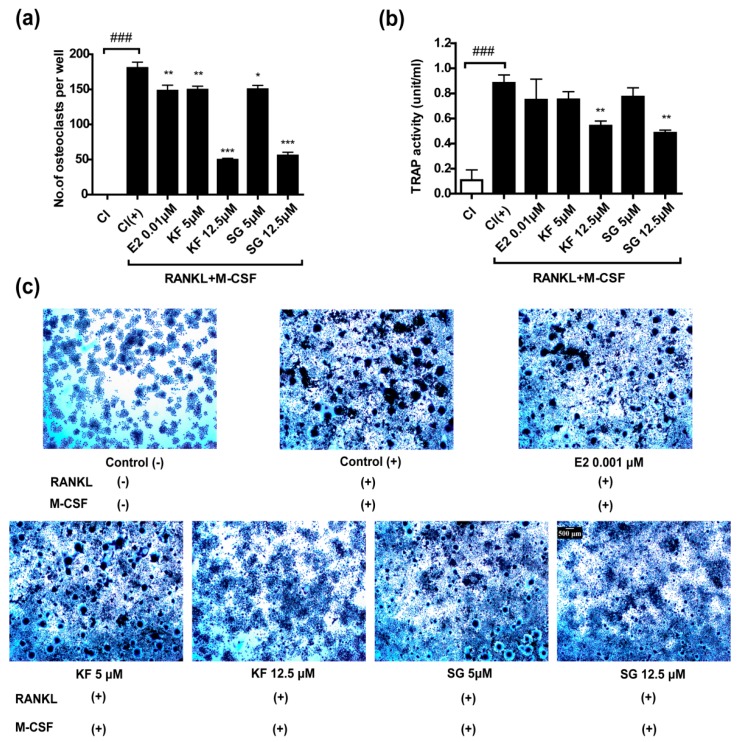
Inhibitory activities of Kushennol F (KF) and Sophoraflavone G (SG) on RANKL-induced RAW264.7 osteoclastogenesis. (**a**) Number of mature osteoclasts TRAP-positive multi-nucleated giant cells were counted as osteoclast-like cells (>3 nucleus); (**b**) Tartrate-resistant acid phosphatase (TRAP) activity; (**c**) TRAP staining photographs captured under light microscope. Data are means ± SEM (*n* = 3). ^###^ *p* < 0.001 versus Control (−); * *p* < 0.05, ** *p* < 0.01, *** *p* < 0.001 versus Control (+).

Pre-osteoclastic RAW264.7 differentiated into osteoclast-like, multinucleated cells (MNCs) in the presence of receptor activator of nuclear factor-κB ligand (RANKL) and macrophage colony-stimulating factor (M-CSF). The effects of KF and SG on the formation of multinucleated cells (MNCs) in RANKL-induced RAW264.7 cells were shown in Figure 4. 17-β estradiol (10^−8^ M) as the positive control significantly reduced the number of tartrate resistance acid phosphatase (TRAP, a marker of osteoclast and macrophage activation)-positive MNCs (*p* < 0.05, vs. control, Figure 4a). Similarly, KF and SG at 5 and 12.5 μM exhibited inhibitory effects on the formation of TRAP-positive MNCs (Figure 4a), 12.5 μM of KF and SG significantly inhibited the formation of osteoclast by 72% and 69%, respectively (*p* < 0.001, vs. control, Figure 4a). The effects of KF and SG on enzymatic activities of TRAP in RANKL-induced RAW264.7 cells were also determined. KF and SG at 12.5 μM significantly inhibited TRAP activities in RANKL-induced RAW264.7 cells by 39% and 45%, respectively (*p* < 0.01, vs. control, Figure 4b). The inhibitory effects of KF and SG were also visualized by TRAP-Staining RANKL-induced RAW264.7 cells as shown in Figure 4c.

### 2.4. Kushennol F and Sophoraflavone G Significantly Inhibited Bone Resorption Pit Formation

**Figure 5:**
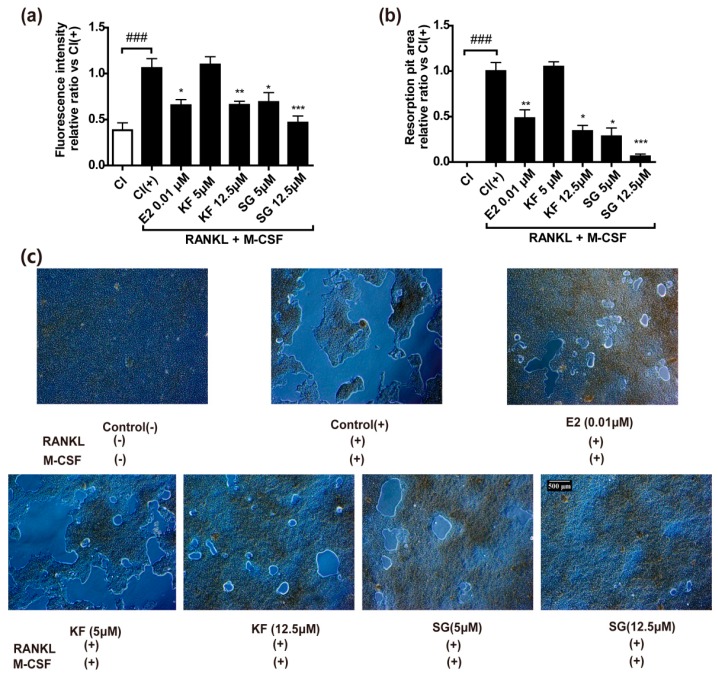
Both KF and SG showed potent inhibitory activities on bone resorption in RANKL-induced RAW264.7 osteoclastic cells. (**a**) The fluosrescence intensity of medium in Fluoresceinamine-labeled chondroitin polysulfate (FACPS)/calcium phosphate (Ca-P)-coated wells after six days of RANKL induction; (**b**) The resorption pit areas. Areas of the formed pit was analyzed and calculated by software Image-Pro-Plus 6.0; (**c**) Representative micrographs of bone resorption pits from each group in day 6 was shown. Control (−) represents for group which was treated with only 1% ethanol for six days; Control (+) stands for group in which cells were induced with 1% ethanol, 100 ng/mL RANKL and 30 ng/mL M-CSF for six days; 17-β Estradiol (E_2_, 10^−7^M), KF (5, 12.5 μM), and SG (5, 12.5μM) were treatment groups in which induced cells (by 100 ng/mL RANKL and 30 ng/mL M-CSF.) were treated for three days. Results were obtained from two independent experiments in triplicate and expressed as mean ± SEM (Standard error of the mean). ^###^ *p* < 0.001 versus Control (−); * *p* < 0.05, ** *p* < 0.01, *** *p* < 0.001 versus Control (+).

Our results indicated that KF and SG exerted inhibitory effects on Ctsk, a key enzyme involved in bone resorption process. To further confirm the direct effects of KF and SG on osteoclastic bone resorption in vitro, their effects on bone resorption pit formation were evaluated using fluoresceinamine-labeled chondroitin sulfate (FACS) labeled calcium phosphate (Ca-P)-coated plate. RAW264.7 cells grown on FACS labeled (Ca-P)-coated plate and induced by RANKL and M-CSF were subjected to treatment with KF and SG for three days. Figure 5a shows that RANKL induced the increase in fluorescence intensity in medium of RAW264.7 cells (*p* < 0.001, vs. control without RANKL), indicating that an increase in osteoclast activities and release of fluoresceinamine-labeled chondroitin sulfate (FACS) from coated plate. 17β-Estradiol (E_2_, 10^−7^ M) significantly inhibited the increase in osteoclastic activities induced by RANKL in RAW264.7 cells (*p* < 0.05, vs. control with RANKL). KF inhibited the increase in osteoclastic activities at 12.5 μM, but not at 5 μM, by 50% in RAW264.7 cells (*p* < 0.01, vs. control with RANKL). In contrast, SG could significantly inhibit the increase in osteoclastic activities at 5 μM by 50% (*p* < 0.05, vs. control with RANKL) and further suppressed the activities to baseline at 12.5 μM (*p* < 0.001, vs. control with RANKL) in RAW264.7 cells. The areas of the bone resorption pits formed in response to treatment with KF and SG were also determined and were shown in Figure 5b. E_2_, KF at 12.5 μM, and SG at both 5 and 12.5 μM significantly decreased the areas of bone resorption pits formed by RANKL-induced RAW264.7 cells (*p* < 0.05, vs. control with RANKL). The morphological changes of bone resorption pit areas under light microscope were shown in Figure 5c.

### 2.5. Molecular Docking Analysis

**Figure 6:**
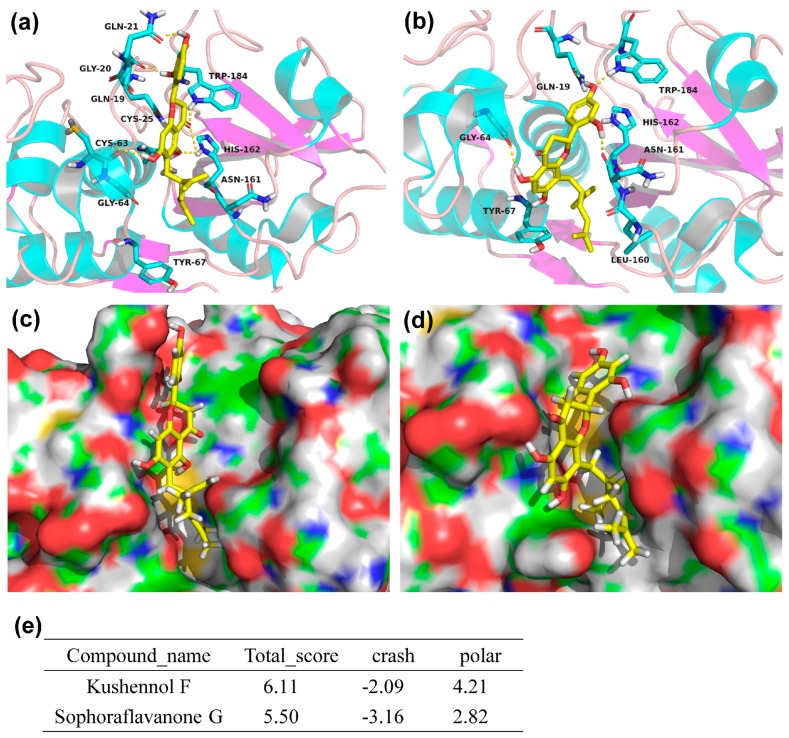
Predicted binding mode of KF and SG to the active site of Ctsk (PDB entry: 3KWZ). The structures of compounds were represented in stick model with color (yellow: carbon atom, red: oxgen atom and white: hydrogen atom). The binding mode of KF (**a**) and SG (**b**) in the active site of Ctsk. The protein was shown in the cartoon representation, the key residues in the active site were also represented as stick model with color (blue, red, and gray), the yellow dashed line denoted protein-ligand H-bonding interactions. The docking pose of KF (**c**) and SG (**d**) located in active site of Ctsk. The protein was illustrated by a solid surface. (**e**) The Docking Scores as acquired by Surflex-dock.

To further characterize the interaction of KF and SG with Ctsk, molecular docking studies of the two compounds with Ctsk were performed. The details of docking analysis were shown in Figure 6. KF and SG were shown to form several H-bonding interactions with the binding site of Ctsk. For KF (Figure 6a), hydrogen bonds were predicted to form between the compound and several key residues in the active site of Ctsk, the details were as follows: one hydrogen bonds (H–B) with GLN-21, GLY-20, CYS-63, TRP-184, and two H–B with HIS-162, respectively. Similarly, for SG (Figure 6b), hydrogen bonding interaction between the compound and several key residues in Ctsk were predicted as follows: one H-bonding with GLN-19, TRP-184, GLY-64, and ASN-161, respectively. The structure of KF (Figure 6c) and SG (Figure 6d) could also fit well into the groove of Cathespin K. The docking scores (Figure 6e) were given by SYBYL Surflex-dock. “Total_score” represents the binding affinity value p*K*d (−log*K*d; *K*d: dissociation constant). Crash score indicates the degree of inappropriate penetration by the ligand into the protein and of interpenetration (self-clash) between ligand atoms that are separated by rotatable bonds. “Crash” scores close to 0 are favorable, negative numbers indicate penetration. “Polar” means contribution of the polar interactions to the total score. In this study, KF and SG obtained total scores of 6.11 and 5.50, respectively; which are just within one log error when compared with experimentally determined PIC50 (−logIC50) of KF (PIC50: 5.056) and SG (PIC50: 4.57) in our validated assay. These results also in part illustrated the reliability of molecular docking methods.

### 2.6. Molecular Dynamic(MD) Simulations

**Figure 7:**
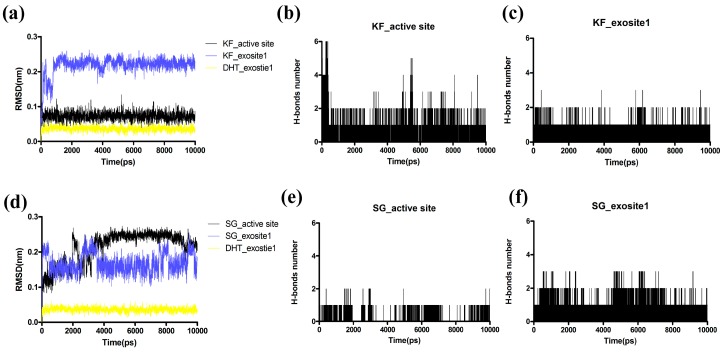
(**a**) and (**d**) Root mean square deviations (RMSD) of KF, SG and DHT (reference compound) during the 10 ns of MD simulation. The number of H-bonds of (**b**) KF and (**e**) SG interact with the active site of Ctsk. The number of H-bonds of (**c**) KF and (**f**) SG interacting with the exosite 1 of Ctsk.

**Figure 8:**
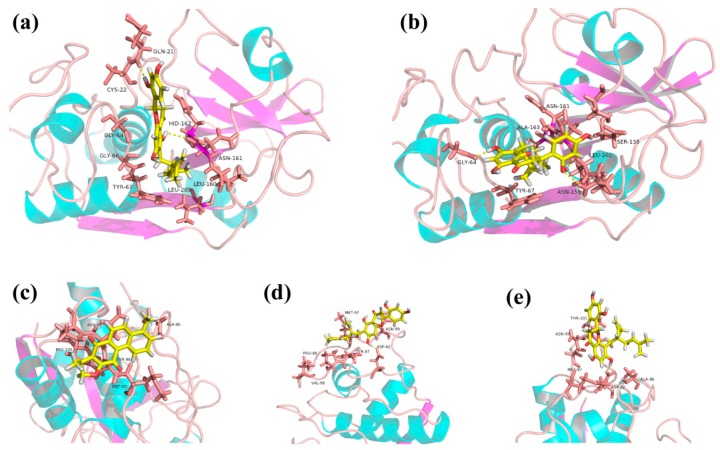
Binding modes of KF and SG in the active site or exosite 1 of Ctsk after 10 ns of MD simulation. The structures of compounds were represented in stick model with color (yellow: carbon atom, red: oxgen atom and white: hydrogen atom). The protein was shown in the cartoon representation, the key residues in the active site were also represented as stick model with color (blue, red, and gray), the yellow dashed line denoted protein-ligand H-bonding interactions. (**a**) KF bound to active site of Ctsk; (**b**) SG bound to active site of Ctsk; (**c**) DHT (as a reference compound) bound to the exosite1 of Ctsk; (**d**) KF bound to the exosite1 of Ctsk; (**e**) SG bound to the exosite1 of Ctsk.

To determine the most stable binding pocket of KF and SG in Ctsk, conformational changes between Ctsk and two compounds were analyzed by MD dynamics simulations via the protocol described in the method section below. RMSD (root mean square deviations) from the initial conformation was used to evaluate the dynamic stability of a system as it is a global measure of protein fluctuations. As shown in Figure 7, *p*-dihydrotanshinone (DHT) as a reference compound as shown in (Figure 7a,d) was reported to bind to exosite 1 [27] and its RMSD fluctuations remained relatively low. However, KF (Figure 7a) and SG (Figure 7d) complex with exosite 1 of Ctsk exhibited higher RMSD fluctuation, RMSD of SG-exostite1 was unable to reach its dynamic equilibrium during the whole 10 ns simulation. On the contrary, both KF (Figure 7a) and SG (Figure 7d) could reach its stability when complexed with the active site even though the RMSD of SG-active site fluctuated sharply at the first 4 ns. Such a phenomenon is in agreement with the experimental results that the SG exhibits a more potent IC50 value than KF in inhibiting Ctsk activities. Moreover, the number of hydrogen bonds and their occupancies during the MD simulations were determined. KF was shown to form 2 H-bonds with active site (Figure 7b) more frequently than exosite1 (Figure 7c) during the whole 10ns simulation. However, SG formed fewer H-bonds (most of time with 1 H-bond) with active sites (Figure 7e) than exosite 1 (Figure 7f), but considering that the RMSD of SG fluctuated sharply (Figure 7d) during the whole 10ns of dynamics simulation, we may speculate that H-bonds contribute little to its binding. The binding modes of KF and SG interacting with Ctsk at the end of the 10 ns MD simulation were captured (shown in Figure 8), We may visualize that both conformations of KF (Figure 8a) and SG (Figure 8b) in active site of Ctsk changed slightly when compared with the initial docking pose showed in Figure 6. Both of them can form two H-bonds with residues in the active site of Ctsk after reaching dynamic equilibrium. The conformation of DHT in exosite1 (Figure 8c) was closed to the docking pose as reported by others previously [27]. It should be noted that both KF (Figure 8d) and SG (Figure 8e) interact with exosite 1 just on the shallow surface, even though both of they can form 1 H-bond with Asp82, nearly half of their structure was exposed outside the site which shows little interaction with residues in exosite 1. All of these results indicate that KF and SG may prefer to interact with active site rather than exosite 1.

## 3. Discussion

Ctsk is a protease abundantly expressed in osteoclasts, and has become the most extensively studied molecule and a novel anti-resorption drug target in preclinical and clinical studies [28,29]. Ctsk is responsible for the degradation of collagen type I and other bone matrix proteins within the acidic environment of resorption lacunae [30]. Inhibition of Ctsk could reduce bone resorption or even increase bone formation by osteoclast-derived factors or matrix-derived growth factors [31]. Several Ctsk inhibitors including Odanacatib [32], ONO5334 [33], balicatib [34], and relacatib [35], have been selected to enter clinical development. However, ONO5334, balicatib, and relacatib unfortunately failed in phase I or II of trials due to their side effects and other commercial reasons [36]. The most promising agent, Odanactib, was declared to discontinued development in phase III trials due to its increased risk of stroke. Thus, only one cathepsin K inhibitor MIV-711 (Medivir) are currently advanced into phase II trials for the treatment of osteoarthritis [37]. Since most of the synthesized Ctsk inhibitors suffered setbacks in the clinical trials, new discovery is needed for the development of Ctsk inhibitors for the treatment of osteoporosis. KF and SG, the naturally-derived compounds from RD were predicted and demonstrated as Ctsk inhibitors in the present study, possessing the potential to safely treat osteoporosis. KF and SG as a pair of isomers, are polyphenols, which are characterized as belonging to the “lavandulyl group” in their chemical structure. They were highly abundant in *Sophora flavecens* [38], and were first isolated from *Drynaria genus* [12] in our previous study. Our results indicated that a neighbor compound identified by similarity searching, known as AT-5-1 (Figure 1c), can act on target Ctsk (IC50: 170 nM) and is a natural product isolated from the buds of *Artocar pus altilis* (Moraceae) in Taiwan [39]. Both of the query compounds, KF and SG, obtained a similarity rate above 80% when compared with AT-5-1 via target prediction tool PredictFX. Indeed, this finding is in agreement with previous results that reported the inhibitory effects of RD crude extracts on bone resorption activity through inhibiting activity of Ctsk in cultured mouse osteoclast [16]. The in silico target fishing method therefore provides two lead compounds in RD that could potentially inhibit Ctsk and the process of bone resorption. The results from the PredictFX 1.1 were then validated by using fluorescence-based Ctsk enzyme assay as well as activities in osteoclastic cell line. Therefore, apart from the classical estrogen dependent actions, our study further provided proof for the inhibitory actions of RD on Ctsk in bone cells. This information will be important for understanding the molecular actions of RD and provide scientific rationale for its use for the treatment of bone-related disease such as osteoporosis.

However, the present study did not provide information regarding its selectivity of these two compounds toward different types of Cathepsins. Recent studies indicated that many Ctsk inhibitors exhibit selectivity towards cathepsin family member, especially Cathepsin B, V, L, and S [40]. The approach is to identify a specific inhibitor that could block bone collagen degradation without affecting other proteolysis activities so as to avoid potential adverse events, such as skin rashes and morphaea-like skin, before clinical trial. However, it should be noted that the selectivity profile of an inhibitor might reduce over time in cell-based enzyme occupancy assay [41] despite the fact that some inhibitors may exhibit high selectivity toward Ctsk in enzyme assay. For example, the selectivity profile of balicatib, a highly selective inhibitor towards non-K cathepsins, was compromised over time as they were found to accumulate in the acidic lysosomes [31]. Thus, future work will be needed to determine if the two compounds will exert selectivity towards specific cathepsin and evaluate its lysosomotropic properties in cell.

Ctsk as an osteoporosis therapeutic target has raised many concerns in recent years. Most of the Ctsk inhibitors were designed based on its active-site, which block the proteolysis activity (remodeling of the extracellular matrix (ECM)) of Ctsk, However, recent reports [27,42] showed that there exist other two exosites that are distant from its active site: the exosite 1 (Tyr87-Gly102) which contributes to elastin and collagen degradation, and exosite 2 (Gly109-Glu118) which specifically contributes to elastin degradation. As Ctsk expressed in osteoclasts is mainly responsible for the degradation of bone collagen, which accounts for 90% of the organic bone matrix, exosite 1 has attracted more attention as a target site for the development of collagenolytic specific inhibitor. This new class of inhibitor might potentially circumvent some adverse effects that are caused by blocking the activities of Ctsk on non-collagen substrate. In our study, we also investigated the possible binding site of these two compounds via utilizing molecular dynamics and docking method, thus allowing us to determine if KF and SG are active site-directed inhibitors or exosite 1-targeting inhibitors. Our results indicated that KF and SG are more likely to bind stably with the active site rather than the exosite 1 of Ctsk. Further study will be required to validate our prediction through collagenase activity assay and X-ray crystallographic studies.

Our results clearly indicated that KF and SG strongly suppressed osteoclastogenesis and that their abilities to inhibit Ctsk-related bone resorption were demonstrated. However, targets other than Ctsk might also be involved in mediating the actions of these compounds on the process of osteoclast differentiation. Previous study showed that estrogen can block osteoclast differentiation in both primary monocytes as well as in RAW264.7 cells via ER (Estrogen receptor) [43]. Other reports further indicated that ERα, but not ERβ, was involved in the regulation of OPG/RANKL ratio and serum levels of several cytokines such as IL-6, TRAP 5b, and FasL in mice [44,45]. Moreover, many reported compounds that possess similar structure with these two components have demonstrated potent binding affinity to ER [46,47,48]. Thus, we speculated that KF and SG as kinds of phytoestrogen may also inhibit osteoclastogenesis through interacting with ER. Our speculation was confirmed by the results of target prediction (Table S2 and Table S3), which indicated that SG and KF might possess binding affinity to ER. In addition, our previous study also discovered that KF and SG are effective in improving osteoblastic differentiation in rat osteoblast-like UMR106 cells. The stimulatory effect of KF in UMR 106 cells could be abolished in the presence of estrogen receptor (ER)-antagonist (ICI 182, 780) [13]. Thus, the results of our previous and present studies both indicate that, except for inhibting Ctsk, KF and SG might also exhibit an osteoprotective effect through simultaneously regulating osteobastogenensis and osteoclastogenesis via ER. Such actions of KF and SG fit into the concept of polypharmacology, which describes how one or more compounds could target several biological targets simultaneously for efficacious disease modulation [18]. Considering that some of the anti-resorptive agents such as bisphosphonates and denosumab were negatively reported to suppress bone formation while inhibiting bone resorption due to the coupling effect of the bone formation and bone resorption process [2]. Thus, KF and SG appear to exhibit a distinct mechanism of action in which their suppression of bone resorption were accompanied by the stimulation of bone formation. Further study will be needed to comprehensively evaluate if these two compounds can inhibit bone resorption while promoting bone formation in vivo.

TCM is a rich medicinal resource that is commonly used to treat various diseases in China but knowledge of their mechanism of actions is limited. With the rapid development of separation and extraction technology, millions of ingredients have been identified and many of them are proven to be active in various biological evaluations. Yet, how to quickly identify targets that are responsible for the actions of TCM is still a challenging task. Here, we proposed an effective strategy to identify the biological targets of ingredients in TCM that account for its anti-osteoporosis actions, through combining a novel ligand-based similarity searching method with target protein-guided biological validation.

## 4. Materials and Methods

### 4.1. The Establishment of RD Ingredients Database

In order to perform in silico target prediction, a ligand database, which is composed of compounds isolated and identified from RD, was first established. The detailed information of compounds was summarized in Table S1. Some of these compounds had been shown to exert osteoprotective effects on osteoblast-like UMR106 cells in our previous study [11,12] and are defined as orphan compounds that modulate cellular phenotypes with unknown molecular targets [22].

### 4.2. In Silico Target Fishing

In order to link the query compounds in RD ingredients database to their biological targets, a commercial in silico target prediction software PredictFX 1.1 (Tripos International, St. Louis, MO, USA) based on descriptor-based similarities, fuzzy fragment-based mapping, and target cross-pharmacology [26] was applied in the present study. PredictFX 1.1 integrated several public biologically annotated chemical databases such as PubChem, BindingDB, and DrugBank. In PredictFX 1.1, an ensemble approach SFP (SHED-FPD-PHRAG) was used to capture and characterize the structural and pharmacophoric features of compounds. This approach contained three types of 2-D (dimensional) descriptors: Pharmacophoric fragments (PHRAG), Feature-pair distributions (FPD) and Shannon entropy descriptors (SHED) [26,49]. PHRAG and FPD were used to calculate the similarity between two molecules that account for the overlapping fraction of their active profiles, and SHED was responsible for computing the Euclidean distance [50]. PredictFX 1.1 has been successfully adopted by others [51,52,53] in identification of novel targets of small molecules.

Here, structure of 48 query compounds were first converted to SMILES (Simplified Molecular Input Line System) string [54], a chemical notation language that can be recognized by PredictFX 1.1. The software using SFP ensemble approach to compare the similarity/distance between these compounds and all 426,202 molecules annotated with affinity data then analyzed the information. As a result, those reference compounds from the library with similarity values above 0.76 (PHRAG) and 0.87 (FPD), and distance values below 0.52 (SHED) were regarded as neighbors of the orphan compounds. These compounds potentially share the same protein targets, and the affinity value of the query compounds for hitting targets such as p*K*i (−log*K*i; *K*i: inhibition constant) and p*K*d (−log*K*d; *K*d: dissociation constant) were estimated by inverse distance weighting (IDW) interpolation of the experimental affinities from all neighboring molecules found within a predetermined applicability domain [50]. Equation of IDW was showed as following:

(1) $$F=\sum_{i=1}^{n}W_{i}f_{i};\;W_{i}=\frac{d_{i}^{-p}}{\sum_{j=1}^{n}d_{j}^{-p}}$$

*“where F is the value of the function (pK_i_, etc.), n represents for the number of scatter plots in the neighborhood of the interpolated point, f_i_ stands for the value of the function in point i, and W_i_ can be counted as assigned weight function. In the calculation of W_i_, d can be regarded as the distance between the interpolated and the scatter points while p is an arbitrary positive real number, also known as the power parameter which generally assigned to 2. In the case of PHRAG and FPD, distances are obtained by subtracting similarities from unity”* [50]. The targets list of RD ingredients attached with detailed affinity data were generated upon computation, the post-analysis for the target profile was further implemented by literature mining and database retrieving.

### 4.3. Experimental Validation

#### 4.3.1. Chemicals and Reagents

The compounds of Kushennol F (KF) and Sohoravanone G (SG) were isolated and identified as previously described [21]. Cell culture medium including Alpha-modified Minimum Essential Medium Eagle (α-MEM), Phenol Red Free α-MEM, Dulbecco’s Modified Eagle’s Medium (DMEM), and cell culture serum including Fetal Bovine Serum (FBS) and Dextran-charcoal-stripped Fetal Bovine Serum (sFBS) were purchased from Gibco (Gibco, Gaithersburg, MD, USA). Penicillin and streptomycin were purchased from Invitrogen (Invitrogen, Carlsbad, CA, USA). 17β-estradiol (E_2_), receptor activator of nuclear factor κB (NF-κB) ligand (sRANKL), and Macrophage Colony-Stimulating Factor (M-CSF) were purchased from Sigma Aldrich (Sigma, St. Louis, MO, USA).

#### 4.3.2. Cathepsin K Inhibition Activity Assay

A Cathepsin K Inhibitor Screening kit (Biovision, Milpitas, CA, USA) was used to validate the inhibitory effects of compounds on Cathepsin K (Ctsk) activities. Ctsk can cleave its preferred synthetic substrate sequence LR (leucine-arginine) labeled with AFC (amino-4-trifluoromethyl coumarin) to release free AFC, the released AFC can be quantified by the intensity of fluorescence. Briefly, Ctsk enzyme protein (Human Recombinant, BioVision, Milpitas, CA, USA) was reconstituted with reaction buffer, and mixed with reaction buffer and Ctsk reagent in a 96-well black microtiter plate. Different concentrations of the Ctsk inhibitor (FF-FMK, positive control, 10^−10^ to 10^−5^ M), KF (10^−9^ to 10^−3^ M) and SG (10^−9^ to 10^−4^ M) were added into Ctsk containing wells and incubated at room temperature for 15 min. Then, substrate solution containing Ctsk substrate LR-AFC and Ctsk Reaction buffer was added into each well and incubated for another 30–60 min at 37 °C in a kinetic mode. The substrate hydrolysis was monitored with FLUOstar Galaxy (BMG LABTECH, Cary, NC, USA) by measurement of fluorescence intensity at excitation wavelength of 400 nm and emission wavelength of 505 nm. Time point *T*_1_: 25 min and *T*_2_: 30 min in the linear range of the plot were selected to obtain the corresponding fluorescence value, the slope for all samples was calculate by dividing the relative fluorescence values with the time Δ*T* (*T*_2_ − *T*_1_), and the data was calculated as below:

Relative Inhibition (%) = [Slope (EC) − Slope (S)] × 100/Slope (EC)(2)

The IC50 values were calculated by using GraphPad Prism 5.0 software (GraphPad Software, Inc, La Jolla, CA, USA).

#### 4.3.3. RAW264.7 Cell Culture

Murine macrophage RAW264.7 cells were obtained from American Type Culture Collection (ATCC) and routinely cultured in DMEM supplemented with 10% FBS, and 100 U/mL of penicillin and 100 μg/mL of streptomycin at 37 °C in a humidified atmosphere of 95% and 5% of CO_2_. Cells were sub-cultured every two to three days and the medium was changed every two days. To assess the effects of compounds on cell cytotoxicity, RAW264.7 cells were seeded at 96-well plate (10^4^ cells/well). After 24 h, the cells were treated with different concentrations of KF (5–50 μM), SG (5–50 μM), 17β-estradiol (E_2_, 10^−8^ M) or vehicle for a further four days followed by cell cytotoxicity assay. To assess the effects of estrogen or the compounds of KF and SG on cell differentiation, cells were seeded in a 96-well or 12-well plate, at a density of 3000 cells/well or 10^5^ cells/well. On the second day, media were changed into phenol-red free α-MEM supplemented with 10% sFBS for another 24 h. Then the cells were treated with different concentrations of KF (5, 12.5 μM), SG (5, 12.5 μM), 17β-estradiol (E_2_, 10^−8^ M), or vehicle (1% ethanol) in differentiation medium containing M-CSF (30 ng/mL), sRANKL (100 ng/mL) for an additional four days. After six days’ culture, cells were collected for cytochemical staining or TRAP activity measurement.

#### 4.3.4. Cell Cytotoxicity Assay

MTS(3-(4,5-dimethylthiazol-2-yl)-5-(3-carboxy-methoxy-phenyl)-2-(4-sulfophenyl)-2*H*-tetrazolium) assay (Promega, Madison, WI, USA) was used as an indirect colorimetric measurement of cell cytotoxicity. Upon treatment of cells as described above, the medium was discarded and replaced with 100 μL of MTS/PMS solution and incubated at 37 °C for 1 h, the absorbance at 490 nm was measured on a spectrophotometric plate reader (Bio-Rad model 550, Richmond, CA, USA).

#### 4.3.5. TRAP Positive Multinucleated Cells Staining

Treated cells were fixed and stained for TRAP, an enzyme generally accepted as a marker for osteoclasts, using a commercially available kit (Sigma, St. Louis, MO, USA). TRAP-positive multinucleated cells showing more than three nuclei were considered to be osteoclasts and were counted as such. Images of the TRAP-positive cells were captured under light microscope by using camera (Olympus, Tokyo, Japan).

#### 4.3.6. TRAP Activity Measurement

Upon treatment for six days, RAW264.7-derived osteoclasts were measured by the Acid Phosphatase Assay Kit (Cat. K411-500, BioVision, Milpitas, CA, USA). Briefly, the cells were lysed and incubated for 1 h with reaction buffer containing paranitrophenylphosphate (pNPP). The reaction was stopped with stop solution. Optical densities (ODs) were read using microplate spectrophotometer at 405 nm. ODs were compared to a standard curve calibrate with paranitrophenol (pNP). Protein contents were quantified with Biorad Protein Assay. Results were expressed as micro-moles of pNP/mg protein.

#### 4.3.7. Pit Formation Assay

Bone resorption activity was assessed by using commercially available bone resorption assay kit 24 (CosMo Bio, Tokyo, Japan). Firstly, the calcium phosphate (Ca-P)-coated plates were incubated with bone resorption assay fluoresceinamine-labeled chondroitin sulfate (FACS) at 37 °C for two hours under light-shielded conditions. RAW264.7 cells were then seeded into such plate at density of 10^4^ cells/well, supplemented with phenol red-free α-MEM containing 10% sFBS. Different concentrations of KF (5, 12.5 μM), SG (5, 12.5 μM), vehicle (1% ethanol) or 17β-estradiol (E_2_, 10^−7^ M) were added together with sRANKL (100 ng/mL) and M-CSF (30 ng/mL) and incubated for another three days. On Day 6, conditioned medium was transferred from each well into 96-well plate (OptiPlate-96F black, PerkinElmer, Inc., Waltham, MA, USA), then mixed well with bone resorption assay buffer, fluorescence intensity generated at excitation wavelength of 485 nm and emission wavelength of 535 nm was measured using CLARIOstar microplate reader (BMG LABTECH, Cary, NC, USA). To observe pit formation, cells in each well were removed by treatment with 5% sodium hypochlorite for 5 min, followed by washing and air drying. Bone resorption pits were observed under microscope and images were captured by Olympus DP2-BSW. Areas of resorption pits were quantified using Image-Pro plus 6.0 software (Media Cybernetics, Silver Spring, MD, USA ).

### 4.4. Docking Analysis

Molecular docking was performed using Surflex-Dock in Sybyl 8.0 (Tripos International, St. Louis, MO, USA) on a Linux operating system. The structure of KF and SG were sketched using SYBYL 8.0 sketch program (Tripos International, St. Louis, MO, USA), hydrogen atoms were added, and their energies were minimized using the the Tripos force field and Powell method with the termination gradient set at 0.05 kcal/mol, and maximum iterations at 2000. Gasteiger-Hückel charges were assigned. The human Cathepsin K crystal structure complexed with a non-selective 2-cyano-pyrimidine inhibitor (PDB: 3KWZ) at 1.49 Å resolution was retrieved from PDB (Protein Data Bank; http://www.pdb.org), GeomX docking mode was chosen, water molecules, and other ligands (SO_4_) were deleted, addition of hydrogen atoms, and the ligand binding site protomol was generated automatically using original ligand KWZ extracted from PDB: 3KWZ as a reference. Other parameters were set by default if not specially illustrated. KF, and SG were then docked into the active site and exosite 1 (a hook-like loop spanning from Tyr87 to Gly102) of Ctsk with Surflex-Dock (Tripos Inc., St. Louis, MO, USA), respectively. The predicted binding modes of KF and SG with an active site of Ctsk were preliminarily visualized by PyMol Molecular Graphics System (The PyMOL Molecular Graphics System, version 1.6.x; Schrödinger, LLC, Portland, OR, USA). Amino acid residues appearing at a 5 Å distance around docked ligand were labeled.

### 4.5. Molecular Dynamics

To determine the stability and mechanistic aspects of KF and SG in complex with Ctsk, MD simulations were carried out using the GROMACS package (version 5.1.2) [55]. Initial protein-ligand complex was derived from the molecular docking, then proteins and query compounds were charged using the AMBER99SB-ILDN force field and the General AMBER force field (GAFF), respectively. The protein-ligand complex was solvated in a cubic box filled with TIP3P water molecules, the minimum distance from the closest atom of solutes to the edge of the box was found to be 0.1 nm. Periodic boundary conditions (PBC) were employed to avoid edge effects in MD simulations. To make the system neutral, we added Na^+^ and Cl^−^ to simulate a physiological NaCl concentration of 0.15 M. A steepest descent energy minimization was carried out to remove poor contacts. Then, the system was equilibrated using 100 ps under constant volume (NVT) and 100 ps constant pressure (NPT) as conditions to ensemble with simultaneous protein-ligand position restraints. Consequently, 10 ns MD production simulation was performed with a time step 2 fs at constant temperature (300 K) and pressure (1 atm). RMSD and number of hydrogen bond analyses were carried out by the g_rmsd and g_hbond tools in the GROMACS package (version 5.1.2) [55], respectively. The last frames (time of 1000 ps) of the equalized trajectory were extracted to determine the optimal docked conformation by using PyMOL (The PyMOL Molecular Graphics System, version 1.6.x; Schrödinger, LLC, Portland, OR, USA).

### 4.6. Statistical Analysis

Results are presented as mean ± SEM (standard error of the mean). All statistical analyses were performed using the one-way analysis of variance (ANOVA) for inter-group differences and followed by post hoc Tukey test for multiple comparisons in PRISM version 5.0.1 (GraphPad, San Diego, CA, USA). *p*-values < 0.05 was considered as significant.

## 5. Conclusions

The present study is our first attempt to utilize an in silico target fishing method to identify potential targets involved in mediating the anti-osteoporotic effects of *Rhizoma Drynariae* (RD), and subsequently we have successfully identified KF and SG to be the active ingredients in RD that specifically act on lysosomal enzyme Ctsk. KF and SG were shown to suppress Ctsk activities with IC50 of 8.80 and 27.24 μM, respectively. The biological actions of KF and SG on pit formation by osteoclasts were further confirmed using cultured RANKL-induced RAW264.7 cells. Our results were further confirmed by the study of the interactions between these two compounds and protease Ctsk using molecular docking and dynamics method. This study clearly demonstrates that in silico target fishing method can be used to quickly identify possible molecular targets and reveal the mechanism of action involved in mediating the anti-osteoporotic actions of TCM. Most importantly, these two natural plant-derived compounds are shown to be promising novel Ctsk inhibitors that might be useful for the management of osteoporosis.

## Figures and Tables

**Table 1:** The information of Kushennol F (KF) and Sophoraflavone G (SG) linked to Ctsk retrieved from PredictFX.

Parameters	Compound Identifier	
	Kushennol F	Sophoraflavanone G
ANN	PRD	--
pIC50	6.8	--
REF_NN	FVNFXIPJDHVJGE-REZTVBANSA-N	--
SIM	0.841	0.838
REF_pACT	6.77	--
SOURCE_DB	ChemblDB, PubChem, Binding DB	--
UNIPROT	P43235	--
TARGET NAME	Cathepsin K	--
FUNCTIONAL	EC, FD, PS	--

--: same as left; ANN: Annotation type; PRD (prediction); pIC50: predicted value for −log10 of IC50; REF_NN: Reference compound (neighbouring compound) identifier; SIM: Similarity (or identity) to the reference compound; REF_pACT: Reference compound experimental pActivity value; SOURCE_DB: Original Database(s) reporting this REF_NN-protein annotation. UNIPROT: protein UNIPROT code; FUNICTIONAL: protein Function family, the details of brief name listed in Table S4. TARGET_NAME: Protein Full Name. DB: Database; EC: Enzymes; FD: Chaperones and folding catalysts; PS: Peptidases.

## Supplementary Materials

Supplementary materials can be found at www.mdpi.com/1422-0067/17/12/2116/s1.

## Abbreviations

Ctsk	Cathepsin K
RD	*Rhizoma Drynariae*
KF	Kushennol F
SG	Sophoraflavanone G
TCM	Traditional Chinese Medicine
MNCs	Multi-nucleated cells
TRAP	Tartrate-resistant acid phosphatase
MD	Molecular Dynamics
