# Supplementary material for: Discovery of a New Class of Cathepsin K Inhibitors in Rhizoma Drynariae as Potential Candidates for the Treatment of Osteoporosis
Source: Int J Mol Sci. 2016 Dec 16;17(12):2116. doi: 10.3390/ijms17122116 (PMC5187916; doi:10.3390/ijms17122116)
Supplement: Supplementary file 1 [file ijms-17-02116-s001.pdf]

Supplementary Materials: Discovery of a New Class of Cathepsin KInhibitors in *Rhizoma Drynariae*as Potential Candidates for the Treatment of Osteoporosis

Zuo-Cheng Qiu, Xiao-Li Dong, Yi Dai, Gao-Keng Xiao, Xin-Luan Wang, Ka-Chun Wong, Man-Sau Wong and Xin-Sheng Yao

Table S1. Compounds identified from *Drynariae rhizome* (DR).

No.	Compound Name	Chemical Structure
**1**	Naringin	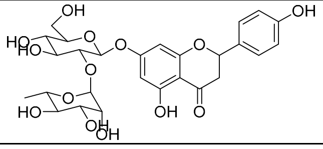
**2**	5,7,3′,5′-Tetrahydroxy-flavanone 7-*O*-neohesperidoside	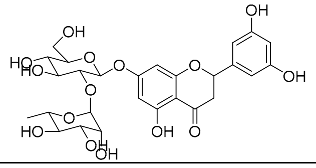
**3**	Narigenin-7-*O*-β-D-glucoside	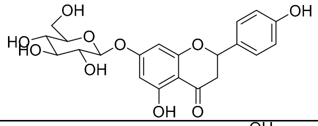
**4**	5,7,3′,5′-Tetrahydroxy-flavanone 7-*O*-β-D-glucopyranoside	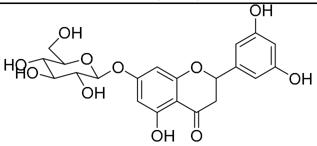
**5**	Naringenin	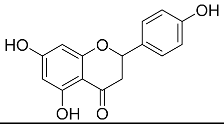
**6**	5,7,3′,5′-Tetrahydroxyflavanone	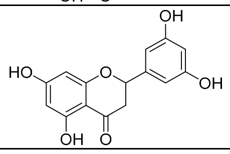
**7**	Kushennol F	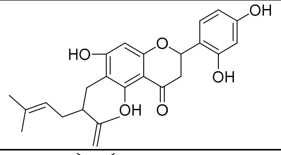
**8**	Sophoraflavanone G	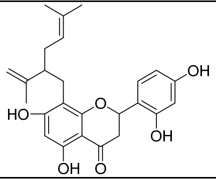
**9**	Kurarinone	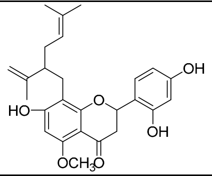

Table S1. *Cont.*

No.	Compound Name	Chemical Structure
**10**	Leachianone A	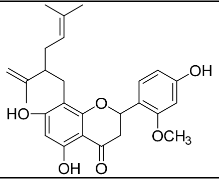
**11**	Luteolin-7-*O*-neohesperidoside	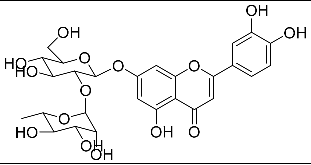
**12**	Luteolin-5-*O*-neohesperidoside	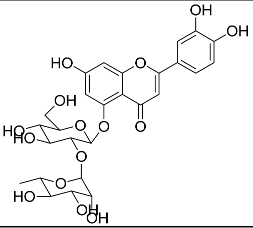
**13**	Kaempferol-7-*O*-α-L-arabinofuranoside	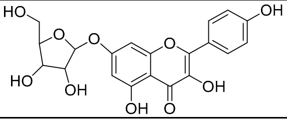
**14**	8-Prenylapigenin	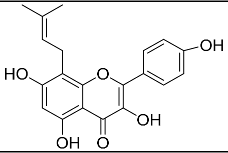
**15**	Apigenine	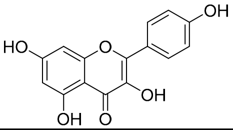
**16**	Kaempferol-3-*O*-α-L-rhamnopyranoside	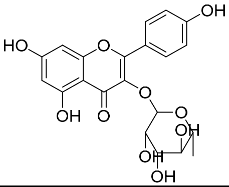
**17**	Astragalin	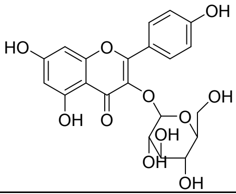
**18**	3-*O*-β-D-Glucopyranoside-7-*O*-α-L-arabinofuranoside	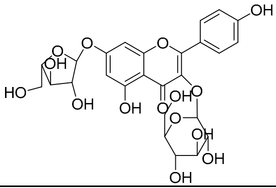
**19**	5,7-Dihydroxychromone-7-*O*-β-D-glucopyranoside	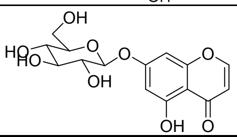

Table S1. *Cont.*

No.	Compound Name	Chemical Structure
**20**	5,7-Dihydroxychromone-7-*O*-neohesperidoside	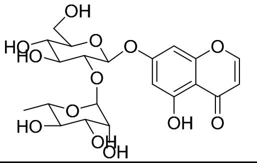
**21**	Kaempferol 3-*O*-β-D-glucopyranoside-7-*O*-β-D-glucopyranoside	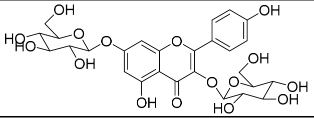
**22**	Xanthohumol	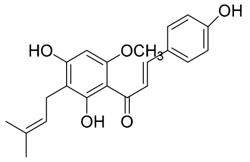
**23**	Epicatechin	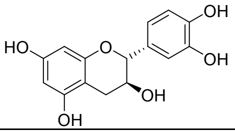
**24**	(*E*)-4-*O*-β-D-Glucopyranosyl caffeic acid	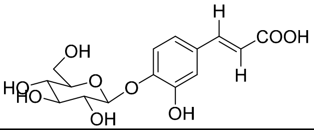
**25**	β-D-Glucopyranosyl sinapoic acid	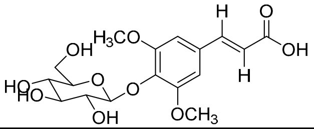
**26**	4-*O*-β-D-Glucopyranosyl ferulic acid	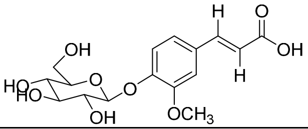
**27**	Trans-caffeic acid	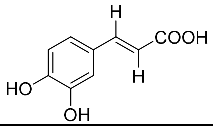
**28**	4-*O*-β-D-Glucopyranosyl coumaric acid	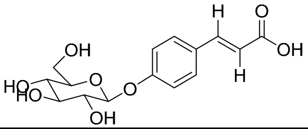
**29**	Dihydrocaffeic acid methyl ester	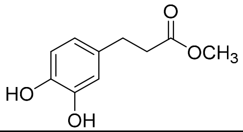
**30**	Dihydrocaffeic acid	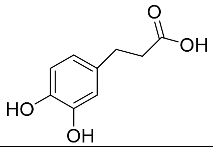
**31**	3,4-Dihydroxyl benzoic acid	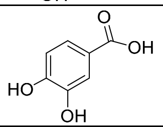
**32**	4-*O*-D-Glucosyl vanillic acid	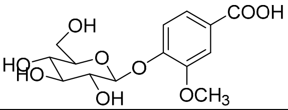

Table S1. *Cont.*

No.	Compound Name	Chemical Structure
**33**	5-Ethoxy-2-hydroxy-benzoate	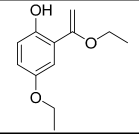
**34**	3-(Acetylamino)-4-hydroxy-benzoic acid	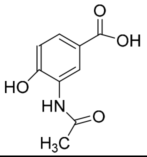
**35**	12-*O*-Caffeoyl-12-hydroxyldodecanoic acid	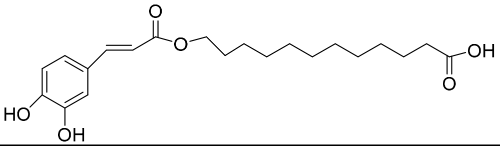
**36**	β-Sitosterol	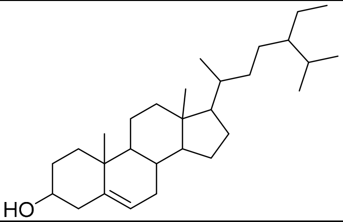
**37**	Maltol glucoside	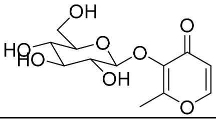
**38**	1,2-Benzenedicarboxylic acid diisobutyl ester	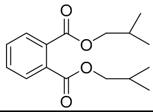
**39**	1,2,3,4,6-Penta-*O*-galloyl-β-D-glucose	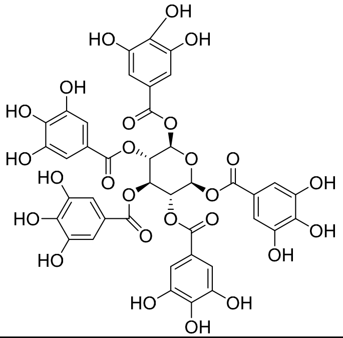
**40**	1,2,3,4,6-Tetra-*O*-galloyl-β-D-glucose	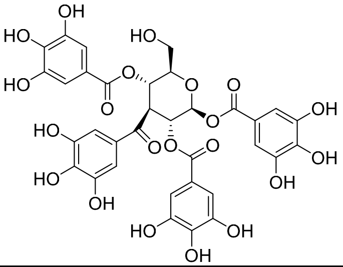

Table S1. *Cont.*

No.	Compound Name	Chemical Structure
**41**	2,3,4,6-Tetra-*O*-galloyl-α-D-glucose	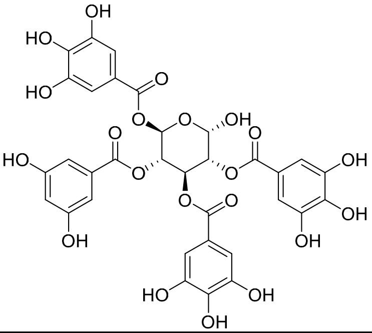
**42**	Palmarumycin JC1	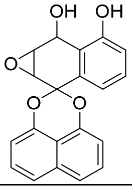
**43**	Kaempferol 3-*O*-α-L-rhamnoside-7-*O*-β-D-glucopyranoside	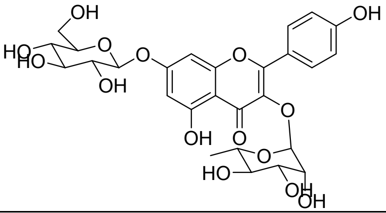
**44**	5-Ethoxy-3-hydroxy-benzoate	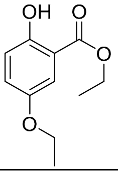
**45**	Protocatechuic acid	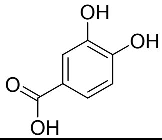
**46**	Prunin	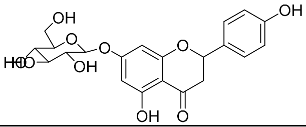
**47**	Kaempferol-3-*O*-α-L-rhamnoside-7-*O*-D-glucoside	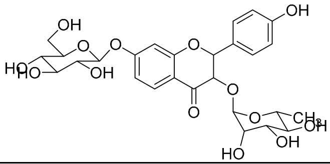
**48**	Aureusidin-6-*O*-neohesperidoside	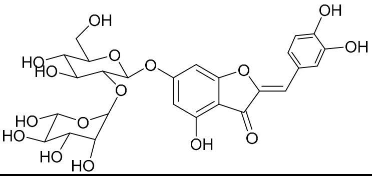

Table S2. Information for evey identified molecule-protein interaction from PredictFX. Anotation: MOLID: Compound identifier; AN: Annotation type; EXP: experimental; PRD: prediction; pActivity: −log10 of measured affinity or "active" or "inactive" tags if quantitative measure is not available for a specific interaction; A: active; pX: Experimental/Predicted value for affinity type X (IC50, EC50, *K*i, *K*d or *K*d); ---: not available; UNIPOT: protein UNIPROT code; FUNTIONAL: Protein Functional Family (The functional families classification in Table S4); TARGET_NAME: Protein Full Name.

MOLID	AN	pACT	p*Ki*	p*Kd*	p*Kb*	pIC50	pEC50	Uniprot	Functional	TARGET_NAME
Naringin	PRD	A	---	---	---	---	---	P42330	EC	Aldo-keto reductase family 1 member C3
Naringin	PRD	8.1	8.1	---	---	---	---	P08913	GR	α-2A adrenergic receptor
Naringin	PRD	7.3	7.3	---	---	---	---	P18825	GR	α-2C adrenergic receptor
Naringin	PRD	5	---	---	---	5	---	Q02410	UC	Amyloidβ A4 precursor protein-binding family A member 1
Naringin	EXP	5.3	---	---	---	5.3	---	P11511	CP, EC	Cytochrome P450 19A1
Naringin	PRD	A	---	---	---	---	---	P10632	CP, EC	Cytochrome P450 2C8
Naringin	PRD	A	---	---	---	---	---	P11712	CP, EC	Cytochrome P450 2C9
Naringin	PRD	A	---	---	---	---	---	P10635	CP, EC	Cytochrome P450 2D6
Naringin	PRD	7.5	7.5	---	---	---	---	P08912	GR	Muscarinic acetylcholine receptor M5
Naringin	EXP	5.1	---	---	---	5.1	---	P61088	BQ, DR, EC	Ubiquitin-conjugating enzyme E2 N
5,7,3′,5′-Tetrahydroxy-flavanone 7-*O*-neohesperidoside	PRD	A	---	---	---	---	---	P42330	EC	Aldo-keto reductase family 1 member C3
5,7,3′,5′-Tetrahydroxy-flavanone 7-*O*-neohesperidoside	PRD	8.1	8.1	---	---	---	---	P08913	GR	α-2A adrenergic receptor
5,7,3′,5′-Tetrahydroxy-flavanone 7-*O*-neohesperidoside	PRD	7.3	7.3	---	---	---	---	P18825	GR	α-2C adrenergic receptor
5,7,3′,5′-Tetrahydroxy-flavanone 7-*O*-neohesperidoside	PRD	5	---	---	---	5	---	Q02410	UC	Amyloid β A4 precursor protein-binding family A member 1
5,7,3′,5′-Tetrahydroxy-flavanone 7-*O*-neohesperidoside	PRD	5.3	---	---	---	5.3	---	P11511	CP, EC	Cytochrome P450 19A1
5,7,3′,5′-Tetrahydroxy-flavanone 7-*O*-neohesperidoside	PRD	A	---	---	---	---	---	P10632	CP, EC	Cytochrome P450 2C8
5,7,3′,5′-Tetrahydroxy-flavanone 7-*O*-neohesperidoside	PRD	A	---	---	---	---	---	P11712	CP, EC	Cytochrome P450 2C9
5,7,3′,5′-Tetrahydroxy-flavanone 7-*O*-neohesperidoside	PRD	A	---	---	---	---	---	P10635	CP, EC	Cytochrome P450 2D6
5,7,3′,5′-Tetrahydroxy-flavanone 7-*O*-neohesperidoside	PRD	7.4	7.4	---	---	---	---	P08912	GR	Muscarinic acetylcholine receptor M5
5,7,3′,5′-Tetrahydroxy-flavanone 7-*O*-neohesperidoside	PRD	5.1	---	---	---	5.1	---	P61088	BQ, DR, EC	Ubiquitin-conjugating enzyme E2 N

Table S2. *Cont.*

MOLID	AN	pACT	p*K*i	p*K*d	p*K*b	pIC50	pEC50	Uniprot	Functional	TARGET_NAME
Narigenin-7-*O*-β-D-glucoside	PRD	A	---	---	---	---	---	P05091	EC	Aldehyde dehydrogenase, mitochondrial
Narigenin-7-*O*-β-D-glucoside	PRD	5.1	---	---	---	5.1	---	P15121	EC	Aldose reductase
Narigenin-7-*O*-β-D-glucoside	PRD	5.6	---	---	---	5.6	---	P10696	EC	Alkaline phosphatase, placental-like
Narigenin-7-*O*-β-D-glucoside	PRD	A	---	---	---	---	---	Q9UNQ0	AN, TC	ATP-binding cassette sub-family G member 2
Narigenin-7-*O*-β-D-glucoside	PRD	5.3	4.9	---	---	5.6	---	P14416	GR	D_2 dopamine receptor
Narigenin-7-*O*-β-D-glucoside	PRD	5.1	5.9	---	---	4.3	---	P21917	GR	D_2 dopamine receptor
Narigenin-7-*O*-β-D-glucoside	PRD	5.2	5.2	---	---	4.7	---	P03372	NR, TR	Estrogen receptor
Narigenin-7-*O*-β-D-glucoside	PRD	5.8	---	---	---	5.8	---	P05113	CY	Interleukin-5
Narigenin-7-*O*-β-D-glucoside	PRD	5.9	---	---	---	5.9	---	P09923	EC	Intestinal-type alkaline phosphatase
Narigenin-7-O-β-D-glucoside	PRD	5.6	5.6	---	---	6.6	6.5	P13866	TC	Sodium/glucose cotransporter 1
Narigenin-7-O-β-D-glucoside	PRD	7.1	7.2	---	---	7.1	7.7	P31639	UC	Sodium/glucose cotransporter 2
5,7,3′,5′-Tetrahydroxy-flavanone 7-*O*-β-D-glucopyranoside	PRD	5.2	---	---	---	5.2	---	P15121	EC	Aldose reductase
5,7,3′,5′-Tetrahydroxy-flavanone 7-*O*-β-D-glucopyranoside	PRD	5.6	---	---	---	5.6	---	P10696	EC	Alkaline phosphatase, placental-like
5,7,3′,5′-Tetrahydroxy-flavanone 7-*O*-β-D-glucopyranoside	PRD	5.3	4.9	---	---	5.6	---	P14416	GR	D_2 dopamine receptor
5,7,3′,5′-Tetrahydroxy-flavanone 7-*O*-β-D-glucopyranoside	PRD	5.1	5.9	---	---	4.3	---	P21917	GR	D_2 dopamine receptor
5,7,3′,5′-Tetrahydroxy-flavanone 7-*O*-β-D-glucopyranoside	PRD	5.2	5.2	---	---	4.7	---	P03372	NR, TR	Estrogen receptor
5,7,3′,5′-Tetrahydroxy-flavanone 7-*O*-β-D-glucopyranoside	PRD	5.8	---	---	---	5.8	---	P05113	CY	Interleukin-5
5,7,3′,5′-Tetrahydroxy-flavanone 7-*O*-β-D-glucopyranoside	PRD	5.9	---	---	---	5.9	---	P09923	EC	Intestinal-type alkaline phosphatase
5,7,3′,5′-Tetrahydroxy-flavanone 7-*O*-β-D-glucopyranoside	PRD	5.1	---	---	---	5.1	---	P03070	EC	Large T antigen
5,7,3′,5′-Tetrahydroxy-flavanone 7-*O*-β-D-glucopyranoside	PRD	5.7	5.5	---	---	6.6	6.5	P13866	TC	Sodium/glucose cotransporter 1
5,7,3′,5′-Tetrahydroxy-flavanone 7-*O*-β-D-glucopyranoside	PRD	7.3	7.5	---	---	7.2	7.5	P31639	UC	Sodium/glucose cotransporter 2

Table S2. *Cont.*

MOLID	AN	pACT	p*Ki*	p*Kd*	p*Kb*	pIC50	pEC50	Uniprot	Functional	TARGET_NAME
Naringenin	PRD	6	6	---	---	---	---	Q5G940	UC	(3*R*)-hydroxymyristoyl-[acyl-carrier-protein] dehydratase 2
Naringenin	PRD	5.4	5.4	---	---	---	---	P30542	GR	Adenosine receptor A1
Naringenin	PRD	5.5	5.5	---	---	---	---	P33765	GR	Adenosine receptor A3
Naringenin	PRD	5.2	---	---	---	5.2	---	P15121	EC	Aldose reductase
Naringenin	PRD	5.8	---	---	---	5.8	---	P21397	EC	Amine oxidase [flavin-containing] A
Naringenin	PRD	5.3	---	---	---	5.3	---	P10275	NR, TR	Androgen receptor
Naringenin	PRD	5.4	---	---	---	5.4	---	P18054	EC	Arachidonate 12-lipoxygenase, 12*S*-type
Naringenin	EXP	A	---	---	---	---	---	Q9UNQ0	AN, TC	ATP-binding cassette sub-family G member 2
Naringenin	PRD	5.5	---	---	---	5.5	---	P08236	EC	β-glucuronidase
Naringenin	PRD	5.9	6.1	---	---	5.6	---	P68400	EC, KC	Casein kinase II subunit α
Naringenin	PRD	5.4	---	---	---	5.4	---	P67870	UC	Casein kinase II subunit β
Naringenin	PRD	5.4	---	---	---	5.4	---	P06493	DL, EC, KC	Cyclin-dependent kinase 1
Naringenin	PRD	5.8	---	---	---	5.8	---	Q15078	UC	Cyclin-dependent kinase 5 activator 1
Naringenin	PRD	5.8	---	---	---	5.8	---	Q00534	EC, KC	Cyclin-dependent kinase 6
Naringenin	EXP	6.7	---	---	---	6.7	---	P11511	CP, EC	Cytochrome P450 19A1
Naringenin	EXP	5.4	---	---	---	5.4	---	Q16678	CP, EC	Cytochrome P450 1B1
Naringenin	PRD	5.3	---	---	---	5.3	---	P10635	CP, EC	Cytochrome P450 2D6
Naringenin	EXP	5.3	---	---	---	5.3	---	P14061	EC	Estradiol 17-βdehydrogenase 1
Naringenin	PRD	5.4	---	---	---	5.4	---	P14635	DL	G2/mitotic-specific cyclin-B1
Naringenin	PRD	5.4	---	---	---	5.4	---	O95067	DL	G2/mitotic-specific cyclin-B2
Naringenin	PRD	5.4	---	---	---	5.4	---	Q8WWL7	DL	G2/mitotic-specific cyclin-B3
Naringenin	PRD	5.5	5.5	---	---	---	---	P14867	IC	γ-Aminobutyric acid receptor subunit α-1
Naringenin	PRD	5.5	5.5	---	---	---	---	P47869	IC, TC	γ-Aminobutyric acid receptor subunit α-2
Naringenin	PRD	5.5	5.5	---	---	---	---	P34903	IC	γ-Aminobutyric acid receptor subunit α-3
Naringenin	PRD	5.5	5.5	---	---	---	---	P48169	IC	γ-Aminobutyric acid receptor subunit α-4
Naringenin	PRD	5.5	5.5	---	---	---	---	P31644	IC	γ-Aminobutyric acid receptor subunit α-5
Naringenin	PRD	5.5	5.5	---	---	---	---	Q16445	IC	γ-Aminobutyric acid receptor subunit α-6
Naringenin	PRD	6.1	6.1	---	---	---	---	P18505	IC	γ-Aminobutyric acid receptor subunit β-1
Naringenin	PRD	6.1	6.1	---	---	---	---	P47870	IC, TC	γ-Aminobutyric acid receptor subunit β-2
Naringenin	PRD	6.1	6.1	---	---	---	---	P28472	IC	γ-Aminobutyric acid receptor subunit β-3
Naringenin	PRD	6.1	6.1	---	---	---	---	O14764	IC	γ-Aminobutyric acid receptor subunit δ
Naringenin	PRD	6.1	6.1	---	---	---	---	P78334	IC	γ-Aminobutyric acid receptor subunit epsilon
Naringenin	PRD	6.1	6.1	---	---	---	---	Q8N1C3	IC	γ-Aminobutyric acid receptor subunit γ-1
Naringenin	PRD	6.1	6.1	---	---	---	---	P18507	IC	γ-Aminobutyric acid receptor subunit γ-2
Naringenin	PRD	6.1	6.1	---	---	---	---	Q99928	IC	γ-Aminobutyric acid receptor subunit γ-3

Table S2. *Cont.*

MOLID	AN	pACT	p*K*i	p*K*d	p*K*b	pIC50	pEC50	Uniprot	Functional	TARGET_NAME
Naringenin	PRD	6.1	6.1	---	---	---	---	O00591	IC	γ-Aminobutyric acid receptor subunit pi
Naringenin	PRD	6.1	6.1	---	---	---	---	Q9UN88	IC	γ-Aminobutyric acid receptor subunit θ
Naringenin	PRD	5.8	---	---	---	5.8	---	P49840	EC, KC	Glycogen synthase kinase-3 α
Naringenin	PRD	5.8	---	---	---	5.8	4.3	P49841	EC, KC	Glycogen synthase kinase-3 β
Naringenin	PRD	5.4	---	---	---	5.4	---	Q07820	UC	Induced myeloid leukemia cell differentiation protein Mcl-1
Naringenin	PRD	A	---	---	---	---	---	P55157	UC	Microsomal triglyceride transfer protein large subunit
Naringenin	PRD	5.2	---	---	---	5.2	---	P90584	UC	MO15-related protein kinase Pfmrk
Naringenin	EXP	A	---	---	---	---	---	P08183	AN, EC, TC	Multidrug resistance protein 1
Naringenin	EXP	5.6	5.6	---	---	---	---	P33527	TC	Multidrug resistance-associated protein 1
Naringenin	PRD	5.2	---	---	---	5.2	---	P23219	EC	Prostaglandin G/H synthase 1
Naringenin	EXP	A	---	---	---	---	---	O94956	UC	Solute carrier organic anion transporter family member 2B1
Naringenin	PRD	A	---	---	---	---	---	P35610	EC	Sterol *O*-acyltransferase 1
Naringenin	PRD	A	---	---	---	---	---	O75908	EC	Sterol *O*-acyltransferase 2
Naringenin	PRD	5.2	---	---	---	---	5.2	P10520	EC	Streptokinase A
5,7,3′,5′-Tetrahydroxyflavanone	PRD	5.7	5.8	---	---	5.4	---	Q965D6	EC, LD	3-oxoacyl-acyl-carrier protein reductase
5,7,3′,5′-Tetrahydroxyflavanone	PRD	5.8	5.8	---	---	---	---	P30542	GR	Adenosine receptor A1
5,7,3′,5′-Tetrahydroxyflavanone	PRD	5.1	---	---	---	5.1	---	P15121	EC	Aldose reductase
5,7,3′,5′-Tetrahydroxyflavanone	PRD	5.3	---	---	---	5.3	---	P21397	EC	Amine oxidase [flavin-containing] A
5,7,3′,5′-Tetrahydroxyflavanone	PRD	5	---	---	---	5	---	P10275	NR, TR	Androgen receptor
5,7,3′,5′-Tetrahydroxyflavanone	PRD	5.4	---	---	---	5.4	---	P18054	EC	Arachidonate 12-lipoxygenase, 12*S*-type
5,7,3′,5′-Tetrahydroxyflavanone	PRD	7.4	---	---	---	7.4	---	P09917	EC	Arachidonate 5-lipoxygenase
5,7,3′,5′-Tetrahydroxyflavanone	PRD	7.6	---	---	---	7.6	---	P35869	TR	Aryl hydrocarbon receptor
5,7,3′,5′-Tetrahydroxyflavanone	PRD	A	---	---	---	---	---	Q9UNQ0	AN, TC	ATP-binding cassette sub-family G member 2
5,7,3′,5′-Tetrahydroxyflavanone	PRD	5.6	5.6	---	---	---	---	P00915	EC	Carbonic anhydrase 1
5,7,3′,5′-Tetrahydroxyflavanone	PRD	5.3	5.3	---	---	---	---	O43570	EC	Carbonic anhydrase 12
5,7,3′,5′-Tetrahydroxyflavanone	PRD	5.1	5.1	---	---	---	---	Q99N23	EC	Carbonic anhydrase 15
5,7,3′,5′-Tetrahydroxyflavanone	PRD	5.7	5.7	---	---	---	---	P00918	EC	Carbonic anhydrase 2
5,7,3′,5′-Tetrahydroxyflavanone	PRD	5.5	5.5	---	---	---	---	P07451	EC	Carbonic anhydrase 3
5,7,3′,5′-Tetrahydroxyflavanone	PRD	5.3	5.3	---	---	---	---	P22748	EC	Carbonic anhydrase 4
5,7,3′,5′-Tetrahydroxyflavanone	PRD	5.4	5.4	---	---	---	---	P35218	EC	Carbonic anhydrase 5A, mitochondrial
5,7,3′,5′-Tetrahydroxyflavanone	PRD	5.4	5.4	---	---	---	---	Q9Y2D0	EC	Carbonic anhydrase 5B, mitochondrial

Table S2. *Cont.*

MOLID	AN	pACT	p*K*i	p*K*d	p*K*b	pIC50	pEC50	Uniprot	Functional	TARGET_NAME
5,7,3′,5′-Tetrahydroxyflavanone	PRD	5.3	5.3	---	---	---	---	P23280	EC	Carbonic anhydrase 6
5,7,3′,5′-Tetrahydroxyflavanone	PRD	6.3	6.3	---	---	---	---	P43166	EC	Carbonic anhydrase 7
5,7,3′,5′-Tetrahydroxyflavanone	PRD	5.3	5.3	---	---	---	---	Q16790	EC	Carbonic anhydrase 9
5,7,3′,5′-Tetrahydroxyflavanone	PRD	5	---	---	---	5	---	P06493	DL, EC, KC	Cyclin-dependent kinase 1
5,7,3′,5′-Tetrahydroxyflavanone	PRD	5.1	---	---	---	5.1	---	Q15078	UC	Cyclin-dependent kinase 5 activator 1
5,7,3′,5′-Tetrahydroxyflavanone	PRD	5.9	---	---	---	5.9	---	P11511	CP, EC	Cytochrome P450 19A1
5,7,3′,5′-Tetrahydroxyflavanone	EXP	5.9	---	---	---	5.9	---	Q16678	CP, EC	Cytochrome P450 1B1
5,7,3′,5′-Tetrahydroxyflavanone	PRD	5.2	5.2	---	---	---	---	P11712	CP, EC	Cytochrome P450 2C9
5,7,3′,5′-Tetrahydroxyflavanone	PRD	6	---	---	---	6	---	P11387	DL, EC	DNA topoisomerase 1
5,7,3′,5′-Tetrahydroxyflavanone	PRD	5.4	5.6	---	---	5.3	---	Q965D5	EC, LD	Enoyl-acyl-carrier protein reductase
5,7,3′,5′-Tetrahydroxyflavanone	PRD	5.4	---	---	---	5.4	---	P14061	EC	Estradiol 17-β-dehydrogenase 1
5,7,3′,5′-Tetrahydroxyflavanone	PRD	5	---	---	---	5	---	P37059	EC	Estradiol 17-β-dehydrogenase 2
5,7,3′,5′-Tetrahydroxyflavanone	PRD	5.3	---	---	---	5.3	---	P03372	NR, TR	Estrogen receptor
5,7,3′,5′-Tetrahydroxyflavanone	PRD	5.9	---	---	---	5.9	---	Q92731	NR, TR	Estrogen receptor β
5,7,3′,5′-Tetrahydroxyflavanone	PRD	5	---	---	---	5	---	P14635	DL	G2/mitotic-specific cyclin-B1
5,7,3′,5′-Tetrahydroxyflavanone	PRD	5	---	---	---	5	---	O95067	DL	G2/mitotic-specific cyclin-B2
5,7,3′,5′-Tetrahydroxyflavanone	PRD	5	---	---	---	5	---	Q8WWL7	DL	G2/mitotic-specific cyclin-B3
5,7,3′,5′-Tetrahydroxyflavanone	PRD	5.9	---	---	---	5.9	---	P49840	EC, KC	Glycogen synthase kinase-3α
5,7,3′,5′-Tetrahydroxyflavanone	PRD	5.6	---	---	---	5.9	5.3	P49841	EC, KC	Glycogen synthase kinase-3β
5,7,3′,5′-Tetrahydroxyflavanone	PRD	5.9	---	5.9	---	---	---	Q9AIU0	UC	HTH-type transcriptional regulator ttgR
5,7,3′,5′-Tetrahydroxyflavanone	PRD	5.5	---	---	---	5.5	---	Q07820	UC	Induced myeloid leukemia cell differentiation protein Mcl-1
5,7,3′,5′-Tetrahydroxyflavanone	PRD	5.3	---	---	---	5.3	4.3	P03070	EC	Large T antigen
5,7,3′,5′-Tetrahydroxyflavanone	PRD	5.5	---	---	---	5.5	---	Q06327	EC	Linoleate 9*S*-lipoxygenase 1
5,7,3′,5′-Tetrahydroxyflavanone	PRD	5.9	---	---	---	5.9	---	Q8I2J3	EC, PS	M18 aspartyl aminopeptidase
5,7,3′,5′-Tetrahydroxyflavanone	PRD	A	---	---	---	---	---	P55157	UC	Microsomal triglyceride transfer protein large subunit
5,7,3′,5′-Tetrahydroxyflavanone	PRD	A	---	---	---	---	---	P08183	AN, EC, TC	Multidrug resistance protein 1
5,7,3′,5′-Tetrahydroxyflavanone	PRD	5.6	5.6	---	---	---	---	P33527	TC	Multidrug resistance-associated protein 1
5,7,3′,5′-Tetrahydroxyflavanone	PRD	5.3	---	5.3	---	---	---	Q88N29	TR	Probable HTH-type transcriptional regulator ttgR
5,7,3′,5′-Tetrahydroxyflavanone	PRD	5.4	---	---	---	---	5.4	P0A7G6	DR	Protein RecA
5,7,3′,5′-Tetrahydroxyflavanone	PRD	5.7	---	---	---	5.7	---	P11309	EC, KC	Proto-oncogene serine/ threonine-protein kinase pim-1
5,7,3′,5′-Tetrahydroxyflavanone	PRD	5.3	---	---	---	5.3	---	P29768	BT, EC	Sialidase
5,7,3′,5′-Tetrahydroxyflavanone	PRD	A	---	---	---	---	---	O94956	UC	Solute carrier organic anion transporter family member 2B1

Table S2. *Cont.*

MOLID	AN	pACT	p*Ki*	p*Kd*	p*Kb*	pIC50	pEC50	Uniprot	Functional	TARGET_NAME
5,7,3′,5′-Tetrahydroxyflavanone	PRD	A	---	---	---	---	---	P35610	EC	Sterol *O*-acyltransferase 1
5,7,3′,5′-Tetrahydroxyflavanone	PRD	A	---	---	---	---	---	O75908	EC	Sterol *O*-acyltransferase 2
5,7,3′,5′-Tetrahydroxyflavanone	PRD	6.3	---	---	---	6.3	---	O14746	DL, EC	Telomerase reverse transcriptase
5,7,3′,5′-Tetrahydroxyflavanone	PRD	A	---	---	---	---	---	Q6NUS8	EC	UDP-glucuronosyltransferase 3A1
Kushennol F	PRD	5.6	5.8	---	---	5.3	---	P56817	EC, PS, TC	β-secretase 1
Kushennol F	PRD	5.1	---	---	---	5.1	---	P27815	EC	cAMP-specific 3′,5′-cyclic phosphodiesterase 4A
Kushennol F	PRD	5.1	---	---	---	5.1	---	Q07343	EC	cAMP-specific 3′,5′-cyclic phosphodiesterase 4B
Kushennol F	PRD	5.1	---	---	---	5.1	---	Q08493	EC	cAMP-specific 3′,5′-cyclic phosphodiesterase 4C
Kushennol F	PRD	5.1	---	---	---	5.1	---	Q08499	EC	cAMP-specific 3′,5′-cyclic phosphodiesterase 4D
Kushennol F	PRD	6.8	---	---	---	6.8	---	P43235	EC, FD, PS	Cathepsin K
Kushennol F	PRD	5.9	---	---	---	5.9	---	Q14432	EC	cGMP-inhibited 3′,5′-cyclic phosphodiesterase A
Kushennol F	PRD	5.9	---	---	---	5.9	---	Q13370	EC	cGMP-inhibited 3′,5′-cyclic phosphodiesterase B
Kushennol F	PRD	6.2	---	---	---	6.2	---	O76074	EC	cGMP-specific 3′,5′-cyclic phosphodiesterase
Kushennol F	PRD	5.6	---	---	---	5.6	5.6	P03372	NR, TR	Estrogen receptor
Kushennol F	PRD	6.2	---	---	---	6.2	---	P23219	EC	Prostaglandin G/H synthase 1
Kushennol F	PRD	5.4	---	---	---	5.4	---	P31639	UC	Sodium/glucose cotransporter 2
Sophoraflavanone G	EXP	5.6	6	---	---	5.3	---	P56817	EC, PS, TC	β-secretase 1
Sophoraflavanone G	PRD	5.1	---	---	---	5.1	---	P27815	EC	cAMP-specific 3′,5′-cyclic phosphodiesterase 4A
Sophoraflavanone G	PRD	5.1	---	---	---	5.1	---	Q07343	EC	cAMP-specific 3′,5′-cyclic phosphodiesterase 4B
Sophoraflavanone G	PRD	5.1	---	---	---	5.1	---	Q08493	EC	cAMP-specific 3′,5′-cyclic phosphodiesterase 4C
Sophoraflavanone G	PRD	5.1	---	---	---	5.1	---	Q08499	EC	cAMP-specific 3′,5′-cyclic phosphodiesterase 4D
Sophoraflavanone G	PRD	6.8	---	---	---	6.8	---	P43235	EC, FD, PS	Cathepsin K
Sophoraflavanone G	PRD	5.9	---	---	---	5.9	---	Q14432	EC	cGMP-inhibited 3′,5′-cyclic phosphodiesterase A
Sophoraflavanone G	PRD	5.9	---	---	---	5.9	---	Q13370	EC	cGMP-inhibited 3′,5′-cyclic phosphodiesterase B
Sophoraflavanone G	PRD	6.2	---	---	---	6.2	---	O76074	EC	cGMP-specific 3′,5′-cyclic phosphodiesterase
Sophoraflavanone G	PRD	5.6	---	---	---	5.6	5.6	P03372	NR, TR	Estrogen receptor
Sophoraflavanone G	PRD	6.2	---	---	---	6.2	---	P23219	EC	Prostaglandin G/H synthase 1
Sophoraflavanone G	EXP	5.4	---	---	---	5.4	---	P31639	UC	Sodium/glucose cotransporter 2
Kurarinone	EXP	5.5	5.1	---	---	5.5	---	P56817	EC, PS, TC	β-secretase 1
Kurarinone	PRD	5.1	---	---	---	5.1	---	P27815	EC	cAMP-specific 3′,5′-cyclic phosphodiesterase 4A
Kurarinone	PRD	5.1	---	---	---	5.1	---	Q07343	EC	cAMP-specific 3′,5′-cyclic phosphodiesterase 4B
Kurarinone	PRD	5.1	---	---	---	5.1	---	Q08493	EC	cAMP-specific 3′,5′-cyclic phosphodiesterase 4C
Kurarinone	PRD	5.1	---	---	---	5.1	---	Q08499	EC	cAMP-specific 3′,5′-cyclic phosphodiesterase 4D
Kurarinone	PRD	5.9	---	---	---	5.9	---	Q14432	EC	cGMP-inhibited 3′,5′-cyclic phosphodiesterase A
Kurarinone	PRD	5.9	---	---	---	5.9	---	Q13370	EC	cGMP-inhibited 3′,5′-cyclic phosphodiesterase B
Kurarinone	PRD	6.2	---	---	---	6.2	---	O76074	EC	cGMP-specific 3′,5′-cyclic phosphodiesterase

Table S2. *Cont.*

MOLID	AN	pACT	p*K*i	p*K*d	p*K*b	pIC50	pEC50	Uniprot	Functional	TARGET_NAME
Kurarinone	PRD	5.5	---	---	---	5.5	---	P11511	CP, EC	Cytochrome P450 19A1
Kurarinone	EXP	5.6	---	---	---	5.6	5.6	P03372	NR, TR	Estrogen receptor
Kurarinone	PRD	5.2	---	---	---	5.2	---	P04054	EC	Phospholipase A2
Kurarinone	EXP	6.2	---	---	---	6.2	---	P23219	EC	Prostaglandin G/H synthase 1
Kurarinone	PRD	5.1	---	---	---	5.1	---	Q9BQF6	BQ, EC, PS	Sentrin-specific protease 7
Kurarinone	EXP	5.8	---	---	---	5.8	---	P31639	UC	Sodium/glucose cotransporter 2
Kurarinone	PRD	5.7	---	---	---	---	5.7	P10520	EC	Streptokinase A
Leachianone A	EXP	5.2	5.4	---	---	5.1	---	P56817	EC, PS, TC	β-secretase 1
Leachianone A	PRD	6.4	---	---	---	6.4	---	P11511	CP, EC	Cytochrome P450 19A1
Leachianone A	PRD	5.7	---	---	---	5.7	5.6	P03372	NR, TR	Estrogen receptor
Leachianone A	PRD	7.2	---	---	---	7.2	---	Q92731	NR, TR	Estrogen receptor β
Leachianone A	PRD	5.2	---	---	---	5.2	---	P04054	EC	Phospholipase A2
Leachianone A	PRD	6.2	---	---	---	6.2	---	P23219	EC	Prostaglandin G/H synthase 1
Leachianone A	PRD	5.1	---	---	---	5.1	---	Q9BQF6	BQ, EC, PS	Sentrin-specific protease 7
Leachianone A	PRD	5.7	---	---	---	5.7	---	P31639	UC	Sodium/glucose cotransporter 2
Leachianone A	PRD	5.6	---	---	---	---	5.6	P10520	EC	Streptokinase A
Luteolin 7-*O*-neohesperidoside	PRD	A	---	---	---	---	---	P42330	EC	Aldo-keto reductase family 1 member C3
Luteolin 7-*O*-neohesperidoside	PRD	8.1	8.1	---	---	---	---	P08913	GR	α-2A adrenergic receptor
Luteolin 7-*O*-neohesperidoside	PRD	7.3	7.3	---	---	---	---	P18825	GR	α-2C adrenergic receptor
Luteolin 7-*O*-neohesperidoside	PRD	5	---	---	---	5	---	Q02410	UC	Amyloid β A4 precursor protein-binding family A member 1
Luteolin 7-*O*-neohesperidoside	PRD	5.3	---	---	---	5.3	---	P11511	CP, EC	Cytochrome P450 19A1
Luteolin 7-*O*-neohesperidoside	PRD	A	---	---	---	---	---	P10632	CP, EC	Cytochrome P450 2C8
Luteolin 7-*O*-neohesperidoside	PRD	A	---	---	---	---	---	P11712	CP, EC	Cytochrome P450 2C9
Luteolin 7-*O*-neohesperidoside	PRD	A	---	---	---	---	---	P10635	CP, EC	Cytochrome P450 2D6
Luteolin 7-*O*-neohesperidoside	PRD	5.1	---	---	---	5.1	---	P03362	EC	Gag-Pro-Pol polyprotein
Luteolin 7-*O*-neohesperidoside	PRD	7.5	7.5	---	---	---	---	P08912	GR	Muscarinic acetylcholine receptor M5
Luteolin 7-*O*-neohesperidoside	PRD	5	---	---	---	5	---	P17252	EC, KC	Protein kinase C αtype
Luteolin 7-*O*-neohesperidoside	PRD	5.1	---	---	---	5.1	---	P61088	BQ, DR, EC	Ubiquitin-conjugating enzyme E2 N
Luteolin 7-*O*-neohesperidoside	PRD	5	---	---	---	---	5	Q9XUB2	UC	Zinc finger protein mex-5
Luteolin-5-*O*-neohesperidoside	PRD	A	---	---	---	---	---	P42330	EC	Aldo-keto reductase family 1 member C3
Luteolin-5-*O*-neohesperidoside	PRD	8.1	8.1	---	---	---	---	P08913	GR	α-2A adrenergic receptor
Luteolin-5-*O*-neohesperidoside	PRD	7.3	7.3	---	---	---	---	P18825	GR	α-2C adrenergic receptor
Luteolin-5-*O*-neohesperidoside	PRD	5	---	---	---	5	---	Q02410	UC	Amyloid β A4 precursor protein-binding family A member 1
Luteolin-5-*O*-neohesperidoside	PRD	5.3	---	---	---	5.3	---	P11511	CP, EC	Cytochrome P450 19A1

Table S2. *Cont.*

MOLID	AN	pACT	p*Ki*	p*Kd*	p*Kb*	pIC50	pEC50	Uniprot	Functional	TARGET_NAME
Luteolin-5-*O*-neohesperidoside	PRD	A	---	---	---	---	---	P10632	CP, EC	Cytochrome P450 2C8
Luteolin-5-*O*-neohesperidoside	PRD	A	---	---	---	---	---	P11712	CP, EC	Cytochrome P450 2C9
Luteolin-5-*O*-neohesperidoside	PRD	A	---	---	---	---	---	P10635	CP, EC	Cytochrome P450 2D6
Luteolin-5-*O*-neohesperidoside	PRD	5.1	---	---	---	5.1	---	P03362	EC	Gag-Pro-Pol polyprotein
Luteolin-5-*O*-neohesperidoside	PRD	7.4	7.4	---	---	---	---	P08912	GR	Muscarinic acetylcholine receptor M5
Luteolin-5-*O*-neohesperidoside	PRD	5	---	---	---	5	---	P17252	EC, KC	Protein kinase C α type
Luteolin-5-*O*-neohesperidoside	PRD	5.1	---	---	---	5.1	---	P61088	BQ, DR, EC	Ubiquitin-conjugating enzyme E2 N
Luteolin-5-*O*-neohesperidoside	PRD	5	---	---	---	---	5	Q9XUB2	UC	Zinc finger protein mex-5
Kaempferol-7-*O*-α-L-arabinofuranoside	PRD	A	---	---	---	---	---	P05091	EC	Aldehyde dehydrogenase, mitochondrial
Kaempferol-7-*O*-α-L-arabinofuranoside	PRD	5.5	---	---	---	5.5	---	P15121	EC	Aldose reductase
Kaempferol-7-*O*-α-L-arabinofuranoside	PRD	A	---	---	---	---	---	Q9UNQ0	AN, TC	ATP-binding cassette sub-family G member 2
Kaempferol-7-*O*-α-L-arabinofuranoside	PRD	5.8	---	---	---	5.8	---	P05113	CY	Interleukin-5
Kaempferol-7-*O*-α-L-arabinofuranoside	PRD	5.5	5.5	---	---	---	---	Q9HAS3	UC	Solute carrier family 28 member 3
8-Prenylapigenin	PRD	A	---	---	---	---	---	Q6NVY1	EC	3-Hydroxyisobutyryl-CoA hydrolase, mitochondrial
8-Prenylapigenin	PRD	5.3	5.8	---	---	5.3	---	Q965D6	EC, LD	3-Oxoacyl-acyl-carrier protein reductase
8-Prenylapigenin	PRD	7.3	7.3	---	---	---	---	P21589	AN, EC	5′-Nucleotidase
8-Prenylapigenin	PRD	5.4	5.4	---	---	---	---	P30542	GR	Adenosine receptor A1
8-Prenylapigenin	PRD	5.2	5	---	---	5.2	---	Q91WR5	EC	Aldo-keto reductase family 1 member C21
8-Prenylapigenin	PRD	5.6	---	---	---	5.6	---	P15121	EC	Aldose reductase
8-Prenylapigenin	PRD	6.7	---	---	---	6.7	---	P21397	EC	Amine oxidase [flavin-containing] A
8-Prenylapigenin	PRD	5.4	---	---	---	5.4	---	P49418	UC	Amphiphysin
8-Prenylapigenin	PRD	5	---	---	---	5	---	P10275	NR, TR	Androgen receptor
8-Prenylapigenin	PRD	6.2	---	---	---	6.2	---	P18054	EC	Arachidonate 12-lipoxygenase, 12*S*-type
8-Prenylapigenin	PRD	5.7	---	---	---	5.7	---	P16050	EC	Arachidonate 15-lipoxygenase
8-Prenylapigenin	PRD	5.7	---	---	---	5.7	---	P09917	EC	Arachidonate 5-lipoxygenase
8-Prenylapigenin	PRD	7.5	---	---	---	7.5	---	P35869	TR	Aryl hydrocarbon receptor
8-Prenylapigenin	PRD	A	---	---	---	---	---	P25705	EC	ATP synthase subunit α, mitochondrial
8-Prenylapigenin	PRD	A	---	---	---	---	---	P06576	EC	ATP synthase subunit β, mitochondrial
8-Prenylapigenin	PRD	A	---	---	---	---	---	P36542	EC	ATP synthase subunit γ, mitochondrial
8-Prenylapigenin	PRD	A	---	---	---	---	---	Q9UNQ0	AN, TC	ATP-binding cassette sub-family G member 2
8-Prenylapigenin	PRD	5.4	---	---	---	5.4	---	P25910	EC	β-lactamase type II
8-Prenylapigenin	PRD	5.6	---	---	---	5.6	---	P27815	EC	cAMP-specific 3′,5′-cyclic phosphodiesterase 4A
8-Prenylapigenin	PRD	5.6	---	---	---	5.6	---	Q07343	EC	cAMP-specific 3′,5′-cyclic phosphodiesterase 4B
8-Prenylapigenin	PRD	5.6	---	---	---	5.6	---	Q08493	EC	cAMP-specific 3′,5′-cyclic phosphodiesterase 4C
8-Prenylapigenin	PRD	5.6	---	---	---	5.6	---	Q08499	EC	cAMP-specific 3′,5′-cyclic phosphodiesterase 4D

Table S2. *Cont.*

MOLID	AN	pACT	p*Ki*	p*Kd*	p*Kb*	pIC50	pEC50	Uniprot	Functional	TARGET_NAME
8-Prenylapigenin	PRD	5.6	5.6	---	---	---	---	P00915	EC	Carbonic anhydrase 1
8-Prenylapigenin	PRD	5	5	---	---	---	---	O43570	EC	Carbonic anhydrase 12
8-Prenylapigenin	PRD	5	5	---	---	---	---	Q8N1Q1	EC	Carbonic anhydrase 13
8-Prenylapigenin	PRD	5.3	5.3	---	---	---	---	Q9ULX7	EC	Carbonic anhydrase 14
8-Prenylapigenin	PRD	5.6	5.6	---	---	---	---	P00918	EC	Carbonic anhydrase 2
8-Prenylapigenin	PRD	5.1	5.1	---	---	---	---	P07451	EC	Carbonic anhydrase 3
8-Prenylapigenin	PRD	5.1	5.1	---	---	---	---	P22748	EC	Carbonic anhydrase 4
8-Prenylapigenin	PRD	5.2	5.2	---	---	---	---	P35218	EC	Carbonic anhydrase 5A, mitochondrial
8-Prenylapigenin	PRD	5.2	5.2	---	---	---	---	P23280	EC	Carbonic anhydrase 6
8-Prenylapigenin	PRD	5.3	5.3	---	---	---	---	P43166	EC	Carbonic anhydrase 7
8-Prenylapigenin	PRD	5.2	5.2	---	---	---	---	Q16790	EC	Carbonic anhydrase 9
8-Prenylapigenin	PRD	6	5.9	---	---	6	---	P68400	EC, KC	Casein kinase II subunit α
8-Prenylapigenin	PRD	5.7	---	---	---	5.7	---	P67870	UC	Casein kinase II subunit β
8-Prenylapigenin	PRD	6.4	---	---	---	6.4	---	Q14432	EC	cGMP-inhibited 3′,5′-cyclic phosphodiesterase A
8-Prenylapigenin	PRD	6.4	---	---	---	6.4	---	Q13370	EC	cGMP-inhibited 3′,5′-cyclic phosphodiesterase B
8-Prenylapigenin	PRD	6.5	8.3	---	---	6.4	---	O76074	EC	cGMP-specific 3′,5′-cyclic phosphodiesterase
8-Prenylapigenin	PRD	A	---	---	---	---	---	Q01043	UC	Cyclin homolog
8-Prenylapigenin	PRD	7	---	---	---	7	---	P11511	CP, EC	Cytochrome P450 19A1
8-Prenylapigenin	PRD	6.8	---	---	---	6.8	---	P04798	CP, EC	Cytochrome P450 1A1
8-Prenylapigenin	PRD	5.8	---	---	---	5.8	---	P05177	CP, EC	Cytochrome P450 1A2
8-Prenylapigenin	PRD	7.6	---	---	---	7.6	---	Q16678	CP, EC	Cytochrome P450 1B1
8-Prenylapigenin	PRD	A	---	---	---	---	---	P10632	CP, EC	Cytochrome P450 2C8
8-Prenylapigenin	PRD	5	5	---	---	---	---	P11712	CP, EC	Cytochrome P450 2C9
8-Prenylapigenin	PRD	5.3	---	---	---	5.3	---	P10635	CP, EC	Cytochrome P450 2D6
8-Prenylapigenin	PRD	6.6	6.6	---	---	---	---	P21917	GR	D_4 dopamine receptor
8-Prenylapigenin	PRD	6.2	---	---	---	6.2	---	P11387	DL, EC	DNA topoisomerase 1
8-Prenylapigenin	PRD	5.3	5.9	---	---	5.3	---	Q965D5	EC, LD	Enoyl-acyl-carrier protein reductase
8-Prenylapigenin	PRD	6	---	---	---	6	---	P14061	EC	Estradiol 17-β-dehydrogenase 1
8-Prenylapigenin	PRD	6.2	---	---	---	6.2	---	P37059	EC	Estradiol 17-β-dehydrogenase 2
8-Prenylapigenin	PRD	6.1	---	---	---	6.1	---	P03372	NR, TR	Estrogen receptor
8-Prenylapigenin	PRD	6.7	---	---	---	6.7	---	Q92731	NR, TR	Estrogen receptor β

Table S2. *Cont.*

MOLID	AN	pACT	p*K*i	p*K*d	p*K*b	pIC50	pEC50	Uniprot	Functional	TARGET_NAME
8-Prenylapigenin	PRD	5.3	5	---	---	5.2	---	P49327	EC, LD	Fatty acid synthase (EC 2.3.1.85) [Includes: [Acyl-carrier-protein] *S*-acetyltransferase (EC 2.3.1.38); [Acyl-carrier-protein] *S*-malonyltransferase (EC 2.3.1.39); 3-oxoacyl-[acyl-carrier-protein] synthase (EC 2.3.1.41); 3-oxoacyl-[acyl-carrier-protein] reductase (EC 1.1.1.100); 3-hydroxypalmitoyl-[acyl-carrier-protein] dehydratase (EC 4.2.1.61); Enoyl-[acyl-carrier-protein] reductase (EC 1.3.1.10); Oleoyl-[acyl-carrier-protein] hydrolase
8-Prenylapigenin	PRD	5.8	---	---	---	---	5.8	P08263	EC	Glutathione S-transferase A1
8-Prenylapigenin	PRD	5.8	---	---	---	5.8	---	P49840	EC, KC	Glycogen synthase kinase-3α
8-Prenylapigenin	PRD	5.5	---	---	---	5.8	5.1	P49841	EC, KC	Glycogen synthase kinase-3β
8-Prenylapigenin	PRD	A	---	---	---	---	---	Q9AIU0	UC	HTH-type transcriptional regulator ttgR
8-Prenylapigenin	PRD	5.5	---	---	---	5.5	---	Q07820	UC	Induced myeloid leukemia cell differentiation protein Mcl-1
8-Prenylapigenin	PRD	5.7	---	---	---	5.7	---	P09923	EC	Intestinal-type alkaline phosphatase
8-Prenylapigenin	PRD	5.5	---	---	---	5.5	---	Q06327	EC	Linoleate 9*S*-lipoxygenase 1
8-Prenylapigenin	PRD	6.4	---	---	---	6.4	---	Q8I2J3	EC, PS	M18 aspartyl aminopeptidase
8-Prenylapigenin	PRD	5.2	---	---	---	5.2	---	P40925	EC	Malate dehydrogenase, cytoplasmic
8-Prenylapigenin	PRD	A	---	---	---	---	---	P53985	TC	Monocarboxylate transporter 1
8-Prenylapigenin	PRD	A	---	---	---	---	---	O60669	TC	Monocarboxylate transporter 2
8-Prenylapigenin	PRD	A	---	---	---	---	---	P08183	AN, EC, TC	Multidrug resistance protein 1
8-Prenylapigenin	PRD	5.3	---	5.3	---	---	---	P21439	EC,TC	Multidrug resistance protein 3
8-Prenylapigenin	PRD	5.6	5.6	---	---	5.5	---	P33527	TC	Multidrug resistance-associated protein 1
8-Prenylapigenin	PRD	5.4	---	---	---	5.4	---	P27986	UC	Phosphatidylinositol 3-kinase regulatory subunit α
8-Prenylapigenin	PRD	5.4	---	---	---	5.4	---	O00459	UC	Phosphatidylinositol 3-kinase regulatory subunit β
8-Prenylapigenin	PRD	5.4	---	---	---	5.4	---	P42336	EC	Phosphatidylinositol-4,5-bisphosphate 3-kinase catalytic subunit α isoform
8-Prenylapigenin	PRD	5.4	---	---	---	5.4	---	P42338	EC	Phosphatidylinositol-4,5-bisphosphate 3-kinase catalytic subunit β isoform
8-Prenylapigenin	PRD	5.4	---	---	---	5.4	---	O00329	EC	Phosphatidylinositol-4,5-bisphosphate 3-kinase catalytic subunit δ isoform
8-Prenylapigenin	PRD	5.4	---	6.5	---	5.4	---	P48736	EC	Phosphatidylinositol-4,5-bisphosphate 3-kinase catalytic subunit γ isoform

Table S2. *Cont.*

MOLID	AN	pACT	p*K*i	p*K*d	p*K*b	pIC50	pEC50	Uniprot	Functional	TARGET_NAME
8-Prenylapigenin	PRD	5.7	---	---	---	5.7	---	P04054	EC	Phospholipase A2
8-Prenylapigenin	PRD	5.4	---	5.4	---	---	---	Q88N29	TR	Probable HTH-type transcriptional regulator ttgR
8-Prenylapigenin	PRD	6	---	7.6	---	5.9	---	P11309	EC, KC	Proto-oncogene serine/ threonine-protein kinase pim-1
8-Prenylapigenin	PRD	A	---	---	---	---	---	O94768	EC, KC	Serine/threonine-protein kinase 17B
8-Prenylapigenin	PRD	5.2	4.8	---	---	5.2	---	P29768	BT, EC	Sialidase
8-Prenylapigenin	PRD	A	---	---	---	---	---	O94956	UC	Solute carrier organic anion transporter family member 2B1
8-Prenylapigenin	PRD	5.7	---	---	---	---	5.7	P10520	EC	Streptokinase A
8-Prenylapigenin	PRD	A	---	---	---	---	---	P02766	UC	Transthyretin
8-Prenylapigenin	PRD	5.5	---	---	---	5.5	---	Q9H4B7	CK	Tubulin β-1 chain
8-Prenylapigenin	PRD	5.5	---	---	---	5.5	---	P29512	CK	Tubulin β-2/β-3 chain
8-Prenylapigenin	PRD	5.5	---	---	---	5.5	---	Q13509	CK	Tubulin β-3 chain
8-Prenylapigenin	PRD	5.5	---	---	---	5.5	---	P04350	CK	Tubulin β-4 chain
8-Prenylapigenin	PRD	5.5	---	---	---	5.5	---	Q3ZCM7	CK	Tubulin β-8 chain
8-Prenylapigenin	PRD	A	---	---	---	---	---	P08631	EC, KC	Tyrosine-protein kinase HCK
8-Prenylapigenin	PRD	A	---	---	---	---	---	Q6NUS8	EC	UDP-glucuronosyltransferase 3A1
8-Prenylapigenin	PRD	5.3	5.8	---	---	5.3	---	P47989	EC	Xanthine dehydrogenase/oxidase
Apigenine	PRD	A	---	---	---	---	---	Q6NVY1	EC	3-Hydroxyisobutyryl-CoA hydrolase, mitochondrial
Apigenine	EXP	5.4	---	---	---	5.4	---	Q965D6	EC, LD	3-Oxoacyl-acyl-carrier protein reductase
Apigenine	PRD	7.3	7.3	---	---	---	---	P21589	AN, EC	5′-Nucleotidase
Apigenine	PRD	5.6	5.6	---	---	---	---	P30542	GR	Adenosine receptor A1
Apigenine	PRD	5	5	---	---	---	---	P29274	GR	Adenosine receptor A2a
Apigenine	PRD	5.5	5.5	---	---	---	---	P29275	GR	Adenosine receptor A2b
Apigenine	PRD	5.2	5	---	---	5.2	---	Q91WR5	EC	Aldo-keto reductase family 1 member C21
Apigenine	EXP	5.9	---	---	---	5.9	---	P15121	EC	Aldose reductase
Apigenine	PRD	5.5	---	---	---	5.5	---	P21397	EC	Amine oxidase [flavin-containing] A
Apigenine	PRD	5.4	---	---	---	5.4	---	P49418	UC	Amphiphysin
Apigenine	EXP	5	---	---	---	5	---	P10275	NR, TR	Androgen receptor
Apigenine	PRD	6.1	---	---	---	6.1	---	P18054	EC	Arachidonate 12-lipoxygenase, 12*S*-type
Apigenine	EXP	5.7	---	---	---	5.7	---	P16050	EC	Arachidonate 15-lipoxygenase
Apigenine	EXP	5.6	---	---	---	5.6	---	P09917	EC	Arachidonate 5-lipoxygenase
Apigenine	EXP	7.5	---	---	---	7.5	---	P35869	TR	Aryl hydrocarbon receptor
Apigenine	PRD	A	---	---	---	---	---	P25705	EC	ATP synthase subunit α, mitochondrial
Apigenine	PRD	A	---	---	---	---	---	P06576	EC	ATP synthase subunit β, mitochondrial
Apigenine	PRD	A	---	---	---	---	---	P36542	EC	ATP synthase subunit γ, mitochondrial

Table S2. *Cont.*

MOLID	AN	pACT	p*Ki*	p*Kd*	p*Kb*	pIC50	pEC50	Uniprot	Functional	TARGET_NAME
Apigenine	PRD	A	---	---	---	---	---	Q9UNQ0	AN, TC	ATP-binding cassette sub-family G member 2
Apigenine	PRD	5.1	---	---	---	5.1	---	P08236	EC	β-glucuronidase
Apigenine	PRD	5.4	---	---	---	5.4	---	P25910	EC	β-lactamase type II
Apigenine	PRD	6.7	---	---	---	6.7	---	P09619	AN, CA, CR, EC, EL, KC	β-type platelet-derived growth factor receptor
Apigenine	PRD	5.6	5.6	---	---	---	---	P00915	EC	Carbonic anhydrase 1
Apigenine	PRD	5	5	---	---	---	---	O43570	EC	Carbonic anhydrase 12
Apigenine	PRD	5	5	---	---	---	---	Q8N1Q1	EC	Carbonic anhydrase 13
Apigenine	PRD	5.3	5.3	---	---	---	---	Q9ULX7	EC	Carbonic anhydrase 14
Apigenine	PRD	5.6	5.6	---	---	---	---	P00918	EC	Carbonic anhydrase 2
Apigenine	PRD	5.1	5.1	---	---	---	---	P07451	EC	Carbonic anhydrase 3
Apigenine	PRD	5.1	5.1	---	---	---	---	P22748	EC	Carbonic anhydrase 4
Apigenine	PRD	5.2	5.2	---	---	---	---	P35218	EC	Carbonic anhydrase 5A, mitochondrial
Apigenine	PRD	5.2	5.2	---	---	---	---	P23280	EC	Carbonic anhydrase 6
Apigenine	PRD	5.3	5.3	---	---	---	---	P43166	EC	Carbonic anhydrase 7
Apigenine	PRD	5.2	5.2	---	---	---	---	Q16790	EC	Carbonic anhydrase 9
Apigenine	PRD	5.9	6	---	---	5.8	---	P68400	EC, KC	Casein kinase II subunit α
Apigenine	PRD	5.5	---	---	---	5.5	---	P67870	UC	Casein kinase II subunit β
Apigenine	PRD	A	---	---	---	---	---	Q01043	UC	Cyclin homolog
Apigenine	PRD	5.6	4	---	---	7	---	P11511	CP, EC	Cytochrome P450 19A1
Apigenine	EXP	6.2	---	---	---	6.2	---	P04798	CP, EC	Cytochrome P450 1A1
Apigenine	EXP	6.1	---	---	---	6.1	---	P05177	CP, EC	Cytochrome P450 1A2
Apigenine	EXP	7.3	---	---	---	7.3	---	Q16678	CP, EC	Cytochrome P450 1B1
Apigenine	PRD	A	---	---	---	---	---	P10632	CP, EC	Cytochrome P450 2C8
Apigenine	EXP	5.2	5.2	---	---	---	---	P11712	CP, EC	Cytochrome P450 2C9
Apigenine	PRD	5.3	---	---	---	5.3	---	P10635	CP, EC	Cytochrome P450 2D6
Apigenine	PRD	6.6	6.6	---	---	---	---	P21917	GR	D_4 dopamine receptor
Apigenine	PRD	5.9	---	---	---	5.9	---	P11387	DL, EC	DNA topoisomerase 1
Apigenine	PRD	7.6	---	---	---	7.6	---	P34021	NR, TR	Ecdysone receptor
Apigenine	EXP	6	---	---	---	6	---	P14061	EC	Estradiol 17-β-dehydrogenase 1
Apigenine	EXP	6.4	---	---	---	6.4	---	P37059	EC	Estradiol 17-β-dehydrogenase 2
Apigenine	PRD	5.4	6.4	6.2	---	5.4	6.5	P03372	NR, TR	Estrogen receptor
Apigenine	PRD	6.4	8.2	6.2	---	6.3	7.2	Q92731	NR, TR	Estrogen receptor β

Table S2. *Cont.*

MOLID	AN	pACT	p*K*i	p*K*d	p*K*b	pIC50	pEC50	Uniprot	Functional	TARGET_NAME
Apigenine	PRD	5.3	5	---	---	5.1	---	P49327	EC, LD	Fatty acid synthase (EC 2.3.1.85) [Includes: [Acyl-carrier-protein] *S*-acetyltransferase (EC 2.3.1.38); [Acyl-carrier-protein] *S*-malonyltransferase (EC 2.3.1.39); 3-oxoacyl-[acyl-carrier-protein] synthase (EC 2.3.1.41); 3-oxoacyl-[acyl-carrier-protein] reductase (EC 1.1.1.100); 3-hydroxypalmitoyl-[acyl-carrier-protein] dehydratase (EC 4.2.1.61); Enoyl-[acyl-carrier-protein] reductase (EC 1.3.1.10); Oleoyl-[acyl-carrier-protein] hydrolase
Apigenine	PRD	5.3	5.3	---	---	---	---	P47869	IC, TC	γ-aminobutyric acid receptor subunit α-2
Apigenine	PRD	5.3	5.3	---	---	---	---	P34903	IC	γ-aminobutyric acid receptor subunit α-3
Apigenine	PRD	5.3	5.3	---	---	---	---	P48169	IC	γ-aminobutyric acid receptor subunit α-4
Apigenine	PRD	5.3	5.3	---	---	---	---	P31644	IC	γ-aminobutyric acid receptor subunit α-5
Apigenine	PRD	5.3	5.3	---	---	---	---	Q16445	IC	γ-aminobutyric acid receptor subunit α-6
Apigenine	PRD	6.1	6.1	---	---	---	---	P18505	IC	γ-aminobutyric acid receptor subunit β-1
Apigenine	PRD	6.1	6.1	---	---	---	---	P47870	IC, TC	γ-aminobutyric acid receptor subunit β-2
Apigenine	PRD	6.1	6.1	---	---	---	---	P28472	IC	γ-aminobutyric acid receptor subunit β-3
Apigenine	PRD	6.1	6.1	---	---	---	---	O14764	IC	γ-aminobutyric acid receptor subunit δ
Apigenine	PRD	6.1	6.1	---	---	---	---	P78334	IC	γ-aminobutyric acid receptor subunit ε
Apigenine	PRD	6.1	6.1	---	---	---	---	Q8N1C3	IC	γ-aminobutyric acid receptor subunit γ-1
Apigenine	PRD	6.1	6.1	---	---	---	---	P18507	IC	γ-aminobutyric acid receptor subunit γ-2
Apigenine	PRD	6.1	6.1	---	---	---	---	Q99928	IC	γ-aminobutyric acid receptor subunit γ-3
Apigenine	PRD	6.1	6.1	---	---	---	---	O00591	IC	γ-aminobutyric acid receptor subunit π
Apigenine	PRD	6.1	6.1	---	---	---	---	Q9UN88	IC	γ-aminobutyric acid receptor subunit θ
Apigenine	PRD	5.8	---	---	---	---	5.8	P08263	EC	Glutathione S-transferase A1
Apigenine	EXP	5.5	---	---	---	5.5	---	P49840	EC, KC	Glycogen synthase kinase-3α
Apigenine	EXP	5.2	---	---	---	5.5	4.9	P49841	EC, KC	Glycogen synthase kinase-3β
Apigenine	PRD	A	---	---	---	---	---	Q9AIU0	UC	HTH-type transcriptional regulator ttgR
Apigenine	PRD	5.6	---	---	---	5.6	---	Q07820	UC	Induced myeloid leukemia cell differentiation protein Mcl-1
Apigenine	PRD	5.3	---	---	---	5.5	4.2	P09923	EC	Intestinal-type alkaline phosphatase
Apigenine	PRD	5.5	---	---	---	5.5	---	Q06327	EC	Linoleate 9*S*-lipoxygenase 1
Apigenine	PRD	6.4	---	---	---	6.4	---	Q8I2J3	EC, PS	M18 aspartyl aminopeptidase

Table S2. *Cont.*

MOLID	AN	pACT	p*Ki*	p*Kd*	p*Kb*	pIC50	pEC50	Uniprot	Functional	TARGET_NAME
Apigenine	PRD	7.4	7.4	---	---	---	---	P14174	EC	Macrophage migration inhibitory factor
Apigenine	PRD	5.2	---	---	---	5.2	---	P40925	EC	Malate dehydrogenase, cytoplasmic
Apigenine	PRD	A	---	---	---	---	---	P53985	TC	Monocarboxylate transporter 1
Apigenine	PRD	A	---	---	---	---	---	O60669	TC	Monocarboxylate transporter 2
Apigenine	PRD	5.2	---	5.3	---	4.3	---	P08183	AN, EC, TC	Multidrug resistance protein 1
Apigenine	EXP	5.2	---	5.2	---	---	---	P21439	EC, TC	Multidrug resistance protein 3
Apigenine	EXP	5.6	5.6	---	---	---	---	P33527	TC	Multidrug resistance-associated protein 1
Apigenine	PRD	A	---	---	---	---	---	Q15788	CH, EC, TR	Nuclear receptor coactivator 1
Apigenine	PRD	A	---	---	---	---	---	Q15596	CH, TR	Nuclear receptor coactivator 2
Apigenine	PRD	5.4	---	---	---	5.4	---	P27986	UC	Phosphatidylinositol 3-kinase regulatory subunit α
Apigenine	PRD	5.4	---	---	---	5.4	---	O00459	UC	Phosphatidylinositol 3-kinase regulatory subunit β
Apigenine	PRD	5.4	---	---	---	5.4	---	P42336	EC	Phosphatidylinositol-4,5-bisphosphate 3-kinase catalytic subunit α isoform
Apigenine	PRD	5.4	---	---	---	5.4	---	P42338	EC	Phosphatidylinositol-4,5-bisphosphate 3-kinase catalytic subunit β isoform
Apigenine	PRD	5.4	---	---	---	5.4	---	O00329	EC	Phosphatidylinositol-4,5-bisphosphate 3-kinase catalytic subunit δ isoform
Apigenine	PRD	5.4	---	6.5	---	5.4	---	P48736	EC	Phosphatidylinositol-4,5-bisphosphate 3-kinase catalytic subunit γ isoform
Apigenine	PRD	5.7	---	---	---	5.7	---	P04054	EC	Phospholipase A2
Apigenine	PRD	5.1	---	5.1	---	---	---	Q88N29	TR	Probable HTH-type transcriptional regulator ttgR
Apigenine	PRD	A	---	---	---	---	---	Q14289	EC, KC	Protein-tyrosine kinase 2-β
Apigenine	EXP	5.9	---	---	---	5.9	---	P11309	EC, KC	Proto-oncogene serine/ threonine-protein kinase pim-1
Apigenine	PRD	6.6	---	---	---	6.6	---	P36888	AN, CR, EC, EL, KC	Receptor-type tyrosine-protein kinase FLT3
Apigenine	PRD	A	---	---	---	---	---	O94768	EC, KC	Serine/threonine-protein kinase 17B
Apigenine	PRD	5	---	---	---	5	---	P42345	EC, KC	Serine/threonine-protein kinase mTOR
Apigenine	EXP	5.1	---	---	---	5.1	---	P29768	BT, EC	Sialidase
Apigenine	PRD	A	---	---	---	---	---	O94956	UC	Solute carrier organic anion transporter family member 2B1
Apigenine	PRD	8	---	---	---	8	---	P11474	NR, TR	Steroid hormone receptor ERR1
Apigenine	PRD	6.4	---	---	---	6.4	---	O95718	NR, TR	Steroid hormone receptor ERR2
Apigenine	PRD	6.1	---	---	---	6.1	---	P14410	EC	Sucrase-isomaltase, intestinal
Apigenine	PRD	5.7	---	---	---	5.7	---	O14746	DL, EC	Telomerase reverse transcriptase
Apigenine	PRD	A	---	---	---	---	---	P02766	UC	Transthyretin

Table S2. *Cont.*

MOLID	AN	pACT	p*Ki*	p*Kd*	p*Kb*	pIC50	pEC50	Uniprot	Functional	TARGET_NAME
Apigenine	PRD	5.5	---	---	---	5.5	---	Q9H4B7	CK	Tubulin β-1 chain
Apigenine	PRD	5.5	---	---	---	5.5	---	P29512	CK	Tubulin beta-2/β-3 chain
Apigenine	PRD	5.5	---	---	---	5.5	---	Q13509	CK	Tubulin β-3 chain
Apigenine	PRD	5.5	---	---	---	5.5	---	P04350	CK	Tubulin β-4 chain
Apigenine	PRD	5.5	---	---	---	5.5	---	Q3ZCM7	CK	Tubulin β-8 chain
Apigenine	PRD	A	---	---	---	---	---	P08631	EC, KC	Tyrosine-protein kinase HCK
Apigenine	EXP	A	---	---	---	---	---	Q6NUS8	EC	UDP-glucuronosyltransferase 3A1
Apigenine	EXP	6	---	---	---	6	---	P47989	EC	Xanthine dehydrogenase/oxidase
Kaempferol-3-*O*-α-L-rhamnopyranoside	PRD	5.4	---	---	---	5.4	---	P15121	EC	Aldose reductase
Kaempferol-3-*O*-α-L-rhamnopyranoside	PRD	5.4	5.4	---	---	---	---	P18825	GR	α-2C adrenergic receptor
Kaempferol-3-*O*-α-L-rhamnopyranoside	PRD	6.3	---	---	---	6.3	---	O76074	EC	cGMP-specific 3',5'-cyclic phosphodiesterase
Kaempferol-3-*O*-α-L-rhamnopyranoside	PRD	5.2	5.2	---	---	4.7	---	P03372	NR, TR	Estrogen receptor
Kaempferol-3-*O*-α-L-rhamnopyranoside	PRD	5.4	---	---	---	5.4	---	P09923	EC	Intestinal-type alkaline phosphatase
Kaempferol-3-*O*-α-L-rhamnopyranoside	PRD	5.5	---	---	---	5.5	---	P03070	EC	Large T antigen
Astragalin	PRD	A	---	---	---	---	---	P05091	EC	Aldehyde dehydrogenase, mitochondrial
Astragalin	EXP	5.3	---	---	---	5.3	---	P15121	EC	Aldose reductase
Astragalin	PRD	5.6	---	---	---	5.6	---	P10696	EC	Alkaline phosphatase, placental-like
Astragalin	PRD	7	7	---	---	---	---	P18825	GR	α-2C adrenergic receptor
Astragalin	PRD	A	---	---	---	---	---	Q9UNQ0	AN, TC	ATP-binding cassette sub-family G member 2
Astragalin	PRD	5.2	5.2	---	---	4.7	---	P03372	NR, TR	Estrogen receptor
Astragalin	PRD	5.5	---	---	---	5.5	---	P09923	EC	Intestinal-type alkaline phosphatase
Astragalin	PRD	5.2	---	---	---	5.2	---	P03070	EC	Large T antigen
Astragalin	PRD	5.7	---	---	---	5.7	---	O14757	DR, EC, KC	Serine/threonine-protein kinase Chk1
Kaempferol 3-*O*-β-D-glucopyranoside-7-*O*-α-L-arabinofuranoside	PRD	5.2	---	---	---	5.2	---	O76074	EC	cGMP-specific 3′,5′-cyclic phosphodiesterase
Kaempferol 3-*O*-β-D-glucopyranoside-7-*O*-α-L-arabinofuranoside	PRD	A	---	---	---	---	---	Q9GZQ4	GR	Neuromedin-U receptor 2
5,7-Dihydroxychromone-7-*O*-β-D-glucopyranoside	PRD	6.1	6.5	---	---	6.1	---	P22303	EC	Acetylcholinesterase
5,7-Dihydroxychromone-7-*O*-β-D-glucopyranoside	PRD	A	---	---	---	---	---	P01556	BT	Cholera enterotoxin subunit B
5,7-Dihydroxychromone-7-*O*-neohesperidoside	PRD	A	---	---	---	---	---	P32890	UC	Heat-labile enterotoxin B chain
5,7-Dihydroxychromone-7-*O*-neohesperidoside	PRD	6.7	---	---	---	6.7	6.5	P31639	UC	Sodium/glucose cotransporter 2

Table S2. *Cont.*

MOLID	AN	pACT	p*Ki*	p*Kd*	p*Kb*	pIC50	pEC50	Uniprot	Functional	TARGET_NAME
5,7-Dihydroxychromone-7-*O*-neohesperidoside	PRD	A	---	---	---	---	---	Q9Y271	GR	Cysteinyl leukotriene receptor 1
5,7-Dihydroxychromone-7-*O*-neohesperidoside	PRD	A	---	---	---	---	---	Q9NS75	GR	Cysteinyl leukotriene receptor 2
5,7-Dihydroxychromone-7-*O*-neohesperidoside	PRD	5.3	---	---	---	5.3	---	P11511	CP, EC	Cytochrome P450 19A1
5,7-Dihydroxychromone-7-*O*-neohesperidoside	PRD	5.8	---	5.8	---	---	---	O00182	GL	Galectin-9
5,7-Dihydroxychromone-7-*O*-neohesperidoside	PRD	5.1	---	---	---	5.1	---	P61088	BQ, DR, EC	Ubiquitin-conjugating enzyme E2 N
kaempferol 3-*O*-β-D-glucopyranoside-7-*O*-β-D-glucopyranoside	PRD	5.2	---	---	---	5.2	---	O76074	EC	cGMP-specific 3′,5′-cyclic phosphodiesterase
kaempferol 3-*O*-β-D-glucopyranoside-7-*O*-β-D-glucopyranoside	PRD	A	---	---	---	---	---	Q9GZQ4	GR	Neuromedin-U receptor 2
Xanthohumol	PRD	5.1	5.1	---	---	---	---	P28222	GR	5-Hydroxytryptamine receptor 1B
Xanthohumol	PRD	6.3	6.3	---	---	---	---	P28223	GR	5-Hydroxytryptamine receptor 2A
Xanthohumol	PRD	5.2	5.2	---	---	---	---	P41595	GR	5-Hydroxytryptamine receptor 2B
Xanthohumol	PRD	5.9	5.9	---	---	---	---	P28335	GR	5-Hydroxytryptamine receptor 2C
Xanthohumol	PRD	5.8	5.8	---	---	---	---	P30542	GR	Adenosine receptor A1
Xanthohumol	PRD	5.2	5.2	---	---	---	---	P29274	GR	Adenosine receptor A2a
Xanthohumol	PRD	5.6	---	---	---	5.6	---	P15121	EC	Aldose reductase
Xanthohumol	PRD	8.1	---	---	---	8.1	---	P09917	EC	Arachidonate 5-lipoxygenase
Xanthohumol	PRD	5.9	---	---	---	5.9	---	O76074	EC	cGMP-specific 3′,5′-cyclic phosphodiesterase
Xanthohumol	PRD	7	---	---	---	7	---	P04798	CP, EC	Cytochrome P450 1A1
Xanthohumol	PRD	5.8	---	---	---	5.8	---	P05177	CP, EC	Cytochrome P450 1A2
Xanthohumol	PRD	7.6	---	---	---	7.6	---	Q16678	CP, EC	Cytochrome P450 1B1
Xanthohumol	PRD	5.3	---	---	---	5.3	---	P10635	CP, EC	Cytochrome P450 2D6
Xanthohumol	PRD	5.5	---	---	---	5.5	---	P00533	CR, EC, EL, KC	Epidermal growth factor receptor
Xanthohumol	PRD	5.7	---	---	---	5.7	---	P03372	NR, TR	Estrogen receptor
Xanthohumol	PRD	6.9	---	---	---	6.9	---	Q92731	NR, TR	Estrogen receptor β
Xanthohumol	PRD	6	6	---	---	---	---	P14867	IC	γ-aminobutyric acid receptor subunit α-1
Xanthohumol	PRD	6	6	---	---	---	---	P47869	IC, TC	γ-aminobutyric acid receptor subunit α-2
Xanthohumol	PRD	6	6	---	---	---	---	P34903	IC	γ-aminobutyric acid receptor subunit α-3

Table S2. *Cont.*

MOLID	AN	pACT	p*K*i	p*K*d	p*K*b	pIC50	pEC50	Uniprot	Functional	TARGET_NAME
Xanthohumol	PRD	6	6	---	---	---	---	P48169	IC	γ-aminobutyric acid receptor subunit α-4
Xanthohumol	PRD	6	6	---	---	---	---	P31644	IC	γ-aminobutyric acid receptor subunit α-5
Xanthohumol	PRD	6	6	---	---	---	---	Q16445	IC	γ-aminobutyric acid receptor subunit α-6
Xanthohumol	PRD	5.8	---	---	---	---	5.8	P08263	EC	Glutathione S-transferase A1
Xanthohumol	PRD	A	---	---	---	---	---	Q9NZK7	EC	Group IIE secretory phospholipase A2
Xanthohumol	PRD	5.5	---	---	---	5.5	---	P33527	TC	Multidrug resistance-associated protein 1
Xanthohumol	PRD	5.1	---	---	---	5.1	---	Q9BQF6	BQ, EC, PS	Sentrin-specific protease 7
Xanthohumol	PRD	5.5	---	---	---	---	5.5	P10520	EC	Streptokinase A
Xanthohumol	PRD	5.5	---	---	---	5.5	---	Q71U36	CK	Tubulin α-1A chain
Xanthohumol	PRD	5	---	---	---	5	---	Q3ZCM7	CK	Tubulin β-8 chain
Xanthohumol	PRD	6.2	---	---	---	6.2	---	P47989	EC	Xanthine dehydrogenase/oxidase
Epicatechin	PRD	6.5	---	---	---	6.5	---	P11362	AN, CA, CL, CR, EC, EL, KC	Basic fibroblast growth factor receptor 1
Epicatechin	EXP	5.6	5.6	---	---	---	---	P00915	EC	Carbonic anhydrase 1
Epicatechin	EXP	5.3	5.3	---	---	---	---	O43570	EC	Carbonic anhydrase 12
Epicatechin	EXP	5.1	5.1	---	---	---	---	Q99N23	EC	Carbonic anhydrase 15
Epicatechin	EXP	5.7	5.7	---	---	---	---	P00918	EC	Carbonic anhydrase 2
Epicatechin	EXP	5.5	5.5	---	---	---	---	P07451	EC	Carbonic anhydrase 3
Epicatechin	EXP	5.3	5.3	---	---	---	---	P22748	EC	Carbonic anhydrase 4
Epicatechin	EXP	5.4	5.4	---	---	---	---	P35218	EC	Carbonic anhydrase 5A, mitochondrial
Epicatechin	EXP	5.4	5.4	---	---	---	---	Q9Y2D0	EC	Carbonic anhydrase 5B, mitochondrial
Epicatechin	EXP	5.3	5.3	---	---	---	---	P23280	EC	Carbonic anhydrase 6
Epicatechin	EXP	6.3	6.3	---	---	---	---	P43166	EC	Carbonic anhydrase 7
Epicatechin	EXP	5.3	5.3	---	---	---	---	Q16790	EC	Carbonic anhydrase 9
Epicatechin	PRD	5.7	---	---	---	5.7	---	P11511	CP, EC	Cytochrome P450 19A1
Epicatechin	PRD	5.9	---	---	---	5.9	---	Q16678	CP, EC	Cytochrome P450 1B1
Epicatechin	PRD	5.4	6.7	---	---	5.2	---	Q965D5	EC, LD	Enoyl-acyl-carrier protein reductase
Epicatechin	PRD	5.5	---	---	---	5.5	---	P00533	CR, EC, EL, KC	Epidermal growth factor receptor
Epicatechin	PRD	6.4	---	---	---	6.4	---	P08581	AN, CR, EC, EL, KC	Hepatocyte growth factor receptor
Epicatechin	PRD	5.5	---	---	---	5.5	---	Q07820	UC	Induced myeloid leukemia cell differentiation protein Mcl-1
Epicatechin	EXP	5.1	---	---	---	5.1	---	P03070	EC	Large T antigen
Epicatechin	EXP	5.9	---	---	---	5.9	---	Q8I2J3	EC, PS	M18 aspartyl aminopeptidase

Table S2. *Cont.*

MOLID	AN	pACT	p*Ki*	p*Kd*	p*Kb*	pIC50	pEC50	Uniprot	Functional	TARGET_NAME
Epicatechin	PRD	5.6	---	---	---	5.6	---	P10721	AN, CR, EC, EL, KC	Mast/stem cell growth factor receptor
Epicatechin	EXP	5.4	---	---	---	---	5.4	P0A7G6	DR	Protein RecA
Epicatechin	PRD	6.4	---	---	---	6.4	---	P12931	EC, KC	Proto-oncogene tyrosine-protein kinase Src
Epicatechin	PRD	6	---	---	---	6	---	P04626	CR, EC, EL, KC	Receptor tyrosine-protein kinase erbB-2
Epicatechin	PRD	5.5	---	---	---	5.5	---	P17948	CR, EC, EL, KC	Vascular endothelial growth factor receptor 1
Epicatechin	PRD	5.7	---	---	---	5.7	---	P35968	AN, CR, EC, EL, KC	Vascular endothelial growth factor receptor 2
(*E*)-4-*O*-β-D-Glucopyranosyl caffeic acid	PRD	A	---	---	---	---	---	P01556	BT	Cholera enterotoxin subunit B
(*E*)-4-*O*-β-D-Glucopyranosyl caffeic acid	PRD	A	---	---	---	---	---	P07464	EC	Galactoside *O*-acetyltransferase
(*E*)-4-*O*-β-D-Glucopyranosyl caffeic acid	PRD	A	---	---	---	---	---	P32890	UC	Heat-labile enterotoxin B chain
(*E*)-4-*O*-β-D-Glucopyranosyl caffeic acid	PRD	A	---	---	---	---	---	Q6UWM7	UC	Lactase-like protein
(*E*)-4-*O*-β-D-Glucopyranosyl caffeic acid	PRD	A	---	---	---	---	---	Q700S9	EC	Probable β-galactosidase A
(*E*)-4-*O*-β-D-Glucopyranosyl caffeic acid	PRD	5.5	---	---	---	5.2	5.1	P13866	TC	Sodium/glucose cotransporter 1
(*E*)-4-*O*-β-D-Glucopyranosyl caffeic acid	PRD	7.1	8.6	---	---	7.1	6.9	P31639	UC	Sodium/glucose cotransporter 2
(*E*)-4-*O*-β-D-Glucopyranosyl sinapoic acid	PRD	A	---	---	---	---	---	P01556	BT	Cholera enterotoxin subunit B
(*E*)-4-*O*-β-D-Glucopyranosyl sinapoic acid	PRD	A	---	---	---	---	---	P07464	EC	Galactoside *O*-acetyltransferase
(*E*)-4-*O*-β-D-Glucopyranosyl sinapoic acid	PRD	A	---	---	---	---	---	P32890	UC	Heat-labile enterotoxin B chain
(*E*)-4-*O*-β-D-Glucopyranosyl sinapoic acid	PRD	A	---	---	---	---	---	Q6UWM7	UC	Lactase-like protein
(*E*)-4-*O*-β-D-Glucopyranosyl sinapoic acid	PRD	A	---	---	---	---	---	Q700S9	EC	Probable β-galactosidase A
4-*O*-β-D-Glucopyranosyl ferulic acid	PRD	A	---	---	---	---	---	P01556	BT	Cholera enterotoxin subunit B
4-*O*-β-D-Glucopyranosyl ferulic acid	PRD	A	---	---	---	---	---	P07464	EC	Galactoside *O*-acetyltransferase
4-*O*-β-D-Glucopyranosyl ferulic acid	PRD	A	---	---	---	---	---	P32890	UC	Heat-labile enterotoxin B chain
4-*O*-β-D-Glucopyranosyl ferulic acid	PRD	A	---	---	---	---	---	Q6UWM7	UC	Lactase-like protein
4-*O*-β-D-Glucopyranosyl ferulic acid	PRD	A	---	---	---	---	---	Q700S9	EC	Probable β-galactosidase A
Trans-caffeic acid	EXP	5.6	5.6	---	---	---	---	P00915	EC	Carbonic anhydrase 1
Trans-caffeic acid	EXP	5	5	---	---	---	---	O43570	EC	Carbonic anhydrase 12
Trans-caffeic acid	EXP	5.1	5.1	---	---	---	---	Q9ULX7	EC	Carbonic anhydrase 14
Trans-caffeic acid	EXP	5.8	5.8	---	---	---	---	P00918	EC	Carbonic anhydrase 2

Table S2. *Cont.*

MOLID	AN	pACT	p*Ki*	p*Kd*	p*Kb*	pIC50	pEC50	Uniprot	Functional	TARGET_NAME
Trans-caffeic acid	EXP	5	5	---	---	---	---	P07451	EC	Carbonic anhydrase 3
Trans-caffeic acid	EXP	5	5	---	---	---	---	P22748	EC	Carbonic anhydrase 4
Trans-caffeic acid	EXP	5.2	5.2	---	---	---	---	P35218	EC	Carbonic anhydrase 5A, mitochondrial
Trans-caffeic acid	EXP	5	5	---	---	---	---	Q9Y2D0	EC	Carbonic anhydrase 5B, mitochondrial
Trans-caffeic acid	EXP	5.1	5.1	---	---	---	---	P23280	EC	Carbonic anhydrase 6
Trans-caffeic acid	EXP	5.2	5.2	---	---	---	---	P43166	EC	Carbonic anhydrase 7
Trans-caffeic acid	EXP	5.1	5.1	---	---	---	---	Q16790	EC	Carbonic anhydrase 9
Trans-caffeic acid	PRD	A	---	---	---	---	---	P17538	EC, PS	Chymotrypsinogen B
Trans-caffeic acid	EXP	A	---	---	---	---	---	P42357	EC	Histidine ammonia-lyase
Trans-caffeic acid	EXP	5.1	---	---	---	5.1	---	P03070	EC	Large T antigen
Trans-caffeic acid	EXP	5.5	---	---	---	5.5	---	Q06327	EC	Linoleate 9S-lipoxygenase 1
Trans-caffeic acid	EXP	A	---	---	---	---	---	P14174	EC	Macrophage migration inhibitory factor
Trans-caffeic acid	EXP	A	---	---	---	---	---	P16113	UC	Photoactive yellow protein
Trans-caffeic acid	PRD	A	---	---	---	---	---	Q9X2W8	UC	PPH
Trans-caffeic acid	PRD	A	---	---	---	---	---	Q14914	EC	Prostaglandin reductase 1
Trans-caffeic acid	EXP	5.5	---	---	---	5.5	---	P18031	EC	Tyrosine-protein phosphatase non-receptor type 1
4-*O*-β-D-Glucopyranosyl coumaric acid	PRD	A	---	---	---	---	---	P01556	BT	Cholera enterotoxin subunit B
4-*O*-β-D-Glucopyranosyl coumaric acid	PRD	A	---	---	---	---	---	P07464	EC	Galactoside *O*-acetyltransferase
4-*O*-β-D-Glucopyranosyl coumaric acid	PRD	A	---	---	---	---	---	P32890	UC	Heat-labile enterotoxin B chain
4-*O*-β-D-Glucopyranosyl coumaric acid	PRD	A	---	---	---	---	---	Q6UWM7	UC	Lactase-like protein
4-*O*-β-D-Glucopyranosyl coumaric acid	PRD	A	---	---	---	---	---	Q700S9	EC	Probable β-galactosidase A
4-*O*-β-D-Glucopyranosyl coumaric acid	PRD	6.6	---	---	---	6.6	6.6	P31639	UC	Sodium/glucose cotransporter 2
Dihydrocaffeic acid methyl ester	PRD	5	---	---	---	5	---	P10696	EC	Alkaline phosphatase, placental-like
Dihydrocaffeic acid	PRD	A	---	---	---	---	---	Q44048	EC	3,4-Dihydroxyphenylacetate 2,3-dioxygenase
Dihydrocaffeic acid	PRD	A	---	---	---	---	---	P21397	EC	Amine oxidase [flavin-containing] A
Dihydrocaffeic acid	PRD	A	---	---	---	---	---	P27338	EC	Amine oxidase [flavin-containing] B
Dihydrocaffeic acid	PRD	A	---	---	---	---	---	P20711	EC	Aromatic-L-amino-acid decarboxylase
Dihydrocaffeic acid	PRD	A	---	---	---	---	---	P10635	CP, EC	Cytochrome P450 2D6
Dihydrocaffeic acid	PRD	A	---	---	---	---	---	P21918	GR	D_{1B} dopamine receptor
Dihydrocaffeic acid	PRD	A	---	---	---	---	---	P35462	GR	D_3 dopamine receptor
Dihydrocaffeic acid	PRD	A	---	---	---	---	---	Q45135	UC	Homoprotocatechuate 2,3-dioxygenase
Dihydrocaffeic acid	PRD	5.4	---	---	---	5.4	---	P09923	EC	Intestinal-type alkaline phosphatase
Dihydrocaffeic acid	PRD	A	---	---	---	---	---	Q8TF71	TC	Monocarboxylate transporter 10
Dihydrocaffeic acid	PRD	A	---	---	---	---	---	P06875	EC	Penicillin G acylase
Dihydrocaffeic acid	PRD	A	---	---	---	---	---	O67636	EC	Prephenate dehydrogenase

Table S2. *Cont.*

MOLID	AN	pACT	p*Ki*	p*Kd*	p*Kb*	pIC50	pEC50	Uniprot	Functional	TARGET_NAME
Dihydrocaffeic acid	PRD	A	---	---	---	---	---	P00436	EC	Protocatechuate 3,4-dioxygenase α chain
Dihydrocaffeic acid	PRD	A	---	---	---	---	---	P00437	EC	Protocatechuate 3,4-dioxygenase β chain
Dihydrocaffeic acid	PRD	A	---	---	---	---	---	P46059	UC	Solute carrier family 15 member 1
Dihydrocaffeic acid	PRD	5.1	---	---	---	5.1	---	P14679	EC	Tyrosinase
3,4-Dihydroxyl benzoic acid	PRD	A	---	---	---	---	---	P40871	EC	2,3-Dihydroxybenzoate-AMP ligase
3,4-Dihydroxyl benzoic acid	PRD	A	---	---	---	---	---	P26281	EC	2-Amino-4-hydroxy-6-hydroxymethyldihydropteridine pyrophosphokinase
3,4-Dihydroxyl benzoic acid	PRD	A	---	---	---	---	---	P46952	EC	3-Hydroxyanthranilate 3,4-dioxygenase
3,4-Dihydroxyl benzoic acid	PRD	A	---	---	---	---	---	Q04416	EC	4-Hydroxybenzoyl-CoA thioesterase
3,4-Dihydroxyl benzoic acid	PRD	A	---	---	---	---	---	P19961	EC	α-Amylase 2B
3,4-Dihydroxyl benzoic acid	PRD	A	---	---	---	---	---	P09917	EC	Arachidonate 5-lipoxygenase
3,4-Dihydroxyl benzoic acid	PRD	5.2	5.2	---	---	---	---	O43570	EC	Carbonic anhydrase 12
3,4-Dihydroxyl benzoic acid	PRD	5	5	---	---	---	---	Q99N23	EC	Carbonic anhydrase 15
3,4-Dihydroxyl benzoic acid	PRD	5.4	5.4	---	---	---	---	P35218	EC	Carbonic anhydrase 5A, mitochondrial
3,4-Dihydroxyl benzoic acid	PRD	5	5	---	---	---	---	Q9Y2D0	EC	Carbonic anhydrase 5B, mitochondrial
3,4-Dihydroxyl benzoic acid	PRD	5.1	5.1	---	---	---	---	P23280	EC	Carbonic anhydrase 6
3,4-Dihydroxyl benzoic acid	PRD	A	---	---	---	---	---	P26602	EC	Chorismate-pyruvate lyase
3,4-Dihydroxyl benzoic acid	PRD	5.2	---	5.2	---	---	---	Q81VW8	EC	Dihydropteroate synthase
3,4-Dihydroxyl benzoic acid	PRD	A	---	---	---	---	---	P23893	AA, EC	Glutamate-1-semialdehyde 2,1-aminomutase
3,4-Dihydroxyl benzoic acid	PRD	A	---	---	---	---	---	Q9NZK7	EC	Group IIE secretory phospholipase A2
3,4-Dihydroxyl benzoic acid	PRD	5.8	---	---	---	5.8	---	Q07820	UC	Induced myeloid leukemia cell differentiation protein Mcl-1
3,4-Dihydroxyl benzoic acid	PRD	A	---	---	---	---	---	O15111	EC, KC	Inhibitor of nuclear factor κ-B kinase subunit α
3,4-Dihydroxyl benzoic acid	PRD	A	---	---	---	---	---	O14920	EC, KC	Inhibitor of nuclear factor κ-B kinase subunit β
3,4-Dihydroxyl benzoic acid	EXP	6.3	---	---	---	6.3	---	Q8I2J3	EC, PS	M18 aspartyl aminopeptidase
3,4-Dihydroxyl benzoic acid	PRD	5.4	---	---	---	---	5.4	Q12851	EC, KC	Mitogen-activated protein kinase kinase kinase kinase 2
3,4-Dihydroxyl benzoic acid	PRD	A	---	---	---	---	---	P08183	AN, EC, TC	Multidrug resistance protein 1
3,4-Dihydroxyl benzoic acid	PRD	8.1	---	8.1	---	---	---	P80188	UC	Neutrophil gelatinase-associated lipocalin
3,4-Dihydroxyl benzoic acid	PRD	A	---	---	---	---	---	P04181	AA, EC	Ornithine aminotransferase, mitochondrial
3,4-Dihydroxyl benzoic acid	PRD	A	---	---	---	---	---	P37231	NR, TR	Peroxisome proliferator-activated receptor γ
3,4-Dihydroxyl benzoic acid	PRD	5.8	---	5.8	---	---	---	Q51792	EC	Phenazine biosynthesis protein phzF
3,4-Dihydroxyl benzoic acid	EXP	5.3	3.3	7.3	---	---	---	P20586	EC	P-hydroxybenzoate hydroxylase
3,4-Dihydroxyl benzoic acid	PRD	5.2	---	5.2	---	---	---	Q9Y6F1	DL, DR, EC	Poly [ADP-ribose] polymerase 3
3,4-Dihydroxyl benzoic acid	PRD	5.2	---	---	---	---	5.2	P0A7G6	DR	Protein RecA
3,4-Dihydroxyl benzoic acid	PRD	A	---	---	---	---	---	P50225	EC	Sulfotransferase 1A1

Table S2. *Cont.*

MOLID	AN	pACT	p*Ki*	p*Kd*	p*Kb*	pIC50	pEC50	Uniprot	Functional	TARGET_NAME
4-*O*-β-D-Glucosyl vanillic acid	PRD	A	---	---	---	---	---	Q46829	EC	6-Phospho-β-glucosidase BglA
4-*O*-β-D-Glucosyl vanillic acid	PRD	A	---	---	---	---	---	Q9XBQ3	EC	α-*N*-arabinofuranosidase
4-*O*-β-D-glucosyl vanillic acid	PRD	A	---	---	---	---	---	Q9RIK9	EC	β-mannosidase
4-*O*-β-D-glucosyl vanillic acid	PRD	A	---	---	---	---	---	P01556	BT	Cholera enterotoxin subunit B
4-*O*-β-D-glucosyl vanillic acid	PRD	A	---	---	---	---	---	P07464	EC	Galactoside *O*-acetyltransferase
4-*O*-β-D-glucosyl vanillic acid	PRD	A	---	---	---	---	---	P32890	UC	Heat-labile enterotoxin B chain
4-*O*-β-D-glucosyl vanillic acid	PRD	A	---	---	---	---	---	Q6UWM7	UC	Lactase-like protein
4-*O*-β-D-glucosyl vanillic acid	PRD	A	---	---	---	---	---	P03023	UC	Lactose operon repressor
4-*O*-β-D-glucosyl vanillic acid	PRD	A	---	---	---	---	---	Q700S9	EC	Probable β-galactosidase A
4-*O*-β-D-glucosyl vanillic acid	PRD	6.2	---	---	---	6.2	6.2	P31639	UC	Sodium/glucose cotransporter 2
3-(Acetylamino)-4-hydroxy-benzoic acid	PRD	A	---	---	---	---	---	P03472	EC	Neuraminidase
β-Sitosterol	PRD	9.1	---	---	---	9.1	---	P18405	EC	3-Oxo-5-α-steroid 4-dehydrogenase 1
β-Sitosterol	PRD	6.8	---	---	---	6.8	---	P31213	EC	3-Oxo-5-α-steroid 4-dehydrogenase 2
β-Sitosterol	PRD	7.8	7.8	---	---	---	---	Q9UBM7	EC	7-Dehydrocholesterol reductase
β-Sitosterol	PRD	5.4	---	---	---	5.4	---	P10275	NR, TR	Androgen receptor
β-Sitosterol	PRD	A	---	---	---	---	---	Q9UNQ0	AN, TC	ATP-binding cassette sub-family G member 2
β-Sitosterol	PRD	5.2	---	---	---	---	5.2	Q96RI1	NR, TR	Bile acid receptor
β-Sitosterol	PRD	5.4	5.4	---	---	5.4	---	P06276	EC	Cholinesterase
β-Sitosterol	PRD	6.5	5.7	---	---	6.8	---	P11511	CP, EC	Cytochrome P450 19A1
β-Sitosterol	PRD	A	---	---	---	---	---	P08183	AN, EC, TC	Multidrug resistance protein 1
β-Sitosterol	PRD	A	---	---	---	---	---	P35398	NR, TR	Nuclear receptor ROR-α
β-Sitosterol	PRD	5.2	---	---	---	5.1	5.2	Q13133	NR, TR	Oxysterols receptor LXR-α
β-Sitosterol	PRD	5	---	---	---	5	5	P55055	NR, TR	Oxysterols receptor LXR-β
Maltol glucoside	PRD	6.3	6.2	---	---	6.4	---	P22303	EC	Acetylcholinesterase
Maltol glucoside	PRD	A	---	---	---	---	---	P32890	UC	Heat-labile enterotoxin B chain
1,2-Benzenedicarboxylic acid diisobutyl ester	PRD	7.4	---	---	---	7.4	---	P48147	EC, PS	Prolyl endopeptidase
1,2,3,4,6-Penta-*O*-galloyl-β-D-glucose	PRD	6.1	---	---	---	6.1	---	P26663	EC	Genome polyprotein
1,2,3,4,6-Tetra-*O*-galloyl-β-D-glucose	PRD	6.2	6.1	---	---	6.3	---	P00742	CL, EC, PS	Coagulation factor X
1,2,3,4,6-Tetra-*O*-galloyl-β-D-glucose	PRD	6.1	---	---	---	6.1	---	P26663	EC	Genome polyprotein
1,2,3,4,6-Tetra-*O*-galloyl-β-D-glucose	PRD	6.8	6.7	---	---	7.1	---	P00734	CL, EC, PS	Prothrombin
1,2,3,4,6-Tetra-*O*-galloyl-β-D-glucose	PRD	5.8	---	---	---	5.8	---	Q14534	EC	Squalene monooxygenase
2,3,4,6-Tetra-*O*-galloyl-α-D-glucose	PRD	6.1	6	---	---	6.3	---	P00742	CL,EC,PS	Coagulation factor X
2,3,4,6-Tetra-*O*-galloyl-α-D-glucose	PRD	6.1	---	---	---	6.1	---	P26663	EC	Genome polyprotein
2,3,4,6-Tetra-*O*-galloyl-α-D-glucose	PRD	6.8	7.3	---	---	6.8	---	P00734	CL, EC, PS	Prothrombin
2,3,4,6-Tetra-*O*-galloyl-α-D-glucose	PRD	5.7	---	---	---	5.7	---	Q14534	EC	Squalene monooxygenase

Table S2. *Cont.*

MOLID	AN	pACT	p*K*i	p*K*d	p*K*b	pIC50	pEC50	Uniprot	Functional	TARGET_NAME
Kaempferol 3-*O*-α-L-rhamnoside-7-*O*-β-D-glucopyranoside	PRD	A	---	---	---	---	---	P42330	EC	Aldo-keto reductase family 1 member C3
Kaempferol 3-*O*-α-L-rhamnoside-7-*O*-β-D-glucopyranoside	PRD	8.1	8.1	---	---	---	---	P08913	GR	α-2A adrenergic receptor
Kaempferol 3-*O*-α-L-rhamnoside-7-*O*-β-D-glucopyranoside	PRD	7.3	7.3	---	---	---	---	P18825	GR	α-2C adrenergic receptor
Kaempferol 3-*O*-α-L-rhamnoside-7-*O*-β-D-glucopyranoside	PRD	5.2	---	---	---	5.2	---	O76074	EC	cGMP-specific 3′,5′-cyclic phosphodiesterase
Kaempferol 3-*O*-α-L-rhamnoside-7-*O*-β-D-glucopyranoside	PRD	A	---	---	---	---	---	P10632	CP, EC	Cytochrome P450 2C8
Kaempferol 3-*O*-α-L-rhamnoside-7-*O*-β-D-glucopyranoside	PRD	A	---	---	---	---	---	P11712	CP, EC	Cytochrome P450 2C9
Kaempferol 3-*O*-α-L-rhamnoside-7-*O*-β-D-glucopyranoside	PRD	A	---	---	---	---	---	P10635	CP, EC	Cytochrome P450 2D6
Kaempferol 3-*O*-α-L-rhamnoside-7-*O*-β-D-glucopyranoside	PRD	7.5	7.5	---	---	---	---	P08912	GR	Muscarinic acetylcholine receptor M5
Kaempferol 3-*O*-α-L-rhamnoside-7-*O*-β-D-glucopyranoside	PRD	A	---	---	---	---	---	Q9GZQ4	GR	Neuromedin-U receptor 2
5-ethoxy-3-hydroxy-benzoate	PRD	5.1	---	---	---	---	5.1	P35869	TR	Aryl hydrocarbon receptor
Kaempferol-3-*O*-α-L-rhamnopyranoside	PRD	5.7	---	---	---	5.7	---	P15121	EC	Aldose reductase
3-acetamino-4-hydroxy-benzoic acid	PRD	A	---	---	---	---	---	P03472	EC	Neuraminidase
Protocatechuic acid	PRD	A	---	---	---	---	---	P40871	EC	2,3-Dihydroxybenzoate-AMP ligase
Protocatechuic acid	PRD	A	---	---	---	---	---	P26281	EC	2-Amino-4-hydroxy-6-hydroxymethyldihydropteridine pyrophosphokinase
Protocatechuic acid	PRD	A	---	---	---	---	---	P46952	EC	3-Hydroxyanthranilate 3,4-dioxygenase
Protocatechuic acid	PRD	A	---	---	---	---	---	Q04416	EC	4-Hydroxybenzoyl-CoA thioesterase

Table S2. *Cont.*

MOLID	AN	pACT	p*K*i	p*K*d	p*K*b	pIC50	pEC50	Uniprot	Functional	TARGET_NAME
Protocatechuic acid	PRD	A	---	---	---	---	---	P19961	EC	α-Amylase 2B
Protocatechuic acid	PRD	A	---	---	---	---	---	P09917	EC	Arachidonate 5-lipoxygenase
Protocatechuic acid	PRD	5.2	5.2	---	---	---	---	O43570	EC	Carbonic anhydrase 12
Protocatechuic acid	PRD	5	5	---	---	---	---	Q99N23	EC	Carbonic anhydrase 15
Protocatechuic acid	PRD	5.4	5.4	---	---	---	---	P35218	EC	Carbonic anhydrase 5A, mitochondrial
Protocatechuic acid	PRD	5	5	---	---	---	---	Q9Y2D0	EC	Carbonic anhydrase 5B, mitochondrial
Protocatechuic acid	PRD	5.1	5.1	---	---	---	---	P23280	EC	Carbonic anhydrase 6
Protocatechuic acid	PRD	A	---	---	---	---	---	P26602	EC	Chorismate-pyruvate lyase
Protocatechuic acid	PRD	5.2	---	5.2	---	---	---	Q81VW8	EC	Dihydropteroate synthase
Protocatechuic acid	PRD	A	---	---	---	---	---	P23893	AA, EC	Glutamate-1-semialdehyde 2,1-aminomutase
Protocatechuic acid	PRD	A	---	---	---	---	---	Q9NZK7	EC	Group IIE secretory phospholipase A2
Protocatechuic acid	PRD	5.8	---	---	---	5.8	---	Q07820	UC	Induced myeloid leukemia cell differentiation protein Mcl-1
Protocatechuic acid	PRD	A	---	---	---	---	---	O15111	EC, KC	Inhibitor of nuclear factor κ-B kinase subunit α
Protocatechuic acid	PRD	A	---	---	---	---	---	O14920	EC, KC	Inhibitor of nuclear factor κ-B kinase subunit β
Protocatechuic acid	EXP	6.3	---	---	---	6.3	---	Q8I2J3	EC, PS	M18 aspartyl aminopeptidase
Protocatechuic acid	PRD	5.4	---	---	---	---	5.4	Q12851	EC, KC	Mitogen-activated protein kinase kinase kinase kinase 2
Protocatechuic acid	PRD	A	---	---	---	---	---	P08183	AN, EC, TC	Multidrug resistance protein 1
Protocatechuic acid	PRD	8.1	---	8.1	---	---	---	P80188	UC	Neutrophil gelatinase-associated lipocalin
Protocatechuic acid	PRD	A	---	---	---	---	---	P04181	AA, EC	Ornithine aminotransferase, mitochondrial
Protocatechuic acid	PRD	A	---	---	---	---	---	P37231	NR, TR	Peroxisome proliferator-activated receptor γ
Protocatechuic acid	PRD	5.8	---	5.8	---	---	---	Q51792	EC	Phenazine biosynthesis protein phzF
Protocatechuic acid	EXP	5.3	3.3	7.3	---	---	---	P20586	EC	P-hydroxybenzoate hydroxylase
Protocatechuic acid	PRD	5.2	---	5.2	---	---	---	Q9Y6F1	DL, DR, EC	Poly [ADP-ribose] polymerase 3
Protocatechuic acid	PRD	5.2	---	---	---	---	5.2	P0A7G6	DR	Protein RecA
Protocatechuic acid	PRD	A	---	---	---	---	---	P50225	EC	Sulfotransferase 1A1
Prunin	PRD	A	---	---	---	---	---	P05091	EC	Aldehyde dehydrogenase, mitochondrial
Prunin	PRD	5.1	---	---	---	5.1	---	P15121	EC	Aldose reductase
Prunin	PRD	5.6	---	---	---	5.6	---	P10696	EC	Alkaline phosphatase, placental-like
Prunin	PRD	A	---	---	---	---	---	Q9UNQ0	AN, TC	ATP-binding cassette sub-family G member 2
Prunin	PRD	5.3	4.9	---	---	5.6	---	P14416	GR	D_2 dopamine receptor
Prunin	PRD	5.1	5.9	---	---	4.3	---	P21917	GR	D_2 dopamine receptor
Prunin	PRD	5.2	5.2	---	---	4.7	---	P03372	NR, TR	Estrogen receptor
Prunin	PRD	5.8	---	---	---	5.8	---	P05113	CY	Interleukin-5
Prunin	PRD	5.9	---	---	---	5.9	---	P09923	EC	Intestinal-type alkaline phosphatase

Table S2. *Cont.*

MOLID	AN	pACT	p*K*i	p*K*d	p*K*b	pIC50	pEC50	Uniprot	Functional	TARGET_NAME
Prunin	PRD	5.6	5.6	---	---	6.6	6.5	P13866	TC	Sodium/glucose cotransporter 1
Prunin	PRD	7.1	7.2	---	---	7.1	7.7	P31639	UC	Sodium/glucose cotransporter 2
Kaempferol-3-*O*-α-L-rhamnoside-7-*O*-β-D-glucoside	PRD	5.2	---	---	---	5.2	---	O76074	EC	cGMP-specific 3′,5′-cyclic phosphodiesterase
Kaempferol-3-*O*-α-L-rhamnoside -7-*O*-β-D-glucoside	PRD	5.3	---	---	---	5.3	---	P11511	CP, EC	Cytochrome P450 19A1
Kaempferol-3-*O*-α-L-rhamnoside-7-*O*-β-D-glucoside	PRD	5.1	---	---	---	5.1	---	P61088	BQ, DR, EC	Ubiquitin-conjugating enzyme E2 N
Aureusidin-6-*O*-neohesperidoside	PRD	A	---	---	---	---	---	P42330	EC	Aldo-keto reductase family 1 member C3
Aureusidin-6-*O*-neohesperidoside	PRD	8.1	8.1	---	---	---	---	P08913	GR	α-2A adrenergic receptor
Aureusidin-6-*O*-neohesperidoside	PRD	7.3	7.3	---	---	---	---	P18825	GR	α-2C adrenergic receptor
Aureusidin-6-*O*-neohesperidoside	PRD	5.3	---	---	---	5.3	---	P11511	CP, EC	Cytochrome P450 19A1
Aureusidin-6-*O*-neohesperidoside	PRD	A	---	---	---	---	---	P10632	CP, EC	Cytochrome P450 2C8
Aureusidin-6-*O*-neohesperidoside	PRD	A	---	---	---	---	---	P11712	CP, EC	Cytochrome P450 2C9
Aureusidin-6-*O*-neohesperidoside	PRD	A	---	---	---	---	---	P10635	CP, EC	Cytochrome P450 2D6
Aureusidin-6-*O*-neohesperidoside	PRD	7.4	7.4	---	---	---	---	P08912	GR	Muscarinic acetylcholine receptor M5
Aureusidin-6-*O*-neohesperidoside	PRD	5.1	---	---	---	5.1	---	P61088	BQ, DR, EC	Ubiquitin-conjugating enzyme E2 N

Table S3. Information of all identified molecule Nighbours retured form PredictFX 1.1. Molid: Compound identifier; Uniprot: Protein UNIPROT code; SIM: Similarity(or identity) to the reference compound; sCOLL: identity to the reference compound; REF_NN: Reference compound identifier(InChIKey generated with OpenBabel v2.2.+ which includes the1.02 version of the inchi library); REF_PACT: Reference compound pACT value; A: active; SOURCE_DB: Orignal Database(s) reproting this REF_NN-protein annotation.

Molid	Uniprot	SIM	REF_NN	REF_pACT	SOURCE_DB
Naringin	P61088	sCOLL	DFPMSGMNTNDNHN-ZFOFJSCHSA-N	5.08	PubChem
Naringin	P61088	sCOLL	DFPMSGMNTNDNHN-ZHNJBYEHSA-N	5.12	PubChem
Naringin	P11712	0.815	IKGXIBQEEMLURG-BKUODXTLSA-N	A	DrugBank
Naringin	P10632	0.815	IKGXIBQEEMLURG-BKUODXTLSA-N	A	DrugBank
Naringin	P10635	0.815	IKGXIBQEEMLURG-BKUODXTLSA-N	A	DrugBank
Naringin	P11511	sCOLL	DFPMSGMNTNDNHN-ZHNJBYEHSA-N	5.3	PubChem
Naringin	P11511	sCOLL	DFPMSGMNTNDNHN-ZPHOTFPESA-N	5.3	BindingDB, ChemblDB
Naringin	P11511	0.971	SXNOCVMJOZRSLS-MCEICCLHSA-N	5.3	BindingDB
Naringin	P42330	0.815	IKGXIBQEEMLURG-BKUODXTLSA-N	A	DrugBank
Naringin	P08912	0.815	IKGXIBQEEMLURG-UHFFFAOYSA-N	7.45	PDSP
Naringin	P08913	0.815	IKGXIBQEEMLURG-NVPNHPEKSA-N	8.05	BindingDB, PubChem, ChemblDB
Naringin	P08913	0.815	IKGXIBQEEMLURG-UHFFFAOYSA-N	8.05	PDSP
Naringin	P18825	0.815	IKGXIBQEEMLURG-NVPNHPEKSA-N	8.05	BindingDB, ChemblDB, PubChem
Naringin	P18825	0.815	IKGXIBQEEMLURG-UHFFFAOYSA-N	6.54	PDSP
Naringin	Q02410	0.91	QUQPHWDTPGMPEX-QJBIFVCTSA-N	5.02	PubChem
5,7,3′,5′-Tetrahydroxy-flavanone 7-*O*-neohesperidoside	P61088	0.972	DFPMSGMNTNDNHN-ZFOFJSCHSA-N	5.08	PubChem
5,7,3′,5′-Tetrahydroxy-flavanone 7-*O*-neohesperidoside	P61088	0.972	DFPMSGMNTNDNHN-ZHNJBYEHSA-N	5.12	PubChem
5,7,3′,5′-Tetrahydroxy-flavanone 7-*O*-neohesperidoside	P11712	0.858	IKGXIBQEEMLURG-BKUODXTLSA-N	A	DrugBank
5,7,3′,5′-Tetrahydroxy-flavanone 7-*O*-neohesperidoside	P10632	0.858	IKGXIBQEEMLURG-BKUODXTLSA-N	A	DrugBank
5,7,3′,5′-Tetrahydroxy-flavanone 7-*O*-neohesperidoside	P10635	0.858	IKGXIBQEEMLURG-BKUODXTLSA-N	A	DrugBank
5,7,3′,5′-Tetrahydroxy-flavanone 7-*O*-neohesperidoside	P11511	0.972	DFPMSGMNTNDNHN-ZHNJBYEHSA-N	5.3	PubChem
5,7,3′,5′-Tetrahydroxy-flavanone 7-*O*-neohesperidoside	P11511	0.972	DFPMSGMNTNDNHN-ZPHOTFPESA-N	5.3	BindingDB, ChemblDB
5,7,3′,5′-Tetrahydroxy-flavanone 7-*O*-neohesperidoside	P11511	0.945	SXNOCVMJOZRSLS-MCEICCLHSA-N	5.3	BindingDB

Table S3. *Cont.*

Molid	Uniprot	SIM	REF_NN	REF_pACT	SOURCE_DB
5,7,3′,5′-Tetrahydroxy-flavanone 7-*O*-neohesperidoside	P42330	0.858	IKGXIBQEEMLURG-BKUODXTLSA-N	A	DrugBank
5,7,3′,5′-Tetrahydroxy-flavanone 7-*O*-neohesperidoside	P08912	0.858	IKGXIBQEEMLURG-UHFFFAOYSA-N	7.45	PDSP
5,7,3′,5′-Tetrahydroxy-flavanone 7-*O*-neohesperidoside	P08913	0.858	IKGXIBQEEMLURG-NVPNHPEKSA-N	8.05	BindingDB, PubChem, ChemblDB
5,7,3′,5′-Tetrahydroxy-flavanone 7-*O*-neohesperidoside	P08913	0.858	IKGXIBQEEMLURG-UHFFFAOYSA-N	8.05	PDSP
5,7,3′,5′-Tetrahydroxy-flavanone 7-*O*-neohesperidoside	P18825	0.858	IKGXIBQEEMLURG-NVPNHPEKSA-N	8.05	BindingDB, ChemblDB, PubChem
5,7,3′,5′-Tetrahydroxy-flavanone 7-*O*-neohesperidoside	P18825	0.858	IKGXIBQEEMLURG-UHFFFAOYSA-N	6.54	PDSP
5,7,3′,5′-Tetrahydroxy-flavanone 7-*O*-neohesperidoside	Q02410	0.891	QUQPHWDTPGMPEX-QJBIFVCTSA-N	5.02	PubChem
Narigenin-7-O-β-D-glucoside	Q9UNQ0	0.848	KYQZWONCHDNPDP-QNDFHXLGSA-N	A	DrugBank
Narigenin-7-O-β-D-glucoside	P05113	0.837	ISQRJFLLIDGZEP-CMWLGVBASA-N	5.85	BindingDB, PubChem
Narigenin-7-O-β-D-glucoside	P15121	0.789	QSLQKIQXZKDLIH-GKARDXTASA-N	4.68	ChemblDB
Narigenin-7-O-β-D-glucoside	P15121	0.779	TXKFRRCKZWJXBW-GPRNFGOXSA-N	5.5	BindingDB, ChemblDB, PubChem
Narigenin-7-O-β-D-glucoside	P15121	0.777	GLTCTFBPNQJRQT-PBTMSNHXSA-N	5.09	ChemblDB
Narigenin-7-O-β-D-glucoside	P05091	0.848	KYQZWONCHDNPDP-QNDFHXLGSA-N	A	DrugBank
Narigenin-7-O-β-D-glucoside	P09923	0.769	AEDDIBAIWPIIBD-UHFFFAOYSA-N	5.92	PubChem
Narigenin-7-O-β-D-glucoside	P10696	0.769	AEDDIBAIWPIIBD-UHFFFAOYSA-N	5.57	PubChem
Narigenin-7-O-β-D-glucoside	P14416	0.793	OEUGQYOMKCJJLJ-UHFFFAOYSA-N	5.28	BindingDB, ChemblDB, PubChem
Narigenin-7-O-β-D-glucoside	P21917	0.793	OEUGQYOMKCJJLJ-LMCMXOCHSA-N	A	hGPCRlig
Narigenin-7-O-β-D-glucoside	P21917	0.793	OEUGQYOMKCJJLJ-UHFFFAOYSA-N	5.12	ChemblDB, BindingDB, PubChem
Narigenin-7-O-β-D-glucoside	P03372	0.819	HSWIRQIYASIOBE-JNHRPPPUSA-N	5.24	ChemblDB, PubChem
Narigenin-7-O-β-D-glucoside	P03372	0.819	HSWIRQIYASIOBE-UHFFFAOYSA-N	5.24	BindingDB
Narigenin-7-O-β-D-glucoside	P13866	0.954	KOTXSQPZNZHNFC-UHFFFAOYSA-N	6.25	BindingDB, ChemblDB, PubChem
Narigenin-7-O-β-D-glucoside	P13866	0.929	GOTAZLUFLPHQJU-UHFFFAOYSA-N	5.55	BindingDB, ChemblDB, PubChem
Narigenin-7-O-β-D-glucoside	P13866	0.901	IOUVKUPGCMBWBT-QNDFHXLGSA-N	6.59	BindingDB, ChemblDB, PubChem
Narigenin-7-O-β-D-glucoside	P13866	0.901	IOUVKUPGCMBWBT-UHFFFAOYSA-N	6.75	BindingDB, ChemblDB, PubChem
Narigenin-7-O-β-D-glucoside	P13866	0.863	IWRUKKIVIXIBRH-DODNOZFWSA-N	5.33	BindingDB, ChemblDB, PubChem
Narigenin-7-O-β-D-glucoside	P13866	0.863	JSFDGGQKOKTSOU-PFKOEMKTSA-N	4.87	BindingDB, ChemblDB, PubChem
Narigenin-7-O-β-D-glucoside	P13866	0.863	RMQQQPQAVFICPZ-PFKOEMKTSA-N	5.8	BindingDB, ChemblDB, PubChem
Narigenin-7-O-β-D-glucoside	P13866	0.858	CLRQMIQNMTYMGA-XIKSMUEASA-N	5.86	BindingDB, ChemblDB, PubChem
Narigenin-7-O-β-D-glucoside	P13866	0.858	GMYFQAHYWIYNES-PFKOEMKTSA-N	6.74	BindingDB, ChemblDB, PubChem

Table S3. *Cont.*

Molid	Uniprot	SIM	REF_NN	REF_pACT	SOURCE_DB
Narigenin-7-O-β-D-glucoside	P13866	0.858	VAVAYLQBNLAMRO-XIKSMUEASA-N	5.36	BindingDB, ChemblDB, PubChem
Narigenin-7-O-β-D-glucoside	P13866	0.855	DKUVOIUBCISXDG-UHFFFAOYSA-N	5.58	BindingDB, ChemblDB, PubChem
Narigenin-7-*O*-β-D-glucoside	P13866	0.847	ACMMSHORHBTOEZ-PRDVQWLOSA-N	5.08	BindingDB, ChemblDB, PubChem
Narigenin-7-*O*-β-D-glucoside	P13866	0.844	IINBYKILNZBSAK-PFKOEMKTSA-N	5.84	BindingDB, ChemblDB, PubChem
Narigenin-7-*O*-β-DD-glucoside	P13866	0.844	KPTNFLTZJSDNHO-PFKOEMKTSA-N	4.79	BindingDB, ChemblDB, PubChem
Narigenin-7-*O*-β-D-glucoside	P13866	0.844	LBKNLPSWXKBZPU-DODNOZFWSA-N	5.43	BindingDB, ChemblDB, PubChem
Narigenin-7-*O*-β-D-glucoside	P13866	0.844	QFUQUZDKFKPDFP-UHFFFAOYSA-N	4.35	BindingDB, ChemblDB, PubChem
Narigenin-7-*O*-β-D-glucoside	P13866	0.843	HZQBMUPOYJUQAR-XDXGNBCUSA-N	5.88	BindingDB, ChemblDB, PubChem
Narigenin-7-*O*-β-D-glucoside	P13866	0.843	QAJZQZIOIFEMTH-PRDVQWLOSA-N	5.32	BindingDB, ChemblDB, PubChem
Narigenin-7-*O*-β-D-glucoside	P13866	0.843	WKPBVEUJSMWTPM-XDXGNBCUSA-N	5	BindingDB ChemblDB, PubChem
Narigenin-7-*O*-β-D-glucoside	P13866	0.843	XVBIDTMBHOOAMG-PRDVQWLOSA-N	5.43	BindingDB, ChemblDB, PubChem
Narigenin-7-*O*-β-D-glucoside	P13866	0.84	WQCWELFQKXIPCN-UTCJRWHESA-N	2.5	BindingDB, ChemblDB, PubChem
Narigenin-7-*O*-β-D-glucoside	P13866	0.834	ZLMFBIABOBCOTJ-UHFFFAOYSA-N	5.42	BindingDB, ChemblDB, PubChem
Narigenin-7-*O*-β-D-glucoside	P13866	0.83	FMFDIEUZQZXVQG-XDXGNBCUSA-N	4.48	BindingDB, ChemblDB, PubChem
Narigenin-7-*O*-β-D-glucoside	P13866	0.83	JVBJTLHQLXTUOY-UIKHAHSZSA-N	5.19	BindingDB, ChemblDB, PubChem
Narigenin-7-*O*-β-D-glucoside	P13866	0.827	NLZYYMHUDULKQF-PRDVQWLOSA-N	5.2	BindingDB, ChemblDB, PubChem
Narigenin-7-*O*-β-D-glucoside	P13866	0.82	RFZGXHLLXXDQJN-UHFFFAOYSA-N	4.64	BindingDB, ChemblDB, PubChem
Narigenin-7-*O*-β-D-glucoside	P13866	0.784	FINNPFQSLRPSQZ-UHFFFAOYSA-N	4.03	BindingDB, ChemblDB, PubChem
Narigenin-7-*O*-β-D-glucoside	P13866	0.779	BJEOSUUCUHNCLB-DODNOZFWSA-N	3.85	BindingDB, ChemblDB, PubChem
Narigenin-7-*O*-β-D-glucoside	P13866	0.779	RTCQWPDTJIXOFG-DODNOZFWSA-N	3.85	BindingDB, ChemblDB, PubChem
Narigenin-7-*O*-β-D-glucoside	P13866	0.774	RKVRUEAVIWWMKJ-PRDVQWLOSA-N	3.85	BindingDB,ChemblDB,PubChem
Narigenin-7-*O*-β-D-glucoside	P31639	0.901	IOUVKUPGCMBWBT-QNDFHXLGSA-N	7.18	BindingDB, ChemblDB, PubChem
Narigenin-7-*O*-β-D-glucoside	P31639	0.863	IWRUKKIVIXIBRH-DODNOZFWSA-N	7.1	BindingDB, ChemblDB, PubChem
Narigenin-7-*O*-β-D-glucoside	P31639	0.863	JSFDGGQKOKTSOU-PFKOEMKTSA-N	5.89	BindingDB, ChemblDB, PubChem
Narigenin-7-*O*-β-D-glucoside	P31639	0.863	RMQQQPQAVFICPZ-PFKOEMKTSA-N	8	BindingDB, ChemblDB, PubChem
Narigenin-7-*O*-β-D-glucoside	P31639	0.858	CLRQMIQNMTYMGA-XIKSMUEASA-N	7.16	BindingDB,ChemblDB, PubChem
Narigenin-7-*O*-β-D-glucoside	P31639	0.858	GMYFQAHYWIYNES-PFKOEMKTSA-N	8.11	BindingDB, ChemblDB, PubChem
Narigenin-7-*O*-β-D-glucoside	P31639	0.858	VAVAYLQBNLAMRO-XIKSMUEASA-N	6.64	BindingDB, ChemblDB, PubChem
Narigenin-7-*O*-β-D-glucoside	P31639	0.847	ACMMSHORHBTOEZ-PRDVQWLOSA-N	7.52	BindingDB, ChemblDB, PubChem
Narigenin-7-*O*-β-D-glucoside	P31639	0.844	IINBYKILNZBSAK-PFKOEMKTSA-N	8.05	BindingDB, ChemblDB, PubChem
Narigenin-7-*O*-β-D-glucoside	P31639	0.844	KPTNFLTZJSDNHO-PFKOEMKTSA-N	6.54	BindingDB, ChemblDB, PubChem
Narigenin-7-*O*-β-D-glucoside	P31639	0.844	LBKNLPSWXKBZPU-DODNOZFWSA-N	7.7	BindingDB, ChemblDB, PubChem
Narigenin-7-*O*-β-D-glucoside	P31639	0.843	HZQBMUPOYJUQAR-XDXGNBCUSA-N	8	BindingDB, ChemblDB, PubChem
Narigenin-7-O-β-D-glucoside	P31639	0.843	QAJZQZIOIFEMTH-PRDVQWLOSA-N	7.52	BindingDB, ChemblDB, PubChem
Narigenin-7-*O*-β-D-glucoside	P31639	0.843	WKPBVEUJSMWTPM-XDXGNBCUSA-N	6.28	BindingDB, ChemblDB, PubChem
Narigenin-7-*O*-β-D-glucoside	P31639	0.843	XVBIDTMBHOOAMG-PRDVQWLOSA-N	7.16	BindingDB, ChemblDB, PubChem

Table S3. *Cont.*

Molid	Uniprot	SIM	REF_NN	REF_pACT	SOURCE_DB
Narigenin-7-*O*-β-D-glucoside	P31639	0.833	JIQDXQSGNNHQOD-DODNOZFWSA-N	5.73	BindingDB, ChemblDB, PubChem
Narigenin-7-*O*-β-D-glucoside	P31639	0.83	FMFDIEUZQZXVQG-XDXGNBCUSA-N	6.96	BindingDB, ChemblDB, PubChem
Narigenin-7-*O*-β-D-glucoside	P31639	0.83	JVBJTLHQLXTUOY-UIKHAHSZSA-N	8	BindingDB, ChemblDB, PubChem
Narigenin-7-*O*-β-D-glucoside	P31639	0.827	NLZYYMHUDULKQF-PRDVQWLOSA-N	8	BindingDB, ChemblDB, PubChem
Narigenin-7-*O*-β-D-glucoside	P31639	0.779	BJEOSUUCUHNCLB-DODNOZFWSA-N	5.27	BindingDB, ChemblDB, PubChem
Narigenin-7-*O*-β-D-glucoside	P31639	0.779	RTCQWPDTJIXOFG-DODNOZFWSA-N	5.27	BindingDB, ChemblDB, PubChem
Narigenin-7-*O*-β-D-glucoside	P31639	0.774	RKVRUEAVIWWMKJ-PRDVQWLOSA-N	5.27	BindingDB, ChemblDB, PubChem
5,7,3′,5′-Tetrahydroxy-flavanone 7-*O*-β-D-glucopyranoside	P05113	0.833	ISQRJFLLIDGZEP-CMWLGVBASA-N	5.85	BindingDB, PubChem
5,7,3′,5′-Tetrahydroxy-flavanone 7-*O*-β-D-glucopyranoside	P15121	0.831	TXKFRRCKZWJXBW-GPRNFGOXSA-N	5.5	BindingDB, ChemblDB, PubChem
5,7,3′,5′-Tetrahydroxy-flavanone 7-*O*-β-D-glucopyranoside	P15121	0.813	JPUKWEQWGBDDQB-QSOFNFLRSA-N	5.29	BindingDB, ChemblDB, PubChem
5,7,3′,5′-Tetrahydroxy-flavanone 7-*O*-β-D-glucopyranoside	P15121	0.775	QSLQKIQXZKDLIH-GKARDXTASA-N	4.68	ChemblDB
5,7,3′,5′-Tetrahydroxy-flavanone 7-*O*-β-D-glucopyranoside	P15121	0.769	GLTCTFBPNQJRQT-PBTMSNHXSA-N	5.09	ChemblDB
5,7,3′,5′-Tetrahydroxy-flavanone 7-*O*-β-D-glucopyranoside	P09923	0.811	AEDDIBAIWPIIBD-UHFFFAOYSA-N	5.92	PubChem
5,7,3′,5′-Tetrahydroxy-flavanone 7-*O*-β-D-glucopyranoside	P10696	0.811	AEDDIBAIWPIIBD-UHFFFAOYSA-N	5.57	PubChem
5,7,3′,5′-Tetrahydroxy-flavanone 7-*O*-β-D-glucopyranoside	P03070	0.853	MYXNWGACZJSMBT-UHFFFAOYSA-N	4.67	PubChem
5,7,3′,5′-Tetrahydroxy-flavanone 7-*O*-β-D-glucopyranoside	P03070	0.844	ODBRNZZJSYPIDI-UHFFFAOYSA-N	5.28	PubChem
5,7,3′,5′-Tetrahydroxy-flavanone 7-*O*-β-D-glucopyranoside	P03070	0.811	AEDDIBAIWPIIBD-UHFFFAOYSA-N	5.06	PubChem
5,7,3′,5′-Tetrahydroxy-flavanone 7-*O*-β-D-glucopyranoside	P03070	0.792	PLAPMLGJVGLZOV-YWFAZRBLSA-N	5.5	PubChem
5,7,3′,5′-Tetrahydroxy-flavanone 7-*O*-β-D-glucopyranoside	P14416	0.793	OEUGQYOMKCJJLJ-UHFFFAOYSA-N	5.28	BindingDB, ChemblDB, PubChem
5,7,3′,5′-Tetrahydroxy-flavanone 7-*O*-β-D-glucopyranoside	P21917	0.793	OEUGQYOMKCJJLJ-LMCMXOCHSA-N	A	hGPCRlig
5,7,3′,5′-Tetrahydroxy-flavanone 7-*O*-β-D-glucopyranoside	P21917	0.793	OEUGQYOMKCJJLJ-UHFFFAOYSA-N	5.12	ChemblDB, BindingDB, PubChem

Table S3. *Cont.*

Molid	Uniprot	SIM	REF_NN	REF_pACT	SOURCE_DB
5,7,3′,5′-Tetrahydroxy-flavanone 7-*O*-β-D-glucopyranoside	P03372	0.792	HSWIRQIYASIOBE-JNHRPPPUSA-N	5.24	ChemblDB, PubChem
5,7,3′,5′-Tetrahydroxy-flavanone 7-*O*-β-D-glucopyranoside	P03372	0.792	HSWIRQIYASIOBE-UHFFFAOYSA-N	5.24	BindingDB
5,7,3′,5′-Tetrahydroxy-flavanone 7-*O*-β-D-glucopyranoside	P13866	0.932	IOUVKUPGCMBWBT-QNDFHXLGSA-N	6.59	BindingDB, ChemblDB, PubChem
5,7,3′,5′-Tetrahydroxy-flavanone 7-*O*-β-D-glucopyranoside	P13866	0.932	IOUVKUPGCMBWBT-UHFFFAOYSA-N	6.75	BindingDB, ChemblDB, PubChem
5,7,3′,5′-Tetrahydroxy-flavanone 7-*O*-β-D-glucopyranoside	P13866	0.906	ZLMFBIABOBCOTJ-UHFFFAOYSA-N	5.42	BindingDB, ChemblDB, PubChem
5,7,3′,5′-Tetrahydroxy-flavanone 7-*O*-β-D-glucopyranoside	P13866	0.886	KOTXSQPZNZHNFC-UHFFFAOYSA-N	6.25	BindingDB, ChemblDB, PubChem
5,7,3′,5′-Tetrahydroxy-flavanone 7-*O*-β-D-glucopyranoside	P13866	0.877	GOTAZLUFLPHQJU-UHFFFAOYSA-N	5.55	BindingDB, ChemblDB, PubChem
5,7,3′,5′-Tetrahydroxy-flavanone 7-*O*-β-D-glucopyranoside	P13866	0.863	WQCWELFQKXIPCN-UTCJRWHESA-N	2.5	BindingDB, ChemblDB, PubChem
5,7,3′,5′-Tetrahydroxy-flavanone 7-*O*-β-D-glucopyranoside	P13866	0.851	DKUVOIUBCISXDG-UHFFFAOYSA-N	5.58	BindingDB, ChemblDB, PubChem
5,7,3′,5′-Tetrahydroxy-flavanone 7-*O*-β-D-glucopyranoside	P13866	0.849	RFZGXHLLXXDQJN-UHFFFAOYSA-N	4.64	BindingDB, ChemblDB, PubChem
5,7,3′,5′-Tetrahydroxy-flavanone 7-*O*-β-D-glucopyranoside	P13866	0.836	QFUQUZDKFKPDFP-UHFFFAOYSA-N	4.35	BindingDB, ChemblDB, PubChem
5,7,3′,5′-Tetrahydroxy-flavanone 7-*O*-β-D-glucopyranoside	P13866	0.825	CLRQMIQNMTYMGA-XIKSMUEASA-N	5.86	BindingDB, ChemblDB, PubChem
5,7,3′,5′-Tetrahydroxy-flavanone 7-*O*-β-D-glucopyranoside	P13866	0.825	VAVAYLQBNLAMRO-XIKSMUEASA-N	5.36	BindingDB, ChemblDB, PubChem
5,7,3′,5′-Tetrahydroxy-flavanone 7-*O*-β-D-glucopyranoside	P13866	0.814	FINNPFQSLRPSQZ-UHFFFAOYSA-N	4.03	BindingDB, ChemblDB, PubChem
5,7,3′,5′-Tetrahydroxy-flavanone 7-*O*-β-D-glucopyranoside	P13866	0.804	ACMMSHORHBTOEZ-PRDVQWLOSA-N	5.08	BindingDB, ChemblDB, PubChem
5,7,3′,5′-Tetrahydroxy-flavanone 7-*O*-β-D-glucopyranoside	P13866	0.804	IINBYKILNZBSAK-PFKOEMKTSA-N	5.84	BindingDB, ChemblDB, PubChem
5,7,3′,5′-Tetrahydroxy-flavanone 7-*O*-β-D-glucopyranoside	P13866	0.804	IWRUKKIVIXIBRH-DODNOZFWSA-N	5.33	BindingDB, ChemblDB, PubChem
5,7,3′,5′-Tetrahydroxy-flavanone 7-*O*-β-D-glucopyranoside	P13866	0.804	JSFDGGQKOKTSOU-PFKOEMKTSA-N	4.87	BindingDB, ChemblDB, PubChem

Table S3. *Cont.*

Molid	Uniprot	SIM	REF_NN	REF_pACT	SOURCE_DB
5,7,3′,5′-Tetrahydroxy-flavanone 7-*O*-β-D-glucopyranoside	P13866	0.804	KPTNFLTZJSDNHO-PFKOEMKTSA-N	4.79	BindingDB, ChemblDB, PubChem
5,7,3′,5′-Tetrahydroxy-flavanone 7-*O*-β-D-glucopyranoside	P13866	0.804	LBKNLPSWXKBZPU-DODNOZFWSA-N	5.43	BindingDB, ChemblDB, PubChem
5,7,3′,5′-Tetrahydroxy-flavanone 7-*O*-β-D-glucopyranoside	P13866	0.804	NLZYYMHUDULKQF-PRDVQWLOSA-N	5.2	BindingDB, ChemblDB, PubChem
5,7,3′,5′-Tetrahydroxy-flavanone 7-*O*-β-D-glucopyranoside	P13866	0.804	RMQQQPQAVFICPZ-PFKOEMKTSA-N	5.8	BindingDB, ChemblDB, PubChem
5,7,3′,5′-Tetrahydroxy-flavanone 7-*O*-β-D-glucopyranoside	P13866	0.8	GMYFQAHYWIYNES-PFKOEMKTSA-N	6.74	BindingDB, ChemblDB, PubChem
5,7,3′,5′-Tetrahydroxy-flavanone 7-*O*-β-D-glucopyranoside	P13866	0.8	HZQBMUPOYJUQAR-XDXGNBCUSA-N	5.88	BindingDB, ChemblDB, PubChem
5,7,3′,5′-Tetrahydroxy-flavanone 7-*O*-β-D-glucopyranoside	P13866	0.8	QAJZQZIOIFEMTH-PRDVQWLOSA-N	5.32	BindingDB, ChemblDB, PubChem
5,7,3′,5′-Tetrahydroxy-flavanone 7-*O*-β-D-glucopyranoside	P13866	0.8	WKPBVEUJSMWTPM-XDXGNBCUSA-N	5	BindingDB, ChemblDB, PubChem
5,7,3′,5′-Tetrahydroxy-flavanone 7-*O*-β-D-glucopyranoside	P13866	0.8	XVBIDTMBHOOAMG-PRDVQWLOSA-N	5.43	BindingDB, ChemblDB, PubChem
5,7,3′,5′-Tetrahydroxy-flavanone 7-*O*-β-D-glucopyranoside	P13866	0.785	CUFHFMHHSMPJBW-UHFFFAOYSA-N	5.11	BindingDB, ChemblDB, PubChem
5,7,3′,5′-Tetrahydroxy-flavanone 7-*O*-β-D-glucopyranoside	P13866	0.785	RYURKHGRTNSWQX-UHFFFAOYSA-N	5.16	BindingDB, ChemblDB, PubChem
5,7,3′,5′-Tetrahydroxy-flavanone 7-*O*-β-D-glucopyranoside	P31639	0.932	IOUVKUPGCMBWBT-QNDFHXLGSA-N	7.18	BindingDB, ChemblDB, PubChem
5,7,3′,5′-Tetrahydroxy-flavanone 7-*O*-β-D-glucopyranoside	P31639	0.825	CLRQMIQNMTYMGA-XIKSMUEASA-N	7.16	BindingDB, ChemblDB, PubChem
5,7,3′,5′-Tetrahydroxy-flavanone 7-*O*-β-D-glucopyranoside	P31639	0.825	VAVAYLQBNLAMRO-XIKSMUEASA-N	6.64	BindingDB, ChemblDB, PubChem
5,7,3′,5′-Tetrahydroxy-flavanone 7-*O*-β-D-glucopyranoside	P31639	0.804	ACMMSHORHBTOEZ-PRDVQWLOSA-N	7.52	BindingDB, ChemblDB, PubChem
5,7,3′,5′-Tetrahydroxy-flavanone 7-*O*-β-D-glucopyranoside	P31639	0.804	IINBYKILNZBSAK-PFKOEMKTSA-N	8.05	BindingDB, ChemblDB, PubChem
5,7,3′,5′-Tetrahydroxy-flavanone 7-*O*-β-D-glucopyranoside	P31639	0.804	IWRUKKIVIXIBRH-DODNOZFWSA-N	7.1	BindingDB, ChemblDB, PubChem
5,7,3′,5′-Tetrahydroxy-flavanone 7-*O*-β-D-glucopyranoside	P31639	0.804	JSFDGGQKOKTSOU-PFKOEMKTSA-N	5.89	BindingDB, ChemblDB, PubChem

Table S3. *Cont.*

Molid	Uniprot	SIM	REF_NN	REF_pACT	SOURCE_DB
5,7,3′,5′-Tetrahydroxy-flavanone 7-*O*-β-D-glucopyranoside	P31639	0.804	KPTNFLTZJSDNHO-PFKOEMKTSA-N	6.54	BindingDB, ChemblDB, PubChem
5,7,3′,5′-Tetrahydroxy-flavanone 7-*O*-β-D-glucopyranoside	P31639	0.804	LBKNLPSWXKBZPU-DODNOZFWSA-N	7.7	BindingDB, ChemblDB, PubChem
5,7,3′,5′-Tetrahydroxy-flavanone 7-*O*-β-D-glucopyranoside	P31639	0.804	NLZYYMHUDULKQF-PRDVQWLOSA-N	8	BindingDB, ChemblDB, PubChem
5,7,3′,5′-Tetrahydroxy-flavanone 7-*O*-β-D-glucopyranoside	P31639	0.804	RMQQQPQAVFICPZ-PFKOEMKTSA-N	8	BindingDB, ChemblDB, PubChem
5,7,3′,5′-Tetrahydroxy-flavanone 7-*O*-β-D-glucopyranoside	P31639	0.8	GMYFQAHYWIYNES-PFKOEMKTSA-N	8.11	BindingDB, ChemblDB, PubChem
5,7,3′,5′-Tetrahydroxy-flavanone 7-*O*-β-D-glucopyranoside	P31639	0.8	HZQBMUPOYJUQAR-XDXGNBCUSA-N	8	BindingDB, ChemblDB, PubChem
5,7,3′,5′-Tetrahydroxy-flavanone 7-*O*-β-D-glucopyranoside	P31639	0.8	QAJZQZIOIFEMTH-PRDVQWLOSA-N	7.52	BindingDB, ChemblDB, PubChem
5,7,3′,5′-Tetrahydroxy-flavanone 7-*O*-β-D-glucopyranoside	P31639	0.8	WKPBVEUJSMWTPM-XDXGNBCUSA-N	6.28	BindingDB, ChemblDB, PubChem
5,7,3′,5′-Tetrahydroxy-flavanone 7-*O*-β-D-glucopyranoside	P31639	0.8	XVBIDTMBHOOAMG-PRDVQWLOSA-N	7.16	BindingDB,ChemblDB,PubChem
Naringenin	P08183	sCOLL	FTVWIRXFELQLPI-ZDUSSCGKSA-N	A	DrugBank
Naringenin	Q9UNQ0	sCOLL	FTVWIRXFELQLPI-ZDUSSCGKSA-N	A	DrugBank
Naringenin	Q9UNQ0	0.901	AIONOLUJZLIMTK-AWEZNQCLSA-N	A	DrugBank
Naringenin	Q16678	sCOLL	FTVWIRXFELQLPI-ZDUSSCGKSA-N	5.44	ChemblDB, PubChem
Naringenin	Q16678	0.908	FTODBIPDTXRIGS-UHFFFAOYSA-N	6.62	BindingDB
Naringenin	Q16678	0.908	FTODBIPDTXRIGS-ZDUSSCGKSA-N	5.76	ChemblDB, PubChem
Naringenin	Q16678	0.901	AIONOLUJZLIMTK-AWEZNQCLSA-N	6.29	ChemblDB, PubChem
Naringenin	Q16678	0.878	KZNIFHPLKGYRTM-UHFFFAOYSA-N	7.6	ChemblDB, PubChem
Naringenin	Q16678	0.878	SBHXYTNGIZCORC-ZDUSSCGKSA-N	5.89	ChemblDB, PubChem
Naringenin	Q16678	0.858	URFCJEUYXNAHFI-ZDUSSCGKSA-N	5.78	ChemblDB, PubChem
Naringenin	Q16678	0.83	HMUJXQRRKBLVOO-AWEZNQCLSA-N	5.99	ChemblDB, PubChem
Naringenin	P10635	0.817	VJJZJBUCDWKPLC-UHFFFAOYSA-N	5.33	BindingDB, ChemblDB, PubChem
Naringenin	P11511	sCOLL	FTVWIRXFELQLPI-ZDUSSCGKSA-N	6.65	BindingDB, ChemblDB, PubChem
Naringenin	P11511	0.878	KZNIFHPLKGYRTM-UHFFFAOYSA-N	6.03	BindingDB, ChemblDB, PubChem
Naringenin	P11511	0.865	QBLQLKNOKUHRCH-ZDUSSCGKSA-N	5.66	BindingDB, ChemblDB, PubChem
Naringenin	P11511	0.858	FURUXTVZLHCCNA-UHFFFAOYSA-N	5.68	ChemblDB, PubChem
Naringenin	P11511	0.836	MXNJBOFUFMJXOB-UHFFFAOYSA-N	5.47	ChemblDB, PubChem
Naringenin	P11511	0.801	CGKWSLSAYABZTL-UHFFFAOYSA-N	6.4	BindingDB, ChemblDB, PubChem
Naringenin	P11511	0.785	XHRMNTNDCDDFQR-ONEGZZNKSA-N	6.7	BindingDB, ChemblDB, PubChem

Table S3. *Cont.*

Molid	Uniprot	SIM	REF_NN	REF_pACT	SOURCE_DB
Naringenin	O95067	0.878	KZNIFHPLKGYRTM-UHFFFAOYSA-N	5.4	ChemblDB
Naringenin	P14635	0.878	KZNIFHPLKGYRTM-UHFFFAOYSA-N	5.4	ChemblDB
Naringenin	Q8WWL7	0.878	KZNIFHPLKGYRTM-UHFFFAOYSA-N	5.4	ChemblDB
Naringenin	P06493	0.878	KZNIFHPLKGYRTM-UHFFFAOYSA-N	5.4	BindingDB, ChemblDB, PubChem
Naringenin	P15121	0.878	KZNIFHPLKGYRTM-UHFFFAOYSA-N	5.18	BindingDB, ChemblDB, PubChem
Naringenin	P14061	COLL	FTVWIRXFELQLPI-UHFFFAOYSA-N	5.3	BindingDB
Naringenin	P14061	sCOLL	FTVWIRXFELQLPI-ZDUSSCGKSA-N	5.3	ChemblDB, PubChem
Naringenin	P14061	0.878	KZNIFHPLKGYRTM-UHFFFAOYSA-N	6.15	BindingDB, ChemblDB, PubChem
Naringenin	P21397	0.878	KZNIFHPLKGYRTM-UHFFFAOYSA-N	5.77	PubChem, ChemblDB, BindingDB
Naringenin	P18054	0.882	ZAIJTQZQMCNJHG-UHFFFAOYSA-N	5.42	BindingDB, ChemblDB, PubChem
Naringenin	P23219	0.865	QBLQLKNOKUHRCH-UHFFFAOYSA-N	5.23	BindingDB, ChemblDB, PubChem
Naringenin	O75908	0.901	AIONOLUJZLIMTK-AWEZNQCLSA-N	A	DrugBank
Naringenin	P35610	0.901	AIONOLUJZLIMTK-AWEZNQCLSA-N	A	DrugBank
Naringenin	P68400	0.878	KZNIFHPLKGYRTM-UHFFFAOYSA-N	5.88	BindingDB, ChemblDB, PubChem
Naringenin	Q00534	0.878	KZNIFHPLKGYRTM-UHFFFAOYSA-N	5.77	ChemblDB, PubChem, BindingDB
Naringenin	P49841	0.878	KZNIFHPLKGYRTM-UHFFFAOYSA-N	5.85	PubChem, BindingDB, ChemblDB
Naringenin	P49840	0.878	KZNIFHPLKGYRTM-UHFFFAOYSA-N	5.85	ChemblDB, PubChem
Naringenin	P08236	0.878	KZNIFHPLKGYRTM-UHFFFAOYSA-N	5.55	PubChem, BindingDB, ChemblDB
Naringenin	P10520	0.795	YHWNASRGLKJRJJ-UHFFFAOYSA-N	5.22	PubChem
Naringenin	P30542	0.878	KZNIFHPLKGYRTM-UHFFFAOYSA-N	5.52	ChemblDB, PubChem, BindingDB
Naringenin	P30542	0.838	DJOJDHGQRNZXQQ-UHFFFAOYSA-N	5.09	ChemblDB, PubChem, BindingDB, IUPHARdb
Naringenin	P33765	0.838	DJOJDHGQRNZXQQ-UHFFFAOYSA-N	5.48	ChemblDB, PubChem, BindingDB, IUPHARdb
Naringenin	P14867	0.878	KZNIFHPLKGYRTM-UHFFFAOYSA-N	5.52	ChemblDB, PubChem
Naringenin	P47869	0.878	KZNIFHPLKGYRTM-UHFFFAOYSA-N	5.52	ChemblDB
Naringenin	P34903	0.878	KZNIFHPLKGYRTM-UHFFFAOYSA-N	5.52	ChemblDB
Naringenin	P48169	0.878	KZNIFHPLKGYRTM-UHFFFAOYSA-N	5.52	ChemblDB
Naringenin	P31644	0.878	KZNIFHPLKGYRTM-UHFFFAOYSA-N	5.52	ChemblDB
Naringenin	Q16445	0.878	KZNIFHPLKGYRTM-UHFFFAOYSA-N	5.52	ChemblDB
Naringenin	P18505	0.878	KZNIFHPLKGYRTM-UHFFFAOYSA-N	6.11	ChemblDB
Naringenin	P47870	0.878	KZNIFHPLKGYRTM-UHFFFAOYSA-N	6.11	ChemblDB
Naringenin	P28472	0.878	KZNIFHPLKGYRTM-UHFFFAOYSA-N	6.11	ChemblDB
Naringenin	O14764	0.878	KZNIFHPLKGYRTM-UHFFFAOYSA-N	6.11	ChemblDB
Naringenin	P78334	0.878	KZNIFHPLKGYRTM-UHFFFAOYSA-N	6.11	ChemblDB
Naringenin	Q8N1C3	0.878	KZNIFHPLKGYRTM-UHFFFAOYSA-N	6.11	ChemblDB

Table S3. *Cont.*

Molid	Uniprot	SIM	REF_NN	REF_pACT	SOURCE_DB
Naringenin	P18507	0.878	KZNIFHPLKGYRTM-UHFFFAOYSA-N	6.11	ChemblDB
Naringenin	Q99928	0.878	KZNIFHPLKGYRTM-UHFFFAOYSA-N	6.11	ChemblDB
Naringenin	O00591	0.878	KZNIFHPLKGYRTM-UHFFFAOYSA-N	6.11	ChemblDB
Naringenin	Q9UN88	0.878	KZNIFHPLKGYRTM-UHFFFAOYSA-N	6.11	ChemblDB
Naringenin	P10275	0.878	KZNIFHPLKGYRTM-UHFFFAOYSA-N	5.28	BindingDB, ChemblDB, PubChem
Naringenin	P33527	sCOLL	FTVWIRXFELQLPI-ZDUSSCGKSA-N	5.62	BindingDB, ChemblDB, PubChem, DrugBank
Naringenin	P33527	0.878	KZNIFHPLKGYRTM-UHFFFAOYSA-N	5.62	BindingDB, ChemblDB, PubChem
Naringenin	O94956	sCOLL	FTVWIRXFELQLPI-ZDUSSCGKSA-N	A	DrugBank
Naringenin	P55157	0.901	AIONOLUJZLIMTK-AWEZNQCLSA-N	A	DrugBank
Naringenin	P67870	0.878	KZNIFHPLKGYRTM-UHFFFAOYSA-N	5.37	ChemblDB, PubChem
Naringenin	Q07820	0.941	SLFZBNOERHGNMI-UHFFFAOYSA-N	5.4	PubChem
Naringenin	P90584	0.878	KZNIFHPLKGYRTM-UHFFFAOYSA-N	5.16	BindingDB, ChemblDB
Naringenin	Q15078	0.878	KZNIFHPLKGYRTM-UHFFFAOYSA-N	5.8	ChemblDB
Naringenin	Q5G940	0.878	KZNIFHPLKGYRTM-UHFFFAOYSA-N	A	DrugBank
Naringenin	Q5G940	0.838	DJOJDHGQRNZXQQ-AWEZNQCLSA-N	6.05	DrugBank, MOAD
5,7,3′,5′-Tetrahydroxyflavanone	P08183	0.89	FTVWIRXFELQLPI-ZDUSSCGKSA-N	A	DrugBank
5,7,3′,5′-Tetrahydroxyflavanone	Q9UNQ0	0.89	FTVWIRXFELQLPI-ZDUSSCGKSA-N	A	DrugBank
5,7,3′,5′-Tetrahydroxyflavanone	Q9UNQ0	0.856	AIONOLUJZLIMTK-AWEZNQCLSA-N	A	DrugBank
5,7,3′,5′-Tetrahydroxyflavanone	P29768	0.878	IQPNAANSBPBGFQ-UHFFFAOYSA-N	5.37	BindingDB, ChemblDB, PubChem
5,7,3′,5′-Tetrahydroxyflavanone	P29768	0.79	IYRMWMYZSQPJKC-UHFFFAOYSA-N	5.1	BindingDB, ChemblDB, PubChem
5,7,3′,5′-Tetrahydroxyflavanone	Q16678	sCOLL	SBHXYTNGIZCORC-ZDUSSCGKSA-N	5.89	ChemblDB, PubChem
5,7,3′,5′-Tetrahydroxyflavanone	Q16678	0.89	FTVWIRXFELQLPI-ZDUSSCGKSA-N	5.44	ChemblDB, PubChem
5,7,3′,5′-Tetrahydroxyflavanone	Q16678	0.878	IQPNAANSBPBGFQ-UHFFFAOYSA-N	7.1	ChemblDB, PubChem
5,7,3′,5′-Tetrahydroxyflavanone	Q16678	0.868	FTODBIPDTXRIGS-UHFFFAOYSA-N	6.62	BindingDB
5,7,3′,5′-Tetrahydroxyflavanone	Q16678	0.868	FTODBIPDTXRIGS-ZDUSSCGKSA-N	5.76	ChemblDB, PubChem
5,7,3′,5′-Tetrahydroxyflavanone	Q16678	0.856	AIONOLUJZLIMTK-AWEZNQCLSA-N	6.29	ChemblDB, PubChem
5,7,3′,5′-Tetrahydroxyflavanone	Q16678	0.79	IYRMWMYZSQPJKC-UHFFFAOYSA-N	7.33	ChemblDB, PubChem
5,7,3′,5′-Tetrahydroxyflavanone	P11712	0.79	IYRMWMYZSQPJKC-UHFFFAOYSA-N	5.22	BindingDB, ChemblDB, PubChem
5,7,3′,5′-Tetrahydroxyflavanone	P11511	0.935	QBLQLKNOKUHRCH-ZDUSSCGKSA-N	5.66	BindingDB, ChemblDB, PubChem
5,7,3′,5′-Tetrahydroxyflavanone	P11511	0.89	FTVWIRXFELQLPI-ZDUSSCGKSA-N	6.65	BindingDB, ChemblDB, PubChem
5,7,3′,5′-Tetrahydroxyflavanone	P11511	0.774	FURUXTVZLHCCNA-UHFFFAOYSA-N	5.68	ChemblDB, PubChem
5,7,3′,5′-Tetrahydroxyflavanone	P11511	0.774	MXNJBOFUFMJXOB-UHFFFAOYSA-N	5.47	ChemblDB, PubChem
5,7,3′,5′-Tetrahydroxyflavanone	O95067	0.878	IQPNAANSBPBGFQ-UHFFFAOYSA-N	5.21	ChemblDB
5,7,3′,5′-Tetrahydroxyflavanone	O95067	0.79	IYRMWMYZSQPJKC-UHFFFAOYSA-N	4.39	ChemblDB
5,7,3′,5′-Tetrahydroxyflavanone	P14635	0.878	IQPNAANSBPBGFQ-UHFFFAOYSA-N	5.21	ChemblDB

Table S3. *Cont.*

Molid	Uniprot	SIM	REF_NN	REF_pACT	SOURCE_DB
5,7,3′,5′-Tetrahydroxyflavanone	P14635	0.79	IYRMWMYZSQPJKC-UHFFFAOYSA-N	4.39	ChemblDB
5,7,3′,5′-Tetrahydroxyflavanone	Q8WWL7	0.878	IQPNAANSBPBGFQ-UHFFFAOYSA-N	5.21	ChemblDB
5,7,3′,5′-Tetrahydroxyflavanone	Q8WWL7	0.79	IYRMWMYZSQPJKC-UHFFFAOYSA-N	4.39	ChemblDB
5,7,3′,5′-Tetrahydroxyflavanone	P06493	0.878	IQPNAANSBPBGFQ-UHFFFAOYSA-N	5.21	BindingDB, ChemblDB, PubChem
5,7,3′,5′-Tetrahydroxyflavanone	P06493	0.79	IYRMWMYZSQPJKC-UHFFFAOYSA-N	4.39	BindingDB, ChemblDB, PubChem
5,7,3′,5′-Tetrahydroxyflavanone	O14746	0.769	ARYCMKPCDNHQCL-UHFFFAOYSA-N	6.7	PubChem, BindingDB, ChemblDB
5,7,3′,5′-Tetrahydroxyflavanone	O14746	0.767	GHPOEPBXSFQHEL-UHFFFAOYSA-N	6.1	PubChem, BindingDB, ChemblDB
5,7,3′,5′-Tetrahydroxyflavanone	O14746	0.767	YINCNTBPLYVMFY-UHFFFAOYSA-N	6.22	PubChem, BindingDB, ChemblDB
5,7,3′,5′-Tetrahydroxyflavanone	P11387	0.878	IQPNAANSBPBGFQ-UHFFFAOYSA-N	6.18	BindingDB, ChemblDB, PubChem
5,7,3′,5′-Tetrahydroxyflavanone	P11387	0.803	JEYUVFHPIMHRPG-XCVCLJGOSA-N	5.52	PubChem
5,7,3′,5′-Tetrahydroxyflavanone	P0A7G6	0.785	PFTAWBLQPZVEMU-DZGCQCFKSA-N	5.39	PubChem
5,7,3′,5′-Tetrahydroxyflavanone	P15121	0.878	IQPNAANSBPBGFQ-UHFFFAOYSA-N	5.02	BindingDB, ChemblDB, PubChem
5,7,3′,5′-Tetrahydroxyflavanone	P15121	0.82	FPSMUVCMXQTXND-UHFFFAOYSA-N	6.55	ChemblDB
5,7,3′,5′-Tetrahydroxyflavanone	P15121	0.792	WTOWZADSSKQQQE-PWJLMRLQSA-N	4.02	ChemblDB, PubChem
5,7,3′,5′-Tetrahydroxyflavanone	P15121	0.79	IYRMWMYZSQPJKC-UHFFFAOYSA-N	5.88	BindingDB, ChemblDB, PubChem
5,7,3′,5′-Tetrahydroxyflavanone	P15121	0.785	PFTAWBLQPZVEMU-DZGCQCFKSA-N	3.52	BindingDB
5,7,3′,5′-Tetrahydroxyflavanone	P15121	0.785	PFTAWBLQPZVEMU-UKRRQHHQSA-N	4.02	BindingDB, ChemblDB, PubChem
5,7,3′,5′-Tetrahydroxyflavanone	P15121	0.766	RRYQDECFPVYHLR-UHFFFAOYSA-N	6.62	ChemblDB
5,7,3′,5′-Tetrahydroxyflavanone	P14061	0.89	FTVWIRXFELQLPI-UHFFFAOYSA-N	5.3	BindingDB
5,7,3′,5′-Tetrahydroxyflavanone	P14061	0.89	FTVWIRXFELQLPI-ZDUSSCGKSA-N	5.3	ChemblDB, PubChem
5,7,3′,5′-Tetrahydroxyflavanone	P14061	0.79	IYRMWMYZSQPJKC-UHFFFAOYSA-N	5.98	BindingDB, ChemblDB, PubChem
5,7,3′,5′-Tetrahydroxyflavanone	P37059	0.89	FTVWIRXFELQLPI-UHFFFAOYSA-N	4.84	BindingDB
5,7,3′,5′-Tetrahydroxyflavanone	P37059	0.89	FTVWIRXFELQLPI-ZDUSSCGKSA-N	4.84	ChemblDB, PubChem
5,7,3′,5′-Tetrahydroxyflavanone	P37059	0.79	IYRMWMYZSQPJKC-UHFFFAOYSA-N	6.44	BindingDB, ChemblDB, PubChem
5,7,3′,5′-Tetrahydroxyflavanone	Q965D6	0.878	IQPNAANSBPBGFQ-UHFFFAOYSA-N	5.75	BindingDB, ChemblDB
5,7,3′,5′-Tetrahydroxyflavanone	Q965D6	0.79	IYRMWMYZSQPJKC-UHFFFAOYSA-N	5.4	BindingDB, ChemblDB
5,7,3′,5′-Tetrahydroxyflavanone	Q965D5	0.878	IQPNAANSBPBGFQ-UHFFFAOYSA-N	5.68	BindingDB, ChemblDB
5,7,3′,5′-Tetrahydroxyflavanone	Q965D5	0.844	AYMYWHCQALZEGT-ORCRQEGFSA-N	5.23	BindingDB, ChemblDB
5,7,3′,5′-Tetrahydroxyflavanone	Q965D5	0.79	IYRMWMYZSQPJKC-UHFFFAOYSA-N	4.7	BindingDB, ChemblDB
5,7,3′,5′-Tetrahydroxyflavanone	P21397	0.878	IQPNAANSBPBGFQ-UHFFFAOYSA-N	5.31	PubChem, ChemblDB, BindingDB
5,7,3′,5′-Tetrahydroxyflavanone	Q06327	0.878	IQPNAANSBPBGFQ-UHFFFAOYSA-N	5.5	BindingDB, ChemblDB, PubChem
5,7,3′,5′-Tetrahydroxyflavanone	P18054	0.828	ZAIJTQZQMCNJHG-UHFFFAOYSA-N	5.42	BindingDB, ChemblDB, PubChem
5,7,3′,5′-Tetrahydroxyflavanone	P09917	0.844	AYMYWHCQALZEGT-ORCRQEGFSA-N	8.34	PubChem, BindingDB, ChemblDB
5,7,3′,5′-Tetrahydroxyflavanone	P09917	0.79	IYRMWMYZSQPJKC-UHFFFAOYSA-N	5.57	PubChem, BindingDB, ChemblDB
5,7,3′,5′-Tetrahydroxyflavanone	O75908	0.856	AIONOLUJZLIMTK-AWEZNQCLSA-N	A	DrugBank
5,7,3′,5′-Tetrahydroxyflavanone	P35610	0.856	AIONOLUJZLIMTK-AWEZNQCLSA-N	A	DrugBank

Table S3. *Cont.*

Molid	Uniprot	SIM	REF_NN	REF_pACT	SOURCE_DB
5,7,3′,5′-Tetrahydroxyflavanone	Q6NUS8	0.79	IYRMWMYZSQPJKC-UHFFFAOYSA-N	A	DrugBank
5,7,3′,5′-Tetrahydroxyflavanone	P11309	0.878	IQPNAANSBPBGFQ-UHFFFAOYSA-N	5.8	BindingDB
5,7,3′,5′-Tetrahydroxyflavanone	P11309	0.807	CCCIGFPBADVTFE-UHFFFAOYSA-N	5.11	BindingDB
5,7,3′,5′-Tetrahydroxyflavanone	P11309	0.79	IYRMWMYZSQPJKC-UHFFFAOYSA-N	5.89	BindingDB
5,7,3′,5′-Tetrahydroxyflavanone	P49841	0.878	IQPNAANSBPBGFQ-UHFFFAOYSA-N	5.76	PubChem, BindingDB, ChemblDB
5,7,3′,5′-Tetrahydroxyflavanone	P49841	0.79	IYRMWMYZSQPJKC-UHFFFAOYSA-N	5.2	PubChem, BindingDB, ChemblDB
5,7,3′,5′-Tetrahydroxyflavanone	P49840	0.878	IQPNAANSBPBGFQ-UHFFFAOYSA-N	6.1	ChemblDB, PubChem
5,7,3′,5′-Tetrahydroxyflavanone	P49840	0.79	IYRMWMYZSQPJKC-UHFFFAOYSA-N	5.46	ChemblDB, PubChem
5,7,3′,5′-Tetrahydroxyflavanone	Q8I2J3	0.785	PFTAWBLQPZVEMU-UKRRQHHQSA-N	5.86	PubChem
5,7,3′,5′-Tetrahydroxyflavanone	P03070	0.94	ZPVNWCMRCGXRJD-UHFFFAOYSA-N	5.72	PubChem
5,7,3′,5′-Tetrahydroxyflavanone	P03070	0.878	IQPNAANSBPBGFQ-UHFFFAOYSA-N	4.62	PubChem
5,7,3′,5′-Tetrahydroxyflavanone	P03070	0.868	FTODBIPDTXRIGS-UHFFFAOYSA-N	4	PubChem
5,7,3′,5′-Tetrahydroxyflavanone	P03070	0.785	PFTAWBLQPZVEMU-DZGCQCFKSA-N	4.94	PubChem
5,7,3′,5′-Tetrahydroxyflavanone	P03070	0.785	PFTAWBLQPZVEMU-UHFFFAOYSA-N	5.06	PubChem
5,7,3′,5′-Tetrahydroxyflavanone	O43570	0.785	PFTAWBLQPZVEMU-HIFRSBDPSA-N	5.33	ChemblDB, PubChem
5,7,3′,5′-Tetrahydroxyflavanone	P00915	0.785	PFTAWBLQPZVEMU-HIFRSBDPSA-N	5.62	ChemblDB, PubChem
5,7,3′,5′-Tetrahydroxyflavanone	P00918	0.785	PFTAWBLQPZVEMU-HIFRSBDPSA-N	5.74	ChemblDB, PubChem
5,7,3′,5′-Tetrahydroxyflavanone	P07451	0.785	PFTAWBLQPZVEMU-HIFRSBDPSA-N	5.45	ChemblDB, PubChem
5,7,3′,5′-Tetrahydroxyflavanone	P22748	0.785	PFTAWBLQPZVEMU-HIFRSBDPSA-N	5.31	ChemblDB, PubChem
5,7,3′,5′-Tetrahydroxyflavanone	P23280	0.785	PFTAWBLQPZVEMU-HIFRSBDPSA-N	5.31	ChemblDB, PubChem
5,7,3′,5′-Tetrahydroxyflavanone	P35218	0.785	PFTAWBLQPZVEMU-HIFRSBDPSA-N	5.38	ChemblDB, PubChem
5,7,3′,5′-Tetrahydroxyflavanone	P43166	0.785	PFTAWBLQPZVEMU-HIFRSBDPSA-N	6.35	ChemblDB, PubChem
5,7,3′,5′-Tetrahydroxyflavanone	Q16790	0.785	PFTAWBLQPZVEMU-HIFRSBDPSA-N	5.3	ChemblDB, PubChem
5,7,3′,5′-Tetrahydroxyflavanone	Q99N23	0.785	PFTAWBLQPZVEMU-HIFRSBDPSA-N	5.12	ChemblDB, PubChem
5,7,3′,5′-Tetrahydroxyflavanone	Q9Y2D0	0.785	PFTAWBLQPZVEMU-HIFRSBDPSA-N	5.4	ChemblDB, PubChem
5,7,3′,5′-Tetrahydroxyflavanone	P30542	0.878	IQPNAANSBPBGFQ-UHFFFAOYSA-N	5.79	ChemblDB, PubChem
5,7,3′,5′-Tetrahydroxyflavanone	P03372	0.89	FTVWIRXFELQLPI-UHFFFAOYSA-N	4.14	BindingDB
5,7,3′,5′-Tetrahydroxyflavanone	P03372	0.89	FTVWIRXFELQLPI-ZDUSSCGKSA-N	4.12	ChemblDB, PubChem
5,7,3′,5′-Tetrahydroxyflavanone	P03372	0.848	GMVYLXBMPRDZDR-AWEZNQCLSA-N	5.28	ChemblDB, PubChem
5,7,3′,5′-Tetrahydroxyflavanone	P03372	0.848	GMVYLXBMPRDZDR-UHFFFAOYSA-N	5.28	BindingDB
5,7,3′,5′-Tetrahydroxyflavanone	P03372	0.848	PFCDDRUHZAMKHP-INIZCTEOSA-N	6.81	ChemblDB, PubChem
5,7,3′,5′-Tetrahydroxyflavanone	P03372	0.848	PFCDDRUHZAMKHP-UHFFFAOYSA-N	6.81	BindingDB
5,7,3′,5′-Tetrahydroxyflavanone	P03372	0.822	ZYVQEHNABDHJGN-KRWDZBQOSA-N	7.1	ChemblDB, PubChem
5,7,3′,5′-Tetrahydroxyflavanone	P03372	0.822	ZYVQEHNABDHJGN-UHFFFAOYSA-N	7.1	BindingDB
5,7,3′,5′-Tetrahydroxyflavanone	P03372	0.817	UDGKKUWYNITJRX-UHFFFAOYSA-N	3	ChemblDB
5,7,3′,5′-Tetrahydroxyflavanone	Q92731	0.89	FTVWIRXFELQLPI-UHFFFAOYSA-N	4.87	BindingDB

Table S3. *Cont.*

Molid	Uniprot	SIM	REF_NN	REF_pACT	SOURCE_DB
5,7,3′,5′-Tetrahydroxyflavanone	Q92731	0.89	FTVWIRXFELQLPI-ZDUSSCGKSA-N	4.87	PubChem, ChemblDB
5,7,3′,5′-Tetrahydroxyflavanone	Q92731	0.848	GMVYLXBMPRDZDR-AWEZNQCLSA-N	6.06	PubChem, ChemblDB
5,7,3′,5′-Tetrahydroxyflavanone	Q92731	0.848	GMVYLXBMPRDZDR-UHFFFAOYSA-N	6.06	BindingDB
5,7,3′,5′-Tetrahydroxyflavanone	Q92731	0.848	PFCDDRUHZAMKHP-INIZCTEOSA-N	7.54	PubChem, ChemblDB
5,7,3′,5′-Tetrahydroxyflavanone	Q92731	0.848	PFCDDRUHZAMKHP-UHFFFAOYSA-N	7.54	BindingDB
5,7,3′,5′-Tetrahydroxyflavanone	Q92731	0.822	ZYVQEHNABDHJGN-KRWDZBQOSA-N	7.43	PubChem, ChemblDB
5,7,3′,5′-Tetrahydroxyflavanone	Q92731	0.822	ZYVQEHNABDHJGN-UHFFFAOYSA-N	7.43	BindingDB
5,7,3′,5′-Tetrahydroxyflavanone	Q92731	0.817	UDGKKUWYNITJRX-UHFFFAOYSA-N	3	ChemblDB
5,7,3′,5′-Tetrahydroxyflavanone	P10275	0.79	IYRMWMYZSQPJKC-UHFFFAOYSA-N	5.01	BindingDB, ChemblDB, PubChem
5,7,3′,5′-Tetrahydroxyflavanone	P33527	0.89	FTVWIRXFELQLPI-ZDUSSCGKSA-N	5.62	BindingDB, ChemblDB, PubChem, DrugBank
5,7,3′,5′-Tetrahydroxyflavanone	P33527	0.79	IYRMWMYZSQPJKC-UHFFFAOYSA-N	5.62	BindingDB, ChemblDB, PubChem
5,7,3′,5′-Tetrahydroxyflavanone	P35869	0.825	PADQINQHPQKXNL-UHFFFAOYSA-N	7.55	BindingDB
5,7,3′,5′-Tetrahydroxyflavanone	P35869	0.79	IYRMWMYZSQPJKC-UHFFFAOYSA-N	7.55	PubChem, ChemblDB
5,7,3′,5′-Tetrahydroxyflavanone	Q88N29	0.89	FTVWIRXFELQLPI-UHFFFAOYSA-N	4.94	BindingDB
5,7,3′,5′-Tetrahydroxyflavanone	Q88N29	0.88	VGEREEWJJVICBM-UHFFFAOYSA-N	5.96	BindingDB
5,7,3′,5′-Tetrahydroxyflavanone	Q88N29	0.878	IQPNAANSBPBGFQ-UHFFFAOYSA-N	5.14	BindingDB
5,7,3′,5′-Tetrahydroxyflavanone	O94956	0.89	FTVWIRXFELQLPI-ZDUSSCGKSA-N	A	DrugBank
5,7,3′,5′-Tetrahydroxyflavanone	P55157	0.856	AIONOLUJZLIMTK-AWEZNQCLSA-N	A	DrugBank
5,7,3′,5′-Tetrahydroxyflavanone	Q07820	0.94	ZPVNWCMRCGXRJD-UHFFFAOYSA-N	5.52	PubChem
5,7,3′,5′-Tetrahydroxyflavanone	Q07820	0.842	SLFZBNOERHGNMI-UHFFFAOYSA-N	5.4	PubChem
5,7,3′,5′-Tetrahydroxyflavanone	Q15078	0.878	IQPNAANSBPBGFQ-UHFFFAOYSA-N	5.42	ChemblDB
5,7,3′,5′-Tetrahydroxyflavanone	Q15078	0.79	IYRMWMYZSQPJKC-UHFFFAOYSA-N	4.29	ChemblDB
5,7,3′,5′-Tetrahydroxyflavanone	Q9AIU0	0.89	FTVWIRXFELQLPI-ZDUSSCGKSA-N	4.74	MOAD, DrugBank
5,7,3′,5′-Tetrahydroxyflavanone	Q9AIU0	0.88	VGEREEWJJVICBM-UHFFFAOYSA-N	7.3	MOAD, DrugBank
Kushennol F	P23219	0.904	LTTQKYMNTNISSZ-KESSSICBSA-N	6.22	ChemblDB, PubChem
Kushennol F	P23219	0.904	LTTQKYMNTNISSZ-MWTRTKDXSA-N	6.22	BindingDB
Kushennol F	P23219	0.884	PIAPWPAWQGDOMN-PKNBQFBNSA-N	6.22	BindingDB
Kushennol F	P23219	0.884	PIAPWPAWQGDOMN-SXAWMYDMSA-N	6.22	ChemblDB, PubChem
Kushennol F	P27815	0.884	PIAPWPAWQGDOMN-SXAWMYDMSA-N	5.08	ChemblDB
Kushennol F	Q07343	0.884	PIAPWPAWQGDOMN-SXAWMYDMSA-N	5.08	ChemblDB, PubChem
Kushennol F	Q08493	0.884	PIAPWPAWQGDOMN-SXAWMYDMSA-N	5.08	ChemblDB
Kushennol F	Q08499	0.884	PIAPWPAWQGDOMN-SXAWMYDMSA-N	5.08	ChemblDB
Kushennol F	Q13370	0.884	PIAPWPAWQGDOMN-SXAWMYDMSA-N	5.9	ChemblDB, PubChem
Kushennol F	Q14432	0.884	PIAPWPAWQGDOMN-SXAWMYDMSA-N	5.9	ChemblDB
Kushennol F	O76074	0.884	PIAPWPAWQGDOMN-PKNBQFBNSA-N	6.19	BindingDB

Table S3. *Cont.*

Molid	Uniprot	SIM	REF_NN	REF_pACT	SOURCE_DB
Kushennol F	O76074	0.884	PIAPWPAWQGDOMN-SXAWMYDMSA-N	6.19	ChemblDB, PubChem
Kushennol F	P43235	0.841	FVNFXIPJDHVJGE-REZTVBANSA-N	6.77	ChemblDB, PubChem, BindingDB
Kushennol F	P56817	0.976	XRYVAQQLDYTHCL-UHFFFAOYSA-N	5.62	ChemblDB, BindingDB
Kushennol F	P56817	0.904	LTTQKYMNTNISSZ-KESSSICBSA-N	5.48	ChemblDB
Kushennol F	P56817	0.904	LTTQKYMNTNISSZ-UHFFFAOYSA-N	5.48	BindingDB
Kushennol F	P56817	0.904	YLTPWCZXKJSORQ-GYCJOSAFSA-N	5.25	ChemblDB
Kushennol F	P56817	0.904	YLTPWCZXKJSORQ-UHFFFAOYSA-N	5.25	BindingDB
Kushennol F	P56817	0.89	OGBMVWVBHWHRGD-MWTRTKDXSA-N	5.55	ChemblDB
Kushennol F	P56817	0.89	OGBMVWVBHWHRGD-UHFFFAOYSA-N	5.55	BindingDB
Kushennol F	P03372	0.904	LTTQKYMNTNISSZ-KESSSICBSA-N	5.56	ChemblDB, PubChem
Kushennol F	P31639	0.976	XRYVAQQLDYTHCL-CMJOXMDJSA-N	5.39	BindingDB, ChemblDB, PubChem
Kushennol F	P31639	0.904	LTTQKYMNTNISSZ-KESSSICBSA-N	5.77	BindingDB, ChemblDB, PubChem
Sophoraflavanone G	P23219	0.898	LTTQKYMNTNISSZ-KESSSICBSA-N	6.22	ChemblDB,PubChem
Sophoraflavanone G	P23219	0.898	LTTQKYMNTNISSZ-MWTRTKDXSA-N	6.22	BindingDB
Sophoraflavanone G	P23219	0.864	PIAPWPAWQGDOMN-PKNBQFBNSA-N	6.22	BindingDB
Sophoraflavanone G	P23219	0.864	PIAPWPAWQGDOMN-SXAWMYDMSA-N	6.22	ChemblDB, PubChem
Sophoraflavanone G	P27815	0.864	PIAPWPAWQGDOMN-SXAWMYDMSA-N	5.08	ChemblDB
Sophoraflavanone G	Q07343	0.864	PIAPWPAWQGDOMN-SXAWMYDMSA-N	5.08	ChemblDB, PubChem
Sophoraflavanone G	Q08493	0.864	PIAPWPAWQGDOMN-SXAWMYDMSA-N	5.08	ChemblDB
Sophoraflavanone G	Q08499	0.864	PIAPWPAWQGDOMN-SXAWMYDMSA-N	5.08	ChemblDB
Sophoraflavanone G	Q13370	0.864	PIAPWPAWQGDOMN-SXAWMYDMSA-N	5.9	ChemblDB, PubChem
Sophoraflavanone G	Q14432	0.864	PIAPWPAWQGDOMN-SXAWMYDMSA-N	5.9	ChemblDB
Sophoraflavanone G	O76074	0.864	PIAPWPAWQGDOMN-PKNBQFBNSA-N	6.19	BindingDB
Sophoraflavanone G	O76074	0.864	PIAPWPAWQGDOMN-SXAWMYDMSA-N	6.19	ChemblDB, PubChem
Sophoraflavanone G	P43235	0.838	FVNFXIPJDHVJGE-REZTVBANSA-N	6.77	ChemblDB, PubChem, BindingDB
Sophoraflavanone G	P56817	COLL	XRYVAQQLDYTHCL-UHFFFAOYSA-N	5.62	ChemblDB, BindingDB
Sophoraflavanone G	P56817	0.898	LTTQKYMNTNISSZ-KESSSICBSA-N	5.48	ChemblDB
Sophoraflavanone G	P56817	0.898	LTTQKYMNTNISSZ-UHFFFAOYSA-N	5.48	BindingDB
Sophoraflavanone G	P56817	0.897	YLTPWCZXKJSORQ-GYCJOSAFSA-N	5.25	ChemblDB
Sophoraflavanone G	P56817	0.897	YLTPWCZXKJSORQ-UHFFFAOYSA-N	5.25	BindingDB
Sophoraflavanone G	P56817	0.89	OGBMVWVBHWHRGD-MWTRTKDXSA-N	5.55	ChemblDB
Sophoraflavanone G	P56817	0.89	OGBMVWVBHWHRGD-UHFFFAOYSA-N	5.55	BindingDB
Sophoraflavanone G	P56817	0.76	PMFICSJNAUBMIQ-QRQCRPRQSA-N	4.43	ChemblDB
Sophoraflavanone G	P56817	0.76	PMFICSJNAUBMIQ-UHFFFAOYSA-N	4.43	BindingDB
Sophoraflavanone G	P03372	0.898	LTTQKYMNTNISSZ-KESSSICBSA-N	5.56	ChemblDB, PubChem
Sophoraflavanone G	P31639	sCOLL	XRYVAQQLDYTHCL-CMJOXMDJSA-N	5.39	BindingDB, ChemblDB, PubChem
Sophoraflavanone G	P31639	0.898	LTTQKYMNTNISSZ-KESSSICBSA-N	5.77	BindingDB, ChemblDB, PubChem

Table S3. *Cont.*

Molid	Uniprot	SIM	REF_NN	REF_pACT	SOURCE_DB
Kurarinone	Q9BQF6	0.777	SVTCZHIDEDUTBH-UHFFFAOYSA-N	5.11	PubChem
Kurarinone	P11511	0.864	JJOUBYOHNYJCOU-IBGZPJMESA-N	5.47	BindingDB, ChemblDB, PubChem
Kurarinone	P23219	sCOLL	LTTQKYMNTNISSZ-KESSSICBSA-N	6.22	ChemblDB, PubChem
Kurarinone	P23219	sCOLL	LTTQKYMNTNISSZ-MWTRTKDXSA-N	6.22	BindingDB
Kurarinone	P23219	0.776	PIAPWPAWQGDOMN-PKNBQFBNSA-N	6.22	BindingDB
Kurarinone	P23219	0.776	PIAPWPAWQGDOMN-SXAWMYDMSA-N	6.22	ChemblDB, PubChem
Kurarinone	P04054	0.813	LQHKFMYWTKORCE-QFIPXVFZSA-N	5.22	BindingDB, ChemblDB, PubChem
Kurarinone	P27815	0.776	PIAPWPAWQGDOMN-SXAWMYDMSA-N	5.08	ChemblDB
Kurarinone	Q07343	0.776	PIAPWPAWQGDOMN-SXAWMYDMSA-N	5.08	ChemblDB, PubChem
Kurarinone	Q08493	0.776	PIAPWPAWQGDOMN-SXAWMYDMSA-N	5.08	ChemblDB
Kurarinone	Q08499	0.776	PIAPWPAWQGDOMN-SXAWMYDMSA-N	5.08	ChemblDB
Kurarinone	Q13370	0.776	PIAPWPAWQGDOMN-SXAWMYDMSA-N	5.9	ChemblDB, PubChem
Kurarinone	Q14432	0.776	PIAPWPAWQGDOMN-SXAWMYDMSA-N	5.9	ChemblDB
Kurarinone	O76074	0.776	PIAPWPAWQGDOMN-PKNBQFBNSA-N	6.19	BindingDB
Kurarinone	O76074	0.776	PIAPWPAWQGDOMN-SXAWMYDMSA-N	6.19	ChemblDB, PubChem
Kurarinone	P56817	COLL	LTTQKYMNTNISSZ-UHFFFAOYSA-N	5.48	BindingDB
Kurarinone	P56817	sCOLL	LTTQKYMNTNISSZ-KESSSICBSA-N	5.48	ChemblDB
Kurarinone	P56817	0.968	YLTPWCZXKJSORQ-GYCJOSAFSA-N	5.25	ChemblDB
Kurarinone	P56817	0.968	YLTPWCZXKJSORQ-UHFFFAOYSA-N	5.25	BindingDB
Kurarinone	P56817	0.911	OGBMVWVBHWHRGD-MWTRTKDXSA-N	5.55	ChemblDB
Kurarinone	P56817	0.911	OGBMVWVBHWHRGD-UHFFFAOYSA-N	5.55	BindingDB
Kurarinone	P56817	0.898	XRYVAQQLDYTHCL-UHFFFAOYSA-N	5.62	ChemblDB, BindingDB
Kurarinone	P56817	0.895	KTAQQSUPNZAWEY-OSPHWJPCSA-N	5.17	ChemblDB
Kurarinone	P56817	0.895	KTAQQSUPNZAWEY-UHFFFAOYSA-N	5.17	BindingDB
Kurarinone	P10520	0.829	RYBGOKVPJPOMQW-UHFFFAOYSA-N	5.73	PubChem
Kurarinone	P10520	0.777	SVTCZHIDEDUTBH-UHFFFAOYSA-N	5.56	PubChem
Kurarinone	P03372	sCOLL	LTTQKYMNTNISSZ-KESSSICBSA-N	5.56	ChemblDB, PubChem
Kurarinone	P31639	sCOLL	LTTQKYMNTNISSZ-KESSSICBSA-N	5.77	BindingDB, ChemblDB, PubChem
Kurarinone	P31639	0.898	XRYVAQQLDYTHCL-CMJOXMDJSA-N	5.39	BindingDB, ChemblDB, PubChem
Leachianone A	Q9BQF6	0.856	SVTCZHIDEDUTBH-UHFFFAOYSA-N	5.11	PubChem
Leachianone A	P11511	0.867	CGKWSLSAYABZTL-UHFFFAOYSA-N	6.4	BindingDB, ChemblDB, PubChem
Leachianone A	P23219	0.968	LTTQKYMNTNISSZ-KESSSICBSA-N	6.22	ChemblDB, PubChem
Leachianone A	P23219	0.968	LTTQKYMNTNISSZ-MWTRTKDXSA-N	6.22	BindingDB
Leachianone A	P04054	0.906	LQHKFMYWTKORCE-QFIPXVFZSA-N	5.22	BindingDB, ChemblDB, PubChem
Leachianone A	P56817	COLL	YLTPWCZXKJSORQ-UHFFFAOYSA-N	5.25	BindingDB
Leachianone A	P56817	sCOLL	YLTPWCZXKJSORQ-GYCJOSAFSA-N	5.25	ChemblDB

Table S3. *Cont.*

Molid	Uniprot	SIM	REF_NN	REF_pACT	SOURCE_DB
Leachianone A	P56817	0.968	LTTQKYMNTNISSZ-KESSSICBSA-N	5.48	ChemblDB
Leachianone A	P56817	0.968	LTTQKYMNTNISSZ-UHFFFAOYSA-N	5.48	BindingDB
Leachianone A	P56817	0.899	OGBMVWVBHWHRGD-MWTRTKDXSA-N	5.55	ChemblDB
Leachianone A	P56817	0.899	OGBMVWVBHWHRGD-UHFFFAOYSA-N	5.55	BindingDB
Leachianone A	P56817	0.897	XRYVAQQLDYTHCL-UHFFFAOYSA-N	5.62	ChemblDB, BindingDB
Leachianone A	P56817	0.895	KTAQQSUPNZAWEY-OSPHWJPCSA-N	5.17	ChemblDB
Leachianone A	P56817	0.895	KTAQQSUPNZAWEY-UHFFFAOYSA-N	5.17	BindingDB
Leachianone A	P10520	0.856	SVTCZHIDEDUTBH-UHFFFAOYSA-N	5.56	PubChem
Leachianone A	P10520	0.807	RYBGOKVPJPOMQW-UHFFFAOYSA-N	5.73	PubChem
Leachianone A	P03372	0.968	LTTQKYMNTNISSZ-KESSSICBSA-N	5.56	ChemblDB, PubChem
Leachianone A	P03372	0.87	LPEPZZAVFJPLNZ-SFHVURJKSA-N	7.24	BindingDB, ChemblDB, PubChem
Leachianone A	Q92731	0.87	LPEPZZAVFJPLNZ-SFHVURJKSA-N	7.17	PubChem, ChemblDB, BindingDB
Leachianone A	P31639	0.968	LTTQKYMNTNISSZ-KESSSICBSA-N	5.77	BindingDB, ChemblDB, PubChem
Leachianone A	P31639	0.897	XRYVAQQLDYTHCL-CMJOXMDJSA-N	5.39	BindingDB, ChemblDB, PubChem
Luteolin 7-*O*-neohesperidoside	P61088	0.91	DFPMSGMNTNDNHN-ZFOFJSCHSA-N	5.08	PubChem
Luteolin 7-*O*-neohesperidoside	P61088	0.91	DFPMSGMNTNDNHN-ZHNJBYEHSA-N	5.12	PubChem
Luteolin 7-*O*-neohesperidoside	P11712	0.9	IKGXIBQEEMLURG-BKUODXTLSA-N	A	DrugBank
Luteolin 7-*O*-neohesperidoside	P10632	0.9	IKGXIBQEEMLURG-BKUODXTLSA-N	A	DrugBank
Luteolin 7-*O*-neohesperidoside	P10635	0.9	IKGXIBQEEMLURG-BKUODXTLSA-N	A	DrugBank
Luteolin 7-*O*-neohesperidoside	P11511	0.91	DFPMSGMNTNDNHN-ZHNJBYEHSA-N	5.3	PubChem
Luteolin 7-*O*-neohesperidoside	P11511	0.91	DFPMSGMNTNDNHN-ZPHOTFPESA-N	5.3	BindingDB, ChemblDB
Luteolin 7-*O*-neohesperidoside	P11511	0.887	SXNOCVMJOZRSLS-MCEICCLHSA-N	5.3	BindingDB
Luteolin 7-*O*-neohesperidoside	P42330	0.9	IKGXIBQEEMLURG-BKUODXTLSA-N	A	DrugBank
Luteolin 7-*O*-neohesperidoside	P03362	0.812	FBSKJMQYURKNSU-GETSLDTQSA-N	5.11	BindingDB, PubChem, ChemblDB
Luteolin 7-*O*-neohesperidoside	P17252	0.815	DTOUWTJYUCZJQD-UJERWXFOSA-N	5.72	ChemblDB, BindingDB, PubChem
Luteolin 7-*O*-neohesperidoside	P17252	0.812	FBSKJMQYURKNSU-PFSLZTKCSA-N	5.03	PubChem
Luteolin 7-*O*-neohesperidoside	P17252	0.812	FBSKJMQYURKNSU-ZLSOWSIRSA-N	5.03	ChemblDB, BindingDB
Luteolin 7-*O*-neohesperidoside	P17252	0.807	ZMYQRHSOVRDQDL-CPPDSBOHSA-N	4.72	ChemblDB, BindingDB
Luteolin 7-*O*-neohesperidoside	P17252	0.807	ZMYQRHSOVRDQDL-ZODWXZNSSA-N	4.72	PubChem
Luteolin 7-*O*-neohesperidoside	P17252	0.768	CBZYUWGJNYOKHT-VBXILIPMSA-N	4.83	PubChem
Luteolin 7-*O*-neohesperidoside	P17252	0.768	CBZYUWGJNYOKHT-ZKDZFUIGSA-N	4.83	ChemblDB, BindingDB
Luteolin 7-*O*-neohesperidoside	P08912	0.9	IKGXIBQEEMLURG-UHFFFAOYSA-N	7.45	PDSP
Luteolin 7-*O*-neohesperidoside	P08913	0.9	IKGXIBQEEMLURG-NVPNHPEKSA-N	8.05	BindingDB, PubChem, ChemblDB
Luteolin 7-*O*-neohesperidoside	P08913	0.9	IKGXIBQEEMLURG-UHFFFAOYSA-N	8.05	PDSP
Luteolin 7-*O*-neohesperidoside	P18825	0.9	IKGXIBQEEMLURG-NVPNHPEKSA-N	8.05	BindingDB, ChemblDB, PubChem
Luteolin 7-*O*-neohesperidoside	P18825	0.9	IKGXIBQEEMLURG-UHFFFAOYSA-N	6.54	PDSP

Table S3. *Cont.*

Molid	Uniprot	SIM	REF_NN	REF_pACT	SOURCE_DB
Luteolin 7-*O*-neohesperidoside	Q02410	0.848	QUQPHWDTPGMPEX-QJBIFVCTSA-N	5.02	PubChem
Luteolin 7-*O*-neohesperidoside	Q9XUB2	0.812	FBSKJMQYURKNSU-OSIOBVTQSA-N	5.01	PubChem
Luteolin-5-*O*-neohesperidoside	P61088	0.908	DFPMSGMNTNDNHN-ZFOFJSCHSA-N	5.08	PubChem
Luteolin-5-*O*-neohesperidoside	P61088	0.908	DFPMSGMNTNDNHN-ZHNJBYEHSA-N	5.12	PubChem
Luteolin-5-*O*-neohesperidoside	P11712	0.891	IKGXIBQEEMLURG-BKUODXTLSA-N	A	DrugBank
Luteolin-5-*O*-neohesperidoside	P10632	0.891	IKGXIBQEEMLURG-BKUODXTLSA-N	A	DrugBank
Luteolin-5-*O*-neohesperidoside	P10635	0.891	IKGXIBQEEMLURG-BKUODXTLSA-N	A	DrugBank
Luteolin-5-*O*-neohesperidoside	P11511	0.908	DFPMSGMNTNDNHN-ZHNJBYEHSA-N	5.3	PubChem
Luteolin-5-*O*-neohesperidoside	P11511	0.908	DFPMSGMNTNDNHN-ZPHOTFPESA-N	5.3	BindingDB, ChemblDB
Luteolin-5-*O*-neohesperidoside	P11511	0.885	SXNOCVMJOZRSLS-MCEICCLHSA-N	5.3	BindingDB
Luteolin-5-*O*-neohesperidoside	P42330	0.891	IKGXIBQEEMLURG-BKUODXTLSA-N	A	DrugBank
Luteolin-5-*O*-neohesperidoside	P03362	0.788	FBSKJMQYURKNSU-GETSLDTQSA-N	5.11	BindingDB, PubChem, ChemblDB
Luteolin-5-*O*-neohesperidoside	P17252	0.81	DTOUWTJYUCZJQD-UJERWXFOSA-N	5.72	ChemblDB, BindingDB, PubChem
Luteolin-5-*O*-neohesperidoside	P17252	0.788	FBSKJMQYURKNSU-PFSLZTKCSA-N	5.03	PubChem
Luteolin-5-*O*-neohesperidoside	P17252	0.788	FBSKJMQYURKNSU-ZLSOWSIRSA-N	5.03	ChemblDB, BindingDB
Luteolin-5-*O*-neohesperidoside	P17252	0.785	ZMYQRHSOVRDQDL-CPPDSBOHSA-N	4.72	ChemblDB, BindingDB
Luteolin-5-*O*-neohesperidoside	P17252	0.785	ZMYQRHSOVRDQDL-ZODWXZNSSA-N	4.72	PubChem
Luteolin-5-*O*-neohesperidoside	P17252	0.765	CBZYUWGJNYOKHT-VBXILIPMSA-N	4.83	PubChem
Luteolin-5-*O*-neohesperidoside	P17252	0.765	CBZYUWGJNYOKHT-ZKDZFUIGSA-N	4.83	ChemblDB, BindingDB
Luteolin-5-*O*-neohesperidoside	P08912	0.891	IKGXIBQEEMLURG-UHFFFAOYSA-N	7.45	PDSP
Luteolin-5-*O*-neohesperidoside	P08913	0.891	IKGXIBQEEMLURG-NVPNHPEKSA-N	8.05	BindingDB, PubChem, ChemblDB
Luteolin-5-*O*-neohesperidoside	P08913	0.891	IKGXIBQEEMLURG-UHFFFAOYSA-N	8.05	PDSP
Luteolin-5-*O*-neohesperidoside	P18825	0.891	IKGXIBQEEMLURG-NVPNHPEKSA-N	8.05	BindingDB, ChemblDB, PubChem
Luteolin-5-*O*-neohesperidoside	P18825	0.891	IKGXIBQEEMLURG-UHFFFAOYSA-N	6.54	PDSP
Luteolin-5-*O*-neohesperidoside	Q02410	0.863	QUQPHWDTPGMPEX-QJBIFVCTSA-N	5.02	PubChem
Luteolin-5-*O*-neohesperidoside	Q9XUB2	0.788	FBSKJMQYURKNSU-OSIOBVTQSA-N	5.01	PubChem
Kaempferol-7-*O*-α-L-arabinofuranoside	Q9UNQ0	0.813	KYQZWONCHDNPDP-QNDFHXLGSA-N	A	DrugBank
Kaempferol-7-*O*-α-L-arabinofuranoside	P05113	0.871	ISQRJFLLIDGZEP-CMWLGVBASA-N	5.85	BindingDB, PubChem
Kaempferol-7-*O*-α-L-arabinofuranoside	P15121	0.837	TXKFRRCKZWJXBW-GPRNFGOXSA-N	5.5	BindingDB, ChemblDB, PubChem
Kaempferol-7-*O*-α-L-arabinofuranoside	P15121	0.802	XXKIWCKZQFBXIR-SXFAUFNYSA-N	5.04	BindingDB, ChemblDB, PubChem
Kaempferol-7-*O*-α-L-arabinofuranoside	P15121	0.779	JBFOLLJCGUCDQP-ZFORQUDYSA-N	5.89	BindingDB, ChemblDB, PubChem
Kaempferol-7-*O*-α-L-arabinofuranoside	P05091	0.813	KYQZWONCHDNPDP-QNDFHXLGSA-N	A	DrugBank
Kaempferol-7-*O*-α-L-arabinofuranoside	Q9HAS3	0.833	ONBQEOIKXPHGMB-VBSBHUPXSA-N	5.54	BindingDB, ChemblDB, PubChem
8-Prenylapigenin	P21589	0.812	REFJWTPEDVJJIY-UHFFFAOYSA-N	7.34	ChemblDB, PubChem
8-Prenylapigenin	P08183	0.812	REFJWTPEDVJJIY-UHFFFAOYSA-N	A	DrugBank
8-Prenylapigenin	Q9UNQ0	0.812	REFJWTPEDVJJIY-UHFFFAOYSA-N	A	DrugBank

Table S3. *Cont.*

Molid	Uniprot	SIM	REF_NN	REF_pACT	SOURCE_DB
8-Prenylapigenin	P29768	0.876	IYRMWMYZSQPJKC-UHFFFAOYSA-N	5.1	BindingDB, ChemblDB, PubChem
8-Prenylapigenin	P29768	0.817	ZDOTZEDNGNPOEW-UHFFFAOYSA-N	5.85	BindingDB, ChemblDB, PubChem
8-Prenylapigenin	P29768	0.812	REFJWTPEDVJJIY-UHFFFAOYSA-N	5.29	BindingDB, ChemblDB, PubChem
8-Prenylapigenin	P29768	0.802	IQPNAANSBPBGFQ-UHFFFAOYSA-N	5.37	BindingDB, ChemblDB, PubChem
8-Prenylapigenin	P29768	0.797	GVQOVMKBYJKZSY-UHFFFAOYSA-N	4.54	ChemblDB, PubChem
8-Prenylapigenin	P04350	0.842	ZSPZNFOLWQEVQJ-UHFFFAOYSA-N	5.52	ChemblDB
8-Prenylapigenin	P29512	0.842	ZSPZNFOLWQEVQJ-UHFFFAOYSA-N	5.52	ChemblDB
8-Prenylapigenin	Q13509	0.842	ZSPZNFOLWQEVQJ-UHFFFAOYSA-N	5.52	ChemblDB
8-Prenylapigenin	Q3ZCM7	0.842	ZSPZNFOLWQEVQJ-UHFFFAOYSA-N	5.52	ChemblDB, PubChem
8-Prenylapigenin	Q9H4B7	0.842	ZSPZNFOLWQEVQJ-UHFFFAOYSA-N	5.52	ChemblDB
8-Prenylapigenin	P04798	0.918	IZQSVPBOUDKVDZ-UHFFFAOYSA-N	7.25	ChemblDB, PubChem
8-Prenylapigenin	P04798	0.876	IYRMWMYZSQPJKC-UHFFFAOYSA-N	6.2	ChemblDB, PubChem
8-Prenylapigenin	P04798	0.853	FPLMIPQZHHQWHN-UHFFFAOYSA-N	6.92	ChemblDB, PubChem
8-Prenylapigenin	P04798	0.817	SQFSKOYWJBQGKQ-UHFFFAOYSA-N	6.52	ChemblDB, BindingDB, PubChem
8-Prenylapigenin	P04798	0.813	MBNGWHIJMBWFHU-UHFFFAOYSA-N	6.85	ChemblDB, PubChem
8-Prenylapigenin	P04798	0.813	SCZVLDHREVKTSH-UHFFFAOYSA-N	7.02	ChemblDB, PubChem
8-Prenylapigenin	P04798	0.812	REFJWTPEDVJJIY-UHFFFAOYSA-N	5.92	ChemblDB, PubChem
8-Prenylapigenin	P04798	0.802	IQPNAANSBPBGFQ-UHFFFAOYSA-N	5.9	ChemblDB, PubChem
8-Prenylapigenin	P05177	0.918	IZQSVPBOUDKVDZ-UHFFFAOYSA-N	5.9	PubChem, ChemblDB
8-Prenylapigenin	P05177	0.876	IYRMWMYZSQPJKC-UHFFFAOYSA-N	6.14	PubChem, ChemblDB
8-Prenylapigenin	P05177	0.853	FPLMIPQZHHQWHN-UHFFFAOYSA-N	5.92	PubChem, ChemblDB
8-Prenylapigenin	P05177	0.817	SQFSKOYWJBQGKQ-UHFFFAOYSA-N	5.52	BindingDB, PubChem, ChemblDB
8-Prenylapigenin	P05177	0.813	MBNGWHIJMBWFHU-UHFFFAOYSA-N	5.61	PubChem, ChemblDB
8-Prenylapigenin	P05177	0.813	SCZVLDHREVKTSH-UHFFFAOYSA-N	5.95	PubChem, ChemblDB
8-Prenylapigenin	P05177	0.812	REFJWTPEDVJJIY-UHFFFAOYSA-N	5.39	PubChem, ChemblDB
8-Prenylapigenin	P05177	0.802	IQPNAANSBPBGFQ-UHFFFAOYSA-N	5.47	PubChem, ChemblDB
8-Prenylapigenin	Q16678	0.918	IZQSVPBOUDKVDZ-UHFFFAOYSA-N	7.77	ChemblDB, PubChem
8-Prenylapigenin	Q16678	0.876	IYRMWMYZSQPJKC-UHFFFAOYSA-N	7.33	ChemblDB, PubChem
8-Prenylapigenin	Q16678	0.853	FPLMIPQZHHQWHN-UHFFFAOYSA-N	7.7	ChemblDB, PubChem
8-Prenylapigenin	Q16678	0.817	SQFSKOYWJBQGKQ-UHFFFAOYSA-N	7.91	BindingDB, ChemblDB, PubChem
8-Prenylapigenin	Q16678	0.813	MBNGWHIJMBWFHU-UHFFFAOYSA-N	7.54	ChemblDB, PubChem
8-Prenylapigenin	Q16678	0.813	SCZVLDHREVKTSH-UHFFFAOYSA-N	7.7	ChemblDB, PubChem
8-Prenylapigenin	Q16678	0.812	REFJWTPEDVJJIY-UHFFFAOYSA-N	7.11	ChemblDB, PubChem
8-Prenylapigenin	Q16678	0.802	IQPNAANSBPBGFQ-UHFFFAOYSA-N	7.1	ChemblDB, PubChem
8-Prenylapigenin	P11712	0.876	IYRMWMYZSQPJKC-UHFFFAOYSA-N	5.22	BindingDB, ChemblDB, PubChem
8-Prenylapigenin	P11712	0.812	REFJWTPEDVJJIY-UHFFFAOYSA-N	4.57	BindingDB, ChemblDB, PubChem

Table S3. *Cont.*

Molid	Uniprot	SIM	REF_NN	REF_pACT	SOURCE_DB
8-Prenylapigenin	P10632	0.812	REFJWTPEDVJJIY-UHFFFAOYSA-N	A	DrugBank
8-Prenylapigenin	P10635	0.809	VJJZJBUCDWKPLC-UHFFFAOYSA-N	5.33	BindingDB, ChemblDB, PubChem
8-Prenylapigenin	P11511	0.992	PGCKDCPTJAQQSQ-UHFFFAOYSA-N	7	BindingDB, ChemblDB, PubChem
8-Prenylapigenin	P11511	0.93	NXBYIJSAISXPKJ-WEVVVXLNSA-N	5.34	BindingDB, ChemblDB, PubChem
8-Prenylapigenin	P11511	0.866	TVUGLERLRIQATC-BJMVGYQFSA-N	3.01	BindingDB, ChemblDB, PubChem
8-Prenylapigenin	P11511	0.832	KNMMNUQOUANAJS-UHFFFAOYSA-N	5.01	BindingDB, ChemblDB, PubChem
8-Prenylapigenin	P11511	0.812	REFJWTPEDVJJIY-UHFFFAOYSA-N	7.92	BindingDB, ChemblDB, PubChem
8-Prenylapigenin	P11511	0.796	KDDIWXQFRQYXCG-UHFFFAOYSA-N	4.51	BindingDB, ChemblDB, PubChem
8-Prenylapigenin	P11387	0.802	IQPNAANSBPBGFQ-UHFFFAOYSA-N	6.18	BindingDB, ChemblDB, PubChem
8-Prenylapigenin	Q91WR5	0.812	REFJWTPEDVJJIY-UHFFFAOYSA-N	5.16	ChemblDB, PubChem
8-Prenylapigenin	P15121	0.918	IZQSVPBOUDKVDZ-UHFFFAOYSA-N	3.52	BindingDB, ChemblDB, PubChem
8-Prenylapigenin	P15121	0.876	IYRMWMYZSQPJKC-UHFFFAOYSA-N	5.88	BindingDB, ChemblDB, PubChem
8-Prenylapigenin	P15121	0.858	KIGVXRGRNLQNNI-UHFFFAOYSA-N	7.55	ChemblDB
8-Prenylapigenin	P15121	0.852	RRYQDECFPVYHLR-UHFFFAOYSA-N	6.62	ChemblDB
8-Prenylapigenin	P15121	0.851	MYMGKIQXYXSRIJ-UHFFFAOYSA-N	3.54	BindingDB, ChemblDB, PubChem
8-Prenylapigenin	P15121	0.834	FPSMUVCMXQTXND-UHFFFAOYSA-N	6.55	ChemblDB
8-Prenylapigenin	P15121	0.817	QZAXKZRZMAXPSF-UHFFFAOYSA-N	7.24	ChemblDB
8-Prenylapigenin	P15121	0.815	XNXWWNNKDCMQFM-UHFFFAOYSA-N	6.92	ChemblDB
8-Prenylapigenin	P15121	0.812	REFJWTPEDVJJIY-UHFFFAOYSA-N	5.41	BindingDB, ChemblDB, PubChem
8-Prenylapigenin	P15121	0.812	SQYJGJIGIBPVLM-UHFFFAOYSA-N	6.64	ChemblDB
8-Prenylapigenin	P15121	0.802	IQPNAANSBPBGFQ-UHFFFAOYSA-N	5.02	BindingDB, ChemblDB, PubChem
8-Prenylapigenin	P15121	0.79	CGIMOCJIBRBBKV-UHFFFAOYSA-N	5.92	ChemblDB
8-Prenylapigenin	P15121	0.79	PEOZKVMXPVPVBH-UHFFFAOYSA-N	5.92	ChemblDB
8-Prenylapigenin	P15121	0.788	BBYWJFDEIJPQMI-UHFFFAOYSA-N	7.19	ChemblDB
8-Prenylapigenin	P15121	0.785	WGWGXVOAFMLMJZ-UHFFFAOYSA-N	6.09	ChemblDB
8-Prenylapigenin	P15121	0.78	QWUHUBDKQQPMQG-UHFFFAOYSA-N	6.52	ChemblDB
8-Prenylapigenin	P40925	0.812	REFJWTPEDVJJIY-UHFFFAOYSA-N	5.22	BindingDB, ChemblDB, PubChem
8-Prenylapigenin	P14061	0.876	IYRMWMYZSQPJKC-UHFFFAOYSA-N	5.98	BindingDB, ChemblDB, PubChem
8-Prenylapigenin	P37059	0.876	IYRMWMYZSQPJKC-UHFFFAOYSA-N	6.44	BindingDB, ChemblDB, PubChem
8-Prenylapigenin	P37059	0.812	REFJWTPEDVJJIY-UHFFFAOYSA-N	5.81	BindingDB, ChemblDB, PubChem
8-Prenylapigenin	P49327	0.812	REFJWTPEDVJJIY-UHFFFAOYSA-N	5.26	ChemblDB, PubChem, BindingDB
8-Prenylapigenin	P49327	0.803	YXOLAZRVSSWPPT-UHFFFAOYSA-N	5.1	ChemblDB, BindingDB
8-Prenylapigenin	P49327	0.802	IQPNAANSBPBGFQ-UHFFFAOYSA-N	5.13	ChemblDB, PubChem,BindingDB
8-Prenylapigenin	P49327	0.782	XHEFDIBZLJXQHF-UHFFFAOYSA-N	5.7	ChemblDB, BindingDB
8-Prenylapigenin	Q965D6	0.918	IZQSVPBOUDKVDZ-UHFFFAOYSA-N	5.08	BindingDB, ChemblDB
8-Prenylapigenin	Q965D6	0.876	IYRMWMYZSQPJKC-UHFFFAOYSA-N	5.4	BindingDB, ChemblDB

Table S3. *Cont.*

Molid	Uniprot	SIM	REF_NN	REF_pACT	SOURCE_DB
8-Prenylapigenin	Q965D6	0.812	REFJWTPEDVJJIY-UHFFFAOYSA-N	5.27	BindingDB, ChemblDB
8-Prenylapigenin	Q965D6	0.803	YXOLAZRVSSWPPT-UHFFFAOYSA-N	5.64	BindingDB, ChemblDB
8-Prenylapigenin	Q965D6	0.802	IQPNAANSBPBGFQ-UHFFFAOYSA-N	5.75	BindingDB, ChemblDB
8-Prenylapigenin	Q965D6	0.782	XHEFDIBZLJXQHF-UHFFFAOYSA-N	5.39	BindingDB, ChemblDB
8-Prenylapigenin	Q965D5	0.918	IZQSVPBOUDKVDZ-UHFFFAOYSA-N	5.3	BindingDB, ChemblDB
8-Prenylapigenin	Q965D5	0.876	IYRMWMYZSQPJKC-UHFFFAOYSA-N	4.7	BindingDB, ChemblDB
8-Prenylapigenin	Q965D5	0.812	REFJWTPEDVJJIY-UHFFFAOYSA-N	5.93	BindingDB, ChemblDB
8-Prenylapigenin	Q965D5	0.803	YXOLAZRVSSWPPT-UHFFFAOYSA-N	5.3	BindingDB, ChemblDB
8-Prenylapigenin	Q965D5	0.802	IQPNAANSBPBGFQ-UHFFFAOYSA-N	5.68	BindingDB, ChemblDB
8-Prenylapigenin	Q965D5	0.782	XHEFDIBZLJXQHF-UHFFFAOYSA-N	6	BindingDB, ChemblDB
8-Prenylapigenin	P21397	0.812	REFJWTPEDVJJIY-UHFFFAOYSA-N	8	PubChem, ChemblDB, BindingDB
8-Prenylapigenin	P21397	0.802	IQPNAANSBPBGFQ-UHFFFAOYSA-N	5.31	PubChem, ChemblDB, BindingDB
8-Prenylapigenin	Q06327	0.802	IQPNAANSBPBGFQ-UHFFFAOYSA-N	5.5	BindingDB, ChemblDB, PubChem
8-Prenylapigenin	P18054	0.812	REFJWTPEDVJJIY-UHFFFAOYSA-N	6.36	BindingDB, ChemblDB, PubChem
8-Prenylapigenin	P18054	0.782	XHEFDIBZLJXQHF-UHFFFAOYSA-N	6.02	BindingDB, ChemblDB, PubChem
8-Prenylapigenin	P16050	0.876	IYRMWMYZSQPJKC-UHFFFAOYSA-N	5.66	BindingDB, ChemblDB, PubChem
8-Prenylapigenin	P16050	0.812	REFJWTPEDVJJIY-UHFFFAOYSA-N	6.02	BindingDB, ChemblDB, PubChem
8-Prenylapigenin	P16050	0.802	IQPNAANSBPBGFQ-UHFFFAOYSA-N	5.49	BindingDB
8-Prenylapigenin	P16050	0.782	XHEFDIBZLJXQHF-UHFFFAOYSA-N	5.85	BindingDB, ChemblDB, PubChem
8-Prenylapigenin	P09917	0.876	IYRMWMYZSQPJKC-UHFFFAOYSA-N	5.57	PubChem, BindingDB, ChemblDB
8-Prenylapigenin	P09917	0.814	WZAVERXTFSLUQU-UHFFFAOYSA-N	5.3	PubChem, BindingDB, ChemblDB
8-Prenylapigenin	P09917	0.812	REFJWTPEDVJJIY-UHFFFAOYSA-N	6.17	PubChem, BindingDB, ChemblDB
8-Prenylapigenin	P09917	0.808	GWTRYHLDEZQBFT-UHFFFAOYSA-N	5.16	PubChem, BindingDB, ChemblDB
8-Prenylapigenin	P09917	0.808	JHIYXUBQIYJRKN-UHFFFAOYSA-N	6.05	PubChem, BindingDB, ChemblDB
8-Prenylapigenin	P09917	0.802	BSSFSJXTDIXIRW-UHFFFAOYSA-N	6	PubChem, BindingDB, ChemblDB
8-Prenylapigenin	P09917	0.776	QLNVCPCSZYFVQY-UHFFFAOYSA-N	5.4	PubChem, BindingDB, ChemblDB
8-Prenylapigenin	P47989	0.918	IZQSVPBOUDKVDZ-UHFFFAOYSA-N	5.6	BindingDB, ChemblDB, PubChem
8-Prenylapigenin	P47989	0.876	IYRMWMYZSQPJKC-UHFFFAOYSA-N	5.97	BindingDB, ChemblDB, PubChem
8-Prenylapigenin	P47989	0.858	KIGVXRGRNLQNNI-UHFFFAOYSA-N	4.44	BindingDB, ChemblDB, PubChem
8-Prenylapigenin	P47989	0.837	WEPBGSIAWZTEJR-UHFFFAOYSA-N	4.5	BindingDB, ChemblDB, PubChem
8-Prenylapigenin	P47989	0.813	SCZVLDHREVKTSH-UHFFFAOYSA-N	4.85	BindingDB, ChemblDB, PubChem
8-Prenylapigenin	P47989	0.812	REFJWTPEDVJJIY-UHFFFAOYSA-N	5.55	BindingDB, ChemblDB, PubChem
8-Prenylapigenin	P47989	0.806	BWORNNDZQGOKBY-UHFFFAOYSA-N	5.11	BindingDB, ChemblDB, PubChem
8-Prenylapigenin	P47989	0.803	YXOLAZRVSSWPPT-UHFFFAOYSA-N	5	BindingDB, ChemblDB, PubChem
8-Prenylapigenin	P47989	0.802	IQPNAANSBPBGFQ-UHFFFAOYSA-N	5.72	BindingDB, ChemblDB, PubChem
8-Prenylapigenin	P47989	0.782	XHEFDIBZLJXQHF-UHFFFAOYSA-N	5.36	BindingDB, ChemblDB, PubChem

Table S3. *Cont.*

Molid	Uniprot	SIM	REF_NN	REF_pACT	SOURCE_DB
8-Prenylapigenin	Q6NUS8	0.876	IYRMWMYZSQPJKC-UHFFFAOYSA-N	A	DrugBank
8-Prenylapigenin	Q6NUS8	0.812	REFJWTPEDVJJIY-UHFFFAOYSA-N	A	DrugBank
8-Prenylapigenin	P08263	0.813	SCZVLDHREVKTSH-UHFFFAOYSA-N	5.76	PubChem
8-Prenylapigenin	O00329	0.812	REFJWTPEDVJJIY-UHFFFAOYSA-N	5.42	ChemblDB
8-Prenylapigenin	P42336	0.812	REFJWTPEDVJJIY-UHFFFAOYSA-N	5.42	ChemblDB
8-Prenylapigenin	P42338	0.812	REFJWTPEDVJJIY-UHFFFAOYSA-N	5.42	ChemblDB
8-Prenylapigenin	P48736	0.812	REFJWTPEDVJJIY-UHFFFAOYSA-N	5.42	ChemblDB, BindingDB, DrugBank
8-Prenylapigenin	P08631	0.812	REFJWTPEDVJJIY-UHFFFAOYSA-N	A	DrugBank
8-Prenylapigenin	P68400	0.812	REFJWTPEDVJJIY-UHFFFAOYSA-N	5.99	BindingDB, ChemblDB, PubChem
8-Prenylapigenin	P11309	0.876	IYRMWMYZSQPJKC-UHFFFAOYSA-N	5.89	BindingDB
8-Prenylapigenin	P11309	0.812	REFJWTPEDVJJIY-UHFFFAOYSA-N	7.51	BindingDB, ChemblDB, PubChem, DrugBank, MOAD
8-Prenylapigenin	P11309	0.81	DNISTMYBAOCXPD-UHFFFAOYSA-N	5.08	BindingDB
8-Prenylapigenin	P11309	0.803	YXOLAZRVSSWPPT-UHFFFAOYSA-N	5.57	BindingDB
8-Prenylapigenin	P11309	0.802	IQPNAANSBPBGFQ-UHFFFAOYSA-N	5.8	BindingDB
8-Prenylapigenin	P11309	0.782	XHEFDIBZLJXQHF-UHFFFAOYSA-N	6.07	BindingDB
8-Prenylapigenin	O94768	0.812	REFJWTPEDVJJIY-UHFFFAOYSA-N	A	DrugBank
8-Prenylapigenin	P49841	0.876	IYRMWMYZSQPJKC-UHFFFAOYSA-N	5.2	PubChem, BindingDB, ChemblDB
8-Prenylapigenin	P49841	0.812	REFJWTPEDVJJIY-UHFFFAOYSA-N	5.34	PubChem, BindingDB, ChemblDB
8-Prenylapigenin	P49841	0.802	IQPNAANSBPBGFQ-UHFFFAOYSA-N	5.76	PubChem, BindingDB, ChemblDB
8-Prenylapigenin	P49841	0.782	XHEFDIBZLJXQHF-UHFFFAOYSA-N	6.38	BindingDB, ChemblDB
8-Prenylapigenin	P49840	0.876	IYRMWMYZSQPJKC-UHFFFAOYSA-N	5.46	ChemblDB, PubChem
8-Prenylapigenin	P49840	0.812	REFJWTPEDVJJIY-UHFFFAOYSA-N	5.68	ChemblDB, PubChem
8-Prenylapigenin	P49840	0.802	IQPNAANSBPBGFQ-UHFFFAOYSA-N	6.1	ChemblDB, PubChem
8-Prenylapigenin	P49840	0.782	XHEFDIBZLJXQHF-UHFFFAOYSA-N	6.38	ChemblDB, PubChem
8-Prenylapigenin	P04054	0.812	REFJWTPEDVJJIY-UHFFFAOYSA-N	5.7	ChemblDB, PubChem
8-Prenylapigenin	Q6NVY1	0.812	REFJWTPEDVJJIY-UHFFFAOYSA-N	A	DrugBank
8-Prenylapigenin	P09923	0.802	NHFGEHLUROYMEB-UHFFFAOYSA-N	5.66	PubChem
8-Prenylapigenin	P27815	0.882	VMLJAWUWVVHRNG-UHFFFAOYSA-N	5.59	ChemblDB
8-Prenylapigenin	Q07343	0.882	VMLJAWUWVVHRNG-UHFFFAOYSA-N	5.59	ChemblDB, PubChem
8-Prenylapigenin	Q08493	0.882	VMLJAWUWVVHRNG-UHFFFAOYSA-N	5.59	ChemblDB
8-Prenylapigenin	Q08499	0.882	VMLJAWUWVVHRNG-UHFFFAOYSA-N	5.59	ChemblDB
8-Prenylapigenin	Q13370	0.882	VMLJAWUWVVHRNG-UHFFFAOYSA-N	6.39	ChemblDB, PubChem
8-Prenylapigenin	Q14432	0.882	VMLJAWUWVVHRNG-UHFFFAOYSA-N	6.39	ChemblDB

Table S3. *Cont.*

Molid	Uniprot	SIM	REF_NN	REF_pACT	SOURCE_DB
8-Prenylapigenin	O76074	0.901	ZHTTWVRMGWQEOH-UHFFFAOYSA-N	6.16	BindingDB, ChemblDB, PubChem
8-Prenylapigenin	O76074	0.89	MEHHCBRCXIDGKZ-UHFFFAOYSA-N	5.89	BindingDB, ChemblDB, PubChem
8-Prenylapigenin	O76074	0.882	VMLJAWUWVVHRNG-UHFFFAOYSA-N	8.3	BindingDB, ChemblDB, PubChem
8-Prenylapigenin	O76074	0.866	TUUXBSASAQJECY-UHFFFAOYSA-N	5.66	BindingDB, ChemblDB, PubChem
8-Prenylapigenin	Q8I2J3	0.812	REFJWTPEDVJJIY-UHFFFAOYSA-N	6.41	PubChem
8-Prenylapigenin	P10520	0.799	UCHYSPNEUSDFQR-UHFFFAOYSA-N	5.73	PubChem
8-Prenylapigenin	P06576	0.812	REFJWTPEDVJJIY-UHFFFAOYSA-N	A	DrugBank
8-Prenylapigenin	P25705	0.812	REFJWTPEDVJJIY-UHFFFAOYSA-N	A	DrugBank
8-Prenylapigenin	P36542	0.812	REFJWTPEDVJJIY-UHFFFAOYSA-N	A	DrugBank
8-Prenylapigenin	P21439	0.876	IYRMWMYZSQPJKC-UHFFFAOYSA-N	5.17	BindingDB, ChemblDB, PubChem
8-Prenylapigenin	P21439	0.817	SQFSKOYWJBQGKQ-UHFFFAOYSA-N	5.32	BindingDB, ChemblDB, PubChem
8-Prenylapigenin	P21439	0.795	ARXMHGNBODSKBL-UHFFFAOYSA-N	5.17	BindingDB, ChemblDB, PubChem
8-Prenylapigenin	P21439	0.795	JZGNQOUGTXYCBJ-UHFFFAOYSA-N	5.97	BindingDB, ChemblDB, PubChem
8-Prenylapigenin	O43570	0.812	REFJWTPEDVJJIY-UHFFFAOYSA-N	5.03	ChemblDB, PubChem, BindingDB
8-Prenylapigenin	P00915	0.812	REFJWTPEDVJJIY-UHFFFAOYSA-N	5.57	ChemblDB, PubChem, BindingDB
8-Prenylapigenin	P00918	0.812	REFJWTPEDVJJIY-UHFFFAOYSA-N	5.59	ChemblDB, BindingDB, PubChem
8-Prenylapigenin	P07451	0.812	REFJWTPEDVJJIY-UHFFFAOYSA-N	5.09	ChemblDB, BindingDB, PubChem
8-Prenylapigenin	P22748	0.812	REFJWTPEDVJJIY-UHFFFAOYSA-N	5.1	ChemblDB, BindingDB, PubChem
8-Prenylapigenin	P23280	0.812	REFJWTPEDVJJIY-UHFFFAOYSA-N	5.21	ChemblDB, BindingDB, PubChem
8-Prenylapigenin	P35218	0.812	REFJWTPEDVJJIY-UHFFFAOYSA-N	5.17	ChemblDB, BindingDB, PubChem
8-Prenylapigenin	P43166	0.812	REFJWTPEDVJJIY-UHFFFAOYSA-N	5.32	ChemblDB, BindingDB, PubChem
8-Prenylapigenin	Q16790	0.812	REFJWTPEDVJJIY-UHFFFAOYSA-N	5.16	ChemblDB, PubChem, BindingDB
8-Prenylapigenin	Q8N1Q1	0.812	REFJWTPEDVJJIY-UHFFFAOYSA-N	5.04	ChemblDB, BindingDB, PubChem
8-Prenylapigenin	Q9ULX7	0.812	REFJWTPEDVJJIY-UHFFFAOYSA-N	5.27	ChemblDB, BindingDB, PubChem
8-Prenylapigenin	P25910	0.812	REFJWTPEDVJJIY-UHFFFAOYSA-N	5.4	BindingDB, ChemblDB, PubChem
8-Prenylapigenin	P21917	0.812	REFJWTPEDVJJIY-UHFFFAOYSA-N	6.61	ChemblDB, PDSP, BindingDB, PubChem
8-Prenylapigenin	P30542	0.812	REFJWTPEDVJJIY-UHFFFAOYSA-N	5.61	ChemblDB, PubChem, BindingDB
8-Prenylapigenin	P30542	0.803	YXOLAZRVSSWPPT-UHFFFAOYSA-N	4.86	ChemblDB, PubChem, BindingDB
8-Prenylapigenin	P30542	0.802	IQPNAANSBPBGFQ-UHFFFAOYSA-N	5.79	ChemblDB, PubChem
8-Prenylapigenin	P03372	0.79	KVKRLMGFFGPZFA-UHFFFAOYSA-N	5.9	BindingDB, ChemblDB, PubChem
8-Prenylapigenin	P03372	0.789	LQBURCXFHFUJJI-UHFFFAOYSA-N	4.8	BindingDB, ChemblDB, PubChem
8-Prenylapigenin	P03372	0.783	JWLBHKJXDMACKX-UHFFFAOYSA-N	5.21	BindingDB
8-Prenylapigenin	P03372	0.768	MYAHOBWCBIQQLV-UHFFFAOYSA-N	7.37	BindingDB, ChemblDB, PubChem
8-Prenylapigenin	P03372	0.758	HHVMWRVANSDUKF-UHFFFAOYSA-N	7.51	BindingDB, ChemblDB, PubChem
8-Prenylapigenin	Q92731	0.79	KVKRLMGFFGPZFA-UHFFFAOYSA-N	6.46	PubChem, ChemblDB, BindingDB
8-Prenylapigenin	Q92731	0.789	LQBURCXFHFUJJI-UHFFFAOYSA-N	5.86	PubChem, ChemblDB, BindingDB

Table S3. *Cont.*

Molid	Uniprot	SIM	REF_NN	REF_pACT	SOURCE_DB
8-Prenylapigenin	Q92731	0.783	JWLBHKJXDMACKX-UHFFFAOYSA-N	6.41	BindingDB
8-Prenylapigenin	Q92731	0.758	HHVMWRVANSDUKF-UHFFFAOYSA-N	8.62	PubChem, ChemblDB, BindingDB
8-Prenylapigenin	P10275	0.876	IYRMWMYZSQPJKC-UHFFFAOYSA-N	5.01	BindingDB, ChemblDB, PubChem
8-Prenylapigenin	P53985	0.812	REFJWTPEDVJJIY-UHFFFAOYSA-N	A	DrugBank
8-Prenylapigenin	O60669	0.812	REFJWTPEDVJJIY-UHFFFAOYSA-N	A	DrugBank
8-Prenylapigenin	P33527	0.876	IYRMWMYZSQPJKC-UHFFFAOYSA-N	5.62	BindingDB, ChemblDB, PubChem
8-Prenylapigenin	P33527	0.813	MBNGWHIJMBWFHU-UHFFFAOYSA-N	5.52	BindingDB, ChemblDB, PubChem
8-Prenylapigenin	P33527	0.813	SCZVLDHREVKTSH-UHFFFAOYSA-N	5.52	BindingDB, ChemblDB, PubChem
8-Prenylapigenin	P33527	0.812	REFJWTPEDVJJIY-UHFFFAOYSA-N	5.62	BindingDB, ChemblDB, PubChem, DrugBank
8-Prenylapigenin	P35869	0.876	IYRMWMYZSQPJKC-UHFFFAOYSA-N	7.55	PubChem, ChemblDB
8-Prenylapigenin	Q88N29	0.812	REFJWTPEDVJJIY-UHFFFAOYSA-N	5.58	BindingDB
8-Prenylapigenin	Q88N29	0.802	IQPNAANSBPBGFQ-UHFFFAOYSA-N	5.14	BindingDB
8-Prenylapigenin	P49418	0.812	REFJWTPEDVJJIY-UHFFFAOYSA-N	5.4	PubChem, ChemblDB
8-Prenylapigenin	O94956	0.812	REFJWTPEDVJJIY-UHFFFAOYSA-N	A	DrugBank
8-Prenylapigenin	O00459	0.812	REFJWTPEDVJJIY-UHFFFAOYSA-N	5.42	ChemblDB, PubChem
8-Prenylapigenin	P67870	0.812	REFJWTPEDVJJIY-UHFFFAOYSA-N	5.73	ChemblDB, PubChem
8-Prenylapigenin	Q07820	0.803	YXOLAZRVSSWPPT-UHFFFAOYSA-N	5.52	PubChem
8-Prenylapigenin	P27986	0.812	REFJWTPEDVJJIY-UHFFFAOYSA-N	5.42	ChemblDB, PubChem
8-Prenylapigenin	P02766	0.842	BRPKBUNFOZFULQ-SGAXSIHGSA-N	A	DrugBank
8-Prenylapigenin	Q01043	0.782	XHEFDIBZLJXQHF-UHFFFAOYSA-N	A	DrugBank
8-Prenylapigenin	Q9AIU0	0.812	REFJWTPEDVJJIY-UHFFFAOYSA-N	A	DrugBank
Apigenine	P21589	0.887	REFJWTPEDVJJIY-UHFFFAOYSA-N	7.34	ChemblDB, PubChem
Apigenine	P36888	0.775	ZKIUEEGLQKCTHN-UHFFFAOYSA-N	6.57	BindingDB, ChemblDB, PubChem
Apigenine	P09619	0.775	ZKIUEEGLQKCTHN-UHFFFAOYSA-N	6.7	BindingDB, ChemblDB, PubChem
Apigenine	P08183	0.907	YQHMWTPYORBCMF-ZZXKWVIFSA-N	5.32	ChemblDB
Apigenine	P08183	0.887	REFJWTPEDVJJIY-UHFFFAOYSA-N	A	DrugBank
Apigenine	P08183	0.876	TZBJGXHYKVUXJN-UHFFFAOYSA-N	A	DrugBank
Apigenine	P08183	0.774	CKQUMNKKOYTAST-UHFFFAOYSA-N	4.28	PubChem, ChemblDB
Apigenine	Q9UNQ0	0.887	REFJWTPEDVJJIY-UHFFFAOYSA-N	A	DrugBank
Apigenine	Q9UNQ0	0.876	TZBJGXHYKVUXJN-UHFFFAOYSA-N	A	DrugBank
Apigenine	P29768	COLL	IYRMWMYZSQPJKC-UHFFFAOYSA-N	5.1	BindingDB, ChemblDB, PubChem
Apigenine	P29768	0.939	IQPNAANSBPBGFQ-UHFFFAOYSA-N	5.37	BindingDB, ChemblDB, PubChem
Apigenine	P29768	0.898	ZDOTZEDNGNPOEW-UHFFFAOYSA-N	5.85	BindingDB, ChemblDB, PubChem
Apigenine	P29768	0.89	KZNIFHPLKGYRTM-UHFFFAOYSA-N	4.76	BindingDB, ChemblDB, PubChem
Apigenine	P29768	0.887	REFJWTPEDVJJIY-UHFFFAOYSA-N	5.29	BindingDB, ChemblDB, PubChem

Table S3. *Cont.*

Molid	Uniprot	SIM	REF_NN	REF_pACT	SOURCE_DB
Apigenine	Q15788	0.876	TZBJGXHYKVUXJN-UHFFFAOYSA-N	A	DrugBank
Apigenine	Q15596	0.876	TZBJGXHYKVUXJN-UHFFFAOYSA-N	A	DrugBank
Apigenine	P04350	0.813	ZSPZNFOLWQEVQJ-UHFFFAOYSA-N	5.52	ChemblDB
Apigenine	P29512	0.813	ZSPZNFOLWQEVQJ-UHFFFAOYSA-N	5.52	ChemblDB
Apigenine	Q13509	0.813	ZSPZNFOLWQEVQJ-UHFFFAOYSA-N	5.52	ChemblDB
Apigenine	Q3ZCM7	0.813	ZSPZNFOLWQEVQJ-UHFFFAOYSA-N	5.52	ChemblDB, PubChem
Apigenine	Q9H4B7	0.813	ZSPZNFOLWQEVQJ-UHFFFAOYSA-N	5.52	ChemblDB
Apigenine	P04798	COLL	IYRMWMYZSQPJKC-UHFFFAOYSA-N	6.2	ChemblDB, PubChem
Apigenine	P04798	0.939	IQPNAANSBPBGFQ-UHFFFAOYSA-N	5.9	ChemblDB, PubChem
Apigenine	P04798	0.916	FPLMIPQZHHQWHN-UHFFFAOYSA-N	6.92	ChemblDB, PubChem
Apigenine	P04798	0.916	IZQSVPBOUDKVDZ-UHFFFAOYSA-N	7.25	ChemblDB, PubChem
Apigenine	P04798	0.89	KZNIFHPLKGYRTM-UHFFFAOYSA-N	6.37	ChemblDB, PubChem
Apigenine	P04798	0.89	VCCRNZQBSJXYJD-UHFFFAOYSA-N	7.11	ChemblDB, PubChem
Apigenine	P04798	0.887	REFJWTPEDVJJIY-UHFFFAOYSA-N	5.92	ChemblDB, PubChem
Apigenine	P04798	0.844	SQFSKOYWJBQGKQ-UHFFFAOYSA-N	6.52	ChemblDB, BindingDB, PubChem
Apigenine	P04798	0.84	MBNGWHIJMBWFHU-UHFFFAOYSA-N	6.85	ChemblDB, PubChem
Apigenine	P04798	0.84	SCZVLDHREVKTSH-UHFFFAOYSA-N	7.02	ChemblDB, PubChem
Apigenine	P04798	0.818	PDHAOJSHSJQANO-OWOJBTEDSA-N	4.82	ChemblDB, BindingDB, PubChem
Apigenine	P04798	0.79	SBHXYTNGIZCORC-ZDUSSCGKSA-N	4.96	ChemblDB, PubChem
Apigenine	P05177	COLL	IYRMWMYZSQPJKC-UHFFFAOYSA-N	6.14	PubChem, ChemblDB
Apigenine	P05177	0.939	IQPNAANSBPBGFQ-UHFFFAOYSA-N	5.47	PubChem, ChemblDB
Apigenine	P05177	0.916	FPLMIPQZHHQWHN-UHFFFAOYSA-N	5.92	PubChem, ChemblDB
Apigenine	P05177	0.916	IZQSVPBOUDKVDZ-UHFFFAOYSA-N	5.9	PubChem, ChemblDB
Apigenine	P05177	0.89	KZNIFHPLKGYRTM-UHFFFAOYSA-N	6.1	PubChem, ChemblDB
Apigenine	P05177	0.89	VCCRNZQBSJXYJD-UHFFFAOYSA-N	7.4	PubChem, ChemblDB
Apigenine	P05177	0.887	REFJWTPEDVJJIY-UHFFFAOYSA-N	5.39	PubChem, ChemblDB
Apigenine	P05177	0.876	TZBJGXHYKVUXJN-UHFFFAOYSA-N	A	DrugBank
Apigenine	P05177	0.844	SQFSKOYWJBQGKQ-UHFFFAOYSA-N	5.52	BindingDB, PubChem, ChemblDB
Apigenine	P05177	0.84	MBNGWHIJMBWFHU-UHFFFAOYSA-N	5.61	PubChem, ChemblDB
Apigenine	P05177	0.84	SCZVLDHREVKTSH-UHFFFAOYSA-N	5.95	PubChem, ChemblDB
Apigenine	P05177	0.818	PDHAOJSHSJQANO-OWOJBTEDSA-N	4.82	BindingDB, PubChem, ChemblDB
Apigenine	P05177	0.79	SBHXYTNGIZCORC-ZDUSSCGKSA-N	4.27	PubChem, ChemblDB
Apigenine	Q16678	COLL	IYRMWMYZSQPJKC-UHFFFAOYSA-N	7.33	ChemblDB, PubChem
Apigenine	Q16678	0.939	IQPNAANSBPBGFQ-UHFFFAOYSA-N	7.1	ChemblDB, PubChem
Apigenine	Q16678	0.916	FPLMIPQZHHQWHN-UHFFFAOYSA-N	7.7	ChemblDB, PubChem
Apigenine	Q16678	0.916	IZQSVPBOUDKVDZ-UHFFFAOYSA-N	7.77	ChemblDB, PubChem

Table S3. *Cont.*

Molid	Uniprot	SIM	REF_NN	REF_pACT	SOURCE_DB
Apigenine	Q16678	0.89	KZNIFHPLKGYRTM-UHFFFAOYSA-N	7.6	ChemblDB, PubChem
Apigenine	Q16678	0.89	VCCRNZQBSJXYJD-UHFFFAOYSA-N	7.6	ChemblDB, PubChem
Apigenine	Q16678	0.887	REFJWTPEDVJJIY-UHFFFAOYSA-N	7.11	ChemblDB, PubChem
Apigenine	Q16678	0.844	SQFSKOYWJBQGKQ-UHFFFAOYSA-N	7.91	BindingDB, ChemblDB, PubChem
Apigenine	Q16678	0.84	MBNGWHIJMBWFHU-UHFFFAOYSA-N	7.54	ChemblDB, PubChem
Apigenine	Q16678	0.84	SCZVLDHREVKTSH-UHFFFAOYSA-N	7.7	ChemblDB, PubChem
Apigenine	Q16678	0.818	PDHAOJSHSJQANO-OWOJBTEDSA-N	4.47	BindingDB, ChemblDB, PubChem
Apigenine	Q16678	0.79	SBHXYTNGIZCORC-ZDUSSCGKSA-N	5.89	ChemblDB, PubChem
Apigenine	P11712	COLL	IYRMWMYZSQPJKC-UHFFFAOYSA-N	5.22	BindingDB, ChemblDB, PubChem
Apigenine	P11712	0.887	REFJWTPEDVJJIY-UHFFFAOYSA-N	4.57	BindingDB, ChemblDB, PubChem
Apigenine	P10632	0.887	REFJWTPEDVJJIY-UHFFFAOYSA-N	A	DrugBank
Apigenine	P10635	0.861	VJJZJBUCDWKPLC-UHFFFAOYSA-N	5.33	BindingDB, ChemblDB, PubChem
Apigenine	P11511	0.89	KZNIFHPLKGYRTM-UHFFFAOYSA-N	6.03	BindingDB, ChemblDB, PubChem
Apigenine	P11511	0.89	VCCRNZQBSJXYJD-UHFFFAOYSA-N	4	BindingDB, ChemblDB, PubChem
Apigenine	P11511	0.887	REFJWTPEDVJJIY-UHFFFAOYSA-N	7.92	BindingDB, ChemblDB, PubChem
Apigenine	P11511	0.876	TZBJGXHYKVUXJN-UHFFFAOYSA-N	3.96	BindingDB, ChemblDB, PubChem
Apigenine	P11511	0.809	PGCKDCPTJAQQSQ-UHFFFAOYSA-N	7	BindingDB, ChemblDB, PubChem
Apigenine	O14746	0.898	ARYCMKPCDNHQCL-UHFFFAOYSA-N	6.7	PubChem, BindingDB, ChemblDB
Apigenine	O14746	0.898	XRQUEUISMRSNHB-UHFFFAOYSA-N	5.44	PubChem, BindingDB, ChemblDB
Apigenine	O14746	0.856	GHPOEPBXSFQHEL-UHFFFAOYSA-N	6.1	PubChem, BindingDB, ChemblDB
Apigenine	O14746	0.856	YINCNTBPLYVMFY-UHFFFAOYSA-N	6.22	PubChem, BindingDB, ChemblDB
Apigenine	O14746	0.823	NXBASGHOYZCCJS-UHFFFAOYSA-N	6.09	PubChem, BindingDB, ChemblDB
Apigenine	O14746	0.815	PVFGJHYLIHMCQD-UHFFFAOYSA-N	3.9	PubChem, BindingDB, ChemblDB
Apigenine	O14746	0.81	QTLGVNKQQSZQNC-UHFFFAOYSA-N	6.89	PubChem, BindingDB, ChemblDB
Apigenine	O14746	0.809	HJCIUNMVLYBADX-UHFFFAOYSA-N	5.52	PubChem, BindingDB, ChemblDB
Apigenine	O14746	0.809	ZJZSQGDOCUHCCW-UHFFFAOYSA-N	4.3	PubChem, BindingDB, ChemblDB
Apigenine	O14746	0.803	JLIZJXVGQAXZGF-UHFFFAOYSA-N	3.9	PubChem, BindingDB, ChemblDB
Apigenine	P11387	0.939	IQPNAANSBPBGFQ-UHFFFAOYSA-N	6.18	BindingDB, ChemblDB, PubChem
Apigenine	P11387	0.91	JEYUVFHPIMHRPG-XCVCLJGOSA-N	5.52	PubChem
Apigenine	P11387	0.815	BOWHITWXJNIGIM-VZUCSPMQSA-N	5.22	PubChem
Apigenine	Q91WR5	0.887	REFJWTPEDVJJIY-UHFFFAOYSA-N	5.16	ChemblDB, PubChem
Apigenine	P15121	COLL	IYRMWMYZSQPJKC-UHFFFAOYSA-N	5.88	BindingDB, ChemblDB, PubChem
Apigenine	P15121	0.939	IQPNAANSBPBGFQ-UHFFFAOYSA-N	5.02	BindingDB, ChemblDB, PubChem
Apigenine	P15121	0.916	IZQSVPBOUDKVDZ-UHFFFAOYSA-N	3.52	BindingDB, ChemblDB, PubChem
Apigenine	P15121	0.89	KZNIFHPLKGYRTM-UHFFFAOYSA-N	5.18	BindingDB, ChemblDB, PubChem
Apigenine	P15121	0.887	REFJWTPEDVJJIY-UHFFFAOYSA-N	5.41	BindingDB, ChemblDB, PubChem

Table S3. *Cont.*

Molid	Uniprot	SIM	REF_NN	REF_pACT	SOURCE_DB
Apigenine	P15121	0.863	FPSMUVCMXQTXND-UHFFFAOYSA-N	6.55	ChemblDB
Apigenine	P15121	0.862	YBJAJJDAKYYNDB-UHFFFAOYSA-N	5.64	ChemblDB
Apigenine	P15121	0.858	SQYJGJIGIBPVLM-UHFFFAOYSA-N	6.64	ChemblDB
Apigenine	P15121	0.848	PEOZKVMXPVPVBH-UHFFFAOYSA-N	5.92	ChemblDB
Apigenine	P15121	0.845	VYAKIUWQLHRZGK-UHFFFAOYSA-N	6.69	ChemblDB
Apigenine	P15121	0.815	PVFGJHYLIHMCQD-UHFFFAOYSA-N	5.19	BindingDB, ChemblDB, PubChem
Apigenine	P15121	0.814	RRYQDECFPVYHLR-UHFFFAOYSA-N	6.62	ChemblDB
Apigenine	P15121	0.812	MYMGKIQXYXSRIJ-UHFFFAOYSA-N	3.54	BindingDB, ChemblDB, PubChem
Apigenine	P15121	0.785	SYGUVOLSUJYPPS-UHFFFAOYSA-N	6.39	ChemblDB
Apigenine	P40925	0.887	REFJWTPEDVJJIY-UHFFFAOYSA-N	5.22	BindingDB, ChemblDB, PubChem
Apigenine	P14061	COLL	IYRMWMYZSQPJKC-UHFFFAOYSA-N	5.98	BindingDB, ChemblDB, PubChem
Apigenine	P14061	0.89	KZNIFHPLKGYRTM-UHFFFAOYSA-N	6.15	BindingDB, ChemblDB, PubChem
Apigenine	P14061	0.876	TZBJGXHYKVUXJN-UHFFFAOYSA-N	5.66	BindingDB, ChemblDB, PubChem
Apigenine	P37059	COLL	IYRMWMYZSQPJKC-UHFFFAOYSA-N	6.44	BindingDB, ChemblDB, PubChem
Apigenine	P37059	0.89	KZNIFHPLKGYRTM-UHFFFAOYSA-N	4.2	BindingDB, ChemblDB, PubChem
Apigenine	P37059	0.887	REFJWTPEDVJJIY-UHFFFAOYSA-N	5.81	BindingDB, ChemblDB, PubChem
Apigenine	P37059	0.876	TZBJGXHYKVUXJN-UHFFFAOYSA-N	4.78	BindingDB, ChemblDB, PubChem
Apigenine	P49327	0.944	XHEFDIBZLJXQHF-UHFFFAOYSA-N	5.7	ChemblDB, BindingDB
Apigenine	P49327	0.939	IQPNAANSBPBGFQ-UHFFFAOYSA-N	5.13	ChemblDB, PubChem, BindingDB
Apigenine	P49327	0.912	AYMYWHCQALZEGT-ORCRQEGFSA-N	4.52	ChemblDB, PubChem
Apigenine	P49327	0.887	REFJWTPEDVJJIY-UHFFFAOYSA-N	5.26	ChemblDB, PubChem, BindingDB
Apigenine	P49327	0.876	TZBJGXHYKVUXJN-UHFFFAOYSA-N	5.16	ChemblDB, BindingDB
Apigenine	P49327	0.876	YXOLAZRVSSWPPT-UHFFFAOYSA-N	5.1	ChemblDB, BindingDB
Apigenine	Q965D6	COLL	IYRMWMYZSQPJKC-UHFFFAOYSA-N	5.4	BindingDB, ChemblDB
Apigenine	Q965D6	0.944	XHEFDIBZLJXQHF-UHFFFAOYSA-N	5.39	BindingDB, ChemblDB
Apigenine	Q965D6	0.939	IQPNAANSBPBGFQ-UHFFFAOYSA-N	5.75	BindingDB, ChemblDB
Apigenine	Q965D6	0.916	IZQSVPBOUDKVDZ-UHFFFAOYSA-N	5.08	BindingDB, ChemblDB
Apigenine	Q965D6	0.89	VCCRNZQBSJXYJD-UHFFFAOYSA-N	4	BindingDB, ChemblDB
Apigenine	Q965D6	0.887	REFJWTPEDVJJIY-UHFFFAOYSA-N	5.27	BindingDB, ChemblDB
Apigenine	Q965D6	0.876	YXOLAZRVSSWPPT-UHFFFAOYSA-N	5.64	BindingDB, ChemblDB
Apigenine	P21397	0.939	IQPNAANSBPBGFQ-UHFFFAOYSA-N	5.31	PubChem, ChemblDB, BindingDB
Apigenine	P21397	0.91	MPXAWSABMVLIBU-UHFFFAOYSA-N	4.89	PubChem, ChemblDB, BindingDB
Apigenine	P21397	0.89	KZNIFHPLKGYRTM-UHFFFAOYSA-N	5.77	PubChem, ChemblDB, BindingDB
Apigenine	P21397	0.888	ZHTQCPCDXKMMLU-UHFFFAOYSA-N	4.6	PubChem, ChemblDB, BindingDB
Apigenine	P21397	0.887	REFJWTPEDVJJIY-UHFFFAOYSA-N	8	PubChem, ChemblDB, BindingDB
Apigenine	P21397	0.886	RVOUOPDWADMVBA-UHFFFAOYSA-N	4.62	PubChem, ChemblDB, BindingDB

Table S3. *Cont.*

Molid	Uniprot	SIM	REF_NN	REF_pACT	SOURCE_DB
Apigenine	P21397	0.876	TZBJGXHYKVUXJN-UHFFFAOYSA-N	6.05	ChemblDB
Apigenine	P21397	0.829	JJUNZBRHHGLJQW-UHFFFAOYSA-N	5.1	PubChem, ChemblDB, BindingDB
Apigenine	P21397	0.824	XESIWQIMUSNPRO-UHFFFAOYSA-N	5.42	PubChem, ChemblDB, BindingDB
Apigenine	P21397	0.79	JAOZFCHZESUBKS-UHFFFAOYSA-N	5.57	PubChem, ChemblDB, BindingDB
Apigenine	Q06327	0.939	IQPNAANSBPBGFQ-UHFFFAOYSA-N	5.5	BindingDB, ChemblDB, PubChem
Apigenine	P18054	0.944	XHEFDIBZLJXQHF-UHFFFAOYSA-N	6.02	BindingDB, ChemblDB, PubChem
Apigenine	P18054	0.887	REFJWTPEDVJJIY-UHFFFAOYSA-N	6.36	BindingDB, ChemblDB, PubChem
Apigenine	P18054	0.815	GYLUFQJZYAJQDI-UHFFFAOYSA-N	5.06	BindingDB, ChemblDB, PubChem
Apigenine	P18054	0.761	FXNFHKRTJBSTCS-UHFFFAOYSA-N	6.95	BindingDB, ChemblDB, PubChem
Apigenine	P16050	COLL	IYRMWMYZSQPJKC-UHFFFAOYSA-N	5.66	BindingDB, ChemblDB, PubChem
Apigenine	P16050	0.944	XHEFDIBZLJXQHF-UHFFFAOYSA-N	5.85	BindingDB, ChemblDB, PubChem
Apigenine	P16050	0.939	IQPNAANSBPBGFQ-UHFFFAOYSA-N	5.49	BindingDB
Apigenine	P16050	0.887	REFJWTPEDVJJIY-UHFFFAOYSA-N	6.02	BindingDB, ChemblDB, PubChem
Apigenine	P16050	0.815	GYLUFQJZYAJQDI-UHFFFAOYSA-N	4.31	BindingDB, ChemblDB, PubChem
Apigenine	P16050	0.761	FXNFHKRTJBSTCS-UHFFFAOYSA-N	5.04	BindingDB, ChemblDB, PubChem
Apigenine	P09917	COLL	IYRMWMYZSQPJKC-UHFFFAOYSA-N	5.57	PubChem, BindingDB, ChemblDB
Apigenine	P09917	0.912	AYMYWHCQALZEGT-ORCRQEGFSA-N	8.34	PubChem, BindingDB, ChemblDB
Apigenine	P09917	0.907	YQHMWTPYORBCMF-ZZXKWVIFSA-N	4	PubChem, BindingDB, ChemblDB
Apigenine	P09917	0.896	PFOGPGPNRKORCH-DAFODLJHSA-N	7.19	PubChem, BindingDB, ChemblDB
Apigenine	P09917	0.887	REFJWTPEDVJJIY-UHFFFAOYSA-N	6.17	PubChem, BindingDB, ChemblDB
Apigenine	P09917	0.853	DXDRHHKMWQZJHT-FPYGCLRLSA-N	4.46	PubChem, BindingDB, ChemblDB
Apigenine	P09917	0.761	FXNFHKRTJBSTCS-UHFFFAOYSA-N	4.5	PubChem, BindingDB, ChemblDB
Apigenine	P47989	COLL	IYRMWMYZSQPJKC-UHFFFAOYSA-N	5.97	BindingDB, ChemblDB, PubChem
Apigenine	P47989	0.944	XHEFDIBZLJXQHF-UHFFFAOYSA-N	5.36	BindingDB, ChemblDB, PubChem
Apigenine	P47989	0.939	IQPNAANSBPBGFQ-UHFFFAOYSA-N	5.72	BindingDB, ChemblDB, PubChem
Apigenine	P47989	0.916	IZQSVPBOUDKVDZ-UHFFFAOYSA-N	5.6	BindingDB, ChemblDB, PubChem
Apigenine	P47989	0.907	JVXZRQGOGOXCEC-UHFFFAOYSA-N	4.9	BindingDB, ChemblDB, PubChem
Apigenine	P47989	0.89	KZNIFHPLKGYRTM-UHFFFAOYSA-N	6.16	BindingDB, ChemblDB, PubChem
Apigenine	P47989	0.89	VCCRNZQBSJXYJD-UHFFFAOYSA-N	5.75	BindingDB, ChemblDB, PubChem
Apigenine	P47989	0.887	REFJWTPEDVJJIY-UHFFFAOYSA-N	5.55	BindingDB, ChemblDB, PubChem
Apigenine	P47989	0.882	WEPBGSIAWZTEJR-UHFFFAOYSA-N	4.5	BindingDB, ChemblDB, PubChem
Apigenine	P47989	0.876	TZBJGXHYKVUXJN-UHFFFAOYSA-N	3	BindingDB, ChemblDB, PubChem
Apigenine	P47989	0.876	YXOLAZRVSSWPPT-UHFFFAOYSA-N	5	BindingDB, ChemblDB, PubChem
Apigenine	P47989	0.868	SOEDEYVDCDYMMH-UHFFFAOYSA-N	5.37	BindingDB, ChemblDB, PubChem
Apigenine	P47989	0.84	SCZVLDHREVKTSH-UHFFFAOYSA-N	4.85	BindingDB, ChemblDB, PubChem
Apigenine	P47989	0.815	DDKGKOOLFLYZDL-UHFFFAOYSA-N	4.18	BindingDB, ChemblDB, PubChem

Table S3. *Cont.*

Molid	Uniprot	SIM	REF_NN	REF_pACT	SOURCE_DB
Apigenine	P47989	0.815	GYLUFQJZYAJQDI-UHFFFAOYSA-N	2.5	BindingDB, ChemblDB, PubChem
Apigenine	P47989	0.809	BMZFZTMWBCFKSS-UHFFFAOYSA-N	3	BindingDB, ChemblDB, PubChem
Apigenine	P47989	0.804	DDNPCXHBFYJXBJ-UHFFFAOYSA-N	7.52	BindingDB, ChemblDB, PubChem
Apigenine	P47989	0.782	PADQINQHPQKXNL-LSDHHAIUSA-N	4.03	BindingDB, ChemblDB, PubChem
Apigenine	P47989	0.761	FXNFHKRTJBSTCS-UHFFFAOYSA-N	5.52	BindingDB, ChemblDB, PubChem
Apigenine	Q6NUS8	COLL	IYRMWMYZSQPJKC-UHFFFAOYSA-N	A	DrugBank
Apigenine	Q6NUS8	0.887	REFJWTPEDVJJIY-UHFFFAOYSA-N	A	DrugBank
Apigenine	P08263	0.84	SCZVLDHREVKTSH-UHFFFAOYSA-N	5.76	PubChem
Apigenine	O00329	0.887	REFJWTPEDVJJIY-UHFFFAOYSA-N	5.42	ChemblDB
Apigenine	P42336	0.887	REFJWTPEDVJJIY-UHFFFAOYSA-N	5.42	ChemblDB
Apigenine	P42336	0.784	BMFNJUDIGQHUBL-WCSRMQSCSA-N	5.17	BindingDB, ChemblDB, PubChem
Apigenine	P42338	0.887	REFJWTPEDVJJIY-UHFFFAOYSA-N	5.42	ChemblDB
Apigenine	P48736	0.887	REFJWTPEDVJJIY-UHFFFAOYSA-N	5.42	ChemblDB, BindingDB, DrugBank
Apigenine	Q14289	0.876	TZBJGXHYKVUXJN-UHFFFAOYSA-N	A	DrugBank
Apigenine	P08631	0.887	REFJWTPEDVJJIY-UHFFFAOYSA-N	A	DrugBank
Apigenine	P68400	0.89	KZNIFHPLKGYRTM-UHFFFAOYSA-N	5.88	BindingDB, ChemblDB, PubChem
Apigenine	P68400	0.887	REFJWTPEDVJJIY-UHFFFAOYSA-N	5.99	BindingDB, ChemblDB, PubChem
Apigenine	P11309	COLL	IYRMWMYZSQPJKC-UHFFFAOYSA-N	5.89	BindingDB
Apigenine	P11309	0.986	DNISTMYBAOCXPD-UHFFFAOYSA-N	5.08	BindingDB
Apigenine	P11309	0.944	XHEFDIBZLJXQHF-UHFFFAOYSA-N	6.07	BindingDB
Apigenine	P11309	0.939	IQPNAANSBPBGFQ-UHFFFAOYSA-N	5.8	BindingDB
Apigenine	P11309	0.91	CCCIGFPBADVTFE-UHFFFAOYSA-N	5.11	BindingDB
Apigenine	P11309	0.89	KZNIFHPLKGYRTM-UHFFFAOYSA-N	6.03	BindingDB
Apigenine	P11309	0.89	OBWHQJYOOCRPST-UHFFFAOYSA-N	6.01	BindingDB
Apigenine	P11309	0.887	REFJWTPEDVJJIY-UHFFFAOYSA-N	7.51	BindingDB, ChemblDB, PubChem, DrugBank, MOAD
Apigenine	P11309	0.876	YXOLAZRVSSWPPT-UHFFFAOYSA-N	5.57	BindingDB
Apigenine	P11309	0.861	ARSRJFRKVXALTF-UHFFFAOYSA-N	6.19	BindingDB, DrugBank
Apigenine	P11309	0.809	HJCIUNMVLYBADX-UHFFFAOYSA-N	4.66	BindingDB
Apigenine	O94768	0.887	REFJWTPEDVJJIY-UHFFFAOYSA-N	A	DrugBank
Apigenine	P42345	0.784	BMFNJUDIGQHUBL-WCSRMQSCSA-N	5.04	BindingDB, ChemblDB, PubChem
Apigenine	P49841	COLL	IYRMWMYZSQPJKC-UHFFFAOYSA-N	5.2	PubChem, BindingDB, ChemblDB
Apigenine	P49841	0.944	XHEFDIBZLJXQHF-UHFFFAOYSA-N	6.38	BindingDB, ChemblDB
Apigenine	P49841	0.939	IQPNAANSBPBGFQ-UHFFFAOYSA-N	5.76	PubChem, BindingDB, ChemblDB
Apigenine	P49841	0.89	KZNIFHPLKGYRTM-UHFFFAOYSA-N	5.85	PubChem, BindingDB, ChemblDB
Apigenine	P49841	0.887	REFJWTPEDVJJIY-UHFFFAOYSA-N	5.34	PubChem, BindingDB, ChemblDB

Table S3. *Cont.*

Molid	Uniprot	SIM	REF_NN	REF_pACT	SOURCE_DB
Apigenine	P49840	COLL	IYRMWMYZSQPJKC-UHFFFAOYSA-N	5.46	ChemblDB, PubChem
Apigenine	P49840	0.944	XHEFDIBZLJXQHF-UHFFFAOYSA-N	6.38	ChemblDB, PubChem
Apigenine	P49840	0.939	IQPNAANSBPBGFQ-UHFFFAOYSA-N	6.1	ChemblDB, PubChem
Apigenine	P49840	0.89	KZNIFHPLKGYRTM-UHFFFAOYSA-N	5.85	ChemblDB, PubChem
Apigenine	P49840	0.887	REFJWTPEDVJJIY-UHFFFAOYSA-N	5.68	ChemblDB, PubChem
Apigenine	P04054	0.887	REFJWTPEDVJJIY-UHFFFAOYSA-N	5.7	ChemblDB, PubChem
Apigenine	Q6NVY1	0.887	REFJWTPEDVJJIY-UHFFFAOYSA-N	A	DrugBank
Apigenine	P09923	0.939	NHFGEHLUROYMEB-UHFFFAOYSA-N	5.66	PubChem
Apigenine	P09923	0.862	SPZXXUUDYMHBSG-UHFFFAOYSA-N	4.12	PubChem
Apigenine	P09923	0.815	RGNXWPVNPFAADO-NSIKDUERSA-N	4.45	PubChem
Apigenine	P09923	0.79	SBHXYTNGIZCORC-UHFFFAOYSA-N	4.29	PubChem
Apigenine	P14410	0.761	FXNFHKRTJBSTCS-UHFFFAOYSA-N	3.82	PubChem, BindingDB, ChemblDB
Apigenine	P14410	0.761	OBLILGZHQIPPOD-UHFFFAOYSA-N	8.36	PubChem, ChemblDB
Apigenine	P08236	0.952	GSSOWCUOWLMMRJ-UHFFFAOYSA-N	5.23	PubChem, BindingDB, ChemblDB
Apigenine	P08236	0.89	KZNIFHPLKGYRTM-UHFFFAOYSA-N	5.55	PubChem, BindingDB, ChemblDB
Apigenine	P08236	0.862	YBJAJJDAKYYNDB-UHFFFAOYSA-N	4.86	PubChem, BindingDB, ChemblDB
Apigenine	P08236	0.862	ZCTNPCRBEWXCGP-UHFFFAOYSA-N	4.02	PubChem, BindingDB, ChemblDB
Apigenine	P08236	0.861	JNJLRXUXPJHPCK-UHFFFAOYSA-N	4.02	PubChem, BindingDB, ChemblDB
Apigenine	Q8I2J3	0.887	REFJWTPEDVJJIY-UHFFFAOYSA-N	6.41	PubChem
Apigenine	P06576	0.887	REFJWTPEDVJJIY-UHFFFAOYSA-N	A	DrugBank
Apigenine	P06576	0.818	CDRPUGZCRXZLFL-OWOJBTEDSA-N	A	DrugBank
Apigenine	P25705	0.887	REFJWTPEDVJJIY-UHFFFAOYSA-N	A	DrugBank
Apigenine	P25705	0.818	CDRPUGZCRXZLFL-OWOJBTEDSA-N	A	DrugBank
Apigenine	P36542	0.887	REFJWTPEDVJJIY-UHFFFAOYSA-N	A	DrugBank
Apigenine	P36542	0.818	CDRPUGZCRXZLFL-OWOJBTEDSA-N	A	DrugBank
Apigenine	P21439	COLL	IYRMWMYZSQPJKC-UHFFFAOYSA-N	5.17	BindingDB, ChemblDB, PubChem
Apigenine	P21439	0.907	YQHMWTPYORBCMF-ZZXKWVIFSA-N	5.32	ChemblDB, PubChem
Apigenine	P21439	0.89	VCCRNZQBSJXYJD-UHFFFAOYSA-N	5.23	BindingDB, ChemblDB, PubChem
Apigenine	P21439	0.844	ARXMHGNBODSKBL-UHFFFAOYSA-N	5.17	BindingDB, ChemblDB, PubChem
Apigenine	P21439	0.844	JZGNQOUGTXYCBJ-UHFFFAOYSA-N	5.97	BindingDB, ChemblDB, PubChem
Apigenine	P21439	0.844	SQFSKOYWJBQGKQ-UHFFFAOYSA-N	5.32	BindingDB, ChemblDB, PubChem
Apigenine	P21439	0.804	SKWMTRHMWQXILY-UHFFFAOYSA-N	5.4	BindingDB, ChemblDB, PubChem
Apigenine	O43570	0.887	REFJWTPEDVJJIY-UHFFFAOYSA-N	5.03	ChemblDB, PubChem, BindingDB
Apigenine	P00915	0.887	REFJWTPEDVJJIY-UHFFFAOYSA-N	5.57	ChemblDB, PubChem, BindingDB
Apigenine	P00918	0.887	REFJWTPEDVJJIY-UHFFFAOYSA-N	5.59	ChemblDB, BindingDB, PubChem
Apigenine	P07451	0.887	REFJWTPEDVJJIY-UHFFFAOYSA-N	5.09	ChemblDB, BindingDB, PubChem

Table S3. *Cont.*

Molid	Uniprot	SIM	REF_NN	REF_pACT	SOURCE_DB
Apigenine	P22748	0.887	REFJWTPEDVJJIY-UHFFFAOYSA-N	5.1	ChemblDB, BindingDB, PubChem
Apigenine	P23280	0.887	REFJWTPEDVJJIY-UHFFFAOYSA-N	5.21	ChemblDB, BindingDB, PubChem
Apigenine	P35218	0.887	REFJWTPEDVJJIY-UHFFFAOYSA-N	5.17	ChemblDB, BindingDB, PubChem
Apigenine	P43166	0.887	REFJWTPEDVJJIY-UHFFFAOYSA-N	5.32	ChemblDB, BindingDB, PubChem
Apigenine	Q16790	0.887	REFJWTPEDVJJIY-UHFFFAOYSA-N	5.16	ChemblDB, PubChem, BindingDB
Apigenine	Q8N1Q1	0.887	REFJWTPEDVJJIY-UHFFFAOYSA-N	5.04	ChemblDB, BindingDB, PubChem
Apigenine	Q9ULX7	0.887	REFJWTPEDVJJIY-UHFFFAOYSA-N	5.27	ChemblDB, BindingDB, PubChem
Apigenine	P14174	0.815	DDKGKOOLFLYZDL-UHFFFAOYSA-N	7.42	BindingDB, ChemblDB, PubChem
Apigenine	P25910	0.887	REFJWTPEDVJJIY-UHFFFAOYSA-N	5.4	BindingDB, ChemblDB, PubChem
Apigenine	P21917	0.887	REFJWTPEDVJJIY-UHFFFAOYSA-N	6.61	ChemblDB, PDSP, BindingDB, PubChem
Apigenine	P30542	0.939	IQPNAANSBPBGFQ-UHFFFAOYSA-N	5.79	ChemblDB, PubChem
Apigenine	P30542	0.89	KZNIFHPLKGYRTM-UHFFFAOYSA-N	5.52	ChemblDB, PubChem, BindingDB
Apigenine	P30542	0.89	VCCRNZQBSJXYJD-UHFFFAOYSA-N	6.1	ChemblDB, PubChem,BindingDB, IUPHARdb
Apigenine	P30542	0.887	REFJWTPEDVJJIY-UHFFFAOYSA-N	5.61	ChemblDB, PubChem, BindingDB
Apigenine	P30542	0.876	TZBJGXHYKVUXJN-UHFFFAOYSA-N	5.3	ChemblDB, PubChem, BindingDB
Apigenine	P30542	0.876	YXOLAZRVSSWPPT-UHFFFAOYSA-N	4.86	ChemblDB, PubChem, BindingDB
Apigenine	P30542	0.826	IHFBPDAQLQOCBX-UHFFFAOYSA-N	5.79	ChemblDB, PubChem, BindingDB
Apigenine	P30542	0.824	WBSZQWVYIZRNQG-UHFFFAOYSA-N	5.79	BindingDB
Apigenine	P29274	0.89	KZNIFHPLKGYRTM-UHFFFAOYSA-N	5.12	BindingDB, ChemblDB, PubChem
Apigenine	P29274	0.89	VCCRNZQBSJXYJD-UHFFFAOYSA-N	5.38	BindingDB, ChemblDB, PubChem, IUPHARdb
Apigenine	P29274	0.887	REFJWTPEDVJJIY-UHFFFAOYSA-N	5.16	BindingDB, ChemblDB, PubChem
Apigenine	P29274	0.876	TZBJGXHYKVUXJN-UHFFFAOYSA-N	4.44	BindingDB, ChemblDB, PubChem
Apigenine	P29274	0.876	YXOLAZRVSSWPPT-UHFFFAOYSA-N	4.76	ChemblDB
Apigenine	P29274	0.826	IHFBPDAQLQOCBX-UHFFFAOYSA-N	5.19	BindingDB, ChemblDB, PubChem
Apigenine	P29275	0.89	VCCRNZQBSJXYJD-UHFFFAOYSA-N	6.01	ChemblDB, PubChem
Apigenine	P29275	0.876	YXOLAZRVSSWPPT-UHFFFAOYSA-N	4.76	ChemblDB, PubChem
Apigenine	P47869	0.907	JVXZRQGOGOXCEC-UHFFFAOYSA-N	4.92	ChemblDB
Apigenine	P47869	0.89	KZNIFHPLKGYRTM-UHFFFAOYSA-N	5.52	ChemblDB
Apigenine	P47869	0.826	IHFBPDAQLQOCBX-UHFFFAOYSA-N	6	ChemblDB
Apigenine	P47869	0.761	FXNFHKRTJBSTCS-UHFFFAOYSA-N	5.25	ChemblDB
Apigenine	P34903	0.907	JVXZRQGOGOXCEC-UHFFFAOYSA-N	4.92	ChemblDB
Apigenine	P34903	0.89	KZNIFHPLKGYRTM-UHFFFAOYSA-N	5.52	ChemblDB
Apigenine	P34903	0.826	IHFBPDAQLQOCBX-UHFFFAOYSA-N	6	ChemblDB

Table S3. *Cont.*

Molid	Uniprot	SIM	REF_NN	REF_pACT	SOURCE_DB
Apigenine	P34903	0.761	FXNFHKRTJBSTCS-UHFFFAOYSA-N	5.25	ChemblDB
Apigenine	P48169	0.907	JVXZRQGOGOXCEC-UHFFFAOYSA-N	4.92	ChemblDB
Apigenine	P48169	0.89	KZNIFHPLKGYRTM-UHFFFAOYSA-N	5.52	ChemblDB
Apigenine	P48169	0.826	IHFBPDAQLQOCBX-UHFFFAOYSA-N	6	ChemblDB
Apigenine	P48169	0.761	FXNFHKRTJBSTCS-UHFFFAOYSA-N	5.25	ChemblDB
Apigenine	P31644	0.907	JVXZRQGOGOXCEC-UHFFFAOYSA-N	4.92	ChemblDB
Apigenine	P31644	0.89	KZNIFHPLKGYRTM-UHFFFAOYSA-N	5.52	ChemblDB
Apigenine	P31644	0.826	IHFBPDAQLQOCBX-UHFFFAOYSA-N	6	ChemblDB
Apigenine	P31644	0.761	FXNFHKRTJBSTCS-UHFFFAOYSA-N	5.25	ChemblDB
Apigenine	Q16445	0.907	JVXZRQGOGOXCEC-UHFFFAOYSA-N	4.92	ChemblDB
Apigenine	Q16445	0.89	KZNIFHPLKGYRTM-UHFFFAOYSA-N	5.52	ChemblDB
Apigenine	Q16445	0.826	IHFBPDAQLQOCBX-UHFFFAOYSA-N	6	ChemblDB
Apigenine	Q16445	0.761	FXNFHKRTJBSTCS-UHFFFAOYSA-N	5.25	ChemblDB
Apigenine	P18505	0.89	KZNIFHPLKGYRTM-UHFFFAOYSA-N	6.11	ChemblDB
Apigenine	P47870	0.89	KZNIFHPLKGYRTM-UHFFFAOYSA-N	6.11	ChemblDB
Apigenine	P28472	0.89	KZNIFHPLKGYRTM-UHFFFAOYSA-N	6.11	ChemblDB
Apigenine	O14764	0.89	KZNIFHPLKGYRTM-UHFFFAOYSA-N	6.11	ChemblDB
Apigenine	P78334	0.89	KZNIFHPLKGYRTM-UHFFFAOYSA-N	6.11	ChemblDB
Apigenine	Q8N1C3	0.89	KZNIFHPLKGYRTM-UHFFFAOYSA-N	6.11	ChemblDB
Apigenine	P18507	0.89	KZNIFHPLKGYRTM-UHFFFAOYSA-N	6.11	ChemblDB
Apigenine	Q99928	0.89	KZNIFHPLKGYRTM-UHFFFAOYSA-N	6.11	ChemblDB
Apigenine	O00591	0.89	KZNIFHPLKGYRTM-UHFFFAOYSA-N	6.11	ChemblDB
Apigenine	Q9UN88	0.89	KZNIFHPLKGYRTM-UHFFFAOYSA-N	6.11	ChemblDB
Apigenine	P34021	0.876	TZBJGXHYKVUXJN-UHFFFAOYSA-N	7.6	NRacl
Apigenine	P03372	0.944	LQBURCXFHFUJJI-UHFFFAOYSA-N	4.8	BindingDB, ChemblDB, PubChem
Apigenine	P03372	0.89	KZNIFHPLKGYRTM-UHFFFAOYSA-N	6.04	BindingDB, ChemblDB, PubChem
Apigenine	P03372	0.876	RPDMOSIZAIBSRV-UHFFFAOYSA-N	4.25	BindingDB, ChemblDB
Apigenine	P03372	0.876	TZBJGXHYKVUXJN-UHFFFAOYSA-N	6.17	NRacl, BindingDB, ChemblDB, PubChem, DrugBank, MOAD
Apigenine	P03372	0.861	BBBAWACESCACAP-UHFFFAOYSA-N	7.55	BindingDB, ChemblDB, PubChem
Apigenine	P03372	0.861	KELRLBNDSOSKDN-UHFFFAOYSA-N	5.53	BindingDB, ChemblDB, PubChem
Apigenine	P03372	0.861	RTSQVGYVPGSBHR-UHFFFAOYSA-N	7.17	BindingDB, ChemblDB, PubChem
Apigenine	P03372	0.855	OCCRBNSOQOLSKV-UHFFFAOYSA-N	5.3	PubChem
Apigenine	P03372	0.855	SDPJKYOUDICJGH-UHFFFAOYSA-N	4.8	BindingDB, ChemblDB, PubChem
Apigenine	P03372	0.855	VXADQLYVEUPXAG-UHFFFAOYSA-N	4.3	BindingDB, ChemblDB
Apigenine	P03372	0.848	MYAHOBWCBIQQLV-UHFFFAOYSA-N	7.37	BindingDB, ChemblDB, PubChem
Apigenine	P03372	0.847	KVKRLMGFFGPZFA-UHFFFAOYSA-N	5.9	BindingDB, ChemblDB, PubChem

Table S3. *Cont.*

Molid	Uniprot	SIM	REF_NN	REF_pACT	SOURCE_DB
Apigenine	P03372	0.831	ZNOIKAWHNVXFEW-UHFFFAOYSA-N	4.25	PubChem
Apigenine	P03372	0.828	JWLBHKJXDMACKX-UHFFFAOYSA-N	5.21	BindingDB
Apigenine	P03372	0.826	HHVMWRVANSDUKF-UHFFFAOYSA-N	7.51	BindingDB, ChemblDB, PubChem
Apigenine	P03372	0.822	YUTJBHHXIAWLOW-MDWZMJQESA-N	5.29	PubChem
Apigenine	P03372	0.81	HSEJJVQDYADLME-VLGSPTGOSA-N	4.3	BindingDB, ChemblDB
Apigenine	P03372	0.791	OIGXKFMCUAECHX-UHFFFAOYSA-N	5.3	PubChem
Apigenine	P03372	0.788	VSEIMGCATUFLSE-UHFFFAOYSA-N	4.99	BindingDB, ChemblDB, PubChem
Apigenine	P03372	0.766	OGCUEJPUMKMXND-UHFFFAOYSA-N	4.81	BindingDB, ChemblDB, PubChem
Apigenine	P03372	0.766	UMIQKPGMIZNJPF-UHFFFAOYSA-N	5.58	BindingDB, ChemblDB
Apigenine	P03372	0.766	VIZPQPBPYLOQMH-UHFFFAOYSA-N	4.96	BindingDB, ChemblDB, PubChem
Apigenine	Q92731	0.944	LQBURCXFHFUJJI-UHFFFAOYSA-N	5.86	PubChem, ChemblDB, BindingDB
Apigenine	Q92731	0.89	KZNIFHPLKGYRTM-UHFFFAOYSA-N	6.18	PubChem, ChemblDB, BindingDB
Apigenine	Q92731	0.876	RPDMOSIZAIBSRV-UHFFFAOYSA-N	5.87	ChemblDB, BindingDB
Apigenine	Q92731	0.876	TZBJGXHYKVUXJN-UHFFFAOYSA-N	7.72	NRacl, PubChem, ChemblDB, BindingDB, DrugBank, MOAD, IUPHARdb
Apigenine	Q92731	0.861	BBBAWACESCACAP-UHFFFAOYSA-N	8.77	PubChem, ChemblDB, BindingDB
Apigenine	Q92731	0.861	KELRLBNDSOSKDN-UHFFFAOYSA-N	7.38	PubChem, ChemblDB, BindingDB
Apigenine	Q92731	0.861	RTSQVGYVPGSBHR-UHFFFAOYSA-N	8.36	PubChem, ChemblDB, BindingDB
Apigenine	Q92731	0.855	OCCRBNSOQOLSKV-UHFFFAOYSA-N	5.58	PubChem
Apigenine	Q92731	0.855	SDPJKYOUDICJGH-UHFFFAOYSA-N	5.94	PubChem, ChemblDB, BindingDB
Apigenine	Q92731	0.855	VXADQLYVEUPXAG-UHFFFAOYSA-N	5.58	ChemblDB, BindingDB
Apigenine	Q92731	0.847	KVKRLMGFFGPZFA-UHFFFAOYSA-N	6.46	PubChem, ChemblDB, BindingDB
Apigenine	Q92731	0.831	ZNOIKAWHNVXFEW-UHFFFAOYSA-N	5.91	PubChem
Apigenine	Q92731	0.828	JWLBHKJXDMACKX-UHFFFAOYSA-N	6.41	BindingDB
Apigenine	Q92731	0.826	HHVMWRVANSDUKF-UHFFFAOYSA-N	8.62	PubChem, ChemblDB, BindingDB
Apigenine	Q92731	0.822	YUTJBHHXIAWLOW-MDWZMJQESA-N	6.02	PubChem
Apigenine	Q92731	0.81	HSEJJVQDYADLME-VLGSPTGOSA-N	5.57	ChemblDB, BindingDB
Apigenine	Q92731	0.791	OIGXKFMCUAECHX-UHFFFAOYSA-N	5.57	PubChem
Apigenine	Q92731	0.788	VSEIMGCATUFLSE-UHFFFAOYSA-N	6.06	PubChem, ChemblDB, BindingDB
Apigenine	Q92731	0.766	OGCUEJPUMKMXND-UHFFFAOYSA-N	5.08	PubChem, ChemblDB, BindingDB
Apigenine	Q92731	0.766	UMIQKPGMIZNJPF-UHFFFAOYSA-N	7.33	ChemblDB, BindingDB
Apigenine	Q92731	0.766	VIZPQPBPYLOQMH-UHFFFAOYSA-N	6.94	PubChem, ChemblDB, BindingDB
Apigenine	P11474	0.876	TZBJGXHYKVUXJN-UHFFFAOYSA-N	8	BindingDB, ChemblDB, PubChem
Apigenine	O95718	0.876	TZBJGXHYKVUXJN-UHFFFAOYSA-N	6.4	BindingDB, ChemblDB, PubChem
Apigenine	P10275	COLL	IYRMWMYZSQPJKC-UHFFFAOYSA-N	5.01	BindingDB, ChemblDB, PubChem

Table S3. *Cont.*

Molid	Uniprot	SIM	REF_NN	REF_pACT	SOURCE_DB
Apigenine	P10275	0.89	KZNIFHPLKGYRTM-UHFFFAOYSA-N	5.28	BindingDB, ChemblDB, PubChem
Apigenine	P10275	0.876	TZBJGXHYKVUXJN-UHFFFAOYSA-N	4.07	BindingDB, ChemblDB, PubChem
Apigenine	P53985	0.887	REFJWTPEDVJJIY-UHFFFAOYSA-N	A	DrugBank
Apigenine	O60669	0.887	REFJWTPEDVJJIY-UHFFFAOYSA-N	A	DrugBank
Apigenine	P33527	COLL	IYRMWMYZSQPJKC-UHFFFAOYSA-N	5.62	BindingDB, ChemblDB, PubChem
Apigenine	P33527	0.89	KZNIFHPLKGYRTM-UHFFFAOYSA-N	5.62	BindingDB, ChemblDB, PubChem
Apigenine	P33527	0.887	REFJWTPEDVJJIY-UHFFFAOYSA-N	5.62	BindingDB, ChemblDB, PubChem, DrugBank
Apigenine	P33527	0.876	TZBJGXHYKVUXJN-UHFFFAOYSA-N	A	DrugBank
Apigenine	P33527	0.868	SOEDEYVDCDYMMH-UHFFFAOYSA-N	5.3	BindingDB, ChemblDB, PubChem
Apigenine	P33527	0.84	MBNGWHIJMBWFHU-UHFFFAOYSA-N	5.52	BindingDB, ChemblDB, PubChem
Apigenine	P33527	0.84	SCZVLDHREVKTSH-UHFFFAOYSA-N	5.52	BindingDB, ChemblDB, PubChem
Apigenine	P35869	COLL	IYRMWMYZSQPJKC-UHFFFAOYSA-N	7.55	PubChem, ChemblDB
Apigenine	P35869	0.782	PADQINQHPQKXNL-UHFFFAOYSA-N	7.55	BindingDB
Apigenine	Q88N29	0.939	IQPNAANSBPBGFQ-UHFFFAOYSA-N	5.14	BindingDB
Apigenine	Q88N29	0.89	KZNIFHPLKGYRTM-UHFFFAOYSA-N	4.8	BindingDB
Apigenine	Q88N29	0.887	REFJWTPEDVJJIY-UHFFFAOYSA-N	5.58	BindingDB
Apigenine	Q88N29	0.876	TZBJGXHYKVUXJN-UHFFFAOYSA-N	4.56	BindingDB
Apigenine	P49418	0.887	REFJWTPEDVJJIY-UHFFFAOYSA-N	5.4	PubChem, ChemblDB
Apigenine	O94956	0.887	REFJWTPEDVJJIY-UHFFFAOYSA-N	A	DrugBank
Apigenine	O00459	0.887	REFJWTPEDVJJIY-UHFFFAOYSA-N	5.42	ChemblDB, PubChem
Apigenine	P67870	0.89	KZNIFHPLKGYRTM-UHFFFAOYSA-N	5.37	ChemblDB, PubChem
Apigenine	P67870	0.887	REFJWTPEDVJJIY-UHFFFAOYSA-N	5.73	ChemblDB, PubChem
Apigenine	Q07820	0.876	YXOLAZRVSSWPPT-UHFFFAOYSA-N	5.52	PubChem
Apigenine	Q07820	0.815	RGNXWPVNPFAADO-NSIKDUERSA-N	5.81	PubChem
Apigenine	P27986	0.887	REFJWTPEDVJJIY-UHFFFAOYSA-N	5.42	ChemblDB, PubChem
Apigenine	P02766	0.876	TZBJGXHYKVUXJN-UHFFFAOYSA-N	A	DrugBank
Apigenine	P02766	0.813	BRPKBUNFOZFULQ-SGAXSIHGSA-N	A	DrugBank
Apigenine	Q01043	0.944	XHEFDIBZLJXQHF-UHFFFAOYSA-N	A	DrugBank
Apigenine	Q9AIU0	0.887	REFJWTPEDVJJIY-UHFFFAOYSA-N	A	DrugBank
Kaempferol-3-*O*-α-L-rhamnopyranoside	P15121	0.944	UXXAEVMOIUAYQT-UFGFRKJLSA-N	4.72	BindingDB, ChemblDB, PubChem
Kaempferol-3-*O*-α-L-rhamnopyranoside	P15121	0.925	OXGUCUVFOIWWQJ-GPTQEAJUSA-N	5.93	ChemblDB
Kaempferol-3-*O*-α-L-rhamnopyranoside	P15121	0.925	OXGUCUVFOIWWQJ-HQBVPOQASA-N	6.87	BindingDB, ChemblDB, PubChem
Kaempferol-3-*O*-α-L-rhamnopyranoside	P15121	0.925	OXGUCUVFOIWWQJ-UHFFFAOYSA-N	5	BindingDB
Kaempferol-3-*O*-α-L-rhamnopyranoside	P15121	0.893	JPUKWEQWGBDDQB-QSOFNFLRSA-N	5.29	BindingDB, ChemblDB, PubChem
Kaempferol-3-*O*-α-L-rhamnopyranoside	P15121	0.879	YAWOKJUAVYWGTC-UHFFFAOYSA-N	4.95	BindingDB, ChemblDB, PubChem
Kaempferol-3-*O*-α-L-rhamnopyranoside	P15121	0.828	OVSQVDMCBVZWGM-QSOFNFLRSA-N	4.8	BindingDB, ChemblDB, PubChem

Table S3. *Cont.*

Molid	Uniprot	SIM	REF_NN	REF_pACT	SOURCE_DB
Kaempferol-3-*O*-α-L-rhamnopyranoside	P09923	0.828	OVSQVDMCBVZWGM-QSOFNFLRSA-N	5.28	PubChem
Kaempferol-3-*O*-α-L-rhamnopyranoside	P09923	0.764	DUBCCGAQYVUYEU-UHFFFAOYSA-N	5.49	PubChem
Kaempferol-3-O-α-L-rhamnopyranoside	O76074	0.816	NGMYNFJANBHLKA-XAPMVYOVSA-N	6.8	BindingDB, ChemblDB, PubChem
Kaempferol-3-*O*-α-L-rhamnopyranoside	O76074	0.816	PFVZUXQCELCLBL-LVKFHIPRSA-N	5.77	BindingDB, MOAD
Kaempferol-3-*O*-α-L-rhamnopyranoside	P03070	0.828	OVSQVDMCBVZWGM-UHFFFAOYSA-N	5.69	PubChem
Kaempferol-3-*O*-α-L-rhamnopyranoside	P03070	0.81	PLAPMLGJVGLZOV-YWFAZRBLSA-N	5.5	PubChem
Kaempferol-3-*O*-α-L-rhamnopyranoside	P03070	0.764	DUBCCGAQYVUYEU-UHFFFAOYSA-N	5.01	PubChem
Kaempferol-3-*O*-α-L-rhamnopyranoside	P18825	0.91	SUYDICWJCDPOPH-UHFFFAOYSA-N	4	PDSP
Kaempferol-3-*O*-α-L-rhamnopyranoside	P18825	0.828	OVSQVDMCBVZWGM-DHALZPNHSA-N	8.4	BindingDB, ChemblDB, PubChem
Kaempferol-3-*O*-α-L-rhamnopyranoside	P18825	0.828	OVSQVDMCBVZWGM-UHFFFAOYSA-N	8.4	PDSP
Kaempferol-3-*O*-α-L-rhamnopyranoside	P18825	0.815	WZCDQUUBUNNBSF-UHFFFAOYSA-N	4.7	PDSP
Kaempferol-3-*O*-α-L-rhamnopyranoside	P03372	0.758	HSWIRQIYASIOBE-JNHRPPPUSA-N	5.24	ChemblDB, PubChem
Kaempferol-3-*O*-α-L-rhamnopyranoside	P03372	0.758	HSWIRQIYASIOBE-UHFFFAOYSA-N	5.24	BindingDB
Astragalin	Q9UNQ0	0.818	KYQZWONCHDNPDP-QNDFHXLGSA-N	A	DrugBank
Astragalin	O14757	0.755	KXEGFJWWRULGOX-HSYQWOAOSA-N	5.72	ChemblDB, PubChem
Astragalin	P15121	sCOLL	JPUKWEQWGBDDQB-QSOFNFLRSA-N	5.29	BindingDB, ChemblDB, PubChem
Astragalin	P15121	0.929	OVSQVDMCBVZWGM-QSOFNFLRSA-N	4.8	BindingDB, ChemblDB, PubChem
Astragalin	P15121	0.926	TXKFRRCKZWJXBW-GPRNFGOXSA-N	5.5	BindingDB, ChemblDB, PubChem
Astragalin	P15121	0.913	OXGUCUVFOIWWQJ-GPTQEAJUSA-N	5.93	ChemblDB
Astragalin	P15121	0.913	OXGUCUVFOIWWQJ-HQBVPOQASA-N	6.87	BindingDB, ChemblDB, PubChem
Astragalin	P15121	0.913	OXGUCUVFOIWWQJ-UHFFFAOYSA-N	5	BindingDB
Astragalin	P15121	0.898	YAWOKJUAVYWGTC-UHFFFAOYSA-N	4.95	BindingDB, ChemblDB, PubChem
Astragalin	P15121	0.876	UXXAEVMOIUAYQT-UFGFRKJLSA-N	4.72	BindingDB, ChemblDB, PubChem
Astragalin	P05091	0.818	KYQZWONCHDNPDP-QNDFHXLGSA-N	A	DrugBank
Astragalin	P09923	0.929	OVSQVDMCBVZWGM-QSOFNFLRSA-N	5.28	PubChem
Astragalin	P09923	0.89	AEDDIBAIWPIIBD-UHFFFAOYSA-N	5.92	PubChem
Astragalin	P09923	0.788	DUBCCGAQYVUYEU-UHFFFAOYSA-N	5.49	PubChem
Astragalin	P10696	0.89	AEDDIBAIWPIIBD-UHFFFAOYSA-N	5.57	PubChem
Astragalin	P03070	0.929	OVSQVDMCBVZWGM-UHFFFAOYSA-N	5.69	PubChem
Astragalin	P03070	0.927	MYXNWGACZJSMBT-UHFFFAOYSA-N	4.67	PubChem
Astragalin	P03070	0.909	PLAPMLGJVGLZOV-YWFAZRBLSA-N	5.5	PubChem
Astragalin	P03070	0.9	ODBRNZZJSYPIDI-UHFFFAOYSA-N	5.28	PubChem
Astragalin	P03070	0.89	AEDDIBAIWPIIBD-UHFFFAOYSA-N	5.06	PubChem
Astragalin	P03070	0.842	HSTZMXCBWJGKHG-CUYWLFDKSA-N	4.95	PubChem
Astragalin	P03070	0.818	KYQZWONCHDNPDP-UHFFFAOYSA-N	4	PubChem
Astragalin	P03070	0.788	DUBCCGAQYVUYEU-UHFFFAOYSA-N	5.01	PubChem

Table S3. *Cont.*

Molid	Uniprot	SIM	REF_NN	REF_pACT	SOURCE_DB
Astragalin	P18825	0.929	OVSQVDMCBVZWGM-DHALZPNHSA-N	8.4	BindingDB, ChemblDB, PubChem
Astragalin	P18825	0.929	OVSQVDMCBVZWGM-UHFFFAOYSA-N	8.4	PDSP
Astragalin	P18825	0.916	WZCDQUUBUNNBSF-UHFFFAOYSA-N	4.7	PDSP
Astragalin	P18825	0.883	SUYDICWJCDPOPH-UHFFFAOYSA-N	4	PDSP
Astragalin	P03372	0.847	HSWIRQIYASIOBE-JNHRPPPUSA-N	5.24	ChemblDB, PubChem
Astragalin	P03372	0.847	HSWIRQIYASIOBE-UHFFFAOYSA-N	5.24	BindingDB
Kaempferol 3-*O*-β-D-glucopyranoside-7-*O*-α-L-arabinofuranoside	O76074	0.833	TZJALUIVHRYQQB-NZDRBQIJSA-N	5.23	BindingDB
Kaempferol 3-*O*-β-D-glucopyranoside-7-*O*-α-L-arabinofuranoside	O76074	0.833	TZJALUIVHRYQQB-XLRXWWTNSA-N	5.23	ChemblDB, PubChem
Kaempferol 3-*O*-β-D-glucopyranoside-7-*O*-α-L-arabinofuranoside	Q9GZQ4	0.8	VQYFUXKCTUPONZ-HJFOHNICSA-N	A	hGPCRlig
5,7-Dihydroxychromone-7-*O*-β-D-glucopyranoside	P01556	0.767	BYSXBFJVGIOFBO-BZNQNGANSA-N	A	DrugBank
5,7-Dihydroxychromone-7-*O*-β-D-glucopyranoside	P22303	0.865	FNGTXIQGXDOBSN-ZBXJEJADSA-N	6.6	PubChem, BindingDB, ChemblDB
5,7-Dihydroxychromone-7-*O*-β-D-glucopyranoside	P22303	0.86	OLZAGZCCJJBKNZ-UJPOAAIJSA-N	6.24	PubChem, BindingDB, ChemblDB
5,7-Dihydroxychromone-7-*O*-β-D-glucopyranoside	P22303	0.838	LHLBYZOFSSUOQJ-XJMPOLSOSA-N	5.32	PubChem, BindingDB, ChemblDB
5,7-Dihydroxychromone-7-*O*-β-D-glucopyranoside	P31639	0.865	UQBOAFNWHZLIFA-SFFUCWETSA-N	6.21	ChemblDB, PubChem
5,7-Dihydroxychromone-7-*O*-β-D-glucopyranoside	P31639	0.835	NCGITCMKOZTTKR-FQBWVUSXSA-N	5.92	ChemblDB, PubChem
5,7-Dihydroxychromone-7-*O*-β-D-glucopyranoside	P31639	0.831	IZNJVARPFYJAJF-FQBWVUSXSA-N	6.54	ChemblDB, PubChem
5,7-Dihydroxychromone-7-*O*-β-D-glucopyranoside	P31639	0.815	ODQAIMBPQWETBE-FQBWVUSXSA-N	7.48	BindingDB, ChemblDB, PubChem
5,7-Dihydroxychromone-7-*O*-β-D-glucopyranoside	P31639	0.797	SZHXKKDXIWLWAN-FQBWVUSXSA-N	6.72	ChemblDB, PubChem
5,7-Dihydroxychromone-7-*O*-β-D-glucopyranoside	P31639	0.788	CRTBCGCHSYCNOS-FQBWVUSXSA-N	7.16	ChemblDB, PubChem

Table S3. *Cont.*

Molid	Uniprot	SIM	REF_NN	REF_pACT	SOURCE_DB
5,7-Dihydroxychromone-7-*O*-β-D-glucopyranoside	P31639	0.788	DFTBZKANYGHGRH-FQBWVUSXSA-N	6.27	ChemblDB, PubChem
5,7-Dihydroxychromone-7-*O*-β-D-glucopyranoside	P31639	0.775	PJFAFCHMHLNMSX-OUUBHVDSSA-N	8.1	ChemblDB, PubChem
5,7-Dihydroxychromone-7-*O*-β-D-glucopyranoside	P32890	0.813	MIAKOEWBCMPCQR-RMPHRYRLSA-N	A	DrugBank
5,7-Dihydroxychromone-7-*O*-β-D-glucopyranoside	P32890	0.749	FSMWGHKWKYCPKE-QTVCLEQKSA-N	A	DrugBank
5,7-Dihydroxychromone-7-*O*-neohesperidoside	P61088	0.814	DFPMSGMNTNDNHN-ZFOFJSCHSA-N	5.08	PubChem
5,7-Dihydroxychromone-7-*O*-neohesperidoside	P61088	0.814	DFPMSGMNTNDNHN-ZHNJBYEHSA-N	5.12	PubChem
5,7-Dihydroxychromone-7-*O*-neohesperidoside	P11511	0.87	SXNOCVMJOZRSLS-MCEICCLHSA-N	5.3	BindingDB
5,7-Dihydroxychromone-7-*O*-neohesperidoside	P11511	0.814	DFPMSGMNTNDNHN-ZHNJBYEHSA-N	5.3	PubChem
5,7-Dihydroxychromone-7-*O*-neohesperidoside	P11511	0.814	DFPMSGMNTNDNHN-ZPHOTFPESA-N	5.3	BindingDB, ChemblDB
5,7-Dihydroxychromone-7-*O*-neohesperidoside	O00182	0.822	CNUQWQWKOYKPBP-AGZUGLJOSA-N	5.8	BindingDB, ChemblDB
5,7-Dihydroxychromone-7-*O*-neohesperidoside	O00182	0.822	CNUQWQWKOYKPBP-MDQQUCTKSA-N	5.8	PubChem
5,7-Dihydroxychromone-7-*O*-neohesperidoside	Q9Y271	0.857	KCTMSAJMWDVYEY-RFBNSGMDSA-N	A	hGPCRlig
5,7-Dihydroxychromone-7-*O*-neohesperidoside	Q9Y271	0.851	ISIAVDYYQSNUOL-GQGAKGAPSA-N	A	hGPCRlig
5,7-Dihydroxychromone-7-*O*-neohesperidoside	Q9NS75	0.857	KCTMSAJMWDVYEY-RFBNSGMDSA-N	A	hGPCRlig
5,7-dihydroxychromone-7-*O*-neohesperidoside	Q9NS75	0.851	ISIAVDYYQSNUOL-GQGAKGAPSA-N	A	hGPCRlig
Kaempferol 3-*O*-β-D-glucopyranoside-7-*O*-β-D-glucopyranoside	O76074	0.845	TZJALUIVHRYQQB-NZDRBQIJSA-N	5.23	BindingDB
Kaempferol 3-*O*-β-D-glucopyranoside-7-*O*-β-D-glucopyranoside	O76074	0.845	TZJALUIVHRYQQB-XLRXWWTNSA-N	5.23	ChemblDB, PubChem

Table S3. *Cont.*

Molid	Uniprot	SIM	REF_NN	REF_pACT	SOURCE_DB
Kaempferol 3-*O*-β-D-glucopyranoside-7-*O*-β-D-glucopyranoside	Q9GZQ4	0.819	VQYFUXKCTUPONZ-HJFOHNICSA-N	A	hGPCRlig
Xanthohumol	Q9BQF6	0.921	SVTCZHIDEDUTBH-UHFFFAOYSA-N	5.11	PubChem
Xanthohumol	Q71U36	0.808	BZXULYMZYPRZOG-UHFFFAOYSA-N	5.52	BindingDB, ChemblDB, PubChem
Xanthohumol	Q3ZCM7	0.814	ZSPZNFOLWQEVQJ-UHFFFAOYSA-N	5.52	ChemblDB, PubChem
Xanthohumol	Q3ZCM7	0.81	DEQJJTUOVGHXHW-UHFFFAOYSA-N	3.9	ChemblDB, PubChem
Xanthohumol	Q3ZCM7	0.808	BZXULYMZYPRZOG-UHFFFAOYSA-N	5.7	ChemblDB, PubChem
Xanthohumol	P04798	0.849	SCZVLDHREVKTSH-UHFFFAOYSA-N	7.02	ChemblDB, PubChem
Xanthohumol	P04798	0.792	MBNGWHIJMBWFHU-UHFFFAOYSA-N	6.85	ChemblDB, PubChem
Xanthohumol	P05177	0.849	SCZVLDHREVKTSH-UHFFFAOYSA-N	5.95	PubChem, ChemblDB
Xanthohumol	P05177	0.792	MBNGWHIJMBWFHU-UHFFFAOYSA-N	5.61	PubChem, ChemblDB
Xanthohumol	Q16678	0.849	SCZVLDHREVKTSH-UHFFFAOYSA-N	7.7	ChemblDB, PubChem
Xanthohumol	Q16678	0.792	MBNGWHIJMBWFHU-UHFFFAOYSA-N	7.54	ChemblDB, PubChem
Xanthohumol	P10635	0.832	VJJZJBUCDWKPLC-UHFFFAOYSA-N	5.33	BindingDB, ChemblDB, PubChem
Xanthohumol	P00533	0.785	OBBCRPUNCUPUOS-UHFFFAOYSA-N	5.48	BindingDB, ChemblDB, PubChem
Xanthohumol	P15121	0.862	XRHHDQSPFPQKMS-UHFFFAOYSA-N	5.35	ChemblDB
Xanthohumol	P15121	0.853	SYGUVOLSUJYPPS-UHFFFAOYSA-N	6.39	ChemblDB
Xanthohumol	P15121	0.828	DAUMHRNXYGHXIC-UHFFFAOYSA-N	6.07	ChemblDB
Xanthohumol	P15121	0.813	BAIRXMVFPKLWSE-UHFFFAOYSA-N	3.96	ChemblDB
Xanthohumol	P09917	0.777	AJSXSWPVSPYVGO-GORDUTHDSA-N	8.05	PubChem, BindingDB, ChemblDB
Xanthohumol	P47989	0.849	SCZVLDHREVKTSH-UHFFFAOYSA-N	4.85	BindingDB, ChemblDB, PubChem
Xanthohumol	P47989	0.846	DDNPCXHBFYJXBJ-UHFFFAOYSA-N	7.52	BindingDB, ChemblDB, PubChem
Xanthohumol	P08263	0.849	SCZVLDHREVKTSH-UHFFFAOYSA-N	5.76	PubChem
Xanthohumol	Q9NZK7	0.884	ZUGCRBMNFSAUOC-YRNVUSSQSA-N	A	DrugBank
Xanthohumol	O76074	0.914	MEHHCBRCXIDGKZ-UHFFFAOYSA-N	5.89	BindingDB, ChemblDB, PubChem
Xanthohumol	P10520	0.921	SVTCZHIDEDUTBH-UHFFFAOYSA-N	5.56	PubChem
Xanthohumol	P10520	0.909	YHWNASRGLKJRJJ-UHFFFAOYSA-N	5.22	PubChem
Xanthohumol	P10520	0.867	XDKYBPGIBVMHHB-KPKJPENVSA-N	5.67	PubChem
Xanthohumol	P10520	0.86	LRSMBOSQWGHYCW-MDGZPELGSA-N	5.99	PubChem
Xanthohumol	P10520	0.841	UCHYSPNEUSDFQR-UHFFFAOYSA-N	5.73	PubChem
Xanthohumol	P28222	0.786	RGGJEENSFLRVRP-UHFFFAOYSA-N	5.07	PDSP
Xanthohumol	P28223	0.786	RGGJEENSFLRVRP-UHFFFAOYSA-N	6.26	PDSP
Xanthohumol	P28335	0.786	RGGJEENSFLRVRP-UHFFFAOYSA-N	5.91	PDSP
Xanthohumol	P41595	0.786	RGGJEENSFLRVRP-UHFFFAOYSA-N	5.17	PDSP

Table S3. *Cont.*

Molid	Uniprot	SIM	REF_NN	REF_pACT	SOURCE_DB
Xanthohumol	P30542	0.841	IHFBPDAQLQOCBX-UHFFFAOYSA-N	5.79	ChemblDB, PubChem, BindingDB
Xanthohumol	P29274	0.841	IHFBPDAQLQOCBX-UHFFFAOYSA-N	5.19	BindingDB, ChemblDB, PubChem
Xanthohumol	P14867	0.841	IHFBPDAQLQOCBX-UHFFFAOYSA-N	6	ChemblDB
Xanthohumol	P47869	0.841	IHFBPDAQLQOCBX-UHFFFAOYSA-N	6	ChemblDB
Xanthohumol	P34903	0.841	IHFBPDAQLQOCBX-UHFFFAOYSA-N	6	ChemblDB
Xanthohumol	P48169	0.841	IHFBPDAQLQOCBX-UHFFFAOYSA-N	6	ChemblDB
Xanthohumol	P31644	0.841	IHFBPDAQLQOCBX-UHFFFAOYSA-N	6	ChemblDB
Xanthohumol	Q16445	0.841	IHFBPDAQLQOCBX-UHFFFAOYSA-N	6	ChemblDB
Xanthohumol	P03372	0.817	VSEIMGCATUFLSE-UHFFFAOYSA-N	4.99	BindingDB, ChemblDB, PubChem
Xanthohumol	P03372	0.791	JWLBHKJXDMACKX-UHFFFAOYSA-N	5.21	BindingDB
Xanthohumol	P03372	0.774	HHVMWRVANSDUKF-UHFFFAOYSA-N	7.51	BindingDB, ChemblDB, PubChem
Xanthohumol	Q92731	0.817	VSEIMGCATUFLSE-UHFFFAOYSA-N	6.06	PubChem, ChemblDB, BindingDB
Xanthohumol	Q92731	0.791	JWLBHKJXDMACKX-UHFFFAOYSA-N	6.41	BindingDB
Xanthohumol	Q92731	0.774	HHVMWRVANSDUKF-UHFFFAOYSA-N	8.62	PubChem, ChemblDB, BindingDB
Xanthohumol	P33527	0.849	SCZVLDHREVKTSH-UHFFFAOYSA-N	5.52	BindingDB, ChemblDB, PubChem
Xanthohumol	P33527	0.792	MBNGWHIJMBWFHU-UHFFFAOYSA-N	5.52	BindingDB, ChemblDB, PubChem
Epicatechin	P10721	0.865	WZUVPPKBWHMQCE-XJKSGUPXSA-N	5.57	BindingDB, ChemblDB, PubChem
Epicatechin	P35968	0.865	WZUVPPKBWHMQCE-XJKSGUPXSA-N	5.68	BindingDB, ChemblDB, PubChem
Epicatechin	P11362	0.865	WZUVPPKBWHMQCE-XJKSGUPXSA-N	6.5	BindingDB, ChemblDB, PubChem
Epicatechin	P08581	0.865	WZUVPPKBWHMQCE-XJKSGUPXSA-N	6.4	BindingDB, ChemblDB, PubChem
Epicatechin	Q16678	0.836	SBHXYTNGIZCORC-ZDUSSCGKSA-N	5.89	ChemblDB, PubChem
Epicatechin	P11511	0.784	QBLQLKNOKUHRCH-ZDUSSCGKSA-N	5.66	BindingDB, ChemblDB, PubChem
Epicatechin	P00533	0.865	WZUVPPKBWHMQCE-XJKSGUPXSA-N	5.47	BindingDB, ChemblDB, PubChem
Epicatechin	P04626	0.865	WZUVPPKBWHMQCE-XJKSGUPXSA-N	6.02	BindingDB, ChemblDB, PubChem
Epicatechin	P17948	0.865	WZUVPPKBWHMQCE-XJKSGUPXSA-N	5.54	BindingDB, ChemblDB, PubChem
Epicatechin	P0A7G6	sCOLL	PFTAWBLQPZVEMU-DZGCQCFKSA-N	5.39	PubChem
Epicatechin	Q965D5	0.939	XMOCLSLCDHWDHP-IUODEOHRSA-N	5.36	BindingDB, ChemblDB
Epicatechin	Q965D5	0.801	CXQWRCVTCMQVQX-HUUCEWRRSA-N	6.14	BindingDB
Epicatechin	P12931	0.865	WZUVPPKBWHMQCE-XJKSGUPXSA-N	6.36	BindingDB, ChemblDB, PubChem
Epicatechin	Q8I2J3	sCOLL	PFTAWBLQPZVEMU-UKRRQHHQSA-N	5.86	PubChem
Epicatechin	P03070	COLL	PFTAWBLQPZVEMU-UHFFFAOYSA-N	5.06	PubChem
Epicatechin	P03070	sCOLL	PFTAWBLQPZVEMU-DZGCQCFKSA-N	4.94	PubChem
Epicatechin	P03070	0.865	WZUVPPKBWHMQCE-UHFFFAOYSA-N	5.33	PubChem
Epicatechin	P03070	0.84	ZPVNWCMRCGXRJD-UHFFFAOYSA-N	5.72	PubChem
Epicatechin	P03070	0.801	CXQWRCVTCMQVQX-LSDHHAIUSA-N	4.5	PubChem

Table S3. *Cont.*

Molid	Uniprot	SIM	REF_NN	REF_pACT	SOURCE_DB
Epicatechin	O43570	sCOLL	PFTAWBLQPZVEMU-HIFRSBDPSA-N	5.33	ChemblDB, PubChem
Epicatechin	P00915	sCOLL	PFTAWBLQPZVEMU-HIFRSBDPSA-N	5.62	ChemblDB, PubChem
Epicatechin	P00918	sCOLL	PFTAWBLQPZVEMU-HIFRSBDPSA-N	5.74	ChemblDB, PubChem
Epicatechin	P07451	sCOLL	PFTAWBLQPZVEMU-HIFRSBDPSA-N	5.45	ChemblDB, PubChem
Epicatechin	P22748	sCOLL	PFTAWBLQPZVEMU-HIFRSBDPSA-N	5.31	ChemblDB, PubChem
Epicatechin	P23280	sCOLL	PFTAWBLQPZVEMU-HIFRSBDPSA-N	5.31	ChemblDB, PubChem
Epicatechin	P35218	sCOLL	PFTAWBLQPZVEMU-HIFRSBDPSA-N	5.38	ChemblDB, PubChem
Epicatechin	P43166	sCOLL	PFTAWBLQPZVEMU-HIFRSBDPSA-N	6.35	ChemblDB, PubChem
Epicatechin	Q16790	sCOLL	PFTAWBLQPZVEMU-HIFRSBDPSA-N	5.3	ChemblDB, PubChem
Epicatechin	Q99N23	sCOLL	PFTAWBLQPZVEMU-HIFRSBDPSA-N	5.12	ChemblDB, PubChem
Epicatechin	Q9Y2D0	sCOLL	PFTAWBLQPZVEMU-HIFRSBDPSA-N	5.4	ChemblDB, PubChem
Epicatechin	Q07820	0.865	WZUVPPKBWHMQCE-UHFFFAOYSA-N	5.46	PubChem
Epicatechin	Q07820	0.84	ZPVNWCMRCGXRJD-UHFFFAOYSA-N	5.52	PubChem
(*E*)-4-*O*-β-D-Glucopyranosyl caffeic acid	P01556	0.887	VCCMGHVCRFMITI-SVNGYHJRSA-N	A	DrugBank
(*E*)-4-*O*-β-D-Glucopyranosyl caffeic acid	P07464	0.887	IFBHRQDFSNCLOZ-SVNGYHJRSA-N	A	DrugBank
(*E*)-4-O-β-D-Glucopyranosyl caffeic acid	Q700S9	0.887	IFBHRQDFSNCLOZ-SVNGYHJRSA-N	A	DrugBank
(*E*)-4-O-β-D-Glucopyranosyl caffeic acid	Q700S9	0.886	KUWPCJHYPSUOFW-SVNGYHJRSA-N	A	DrugBank
(*E*)-4-*O*-β-D-Glucopyranosyl caffeic acid	P13866	0.793	HFLCZNNDZKKXCS-OUUBHVDSSA-N	4.6	ChemblDB, PubChem
(*E*)-4-*O*-β-D-Glucopyranosyl caffeic acid	P13866	0.77		6.62	BindingDB, ChemblDB, PubChem
(*E*)-4-*O*-β-D-Glucopyranosyl caffeic acid	P31639	0.826	NCGITCMKOZTTKR-FQBWVUSXSA-N	5.92	ChemblDB, PubChem
(*E*)-4-*O*-β-D-Glucopyranosyl caffeic acid	P31639	0.816	IZNJVARPFYJAJF-FQBWVUSXSA-N	6.54	ChemblDB, PubChem
(*E*)-4-*O*-β-D-Glucopyranosyl caffeic acid	P31639	0.796	GMIUHEOBZYBBHN-OBKDMQGPSA-N	6.29	ChemblDB, PubChem
(*E*)-4-*O*-β-D-Glucopyranosyl caffeic acid	P31639	0.793	HFLCZNNDZKKXCS-OUUBHVDSSA-N	8.08	BindingDB, ChemblDB, PubChem
(*E*)-4-*O*-β-D-Glucopyranosyl caffeic acid	P31639	0.79	CRTBCGCHSYCNOS-FQBWVUSXSA-N	7.16	ChemblDB, PubChem
(*E*)-4-*O*-β-D-Glucopyranosyl caffeic acid	P31639	0.79	DFTBZKANYGHGRH-FQBWVUSXSA-N	6.27	ChemblDB, PubChem
(*E*)-4-*O*-β-D-Glucopyranosyl caffeic acid	P31639	0.789	KHFPHQJDPNKPJV-OBKDMQGPSA-N	7.66	ChemblDB, PubChem
(*E*)-4-*O*-β-D-Glucopyranosyl caffeic acid	P31639	0.784	PJFAFCHMHLNMSX-OUUBHVDSSA-N	8.1	ChemblDB, PubChem
(*E*)-4-*O*-β-D-Glucopyranosyl caffeic acid	P31639	0.77	UEHTWMMZNLKDPS-OBKDMQGPSA-N	9.16	BindingDB, ChemblDB, PubChem
(*E*)-4-O-β-D-Glucopyranosyl caffeic acid	P32890	0.887	VCCMGHVCRFMITI-SVNGYHJRSA-N	A	DrugBank
(*E*)-4-O-β-D-Glucopyranosyl caffeic acid	Q6UWM7	0.887	IXFOBQXJWRLXMD-ZIQFBCGOSA-N	A	DrugBank
(*E*)-4-*O*-β-D-Glucopyranosyl sinapoic acid	P01556	0.86	BYSXBFJVGIOFBO-BZNQNGANSA-N	A	DrugBank
(*E*)-4-*O*-β-D-Glucopyranosyl sinapoic acid	P01556	0.859	VCCMGHVCRFMITI-SVNGYHJRSA-N	A	DrugBank
(*E*)-4-*O*-β-D-Glucopyranosyl sinapoic acid	P07464	0.859	IFBHRQDFSNCLOZ-SVNGYHJRSA-N	A	DrugBank

Table S3. *Cont.*

Molid	Uniprot	SIM	REF_NN	REF_pACT	SOURCE_DB
(*E*)-4-*O*-β-D-Glucopyranosyl sinapoic acid	Q700S9	0.86	KUWPCJHYPSUOFW-SVNGYHJRSA-N	A	DrugBank
(*E*)-4-*O*-β-D-Glucopyranosyl sinapoic acid	Q700S9	0.859	IFBHRQDFSNCLOZ-SVNGYHJRSA-N	A	DrugBank
(*E*)-4-*O*-β-D-Glucopyranosyl sinapoic acid	P32890	0.859	VCCMGHVCRFMITI-SVNGYHJRSA-N	A	DrugBank
(*E*)-4-*O*-β-D-Glucopyranosyl sinapoic acid	P32890	0.781	MIAKOEWBCMPCQR-RMPHRYRLSA-N	A	DrugBank
(*E*)-4-*O*-β-D-Glucopyranosyl sinapoic acid	Q6UWM7	0.859	IXFOBQXJWRLXMD-ZIQFBCGOSA-N	A	DrugBank
4-*O*-β-D-glucopyranosyl ferulic acid	P01556	0.86	BYSXBFJVGIOFBO-BZNQNGANSA-N	A	DrugBank
4-*O*-β-D-glucopyranosyl ferulic acid	P01556	0.859	VCCMGHVCRFMITI-SVNGYHJRSA-N	A	DrugBank
4-*O*-β-D-glucopyranosyl ferulic acid	P07464	0.859	IFBHRQDFSNCLOZ-SVNGYHJRSA-N	A	DrugBank
4-*O*-β-D-glucopyranosyl ferulic acid	Q700S9	0.86	KUWPCJHYPSUOFW-SVNGYHJRSA-N	A	DrugBank
4-*O*-β-D-glucopyranosyl ferulic acid	Q700S9	0.859	IFBHRQDFSNCLOZ-SVNGYHJRSA-N	A	DrugBank
4-*O*-β-D-glucopyranosyl ferulic acid	P32890	0.859	VCCMGHVCRFMITI-SVNGYHJRSA-N	A	DrugBank
4-*O*-β-D-glucopyranosyl ferulic acid	P32890	0.781	MIAKOEWBCMPCQR-RMPHRYRLSA-N	A	DrugBank
4-*O*-β-D-glucopyranosyl ferulic acid	Q6UWM7	0.859	IXFOBQXJWRLXMD-ZIQFBCGOSA-N	A	DrugBank
Trans-caffeic acid	Q14914	0.858	NGSWKAQJJWESNS-ZZXKWVIFSA-N	A	DrugBank
Trans-caffeic acid	Q06327	sCOLL	QAIPRVGONGVQAS-DUXPYHPUSA-N	5.52	BindingDB, ChemblDB, PubChem
Trans-caffeic acid	P18031	sCOLL	QAIPRVGONGVQAS-DUXPYHPUSA-N	5.51	BindingDB, ChemblDB, PubChem
Trans-caffeic acid	P17538	0.93	HGEFWFBFQKWVMY-DUXPYHPUSA-N	A	DrugBank
Trans-caffeic acid	P03070	sCOLL	QAIPRVGONGVQAS-DUXPYHPUSA-N	5.14	PubChem
Trans-caffeic acid	P03070	0.858	NGSWKAQJJWESNS-ZZXKWVIFSA-N	4.72	PubChem
Trans-caffeic acid	O43570	sCOLL	QAIPRVGONGVQAS-DUXPYHPUSA-N	5.04	ChemblDB, PubChem, BindingDB
Trans-caffeic acid	O43570	0.858	NGSWKAQJJWESNS-ZZXKWVIFSA-N	5.1	ChemblDB, PubChem, BindingDB
Trans-caffeic acid	P00915	sCOLL	QAIPRVGONGVQAS-DUXPYHPUSA-N	5.62	ChemblDB, PubChem, BindingDB
Trans-caffeic acid	P00915	0.858	NGSWKAQJJWESNS-ZZXKWVIFSA-N	5.97	ChemblDB, PubChem, BindingDB
Trans-caffeic acid	P00918	sCOLL	QAIPRVGONGVQAS-DUXPYHPUSA-N	5.79	ChemblDB, BindingDB, PubChem
Trans-caffeic acid	P00918	0.858	NGSWKAQJJWESNS-ZZXKWVIFSA-N	6.01	ChemblDB, BindingDB, PubChem
Trans-caffeic acid	P07451	sCOLL	QAIPRVGONGVQAS-DUXPYHPUSA-N	5	ChemblDB, BindingDB, PubChem
Trans-caffeic acid	P07451	0.858	NGSWKAQJJWESNS-ZZXKWVIFSA-N	5.12	ChemblDB, BindingDB, PubChem
Trans-caffeic acid	P22748	sCOLL	QAIPRVGONGVQAS-DUXPYHPUSA-N	5	ChemblDB, BindingDB, PubChem
Trans-caffeic acid	P22748	0.858	NGSWKAQJJWESNS-ZZXKWVIFSA-N	5.02	ChemblDB, BindingDB, PubChem
Trans-caffeic acid	P23280	sCOLL	QAIPRVGONGVQAS-DUXPYHPUSA-N	5.13	ChemblDB, BindingDB, PubChem
Trans-caffeic acid	P23280	0.858	NGSWKAQJJWESNS-ZZXKWVIFSA-N	5.17	ChemblDB, BindingDB, PubChem

Table S3. *Cont.*

Molid	Uniprot	SIM	REF_NN	REF_pACT	SOURCE_DB
Trans-caffeic acid	P35218	sCOLL	QAIPRVGONGVQAS-DUXPYHPUSA-N	5.19	ChemblDB, BindingDB, PubChem
Trans-caffeic acid	P35218	0.858	NGSWKAQJJWESNS-ZZXKWVIFSA-N	5.22	ChemblDB, BindingDB, PubChem
Trans-caffeic acid	P43166	sCOLL	QAIPRVGONGVQAS-DUXPYHPUSA-N	5.19	ChemblDB, BindingDB, PubChem
Trans-caffeic acid	P43166	0.858	NGSWKAQJJWESNS-ZZXKWVIFSA-N	5.28	ChemblDB, BindingDB, PubChem
Trans-caffeic acid	Q16790	sCOLL	QAIPRVGONGVQAS-DUXPYHPUSA-N	5.1	ChemblDB, PubChem, BindingDB
Trans-caffeic acid	Q16790	0.858	NGSWKAQJJWESNS-ZZXKWVIFSA-N	5.27	ChemblDB, PubChem, BindingDB
Trans-caffeic acid	Q9ULX7	sCOLL	QAIPRVGONGVQAS-DUXPYHPUSA-N	5.06	ChemblDB, BindingDB, PubChem
Trans-caffeic acid	Q9ULX7	0.858	NGSWKAQJJWESNS-ZZXKWVIFSA-N	5.17	ChemblDB, BindingDB, PubChem
Trans-caffeic acid	Q9Y2D0	sCOLL	QAIPRVGONGVQAS-DUXPYHPUSA-N	5.04	ChemblDB, BindingDB, PubChem
Trans-caffeic acid	Q9Y2D0	0.858	NGSWKAQJJWESNS-ZZXKWVIFSA-N	5.11	ChemblDB, BindingDB, PubChem
Trans-caffeic acid	P42357	sCOLL	QAIPRVGONGVQAS-DUXPYHPUSA-N	A	DrugBank
Trans-caffeic acid	P42357	0.858	NGSWKAQJJWESNS-ZZXKWVIFSA-N	A	DrugBank
Trans-caffeic acid	P14174	sCOLL	QAIPRVGONGVQAS-DUXPYHPUSA-N	A	DrugBank
Trans-caffeic acid	P14174	0.785	FYJKSAQOWJFNMW-DUXPYHPUSA-N	5.58	BindingDB, ChemblDB, PubChem
Trans-caffeic acid	P16113	sCOLL	QAIPRVGONGVQAS-DUXPYHPUSA-N	A	DrugBank
Trans-caffeic acid	P16113	0.858	NGSWKAQJJWESNS-ZZXKWVIFSA-N	A	DrugBank
Trans-caffeic acid	Q9X2W8	0.858	NGSWKAQJJWESNS-ZZXKWVIFSA-N	A	DrugBank
4-*O*-β-D-Glucopyranosyl coumaric acid	P01556	0.934	VCCMGHVCRFMITI-SVNGYHJRSA-N	A	DrugBank
4-*O*-β-D-Glucopyranosyl coumaric acid	P07464	0.934	IFBHRQDFSNCLOZ-SVNGYHJRSA-N	A	DrugBank
4-*O*-β-D-Glucopyranosyl coumaric acid	Q700S9	0.934	IFBHRQDFSNCLOZ-SVNGYHJRSA-N	A	DrugBank
4-*O*-β-D-Glucopyranosyl coumaric acid	Q700S9	0.933	KUWPCJHYPSUOFW-SVNGYHJRSA-N	A	DrugBank
4-*O*-β-D-Glucopyranosyl coumaric acid	P31639	0.793	UQBOAFNWHZLIFA-SFFUCWETSA-N	6.21	ChemblDB, PubChem
4-*O*-β-D-Glucopyranosyl coumaric acid	P31639	0.773	SZHXKKDXIWLWAN-FQBWVUSXSA-N	6.72	ChemblDB, PubChem
4-*O*-β-D-Glucopyranosyl coumaric acid	P31639	0.765	CRTBCGCHSYCNOS-FQBWVUSXSA-N	7.16	ChemblDB, PubChem
4-*O*-β-D-Glucopyranosyl coumaric acid	P31639	0.765	DFTBZKANYGHGRH-FQBWVUSXSA-N	6.27	ChemblDB, PubChem
4-*O*-β-D-Glucopyranosyl coumaric acid	P31639	0.765	IZNJVARPFYJAJF-FQBWVUSXSA-N	6.54	ChemblDB, PubChem
4-*O*-β-D-Glucopyranosyl coumaric acid	P31639	0.765	NCGITCMKOZTTKR-FQBWVUSXSA-N	5.92	ChemblDB, PubChem
4-*O*-β-D-Glucopyranosyl coumaric acid	P31639	0.749	GMIUHEOBZYBBHN-OBKDMQGPSA-N	6.29	ChemblDB, PubChem
4-*O*-β-D-Glucopyranosyl coumaric acid	P31639	0.742	KHFPHQJDPNKPJV-OBKDMQGPSA-N	7.66	ChemblDB, PubChem
4-*O*-β-D-Glucopyranosyl coumaric acid	P32890	0.934	VCCMGHVCRFMITI-SVNGYHJRSA-N	A	DrugBank
4-*O*-β-D-Glucopyranosyl coumaric acid	Q6UWM7	0.934	IXFOBQXJWRLXMD-ZIQFBCGOSA-N	A	DrugBank
Dihydrocaffeic acid methyl ester	P10696	0.79	XBBDACCLCFWBSI-UHFFFAOYSA-N	5.03	PubChem
Dihydrocaffeic acid	P10635	0.8	WTDRDQBEARUVNC-LURJTMIESA-N	A	DrugBank
Dihydrocaffeic acid	O67636	0.858	NMHMNPHRMNGLLB-UHFFFAOYSA-N	A	DrugBank
Dihydrocaffeic acid	P21397	0.8	WTDRDQBEARUVNC-LURJTMIESA-N	A	DrugBank
Dihydrocaffeic acid	P27338	0.8	WTDRDQBEARUVNC-LURJTMIESA-N	A	DrugBank

Table S3. *Cont.*

Molid	Uniprot	SIM	REF_NN	REF_pACT	SOURCE_DB
Dihydrocaffeic acid	P00436	0.78	CFFZDZCDUFSOFZ-UHFFFAOYSA-N	A	DrugBank
Dihydrocaffeic acid	P00437	0.78	CFFZDZCDUFSOFZ-UHFFFAOYSA-N	A	DrugBank
Dihydrocaffeic acid	Q44048	0.78	CFFZDZCDUFSOFZ-UHFFFAOYSA-N	A	DrugBank
Dihydrocaffeic acid	P14679	0.909	HMCMTJPPXSGYJY-UHFFFAOYSA-N	5.73	ChemblDB, PubChem, BindingDB
Dihydrocaffeic acid	P14679	0.8	WTDRDQBEARUVNC-LURJTMIESA-N	2.08	ChemblDB, PubChem, BindingDB
Dihydrocaffeic acid	P09923	0.78	CFFZDZCDUFSOFZ-UHFFFAOYSA-N	5.38	PubChem
Dihydrocaffeic acid	P06875	0.78	CFFZDZCDUFSOFZ-UHFFFAOYSA-N	A	DrugBank
Dihydrocaffeic acid	P20711	0.8	WTDRDQBEARUVNC-LURJTMIESA-N	A	DrugBank
Dihydrocaffeic acid	P21918	0.8	WTDRDQBEARUVNC-LURJTMIESA-N	A	DrugBank
Dihydrocaffeic acid	P35462	0.8	WTDRDQBEARUVNC-LURJTMIESA-N	A	DrugBank
Dihydrocaffeic acid	Q8TF71	0.8	WTDRDQBEARUVNC-LURJTMIESA-N	A	DrugBank
Dihydrocaffeic acid	P46059	0.8	WTDRDQBEARUVNC-LURJTMIESA-N	A	DrugBank
Dihydrocaffeic acid	Q45135	0.78	CFFZDZCDUFSOFZ-UHFFFAOYSA-N	A	DrugBank
3,4-Dihydroxyl benzoic acid	P04181	0.858	XFDUHJPVQKIXHO-UHFFFAOYSA-N	A	DrugBank
3,4-Dihydroxyl benzoic acid	P23893	0.858	XFDUHJPVQKIXHO-UHFFFAOYSA-N	A	DrugBank
3,4-Dihydroxyl benzoic acid	P08183	0.858	BTJIUGUIPKRLHP-UHFFFAOYSA-N	A	DrugBank
3,4-Dihydroxyl benzoic acid	Q9Y6F1	0.858	XFDUHJPVQKIXHO-UHFFFAOYSA-N	5.2	BindingDB, ChemblDB, PubChem
3,4-Dihydroxyl benzoic acid	P0A7G6	0.928	WJXSWCUQABXPFS-UHFFFAOYSA-N	5.27	PubChem
3,4-Dihydroxyl benzoic acid	P0A7G6	0.901	UONVFNLDGRWLKF-UHFFFAOYSA-N	5.21	PubChem
3,4-Dihydroxyl benzoic acid	P46952	0.822	VWEPFJPQZFIOAU-UHFFFAOYSA-N	A	DrugBank
3,4-Dihydroxyl benzoic acid	P09917	0.901	KBOPZPXVLCULAV-UHFFFAOYSA-N	A	DrugBank
3,4-Dihydroxyl benzoic acid	P09917	0.887	WUBBRNOQWQTFEX-UHFFFAOYSA-N	A	DrugBank
3,4-Dihydroxyl benzoic acid	P20586	COLL	YQUVCSBJEUQKSH-UHFFFAOYSA-N	5.34	MOAD, affinDB
3,4-Dihydroxyl benzoic acid	P20586	0.887	UIAFKZKHHVMJGS-UHFFFAOYSA-N	A	DrugBank
3,4-Dihydroxyl benzoic acid	P20586	0.858	ALYNCZNDIQEVRV-UHFFFAOYSA-N	3.83	MOAD, DrugBank
3,4-Dihydroxyl benzoic acid	P20586	0.858	FJKROLUGYXJWQN-UHFFFAOYSA-N	4.49	MOAD, DrugBank, affinDB
3,4-Dihydroxyl benzoic acid	Q81VW8	0.858	ALYNCZNDIQEVRV-UHFFFAOYSA-N	5.25	ChemblDB, PubChem
3,4-Dihydroxyl benzoic acid	P26281	0.887	WUBBRNOQWQTFEX-UHFFFAOYSA-N	A	DrugBank
3,4-Dihydroxyl benzoic acid	P40871	0.928	GLDQAMYCGOIJDV-UHFFFAOYSA-N	A	DrugBank
3,4-Dihydroxyl benzoic acid	Q12851	0.901	UONVFNLDGRWLKF-UHFFFAOYSA-N	5.42	PubChem
3,4-Dihydroxyl benzoic acid	O15111	0.901	KBOPZPXVLCULAV-UHFFFAOYSA-N	A	DrugBank
3,4-Dihydroxyl benzoic acid	O15111	0.887	WUBBRNOQWQTFEX-UHFFFAOYSA-N	A	DrugBank
3,4-Dihydroxyl benzoic acid	O14920	0.901	KBOPZPXVLCULAV-UHFFFAOYSA-N	A	DrugBank
3,4-Dihydroxyl benzoic acid	P50225	0.858	BTJIUGUIPKRLHP-UHFFFAOYSA-N	A	DrugBank
3,4-Dihydroxyl benzoic acid	Q9NZK7	0.887	WUBBRNOQWQTFEX-UHFFFAOYSA-N	A	DrugBank
3,4-Dihydroxyl benzoic acid	Q04416	0.858	FJKROLUGYXJWQN-UHFFFAOYSA-N	A	DrugBank

Table S3. *Cont.*

MOLID	UNIPROT	SIM	REF_NN	REF_PACT	SOURCE_DB
3,4-Dihydroxyl benzoic acid	P19961	0.858	BTJIUGUIPKRLHP-UHFFFAOYSA-N	A	DrugBank
3,4-Dihydroxyl benzoic acid	Q8I2J3	COLL	YQUVCSBJEUQKSH-UHFFFAOYSA-N	6.32	PubChem
3,4-Dihydroxyl benzoic acid	P26602	0.858	FJKROLUGYXJWQN-UHFFFAOYSA-N	A	DrugBank
3,4-Dihydroxyl benzoic acid	O43570	0.858	FJKROLUGYXJWQN-UHFFFAOYSA-N	5.26	ChemblDB, PubChem, BindingDB
3,4-Dihydroxyl benzoic acid	O43570	0.832	LNTHITQWFMADLM-UHFFFAOYSA-N	5.11	ChemblDB, PubChem, BindingDB
3,4-Dihydroxyl benzoic acid	P23280	0.858	FJKROLUGYXJWQN-UHFFFAOYSA-N	5.09	ChemblDB, BindingDB, PubChem
3,4-Dihydroxyl benzoic acid	P23280	0.832	LNTHITQWFMADLM-UHFFFAOYSA-N	5.21	ChemblDB, BindingDB, PubChem
3,4-Dihydroxyl benzoic acid	P35218	0.858	FJKROLUGYXJWQN-UHFFFAOYSA-N	5.44	ChemblDB, BindingDB, PubChem
3,4-Dihydroxyl benzoic acid	P35218	0.832	LNTHITQWFMADLM-UHFFFAOYSA-N	5.39	ChemblDB, BindingDB, PubChem
3,4-Dihydroxyl benzoic acid	Q99N23	0.858	FJKROLUGYXJWQN-UHFFFAOYSA-N	5.03	ChemblDB, PubChem
3,4-Dihydroxyl benzoic acid	Q9Y2D0	0.858	FJKROLUGYXJWQN-UHFFFAOYSA-N	5.07	ChemblDB, BindingDB, PubChem
3,4-Dihydroxyl benzoic acid	Q9Y2D0	0.832	LNTHITQWFMADLM-UHFFFAOYSA-N	5	ChemblDB, BindingDB, PubChem
3,4-Dihydroxyl benzoic acid	Q51792	0.928	WJXSWCUQABXPFS-UHFFFAOYSA-N	5.85	DrugBank, MOAD
3,4-Dihydroxyl benzoic acid	P37231	0.901	KBOPZPXVLCULAV-UHFFFAOYSA-N	A	DrugBank
3,4-Dihydroxyl benzoic acid	Q07820	0.901	UONVFNLDGRWLKF-UHFFFAOYSA-N	5.8	PubChem
3,4-Dihydroxyl benzoic acid	P80188	0.928	GLDQAMYCGOIJDV-UHFFFAOYSA-N	8.1	DrugBank, MOAD
4-*O*-β-D-Glucosyl vanillic acid	P01556	0.933	VCCMGHVCRFMITI-SVNGYHJRSA-N	A	DrugBank
4-*O*-β-D-Glucosyl vanillic acid	P01556	0.817	BYSXBFJVGIOFBO-BZNQNGANSA-N	A	DrugBank
4-*O*-β-D-Glucosyl vanillic acid	P07464	0.935	IFBHRQDFSNCLOZ-SVNGYHJRSA-N	A	DrugBank
4-*O*-β-D-Glucosyl vanillic acid	Q700S9	0.935	IFBHRQDFSNCLOZ-SVNGYHJRSA-N	A	DrugBank
4-*O*-β-D-Glucosyl vanillic acid	Q700S9	0.928	KUWPCJHYPSUOFW-SVNGYHJRSA-N	A	DrugBank
4-*O*-β-D-Glucosyl vanillic acid	Q9RIK9	0.812	UFSBFVZQJZMIOU-IYKVGLELSA-N	A	DrugBank
4-*O*-β-D-Glucosyl vanillic acid	Q9XBQ3	0.832	DUYYBTBDYZXISX-UKKRHICBSA-N	A	DrugBank
4-*O*-β-D-Glucosyl vanillic acid	Q46829	0.812	UFSBFVZQJZMIOU-LZQZFOIKSA-N	A	DrugBank
4-*O*-β-D-Glucosyl vanillic acid	P31639	0.768	IZNJVARPFYJAJF-FQBWVUSXSA-N	6.54	ChemblDB, PubChem
4-*O*-β-D-Glucosyl vanillic acid	P31639	0.768	NCGITCMKOZTTKR-FQBWVUSXSA-N	5.92	ChemblDB, PubChem
4-*O*-β-D-Glucosyl vanillic acid	P32890	0.933	VCCMGHVCRFMITI-SVNGYHJRSA-N	A	DrugBank
4-*O*-β-D-Glucosyl vanillic acid	Q6UWM7	0.935	IXFOBQXJWRLXMD-ZIQFBCGOSA-N	A	DrugBank
4-*O*-β-D-Glucosyl vanillic acid	P03023	0.785	SWRPIVXPHLYETN-BVWHHUJWSA-N	A	DrugBank
3-(Acetylamino)-4-hydroxy-benzoic acid	P03472	1	MJMLUICFHWSBQZ-UHFFFAOYSA-N	A	DrugBank
3-(Acetylamino)-4-hydroxy-benzoic acid	P03472	0.861	CALDTVBHJMBRTM-UHFFFAOYSA-N	A	DrugBank
β-Sitosterol	P08183	0.947	HVYWMOMLDIMFJA-MFYRMPRMSA-N	A	DrugBank
β-Sitosterol	Q9UNQ0	0.947	HVYWMOMLDIMFJA-MFYRMPRMSA-N	A	DrugBank
β-Sitosterol	P11511	0.831	UYZADDOVTMAOQB-QTOSTRNKSA-N	6.8	BindingDB, ChemblDB, PubChem
β-Sitosterol	P11511	0.831	UYZADDOVTMAOQB-SUAWPXJKSA-N	6.8	BindingDB, ChemblDB, PubChem
β-Sitosterol	P11511	0.757	PFMMKGGPOLDMEE-NYYHRPKISA-N	5.77	BindingDB, ChemblDB, PubChem
β-Sitosterol	P11511	0.757	PFMMKGGPOLDMEE-XRMCMGSZSA-N	5.99	BindingDB, ChemblDB, PubChem

Table S3. *Cont.*

MOLID	UNIPROT	SIM	REF_NN	REF_PACT	SOURCE_DB
β-Sitosterol	Q9UBM7	0.947	KALVPLHXPXPTAM-ZBTTZMLFSA-N	8.4	PubChem
β-Sitosterol	Q9UBM7	0.903	ABMKCJQMQXHKCT-NZXYOJQWSA-N	5.7	PubChem
β-Sitosterol	P18405	0.756	XUTZDXHKQDPUMA-PSULBJLCSA-N	9.18	PubChem
β-Sitosterol	P18405	0.756	XUTZDXHKQDPUMA-RTHGOUKRSA-N	9.18	BindingDB, ChemblDB
β-Sitosterol	P18405	0.756	XUTZDXHKQDPUMA-WOCATEOUSA-N	9.05	BindingDB
β-Sitosterol	P31213	0.756	XUTZDXHKQDPUMA-PSULBJLCSA-N	6.81	PubChem
β-Sitosterol	P31213	0.756	XUTZDXHKQDPUMA-RTHGOUKRSA-N	6.81	BindingDB, ChemblDB
β-Sitosterol	P31213	0.756	XUTZDXHKQDPUMA-WOCATEOUSA-N	6.81	BindingDB
β-Sitosterol	P06276	0.951	BBTIMXAYZRWPNG-VJSFXXLFSA-N	5.42	BindingDB, ChemblDB, PubChem
β-Sitosterol	P35398	0.947	HVYWMOMLDIMFJA-MFYRMPRMSA-N	A	DrugBank
β-Sitosterol	Q13133	0.947	HVYWMOMLDIMFJA-VUDDDUNTSA-N	4.82	PubChem, ChemblDB
β-Sitosterol	Q13133	0.827	OSENKJZWYQXHBN-ABBSENGZSA-N	6.27	PubChem, ChemblDB
β-Sitosterol	Q13133	0.827	OSENKJZWYQXHBN-KRRUWTDCSA-N	6.3	NRacl
β-Sitosterol	Q13133	0.827	OSENKJZWYQXHBN-PJXSLZQESA-N	6.17	PubChem, ChemblDB
β-Sitosterol	Q13133	0.827	OSENKJZWYQXHBN-UHFFFAOYSA-N	5.4	IUPHARdb
β-Sitosterol	Q13133	0.827	OSENKJZWYQXHBN-XVYZBDJZSA-N	6.34	PubChem, ChemblDB
β-Sitosterol	P55055	0.947	HVYWMOMLDIMFJA-VUDDDUNTSA-N	4.82	PubChem, ChemblDB
β-Sitosterol	P55055	0.827	OSENKJZWYQXHBN-UHFFFAOYSA-N	5.52	IUPHARdb
β-Sitosterol	P55055	0.827	OSENKJZWYQXHBN-XVYZBDJZSA-N	6.47	PubChem, ChemblDB
β-Sitosterol	Q96RI1	0.892	FSLPMRQHCOLESF-SFMCKYFRSA-N	5.17	PubChem
β-Sitosterol	P10275	0.947	HVYWMOMLDIMFJA-DPAQBDIFSA-N	5.37	ChemblDB
Maltol glucoside	P22303	0.922	OLZAGZCCJJBKNZ-UJPOAAIJSA-N	6.24	PubChem, BindingDB, ChemblDB
Maltol glucoside	P22303	0.775	FNGTXIQGXDOBSN-ZBXJEJADSA-N	6.6	PubChem, BindingDB, ChemblDB
Maltol glucoside	P22303	0.772	QGBRWWHJQCFYEI-KKOKHZNYSA-N	6.5	PubChem, BindingDB, ChemblDB
Maltol glucoside	P32890	0.892	MIAKOEWBCMPCQR-RMPHRYRLSA-N	A	DrugBank
1,2-Benzenedicarboxylic acid diisobutyl ester	P48147	0.769	GDXKWQUHCWPVIF-LBPRGKRZSA-N	7.43	PubChem, BindingDB, ChemblDB
1,2,3,4,6-Penta-*O*-galloyl-β-D-glucose	P26663	0.818	RATQVALKDAUZBW-UEKZKNBCSA-N	6.12	ChemblDB, PubChem
1,2,3,4,6-Penta-*O*-galloyl-β-D-glucose	P26663	0.818	RATQVALKDAUZBW-UHFFFAOYSA-N	6.12	BindingDB
1,2,3,4,6-Tetra-*O*-galloyl-β-D-glucose	P00742	0.912	IYMHVUYNBVWXKH-ZITZVVOASA-N	6.12	BindingDB, ChemblDB, PubChem
1,2,3,4,6-Tetra-*O*-galloyl-β-D-glucose	P00742	0.875	CHBITXAMNKHJCR-JNUHSSLSSA-N	6.24	BindingDB, ChemblDB, PubChem
1,2,3,4,6-Tetra-*O*-galloyl-β-D-glucose	P00742	0.875	CHBITXAMNKHJCR-VGYCLGPVSA-N	6.36	BindingDB, ChemblDB, PubChem
1,2,3,4,6-Tetra-*O*-galloyl-β-D-glucose	P00734	0.912	IYMHVUYNBVWXKH-ZITZVVOASA-N	6.77	BindingDB, ChemblDB, PubChem
1,2,3,4,6-Tetra-*O*-galloyl-β-D-glucose	P00734	0.875	CHBITXAMNKHJCR-JNUHSSLSSA-N	6.55	BindingDB, ChemblDB, PubChem
1,2,3,4,6-Tetra-*O*-galloyl-β-D-glucose	P00734	0.875	CHBITXAMNKHJCR-VGYCLGPVSA-N	7.3	BindingDB, ChemblDB, PubChem
1,2,3,4,6-Tetra-*O*-galloyl-β-D-glucose	Q14534	0.912	IYMHVUYNBVWXKH-ZITZVVOASA-N	5.7	ChemblDB, PubChem

Table S3. *Cont.*

MOLID	UNIPROT	SIM	REF_NN	REF_PACT	SOURCE_DB
1,2,3,4,6-Tetra-*O*-galloyl-β-D-glucose	Q14534	0.762	LLENXGNWVNSBQG-VFTFQOQOSA-N	6.2	BindingDB, ChemblDB, PubChem
1,2,3,4,6-Tetra-*O*-galloyl-β-D-glucose	P26663	0.979	RATQVALKDAUZBW-UEKZKNBCSA-N	6.12	ChemblDB, PubChem
1,2,3,4,6-Tetra-O-galloyl-β-D-glucose	P26663	0.979	RATQVALKDAUZBW-UHFFFAOYSA-N	6.12	BindingDB
1,2,3,4,6-Tetra-*O*-galloyl-β-D-glucose	P26663	0.762	LLENXGNWVNSBQG-UHFFFAOYSA-N	5.72	BindingDB
1,2,3,4,6-Tetra-*O*-galloyl-β-D-glucose	P26663	0.762	LLENXGNWVNSBQG-VFTFQOQOSA-N	5.72	ChemblDB, PubChem
2,3,4,6-Tetra-*O*-galloyl-α-D-glucose	P00742	0.979	IYMHVUYNBVWXKH-ZITZVVOASA-N	6.12	BindingDB, ChemblDB, PubChem
2,3,4,6-Tetra-*O*-galloyl-α-D-glucose	P00742	0.876	CHBITXAMNKHJCR-JNUHSSLSSA-N	6.24	BindingDB, ChemblDB, PubChem
2,3,4,6-Tetra-*O*-galloyl-α-D-glucose	P00742	0.876	CHBITXAMNKHJCR-VGYCLGPVSA-N	6.36	BindingDB, ChemblDB, PubChem
2,3,4,6-Tetra-*O*-galloyl-α-D-glucose	P00734	0.979	IYMHVUYNBVWXKH-ZITZVVOASA-N	6.77	BindingDB, ChemblDB, PubChem
2,3,4,6-tetra-*O*-galloyl-α-D-glucose	P00734	0.876	CHBITXAMNKHJCR-JNUHSSLSSA-N	6.55	BindingDB, ChemblDB, PubChem
2,3,4,6-Tetra-*O*-galloyl-α-D-glucose	P00734	0.876	CHBITXAMNKHJCR-VGYCLGPVSA-N	7.3	BindingDB, ChemblDB, PubChem
2,3,4,6-Tetra-*O*-galloyl-α-D-glucose	Q14534	0.979	IYMHVUYNBVWXKH-ZITZVVOASA-N	5.7	ChemblDB, PubChem
2,3,4,6-Tetra-*O*-galloyl-α-D-glucose	P26663	0.956	RATQVALKDAUZBW-UEKZKNBCSA-N	6.12	ChemblDB, PubChem
2,3,4,6-Tetra-*O*-galloyl-α-D-glucose	P26663	0.956	RATQVALKDAUZBW-UHFFFAOYSA-N	6.12	BindingDB
5-Ethoxy-3-hydroxy-benzoate	P35869	0.855	PWOARNMOPCOJEV-UHFFFAOYSA-N	5.13	PubChem
Protocatechuic acid	P04181	0.858	XFDUHJPVQKIXHO-UHFFFAOYSA-N	A	DrugBank
Protocatechuic acid	P23893	0.858	XFDUHJPVQKIXHO-UHFFFAOYSA-N	A	DrugBank
Protocatechuic acid	P08183	0.858	BTJIUGUIPKRLHP-UHFFFAOYSA-N	A	DrugBank
Protocatechuic acid	Q9Y6F1	0.858	XFDUHJPVQKIXHO-UHFFFAOYSA-N	5.2	BindingDB, ChemblDB, PubChem
Protocatechuic acid	P0A7G6	0.928	WJXSWCUQABXPFS-UHFFFAOYSA-N	5.27	PubChem
Protocatechuic acid	P0A7G6	0.901	UONVFNLDGRWLKF-UHFFFAOYSA-N	5.21	PubChem
Protocatechuic acid	P46952	0.822	VWEPFJPQZFIOAU-UHFFFAOYSA-N	A	DrugBank
Protocatechuic acid	P09917	0.901	KBOPZPXVLCULAV-UHFFFAOYSA-N	A	DrugBank
Protocatechuic acid	P09917	0.887	WUBBRNOQWQTFEX-UHFFFAOYSA-N	A	DrugBank
Protocatechuic acid	P20586	COLL	YQUVCSBJEUQKSH-UHFFFAOYSA-N	5.34	MOAD, affinDB
Protocatechuic acid	P20586	0.887	UIAFKZKHHVMJGS-UHFFFAOYSA-N	A	DrugBank
Protocatechuic acid	P20586	0.858	ALYNCZNDIQEVRV-UHFFFAOYSA-N	3.83	MOAD, DrugBank
Protocatechuic acid	P20586	0.858	FJKROLUGYXJWQN-UHFFFAOYSA-N	4.49	MOAD, DrugBank, affinDB
Protocatechuic acid	Q81VW8	0.858	ALYNCZNDIQEVRV-UHFFFAOYSA-N	5.25	ChemblDB, PubChem
Protocatechuic acid	P26281	0.887	WUBBRNOQWQTFEX-UHFFFAOYSA-N	A	DrugBank
Protocatechuic acid	P40871	0.928	GLDQAMYCGOIJDV-UHFFFAOYSA-N	A	DrugBank
Protocatechuic acid	Q12851	0.901	UONVFNLDGRWLKF-UHFFFAOYSA-N	5.42	PubChem
Protocatechuic acid	O15111	0.901	KBOPZPXVLCULAV-UHFFFAOYSA-N	A	DrugBank
Protocatechuic acid	O15111	0.887	WUBBRNOQWQTFEX-UHFFFAOYSA-N	A	DrugBank
Protocatechuic acid	O14920	0.901	KBOPZPXVLCULAV-UHFFFAOYSA-N	A	DrugBank
Protocatechuic acid	P50225	0.858	BTJIUGUIPKRLHP-UHFFFAOYSA-N	A	DrugBank

Table S3. *Cont.*

MOLID	UNIPROT	SIM	REF_NN	REF_PACT	SOURCE_DB
Protocatechuic acid	Q9NZK7	0.887	WUBBRNOQWQTFEX-UHFFFAOYSA-N	A	DrugBank
Protocatechuic acid	Q04416	0.858	FJKROLUGYXJWQN-UHFFFAOYSA-N	A	DrugBank
Protocatechuic acid	P19961	0.858	BTJIUGUIPKRLHP-UHFFFAOYSA-N	A	DrugBank
Protocatechuic acid	Q8I2J3	COLL	YQUVCSBJEUQKSH-UHFFFAOYSA-N	6.32	PubChem
Protocatechuic acid	P26602	0.858	FJKROLUGYXJWQN-UHFFFAOYSA-N	A	DrugBank
Protocatechuic acid	O43570	0.858	FJKROLUGYXJWQN-UHFFFAOYSA-N	5.26	ChemblDB, PubChem, BindingDB
Protocatechuic acid	O43570	0.832	LNTHITQWFMADLM-UHFFFAOYSA-N	5.11	ChemblDB, PubChem, BindingDB
Protocatechuic acid	P23280	0.858	FJKROLUGYXJWQN-UHFFFAOYSA-N	5.09	ChemblDB, BindingDB, PubChem
Protocatechuic acid	P23280	0.832	LNTHITQWFMADLM-UHFFFAOYSA-N	5.21	ChemblDB, BindingDB, PubChem
Protocatechuic acid	P35218	0.858	FJKROLUGYXJWQN-UHFFFAOYSA-N	5.44	ChemblDB, BindingDB, PubChem
Protocatechuic acid	P35218	0.832	LNTHITQWFMADLM-UHFFFAOYSA-N	5.39	ChemblDB, BindingDB, PubChem
Protocatechuic acid	Q99N23	0.858	FJKROLUGYXJWQN-UHFFFAOYSA-N	5.03	ChemblDB, PubChem
Protocatechuic acid	Q9Y2D0	0.858	FJKROLUGYXJWQN-UHFFFAOYSA-N	5.07	ChemblDB, BindingDB, PubChem
Protocatechuic acid	Q9Y2D0	0.832	LNTHITQWFMADLM-UHFFFAOYSA-N	5	ChemblDB, BindingDB, PubChem
Protocatechuic acid	Q51792	0.928	WJXSWCUQABXPFS-UHFFFAOYSA-N	5.85	DrugBank, MOAD
Protocatechuic acid	P37231	0.901	KBOPZPXVLCULAV-UHFFFAOYSA-N	A	DrugBank
Protocatechuic acid	Q07820	0.901	UONVFNLDGRWLKF-UHFFFAOYSA-N	5.8	PubChem
Protocatechuic acid	P80188	0.928	GLDQAMYCGOIJDV-UHFFFAOYSA-N	8.1	DrugBank, MOAD
Prunin	Q9UNQ0	0.848	KYQZWONCHDNPDP-QNDFHXLGSA-N	A	DrugBank
Prunin	P05113	0.837	ISQRJFLLIDGZEP-CMWLGVBASA-N	5.85	BindingDB, PubChem
Prunin	P15121	0.789	QSLQKIQXZKDLIH-GKARDXTASA-N	4.68	ChemblDB
Prunin	P15121	0.779	TXKFRRCKZWJXBW-GPRNFGOXSA-N	5.5	BindingDB, ChemblDB, PubChem
Prunin	P15121	0.777	GLTCTFBPNQJRQT-PBTMSNHXSA-N	5.09	ChemblDB
Prunin	P05091	0.848	KYQZWONCHDNPDP-QNDFHXLGSA-N	A	DrugBank
Prunin	P09923	0.769	AEDDIBAIWPIIBD-UHFFFAOYSA-N	5.92	PubChem
Prunin	P10696	0.769	AEDDIBAIWPIIBD-UHFFFAOYSA-N	5.57	PubChem
Prunin	P14416	0.793	OEUGQYOMKCJJLJ-UHFFFAOYSA-N	5.28	BindingDB, ChemblDB, PubChem
Prunin	P21917	0.793	OEUGQYOMKCJJLJ-LMCMXOCHSA-N	A	hGPCRlig
Prunin	P21917	0.793	OEUGQYOMKCJJLJ-UHFFFAOYSA-N	5.12	ChemblDB, BindingDB, PubChem
Prunin	P03372	0.819	HSWIRQIYASIOBE-JNHRPPPUSA-N	5.24	ChemblDB, PubChem
Prunin	P03372	0.819	HSWIRQIYASIOBE-UHFFFAOYSA-N	5.24	BindingDB
Prunin	P13866	0.954	KOTXSQPZNZHNFC-UHFFFAOYSA-N	6.25	BindingDB, ChemblDB, PubChem
Prunin	P13866	0.929	GOTAZLUFLPHQJU-UHFFFAOYSA-N	5.55	BindingDB, ChemblDB, PubChem
Prunin	P13866	0.901	IOUVKUPGCMBWBT-QNDFHXLGSA-N	6.59	BindingDB, ChemblDB, PubChem
Prunin	P13866	0.901	IOUVKUPGCMBWBT-UHFFFAOYSA-N	6.75	BindingDB, ChemblDB, PubChem
Prunin	P13866	0.863	IWRUKKIVIXIBRH-DODNOZFWSA-N	5.33	BindingDB, ChemblDB, PubChem

Table S3. *Cont.*

MOLID	UNIPROT	SIM	REF_NN	REF_PACT	SOURCE_DB
Prunin	P13866	0.863	JSFDGGQKOKTSOU-PFKOEMKTSA-N	4.87	BindingDB, ChemblDB, PubChem
Prunin	P13866	0.863	RMQQQPQAVFICPZ-PFKOEMKTSA-N	5.8	BindingDB, ChemblDB, PubChem
Prunin	P13866	0.858	CLRQMIQNMTYMGA-XIKSMUEASA-N	5.86	BindingDB, ChemblDB, PubChem
Prunin	P13866	0.858	GMYFQAHYWIYNES-PFKOEMKTSA-N	6.74	BindingDB, ChemblDB, PubChem
Prunin	P13866	0.858	VAVAYLQBNLAMRO-XIKSMUEASA-N	5.36	BindingDB, ChemblDB, PubChem
Prunin	P13866	0.855	DKUVOIUBCISXDG-UHFFFAOYSA-N	5.58	BindingDB, ChemblDB, PubChem
Prunin	P13866	0.847	ACMMSHORHBTOEZ-PRDVQWLOSA-N	5.08	BindingDB, ChemblDB, PubChem
Prunin	P13866	0.844	IINBYKILNZBSAK-PFKOEMKTSA-N	5.84	BindingDB, ChemblDB, PubChem
Prunin	P13866	0.844	KPTNFLTZJSDNHO-PFKOEMKTSA-N	4.79	BindingDB, ChemblDB, PubChem
Prunin	P13866	0.844	LBKNLPSWXKBZPU-DODNOZFWSA-N	5.43	BindingDB, ChemblDB, PubChem
Prunin	P13866	0.844	QFUQUZDKFKPDFP-UHFFFAOYSA-N	4.35	BindingDB, ChemblDB, PubChem
Prunin	P13866	0.842	HZQBMUPOYJUQAR-XDXGNBCUSA-N	5.88	BindingDB, ChemblDB, PubChem
Prunin	P13866	0.842	QAJZQZIOIFEMTH-PRDVQWLOSA-N	5.32	BindingDB, ChemblDB, PubChem
Prunin	P13866	0.842	WKPBVEUJSMWTPM-XDXGNBCUSA-N	5	BindingDB, ChemblDB, PubChem
Prunin	P13866	0.842	XVBIDTMBHOOAMG-PRDVQWLOSA-N	5.43	BindingDB, ChemblDB, PubChem
Prunin	P13866	0.84	WQCWELFQKXIPCN-UTCJRWHESA-N	2.5	BindingDB, ChemblDB, PubChem
Prunin	P13866	0.834	ZLMFBIABOBCOTJ-UHFFFAOYSA-N	5.42	BindingDB, ChemblDB, PubChem
Prunin	P13866	0.83	FMFDIEUZQZXVQG-XDXGNBCUSA-N	4.48	BindingDB, ChemblDB, PubChem
Prunin	P13866	0.83	JVBJTLHQLXTUOY-UIKHAHSZSA-N	5.19	BindingDB, ChemblDB, PubChem
Prunin	P13866	0.827	NLZYYMHUDULKQF-PRDVQWLOSA-N	5.2	BindingDB, ChemblDB, PubChem
Prunin	P13866	0.819	RFZGXHLLXXDQJN-UHFFFAOYSA-N	4.64	BindingDB, ChemblDB, PubChem
Prunin	P13866	0.784	FINNPFQSLRPSQZ-UHFFFAOYSA-N	4.03	BindingDB, ChemblDB, PubChem
Prunin	P13866	0.779	BJEOSUUCUHNCLB-DODNOZFWSA-N	3.85	BindingDB, ChemblDB, PubChem
Prunin	P13866	0.779	RTCQWPDTJIXOFG-DODNOZFWSA-N	3.85	BindingDB, ChemblDB, PubChem
Prunin	P13866	0.774	RKVRUEAVIWWMKJ-PRDVQWLOSA-N	3.85	BindingDB, ChemblDB, PubChem
Prunin	P31639	0.901	IOUVKUPGCMBWBT-QNDFHXLGSA-N	7.18	BindingDB, ChemblDB, PubChem
Prunin	P31639	0.863	IWRUKKIVIXIBRH-DODNOZFWSA-N	7.1	BindingDB, ChemblDB, PubChem
Prunin	P31639	0.863	JSFDGGQKOKTSOU-PFKOEMKTSA-N	5.89	BindingDB, ChemblDB, PubChem
Prunin	P31639	0.863	RMQQQPQAVFICPZ-PFKOEMKTSA-N	8	BindingDB, ChemblDB, PubChem
Prunin	P31639	0.858	CLRQMIQNMTYMGA-XIKSMUEASA-N	7.16	BindingDB, ChemblDB, PubChem
Prunin	P31639	0.858	GMYFQAHYWIYNES-PFKOEMKTSA-N	8.11	BindingDB, ChemblDB, PubChem
Prunin	P31639	0.858	VAVAYLQBNLAMRO-XIKSMUEASA-N	6.64	BindingDB, ChemblDB, PubChem
Prunin	P31639	0.847	ACMMSHORHBTOEZ-PRDVQWLOSA-N	7.52	BindingDB, ChemblDB, PubChem
Prunin	P31639	0.844	IINBYKILNZBSAK-PFKOEMKTSA-N	8.05	BindingDB, ChemblDB, PubChem
Prunin	P31639	0.844	KPTNFLTZJSDNHO-PFKOEMKTSA-N	6.54	BindingDB, ChemblDB, PubChem
Prunin	P31639	0.844	LBKNLPSWXKBZPU-DODNOZFWSA-N	7.7	BindingDB, ChemblDB, PubChem

Table S3. *Cont.*

MOLID	UNIPROT	SIM	REF_NN	REF_PACT	SOURCE_DB
Prunin	P31639	0.842	HZQBMUPOYJUQAR-XDXGNBCUSA-N	8	BindingDB, ChemblDB, PubChem
Prunin	P31639	0.842	QAJZQZIOIFEMTH-PRDVQWLOSA-N	7.52	BindingDB, ChemblDB, PubChem
Prunin	P31639	0.842	WKPBVEUJSMWTPM-XDXGNBCUSA-N	6.28	BindingDB, ChemblDB, PubChem
Prunin	P31639	0.842	XVBIDTMBHOOAMG-PRDVQWLOSA-N	7.16	BindingDB, ChemblDB, PubChem
Prunin	P31639	0.833	JIQDXQSGNNHQOD-DODNOZFWSA-N	5.73	BindingDB, ChemblDB, PubChem
Prunin	P31639	0.83	FMFDIEUZQZXVQG-XDXGNBCUSA-N	6.96	BindingDB, ChemblDB, PubChem
Prunin	P31639	0.83	JVBJTLHQLXTUOY-UIKHAHSZSA-N	8	BindingDB, ChemblDB, PubChem
Prunin	P31639	0.827	NLZYYMHUDULKQF-PRDVQWLOSA-N	8	BindingDB, ChemblDB, PubChem
Prunin	P31639	0.779	BJEOSUUCUHNCLB-DODNOZFWSA-N	5.27	BindingDB, ChemblDB, PubChem
Prunin	P31639	0.779	RTCQWPDTJIXOFG-DODNOZFWSA-N	5.27	BindingDB, ChemblDB, PubChem
Prunin	P31639	0.774	RKVRUEAVIWWMKJ-PRDVQWLOSA-N	5.27	BindingDB, ChemblDB, PubChem
Kaempferol-3-*O*-α-L-rhamnoside-7-*O*-β-D-glucoside	P61088	0.87	DFPMSGMNTNDNHN-ZFOFJSCHSA-N	5.08	PubChem
Kaempferol-3-*O*-α-L-rhamnoside-7-*O*-β-D-glucoside	P61088	0.87	DFPMSGMNTNDNHN-ZHNJBYEHSA-N	5.12	PubChem
Kaempferol-3-*O*-α-L-rhamnoside-7-*O*-β-D-glucoside	P11511	0.896	SXNOCVMJOZRSLS-MCEICCLHSA-N	5.3	BindingDB
Kaempferol-3-*O*-α-L-rhamnoside-7-*O*-β-D-glucoside	P11511	0.87	DFPMSGMNTNDNHN-ZHNJBYEHSA-N	5.3	PubChem
Kaempferol-3-*O*-α-L-rhamnoside-7-*O*-β-D-glucoside	P11511	0.87	DFPMSGMNTNDNHN-ZPHOTFPESA-N	5.3	BindingDB, ChemblDB
Kaempferol-3-*O*-α-L-rhamnoside-7-*O*-β-D-glucoside	O76074	0.784	TZJALUIVHRYQQB-NZDRBQIJSA-N	5.23	BindingDB
Kaempferol-3-*O*-α-L-rhamnoside-7-*O*-β-D-glucoside	O76074	0.784	TZJALUIVHRYQQB-XLRXWWTNSA-N	5.23	ChemblDB, PubChem
Aureusidin-6-*O*-neohesperidoside	P61088	0.856	DFPMSGMNTNDNHN-ZFOFJSCHSA-N	5.08	PubChem
Aureusidin-6-*O*-neohesperidoside	P61088	0.856	DFPMSGMNTNDNHN-ZHNJBYEHSA-N	5.12	PubChem
Aureusidin-6-*O*-neohesperidoside	P11712	0.885	IKGXIBQEEMLURG-BKUODXTLSA-N	A	DrugBank
Aureusidin-6-*O*-neohesperidoside	P10632	0.885	IKGXIBQEEMLURG-BKUODXTLSA-N	A	DrugBank
Aureusidin-6-*O*-neohesperidoside	P10635	0.885	IKGXIBQEEMLURG-BKUODXTLSA-N	A	DrugBank
Aureusidin-6-*O*-neohesperidoside	P11511	0.856	DFPMSGMNTNDNHN-ZHNJBYEHSA-N	5.3	PubChem
Aureusidin-6-*O*-neohesperidoside	P11511	0.856	DFPMSGMNTNDNHN-ZPHOTFPESA-N	5.3	BindingDB, ChemblDB
Aureusidin-6-*O*-neohesperidoside	P42330	0.885	IKGXIBQEEMLURG-BKUODXTLSA-N	A	DrugBank
Aureusidin-6-*O*-neohesperidoside	P08912	0.885	IKGXIBQEEMLURG-UHFFFAOYSA-N	7.45	PDSP

Table S3. *Cont.*

MOLID	UNIPROT	SIM	REF_NN	REF_PACT	SOURCE_DB
Aureusidin-6-*O*-neohesperidoside	P08913	0.885	IKGXIBQEEMLURG-NVPNHPEKSA-N	8.05	BindingDB, PubChem, ChemblDB
Aureusidin-6-*O*-neohesperidoside	P08913	0.885	IKGXIBQEEMLURG-UHFFFAOYSA-N	8.05	PDSP
Aureusidin-6-*O*-neohesperidoside	P18825	0.885	IKGXIBQEEMLURG-NVPNHPEKSA-N	8.05	BindingDB, ChemblDB, PubChem
Aureusidin-6-*O*-neohesperidoside	P18825	0.885	IKGXIBQEEMLURG-UHFFFAOYSA-N	6.54	PDSP

Table S4. The Protein Functional Families.

Abbreviation_Name	Full_Name	Abbreviation_Name	Full_Name
AA	Amino acid related enzymes	GT	Glycosyltransferases
AN	Cellular antigens	IC	Ion channels
BM	Bacterial motility proteins	KC	Protein kinases
BQ	Ubiquitin system	LD	Lipid biosynthesis proteins
BT	Bacterial toxins	LP	Lipopolysaccharide biosynthesis proteins
CA	Cell adhesion molecules (CAMs)	NR	Nuclear receptors
CH	Chromosome	PA	Proteasome
CK	Cytoskeleton proteins	PG	Proteoglycans
CL	CAM ligands	PH	Photosynthesis proteins
CP	Cytochrome P450	PS	Peptidases
CR	Cytokine receptors	PT	Prenyltransferases
CY	Cytokines	RS	Ribosome
DL	DNA replication protein	SC	Secretion system
DR	DNA repair and recombination proteins	SN	SNAREs
EC	Enzymes	SS	Spliceosome
EL	Enzyme-linked receptors	TC	Transporters
FD	Chaperones and folding catalysts	TL	Translation factors
GB	GTP-binding proteins	TR	Transcription factors
GL	Glycan binding proteins	TS	Two-component system
GR	G protein-coupled receptors	UC	Unclassified
